# Autophagy protein 5 controls flow-dependent endothelial functions

**DOI:** 10.1007/s00018-023-04859-9

**Published:** 2023-07-18

**Authors:** Pierre Nivoit, Thomas Mathivet, Junxi Wu, Yann Salemkour, Devanarayanan Siva Sankar, Véronique Baudrie, Jennifer Bourreau, Anne-Laure Guihot, Emilie Vessieres, Mathilde Lemitre, Cinzia Bocca, Jérémie Teillon, Morgane Le Gall, Anna Chipont, Estelle Robidel, Neeraj Dhaun, Eric Camerer, Pascal Reynier, Etienne Roux, Thierry Couffinhal, Patrick W. F. Hadoke, Jean-Sébastien Silvestre, Xavier Guillonneau, Philippe Bonnin, Daniel Henrion, Joern Dengjel, Pierre-Louis Tharaux, Olivia Lenoir

**Affiliations:** 1 Inserm, Université Paris Cité, PARCC, 56 Rue Leblanc, 75015 Paris, France; 2 Centre for Cardiovascular Science, The Queen’s Medical Research Institute, University of Edinburgh, Edinburgh, EH16 4TJ UK; 3 Department of Biomedical Engineering, University of Strathclyde, Glasgow, G4 ONW UK; 4 Department of Biology, University of Fribourg, 1700 Fribourg, Switzerland; 5 MITOVASC, CNRS UMR 6015, Inserm U1083, Université d’Angers, 49500 Angers, France; 6 Département de Biochimie et Biologie Moléculaire, Centre Hospitalier Universitaire d’Angers, 49500 Angers, France; 7 CNRS, Inserm, Bordeaux Imaging Center, BIC, UMS 3420, US 4, Université de Bordeaux, 33000 Bordeaux, France; 8 Plateforme Protéomique 3P5-Proteom’IC, Institut Cochin, INSERM U1016, CNRS UMR8104, Université Paris Cité, 75014 Paris, France; 9 Inserm, Biologie Des Maladies Cardiovasculaires, U1034, Université de Bordeaux, 33600 Pessac, France; 10 Institut de La Vision, INSERM, CNRS, Sorbonne Université, 75012 Paris, France; 11 AP-HP, Hôpital Lariboisière, Physiologie Clinique - Explorations Fonctionnelles, Hypertension Unit, Université Paris Cité, 75010 Paris, France

**Keywords:** Autophagy, Endothelium, Flow-mediated dilatation, Mechanosensing, eNOS, VEGFR2

## Abstract

Dysregulated autophagy is associated with cardiovascular and metabolic diseases, where impaired flow-mediated endothelial cell responses promote cardiovascular risk. The mechanism by which the autophagy machinery regulates endothelial functions is complex. We applied multi-omics approaches and in vitro and in vivo functional assays to decipher the diverse roles of autophagy in endothelial cells. We demonstrate that autophagy regulates VEGF-dependent VEGFR signaling and VEGFR-mediated and flow-mediated eNOS activation. Endothelial ATG5 deficiency in vivo results in selective loss of flow-induced vasodilation in mesenteric arteries and kidneys and increased cerebral and renal vascular resistance in vivo. We found a crucial pathophysiological role for autophagy in endothelial cells in flow-mediated outward arterial remodeling, prevention of neointima formation following wire injury, and recovery after myocardial infarction. Together, these findings unravel a fundamental role of autophagy in endothelial function, linking cell proteostasis to mechanosensing.

**Supplementary Information:**

The online version contains supplementary material available at 10.1007/s00018-023-04859-9.

## Introduction

The major roles of autophagy are to provide metabolic precursors for survival under stress conditions and to serve quality control by clearing misfolded proteins and other cellular debris [1–3]. Aging is often associated with a reduced autophagic potential [2, 4], and dysregulation of autophagy is associated with various metabolic and age-related diseases, including cancer, autoimmune and inflammatory diseases, diabetes mellitus, kidney diseases, atherosclerosis, and infections [4–11]. Furthermore, pharmacological or genetic manipulations that increase the life span in model organisms often stimulate autophagy [4, 12–14]. We previously demonstrated that the autophagy gene, *Atg5*, is essential in endothelial cells to maintain kidney microvascular integrity during diabetes mellitus in mice [15]. Endothelial-specific ATG5 deficiency was also found to attenuate ischemia-related angiogenesis and aggravate experimental atherosclerosis [16, 17]. Thus, autophagy is a major protective factor in endothelial cells against metabolic stress and accelerated vascular aging, although through unclear mechanisms.

Blood flow-elicited “shear stress” promotes vessel dilation with a direct effect on endothelial cells. Shear stress increases the activity of eNOS and the cyclooxygenases (COX), and their respective products, NO and PGI_2,_ relax blood vessels [18]. A decrease in endothelial NO production or bioavailability may impair tissue perfusion, leading to accelerated aging associated with hypertension, obesity, and diabetes mellitus. Transduction of the flow sensing also involves the angiogenic signaling machinery that regulates essential endothelial functions such as proliferation, migration, or permeability [19, 20]. The PECAM-1/VE-cadherin/VEGFR2 complex at cell–cell junctions is one important endothelial-specific flow sensor [21]. VEGFR2 is a tyrosine kinase receptor that mediates most of the mitogenic and chemotactic effects of the VEGF. Activated VEGFR2 signals through multiple downstream pathways, including PI3K-AKT [22], in turn mediates the activation of eNOS and COX production [23].

Despite increasing studies on the role of autophagy in the endothelium, its role as a modulator of endothelial cell responses is still controversial, probably because it is likely dependent on the type of metabolic stress explored and the design of experiments (reviewed in [24]). Furthermore, the links between autophagy and fundamental endothelial functions are not well defined. The role of autophagy at the crossroad between nutritional factors that are disturbed in cardiovascular diseases and the concomitance of dysfunction in autophagy in the endothelium and endothelium-mediated vasodilatation during physiological and accelerated aging led us to ask if reduced endothelial autophagy directly impairs endothelial mechanosensing. To this end, we analyzed the effects of selective endothelial deletion of *Atg5* on baseline endothelial cell function and under various conditions associated with vascular stress in vivo.

Transcriptomic, proteomic, and metabolomic analyses in combination with autophagosome content profiling highlighted that autophagy deficiency in endothelial cells led to mitochondrial dysfunction and promoted a complete change in endothelial differentiation, impairing endothelial mechanosensing. We show that endothelial ATG5 plays a critical role in both fluid shear stress-induced and VEGF-induced eNOS activation, thereby controlling vascular tone, tissue perfusion, and adaptive arterial remodeling. We further demonstrate that endothelial autophagy is fundamental to sustain intact VEGFR2 activity, endothelial recovery after injury, and neoangiogenesis.

## Results

### Impaired mitochondrial function and metabolism in ATG5-deficient endothelial cells

**Fig. 1:**
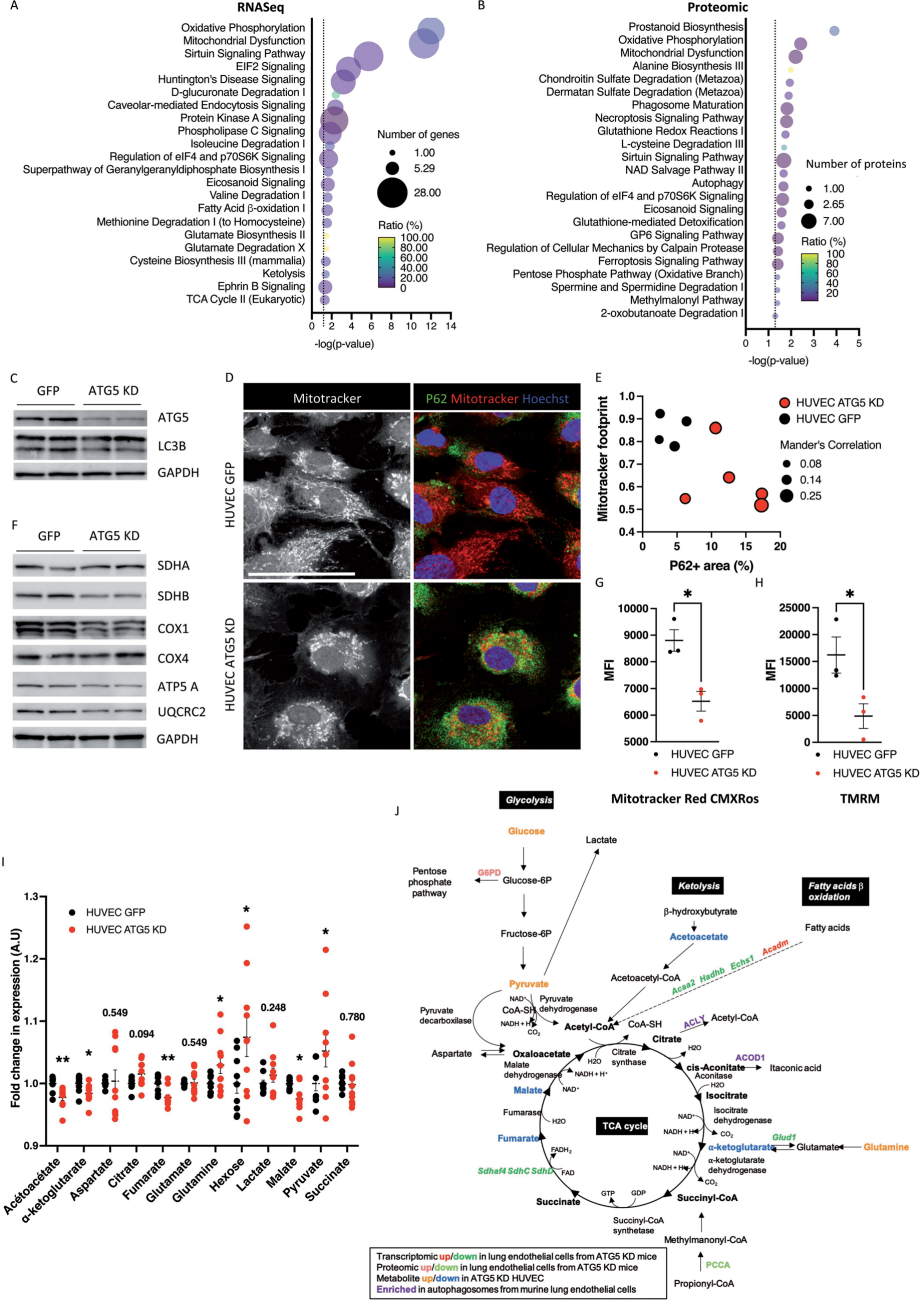
ATG5 deficiency promotes mitochondrial dysfunction and metabolic changes in endothelial cells. **A-B** Selected canonical pathways identified by Ingenuity Pathway Analysis (IPA) performed on differentially expressed genes (**A**) and proteins (**B**) between primary lung endothelial cells isolated from *Atg5*^lox/lox^ and *Cdh5*.Cre *Atg5*^lox/lox^ mice. *n* = 3 replicates. **C** Western blot analysis of the expression of ATG5 and LC3B showing decreased autophagic flux in HUVECs transduced with a lentivirus coding for shRNA against ATG5 (ATG5 KD). GAPDH was used as a loading control. Representative of *n* = 4 replicates. Lanes #1 and #2 represent 2 different protein extracts from 2 different experiments. **D** Immunofluorescence of P62 (green) and mitotracker (red) in control (GFP) and ATG5 KD HUVECs. Scale bar 25 μm. **E** Quantification of the mitochondrial footprint, P62 expression, and Mander’s correlation of the mitochondria staining colocalizing with P62 staining in control and ATG5 KD HUVECs. ATG5 KD HUVECs present P62 accumulation, altered mitochondria architecture, and increased number of mitochondria colocalizing with P62. Each dot represents a different culture well. **F** Western blot analysis of the expression of mitochondrial proteins: SDHA, SDHB, COX1, COX4, ATP5A and UQCRC2. GAPDH was used as a loading control. Representative of *n* = 4 replicates. Lanes #1 and #2 represent 2 different protein extracts from 2 different experiments. **G–H** Flow cytometry analysis of the active pool of mitochondria in control and ATG5 KD HUVECs using mitotracker Red CMXRos (**G**) and TRMR (**H**) stainings. *n* = 3 experimental replicates. **I** Metabolomic detection of TCA intermediates in control and ATG5 KD HUVECs. *n* = 9 experimental replicates. **J** Graphical summary

Efficient CRE-mediated excision of floxed *Atg5* alleles in mice with endothelial-selective *Atg5* deletion was demonstrated [15]. Endothelial autophagy deficiency was confirmed by accumulation of the autophagy degradation product P62 in aortic endothelial cells of endothelial-selective ATG5-deficient mice (*Cdh5*.cre-*Atg5*^lox/lox^ mice) on flat mount aorta (Supp Fig. 1). RNA sequencing of primary lung endothelial cells from wild-type (WT) and endothelial-selective ATG5-deficient mice was performed. We selected 892 differentially expressed genes (DEG) (fold-change > 1.2, *p*-value < 0.05, Supp Table S1), and Ingenuity Pathway Analysis (IPA) was performed. Oxidative phosphorylation, mitochondrial dysfunction, and EIF2 signaling were among the most decreased pathways in *Atg5* KO endothelial cells. At the same time, sirtuin, p53, unfolded protein response, Ca2 + , and phospholipase C signaling cascades were among the most activated pathways (Fig. 1A**, **Table 1, Supp Fig. 2). IPA identified molecular functions in autophagy-deficient cells related to endothelial homeostasis and maintenance, carbohydrate and lipid metabolism, cell organization, Ca2 + signaling and cellular transport (Supp Table S2). Gene ontology (GO) analysis further confirmed dysregulation of genes implicated in biological functions (BF) such as Ca2 + signaling, cell adhesion, mitochondrial electron transport, tyrosine kinase receptor signaling, fatty acid metabolism, transport, and platelet adhesion, among others (Supp Fig. 2). GO cellular components (CC) confirmed deregulation of genes encoding for mitochondrial proteins in ATG5-deficient endothelial cells (Supp Fig. 2). IPA upstream functional analysis was used to predict the top upstream transcriptional regulators from DEGs and predicted transcriptional regulators to be activated such as RICTOR, KDM5A, BDNF, P53 or to be repressed such as STK11, RB1 or IKZF1 (Supp Fig. 2); factors associated with angiogenesis and endothelial function.

**Table 1:** Ingenuity Pathway Analysis (IPA) on RNAseq identified canonical pathways dysregulated in primary endothelial cells from *Cdh5.cre Atg5*^lox/lox^ mice

Ingenuity Canonical Pathways	– log (*p* value)	*p* value	Ratio (%)	z-score	Genes	Number of genes
Oxidative Phosphorylation	12.00	1.00E-12	23.0	– 4.379	Atp5e,ATP5MC1,ATPAF1,COX4I2,COX7A2,CYC1,MT-CO3,MT-ND3,MT-ND4L,NDUFA10,NDUFA11,NDUFA2,NDUFA3,NDUFA4,NDUFA7,NDUFB1,NDUFB6,NDUFV2,SDHC,SDHD,UQCR10,UQCRC1,UQCRQ	23
Mitochondrial Dysfunction	11.30	5.01E-12	17.5	NaN	APH1A,Aph1c,Atp5e,ATP5MC1,ATPAF1,COX4I2,COX7A2,CYC1,FIS1,MT-CO3,MT-ND3,MT-ND4L,NDUFA10,NDUFA11,NDUFA2,NDUFA3,NDUFA4,NDUFA7,NDUFB1,NDUFB6,NDUFV2,PRDX3,SDHC,SDHD,UQCR10,UQCRC1,UQCRQ,VDAC3	28
Sirtuin Signaling Pathway	5.71	1.95E-06	10.1	1.807	ATP5MC1,CYC1,GADD45G,GLUD1,H3C14,MAP1LC3A,MT-ND3,MT-ND4L,NDUFA10,NDUFA11,NDUFA2,NDUFA3,NDUFA4,NDUFA7,NDUFB1,NDUFB6,NDUFV2,PAM16,POLR2F,PPID,SDHC,SDHD,SIRT3,TIMM8A,TOMM22,TSPO,VDAC3	27
EIF2 Signaling	3.69	2.04E-04	9.2	– 3.162	RPL10,RPL15,RPL17,Rpl22l1,RPL24,RPL3,RPL37A,RPL38,RPL39,RPL41,RPS14,RPS18,RPS19,RPS20,RPS25,RPS27L,RPS29,RPS4Y1,RPS5	19
Huntington’s Disease Signaling	3.16	6.92E-04	8.4	NaN	ATP5MC1,CAPN11,CREB5,DNM1,GNB2,GNG10,GNG4,GOSR2,HDAC9,HSPA1A/HSPA1B,Hspa1b,NCOR1,POLR2B,POLR2F,POLR2I,PRKCB,SIN3A,TCERG1,VAMP3	19
D-glucuronate Degradation I	2.41	3.89E-03	66.7	NaN	CRYL1,DCXR	2
Caveolar-mediated Endocytosis Signaling	2.37	4.27E-03	11.3	NaN	ACTG1,CD55,DYRK3,ITGA2B,ITGA4,ITGA9,ITGAL,ITGB5	8
Protein Kinase A Signaling	2.26	5.50E-03	6.4	0.471	AKAP8,ANAPC11,CREB5,CTNNB1,DUSP10,EPM2A,GDE1,GNB2,GNG10,GNG4,H3C14,NFAT5,NFKBID,PDE4A,PPP1R7,PRKCB,PTPN22,PTPN5,PTPN6,PTPRC,PTPRS,PTPRT,YWHAH,YWHAQ	24
Phospholipase C Signaling	1.85	1.41E-02	6.7	3.464	CD3G,CREB5,GNB2,GNG10,GNG4,HDAC9,ITGA4,ITK,LAT,LCK,LCP2,NFAT5,PLA2G6,PRKCB,RHOF,ZAP70	16
Isoleucine Degradation I	1.80	1.58E-02	20.0	NaN	ACAA2,ECHS1,HADHB	3
Regulation of eIF4 and p70S6K Signaling	1.68	2.09E-02	7.4	NaN	ITGA4,PPP2R2C,RPS14,RPS18,RPS19,RPS20,RPS25,RPS27L,RPS29,RPS4Y1,RPS5	11
Superpathway of Geranylgeranyldiphosphate Biosynthesis I (via Mevalonate)	1.65	2.24E-02	17.6	NaN	ACAA2,FDPS,HADHB	3
Eicosanoid Signaling	1.60	2.51E-02	9.7	NaN	ALOX12,ALOX15,CYSLTR2,PLA2G6,PTGER1,PTGIS	6
Valine Degradation I	1.58	2.63E-02	16.7	NaN	BCKDHA,ECHS1,HADHB	3
Fatty Acid beta-oxidation I	1.55	2.82E-02	12.5	– 1	ACAA2,ACADM,ECHS1,HADHB	4
Methionine Degradation I (to Homocysteine)	1.52	3.02E-02	15.8	NaN	EEF1AKMT1,EHMT1,PRMT1	3
Glutamate Biosynthesis II	1.44	3.63E-02	100.0	NaN	GLUD1	1
Glutamate Degradation X	1.44	3.63E-02	100.0	NaN	GLUD1	1
Cysteine Biosynthesis III (mammalia)	1.40	3.98E-02	14.3	NaN	EEF1AKMT1,EHMT1,PRMT1	3
Ketolysis	1.39	4.07E-02	22.2	NaN	ACAA2,HADHB	2
Ephrin B Signaling	1.35	4.47E-02	8.5	NaN	AXIN1,CFL2,CTNNB1,GNB2,GNG10,GNG4	6
TCA Cycle II (Eukaryotic)	1.30	5.01E-02	13.0	NaN	SDHAF4,SDHC,SDHD	3
p53 Signaling	0.89	1.29E-01	6.4	2	BBC3,CTNNB1,GADD45G,HDAC9,KAT2B,ST13	6
Unfolded protein response	0.85	1.40E-01	7.3	2	CALR,CEBPD,HSPA1A/HSPA1B,Hspa1b	4
Wnt/Ca + pathway	0.72	1.90E-01	6.5	2	AXIN1,CREB5,DVL3,NFAT5	4
Netrin Signaling	0.69	2.05E-01	6.3	2	CACNA1A,CACNA1I,CACNB4,NFAT5	4
Calcium Signaling	0.00	1.00E + 00	3.6	2.236	CACNA1A,CACNA1I,CACNB4,CALR,CREB5,HDAC9,NFAT5	7

A cutoff of *p* < 0.05 and FC > 1.2 was applied, and 892 DEGs were selected on RNAseq data for IPA analysis

**Fig. 2:**
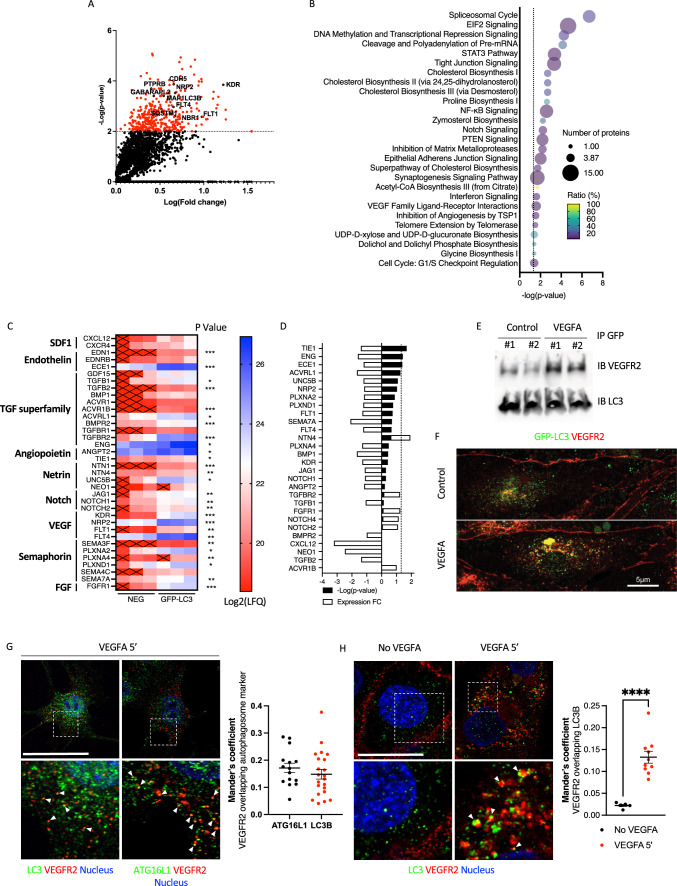
Endothelial autophagosome content. **A** Volcano plot of proteins enriched in autophagosomes of lung endothelial cells isolated from GFP-LC3 mice. **B** Selected canonical pathways identified by Ingenuity Pathway Analysis (IPA) performed on proteins contained into autophagosomes isolated from primary lung endothelial cells from GFP-LC3 mice. **C** Heatmap representation of some membrane and extracellular proteins contained in autophagosomes. *n* = 3 replicates. *p < 0.05, **p < 0.01, ***p < 0.001 GFP-LC3 *vs.* NEG (**D**) Fold-change in protein content of some cell surface and extracellular proteins in primary lung endothelial cells isolated from *Cdh5*.Cre *Atg5*^lox/lox^ mice compared to protein content in primary lung endothelial cells from *Atg5*^lox/lox^. **E** Immunoblot analysis of VEGFR2 and LC3B expression in autophagosomes isolated by GFP immunoprecipitation without or with VEGFA treatment. Lanes #1 and #2 represent two different protein extracts from two different experiments. **F** Immunofluorescence of VEGFR2 and GFP in primary lung endothelial cells isolated from GFP-LC3 mice with or without VEGFA treatment. Scale bar 5 μm. *n* = 6 mice. **G** Immunofluorescence of VEGFR2, LC3B and ATG16L1 in primary lung endothelial cells from WT mice after VEGFA treatment. Cell surface VEGFR2 only was stained to analyze VEGFR2 internalization. *n* = 3 replicates. Scale bar 30 μm. **H** Immunofluorescence of VEGFR2 and LC3B in HUVECs. Cell surface VEGFR2 only was stained to analyze VEGFR2 internalization. *n* = 3 replicates. Scale bar 15 μm. **G**,**H** Colocalization was quantified using Mander’s coefficient

Arguing that autophagy deficiency would modulate proteostasis, we analyzed the proteome of primary lung endothelial cells from control and *Cdh5*.cre-*Atg5*^lox/lox^ mice. We selected 198 DEGs (FC > 1.2, *p* < 0.05, Supp Table S3) and performed IPA and GO pathway analyses. Oxidative phosphorylation and mitochondrial dysfunction were among the most dysregulated canonical pathways in *Atg5* KO endothelial cells. Canonical pathways regulating cell metabolism, autophagy, and prostanoid signaling were also identified (Fig. 1B, Table 2, Supp Fig. 3). ATG5 deficiency also altered carbohydrate and lipid metabolism functions, cell-to-cell signaling and interaction/organization of organelles (Supp Table S4).

**Table 2:** Ingenuity Pathway Analysis (IPA) on proteomic data identified canonical pathways dysregulated in primary endothelial cells from *Cdh5.cre Atg5*^lox/lox^ mice

Ingenuity Canonical Pathways	– log (*p* value)	*p* value	Ratio (%)	z-score	Genes	Number of genes
Prostanoid Biosynthesis	3.91	0.0001230	30	NaN	HPGDS,PTGS1,TBXAS1	3
Oxidative Phosphorylation	2.42	0.0038019	5	– 2.236	ATP5MG,ATP5PO,NDUFB10,NDUFB2,UQCRFS1	5
Mitochondrial Dysfunction	2.20	0.0063096	4	NaN	ATP5MG,ATP5PO,GPX4,NDUFB10,NDUFB2,UQCRFS1	6
Alanine Biosynthesis III	1.99	0.0102329	100	NaN	NFS1	1
Chondroitin Sulfate Degradation (Metazoa)	1.94	0.0114815	13	NaN	HEXB,HYAL2	2
Dermatan Sulfate Degradation (Metazoa)	1.89	0.0128825	12	NaN	HEXB,HYAL2	2
Phagosome Maturation	1.82	0.0151356	4	NaN	CTSB,CTSZ,HLA-DRB5,PRDX2,VPS18	5
Necroptosis Signaling Pathway	1.80	0.0158489	4	– 1.342	CAPNS1,CHUK,PPIF,RBCK1,TSPO	5
Glutathione Redox Reactions I	1.75	0.0177828	10	NaN	GPX4,MGST3	2
L-cysteine Degradation III	1.69	0.0204174	50	NaN	MPST	1
Sirtuin Signaling Pathway	1.67	0.0213796	3	1.000	G6PD,NDUFB10,NDUFB2,PPIF,SOD1,TSPO,UQCRFS1	7
NAD Salvage Pathway II	1.67	0.0213796	9	NaN	ACP1,NT5E	2
Autophagy	1.66	0.0218776	5	NaN	CTSB,CTSZ,VPS18	3
Regulation of eIF4 and p70S6K Signaling	1.62	0.0239883	3	NaN	ITGA3,ITGA9,RPS10,RPS17,RPS29	5
Eicosanoid Signaling	1.58	0.0263027	5	NaN	HPGDS,PTGS1,TBXAS1	3
Glutathione-mediated Detoxification	1.57	0.0269153	8	NaN	HPGDS,MGST3	2
GP6 Signaling Pathway	1.42	0.0380189	3	NaN	COL6A1,COL6A2,COL6A3,FYB1	4
Regulation of Cellular Mechanics by Calpain Protease	1.42	0.0380189	4	NaN	CAPNS1,ITGA3,ITGA9	3
Ferroptosis Signaling Pathway	1.41	0.0389045	3	2.000	CTSB,GPX4,NFS1,TFRC	4
Pentose Phosphate Pathway (Oxidative Branch)	1.39	0.0407380	25	NaN	G6PD	1
Spermine and Spermidine Degradation I	1.39	0.0407380	25	NaN	PAOX	1
Methylmalonyl Pathway	1.39	0.0407380	25	NaN	PCCA	1
2-oxobutanoate Degradation I	1.30	0.0501187	20	NaN	PCCA	1

A cutoff of *p* < 0.05 and FC > 1.2 was applied, and 198 proteins were selected for IPA analysis

We next confirmed mitochondrial dysfunction in ATG5-deficient murine endothelial cells by using shRNA-mediated ATG5 knockdown (KD) in HUVECs. Significant diminution in ATG5 expression and impaired autophagy were confirmed by immunoblot of ATG5 and LC3B and P62 immunofluorescence (Fig. 1C–D). Bafilomycin A1 treatment confirmed autophagy flux reduction in ATG5 KD cells, as shown by decreased P62 accumulation in ATG5 KD cells compared to control cells (Supp Fig. 3).

In ATG5 KD HUVECs, the pool of active mitochondria was reduced, as assessed by a mitotracker red CMXRos staining. In ATG5 KD cells, we observed a rise in the proportion of mitochondria that colocalize within P62. P62 is involved in mitochondrial recycling (Fig. 1. D–E); this suggests that ATG5 deficiency leads to the accumulation of non-recycled P62 + mitochondria. We also found increased expression of the mitophagy initiation proteins BNIP3L (but not BNIP3 and PINK1) in ATG5 KD HUVECs (Supp Fig. 3), suggesting that ATG5 KD cells initiate mitophagy, which is incomplete due to ATG5 deficiency. Western blot analysis also demonstrated a decrease of the mitochondrial respiratory chains proteins SDHB, COX1, ATP5A and UQCRC2, but not SDHA and COX4 (Fig. 1 F). The reduction of active mitochondria in ATG5 KD HUVECs was further confirmed by flow cytometry analysis (Fig. 1 G-H). Thus, these data indicate that ATG5 KD HUVECs had defective oxidative phosphorylation and mitochondrial dysfunction due to a decreased pool of active mitochondria in the absence of a proper recycling process.

Next, we detected metabolites of the tricarboxylic acid (TCA) cycle in ATG5 KD HUVECs. ATG5 KD HUVECs showed an altered abundance of some metabolites: decreased levels of acetoacetate, αketoglutarate, fumarate, and malate and increased levels of hexose, glutamine, and pyruvate (Fig. 1 I–J). Taken together, our results demonstrate that ATG5 deficiency in endothelial cells leads to a global change in endothelial cell metabolism and mitochondrial dysfunction.

### Endothelial autophagosomal content highlighted that proteins of the VEGF pathway are enriched in autophagosomes

The GFP-positive membrane-bound vesicles of bafilomycin A1-treated primary lung endothelial cells from GFP-LC3 mice were isolated using GFP immunoprecipitation to analyze autophagosome protein content [25]. Bafilomycin A1 was used to increase the number of autophagosomes for proteomic analysis. As expected, some autophagosomal proteins (MAP1LC3B, GABARPL2, NBR1 and SQSTM1) and proteins related to mitophagy (TBK1, TBC1D17 and TAX1BP1) were among the most enriched proteins in autophagosomes (Fig. 2A, Supp Table S5). We selected 391 enriched proteins in autophagosomes for pathway analysis (FC > 2, *p* < 0.01, Supp Table S5). Consistent with autophagy interplay with RNA transcription and translation, the top enriched canonical pathways were the spliceosomal cycle, EIF2 signaling, transcriptional repression signaling, and cleavage and polyadenylation of pre-mRNA (Fig. 2B, Table 3). Interestingly, autophagosomes contained proteins necessary for the regulation of endothelial function and signaling pathways such as STAT3-, Notch-, PTEN-, NFκB-, VEGF-, TSP1- or tight junction- and epithelial adherens junction-signaling. Some proteins related to mitochondria (Gene ontology GO:0005739) were also found in autophagosomes (Supp Table S5). GO analysis confirmed the enrichment of pathways related to mRNA processing, but also to angiogenesis, blood vessel morphogenesis, cell adhesion, sprouting angiogenesis, or extracellular matrix organization (Supp Fig. 4). Of note, proteins of the Weibel–Palade bodies were detected in autophagosomes (Supp Fig. 4), consistent with previous findings showing that autophagy is involved in Weibel–Palade maturation and is related to the blood coagulation defect in endothelial-selective ATG7-deficient mice [26], that we also observed in endothelial-selective ATG5-deficient mice (Supp Fig. 4).

**Table 3:** Ingenuity Pathway Analysis (IPA) on autophagosomic data identified canonical pathways enriched in autophagosomes

Ingenuity Canonical Pathways	– log (*p* value)	*p* value	-log (B-H *p* value)	B-H *p* value	Ratio (%)	z-score	Molecules
Spliceosomal Cycle	6.69	2.04E-07	4.47	3.41E-05	20.5	3	CDC5L,DHX15,EIF4A3,RBM8A,SF3A3,SF3B1,SF3B2,U2AF2,XAB2
EIF2 Signaling	4.66	2.19E-05	2.61	2.43E-03	7.3	3.317	EIF4A3,PTBP1,RPL13A,RPL17,RPL18A,RPL27,RPL27A,RPL35,RPL35A,RPL5,RPL7L1,RPL9,RPS11,RPS27L,SHC1
DNA Methylation and Transcriptional Repression Signaling	4.25	5.62E-05	2.33	4.71E-03	17.6	#NOMBRE!	CHD4,HDAC2,MBD3,MTA1,MTA2,RBBP7
Cleavage and Polyadenylation of Pre-mRNA	4.14	7.24E-05	2.31	4.89E-03	33.3	#NOMBRE!	CPSF3,CPSF6,CSTF2,PABPN1
STAT3 Pathway	3.35	4.47E-04	1.63	2.34E-02	7.4	3.162	BMPR2,FGFR1,FLT1,FLT4,IFNAR1,IL2RG,IL4R,KDR,TGFB2,TGFBR2
Tight Junction Signaling	3.31	4.90E-04	1.63	2.34E-02	6.8	#NOMBRE!	BET1L,CNKSR3,CPSF3,CPSF6,CSTF2,GOSR1,GPAA1,SAFB,TGFB2,TGFBR2,YBX3
Cholesterol Biosynthesis I	2.69	2.04E-03	< 1.3	> 0.05	23.1	#NOMBRE!	DHCR24,HSD17B7,MSMO1
Cholesterol Biosynthesis II (via 24,25-dihydrolanosterol)	2.69	2.04E-03	< 1.3	> 0.05	23.1	#NOMBRE!	DHCR24,HSD17B7,MSMO1
Cholesterol Biosynthesis III (via Desmosterol)	2.69	2.04E-03	< 1.3	> 0.05	23.1	#NOMBRE!	DHCR24,HSD17B7,MSMO1
Proline Biosynthesis I	2.62	2.40E-03	< 1.3	> 0.05	50.0	#NOMBRE!	ALDH18A1,PYCR2
NF-κB Signaling	2.58	2.63E-03	< 1.3	> 0.05	5.9	1.897	BMPR2,FCER1G,FGFR1,FLT1,FLT4,HDAC2,KDR,TBK1,TGFBR2,TNIP1
Zymosterol Biosynthesis	2.23	5.89E-03	< 1.3	> 0.05	33.3	#NOMBRE!	HSD17B7,MSMO1
Notch Signaling	2.23	5.89E-03	< 1.3	> 0.05	11.1	#NOMBRE!	DTX2,JAG1,NOTCH1,NOTCH2
PTEN Signaling	2.19	6.46E-03	< 1.3	> 0.05	5.9	-2.828	BMPR2,CNKSR3,FGFR1,FLT1,FLT4,KDR,SHC1,TGFBR2
Inhibition of Matrix Metalloproteases	2.19	6.46E-03	< 1.3	> 0.05	10.8	0	ADAM12,MMP14,THBS2,TIMP3
Epithelial Adherens Junction Signaling	2.01	9.77E-03	< 1.3	> 0.05	5.5	#NOMBRE!	ACVR1B,BMPR2,FGFR1,KEAP1,NOTCH1,NOTCH2,TGFB2,TGFBR2
Superpathway of Cholesterol Biosynthesis	1.73	1.86E-02	< 1.3	> 0.05	10.7	#NOMBRE!	DHCR24,HSD17B7,MSMO1
Synaptogenesis Signaling Pathway	1.69	2.04E-02	< 1.3	> 0.05	4.0	3.464	BET1L,CDH5,CREB1,EPHA2,EPHB4,GOSR1,GPAA1,LRP8,RELN,SHC1,THBS1,THBS2
Acetyl-CoA Biosynthesis III (from Citrate)	1.69	2.04E-02	< 1.3	> 0.05	100.0	#NOMBRE!	ACLY
Interferon Signaling	1.61	2.45E-02	< 1.3	> 0.05	9.7	#NOMBRE!	IFITM2,IFITM3,IFNAR1
VEGF Family Ligand-Receptor Interactions	1.59	2.57E-02	< 1.3	> 0.05	6.1	2	FLT1,FLT4,KDR,NRP2,SHC1
Inhibition of Angiogenesis by TSP1	1.54	2.88E-02	< 1.3	> 0.05	9.1	#NOMBRE!	KDR,TGFBR2,THBS1
Telomere Extension by Telomerase	1.50	3.16E-02	< 1.3	> 0.05	14.3	#NOMBRE!	HNRNPA2B1,RAD50
UDP-D-xylose and UDP-D-glucuronate Biosynthesis	1.40	3.98E-02	< 1.3	> 0.05	50.0	#NOMBRE!	UXS1
Dolichol and Dolichyl Phosphate Biosynthesis	1.40	3.98E-02	< 1.3	> 0.05	50.0	#NOMBRE!	DHDDS
Glycine Biosynthesis I	1.40	3.98E-02	< 1.3	> 0.05	50.0	#NOMBRE!	SHMT2
Cell Cycle: G1/S Checkpoint Regulation	1.39	4.07E-02	< 1.3	> 0.05	6.3	#NOMBRE!	HDAC2,PAK1IP1,RPL5,TGFB2

A cutoff of *p* < 0.01 and FC > 2 was applied, and 391 proteins were selected for IPA

We detected 63 proteins expressed at the plasma membrane and 36 secreted proteins, including members of protein families important to maintain endothelial function such as SDF1-, Endothelin-, TGF-, angiopoietin-, Netrin-, VEGF-, Notch-, Semaphorin-, FGF-pathways (Fig. 2C**, **Table 3). None of these proteins were dysregulated at the mRNA level in the RNAseq analysis of primary lung endothelial cells isolated from *Cdh5*.Cre *Atg5*^lox/lox^ mice as compared to cells from littermate controls. The comparative proteome of these endothelial cells evidenced a significantly decreased abundance of TIE1, ENG and ECE1 (Fig. 2D). Interestingly, the VEGF receptor type 2 VEGFR2/KDR and other members of the VEGF signaling pathway at plasma membrane (NRP2, FLT1, FLT4, CDH5 and PTPRB) were among the most enriched proteins in autophagosomes (Fig. 2A**, **Table 3). We confirmed by immunoblot the presence of VEGFR2 in autophagosomes of primary lung endothelial cells isolated from GFP-LC3 mice and showed that VEGFA treatment further increased the detection of VEGFR2 in autophagosomes (Fig. 2E). Immunofluorescence confirmed that VEGFA treatment promoted partial relocalization of VEGFR2 to GFP-LC3 + dots (Fig. 2F). Because bafilomycin A1 treatment could alter lysosomal trafficking, we also immunoprecipitated GFP-LC3 vesicles in the absence of Bafilomycin A1 and still demonstrate VEGFR2 enrichment in autophagosomes (Supp Fig. 4). To exclude a putative GFP-mediated artifact in cells isolated from GFP-LC3 mice, we next confirmed partial VEGFR2 colocalization with the autophagosome markers LC3B and ATG16L1 in VEGF-treated primary lung endothelial cells from WT mice (Fig. 2G) and in HUVECs (Fig. 2H).

### ATG5 deficiency impacts VEGF signaling

**Fig. 3:**
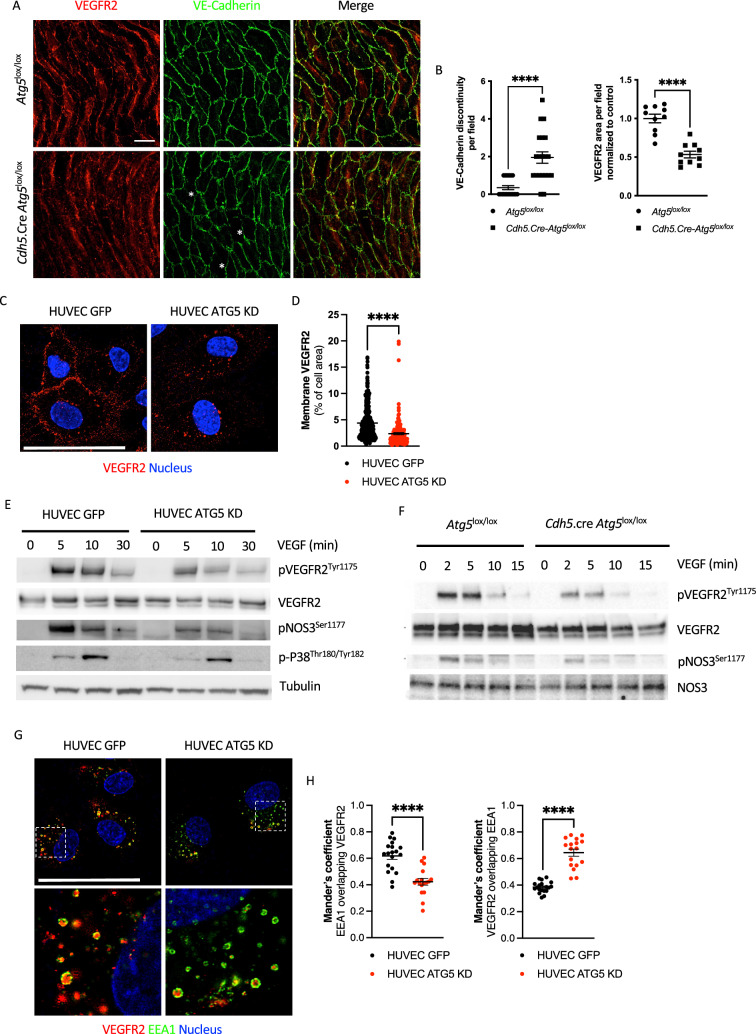
ATG5 deficiency induces defective VEGFR2 signaling. **A** Immunofluorescence of VEGFR2 (red) and VE-cadherin (green) expressions on flat mount aortas from *Atg5*^lox/lox^ and *Cdh5*.Cre *Atg5*^lox/lox^ mice. *n* = 5 mice per genotype. Scale bar 20μ m. **B** Quantification of VE-cadherin discontinuity and VEGFR2 area per field in flat-mount aortas from *Atg5*^lox/lox^ and *Cdh5*.Cre.*Atg5*^lox/lox^ mice. 4 fields per mouse were analyzed for VE-cadherin quantification, and 2 fields per mouse for VEGFR2 quantification. **C** Immunofluorescence of cell surface VEGFR2 (red) expression in control and ATG5 KD HUVECs. Nuclei were stained with Hoechst. *n* = 3 replicates. Scale bar 25 μm (**D**) Quantification of the cell surface area of VEGFR2 per cell area in control and ATG5 KD HUVECs. **E** Western blot analysis of phospho-VEGFR2, VEGFR2, phospho-eNOS and phospho-P38 in control and ATG5 KD HUVECs after VEGFA treatment. Tubulin was used as a loading control. *n* = 4 replicates. **F** Western blot analysis of phospho-VEGFR2, VEGFR2, phospho-NOS3 and NOS3 expression in primary lung endothelial cells from *Atg5*^lox/lox^ and *Cdh5.cre Atg5*^lox/lox^ mice after VEGFA treatment. *n* = 4 replicates. **G** Immunofluorescence of VEGFR2 (red) and EEA1 (green) expression in control and ATG5 KD HUVECs after 20 min of VEGFA treatment. Cell surface VEGFR2 was stained to visualize internalization upon VEGFA treatment. Nuclei were stained with Hoechst. *n* = 3 replicates. Scale bar 25 μm. **H** Quantification of VEGFR2 staining overlapping EEA1 staining and EEA1 staining overlapping VEGFR2 staining in control and ATG5 KD HUVECs after 20 min of VEGFA treatment

We then speculate that autophagy could be involved in VEGF signaling. As no significant change in total endothelial VEGFR2 mRNA or protein content was detected in primary lung endothelial cells from *Cdh5.cre-Atg5*^*lox/lox*^ mice (Fig. 2D, Supp Table S1, Supp Table S3, Supp Fig. 3) nor in HUVECs ATG5 KD (Supp Fig. 3), we hypothesized that VEGFR2 subcellular localization or signaling could be impaired by ATG5 deficiency. We found decreased cell surface VEGFR2 expression in endothelial cells on flat mount aortas from *Cdh5.cre-Atg5*^*lox/lox*^ mice, which was associated with discrete and local VE-Cadherin junction disruptions (Fig. 3A, B). Such decreased expression of cell surface VEGFR2 was confirmed in vitro in ATG5 KD HUVECs (Fig. 3C, D and Supp Fig. 4). Furthermore, VEGF-mediated VEGFR2 activation was impaired in ATG5 KD HUVECs: VEGFR2 phosphorylation was reduced, as well as subsequent eNOS and P38 activation (Fig. 3E). Of note, ATG5 deficiency altered not only VEGF/VEGFR2 signaling but also HGF/MET signaling in HUVECs (Supp Fig. 5), suggesting that autophagy could be essential for proper signaling of several tyrosine kinase receptors. Similar impaired VEGFR2-eNOS signaling was found in primary lung endothelial cells from *Cdh5.cre-Atg5*^*lox/lox*^ mice (Fig. 3F). VEGF-independent shear-stress-induced VEGFR2 phosphorylation was also partially impaired in ATG5 KD HUVECs (Supp Fig. 5). Finally, VEGF-mediated VEGFR2 internalization and recycling were analyzed by quantifying VEGFR2 colocalization to EEA1 + endosomes. After 20 min of VEGFA treatment, most of the internalized VEGFR2 was no longer localized in EEA1 + endosomes in control HUVECs, but the majority of remaining EEA1 + endosomes still contained some VEGFR2. Conversely, in ATG5 KD HUVECs, most internalized VEGFR2 still localized to EEA1 + endosomes, but most EEA1 + endosomes did not contain VEGFR2 (Fig. 3F, G). This result suggests impaired VEGFR2 routing and recycling in ATG5-deficient endothelial cells.

To exclude ATG5-selective autophagy-independent effects, we also generated ATG7 KD HUVECs. Despite a > 70% decrease in ATG7 protein expression in the sh-ATG7 HUVECs, and decreased expression of LC3-B autophagosomal form, we could not measure a significant P62 accumulation at baseline, whereas bafilomycin A1 still induced P62 accumulation in ATG7 KD HUVECs (Supp Fig. 6). These suggested that ATG7 KD was less efficient than ATG5 KD to block autophagy in HUVECs. Conversely to ATG5 KD cells, ATG7 KD HUVECs did not present mitochondrial abnormalities: mitochondrial footprint was similar to Control HUVECs, and mitochondrial ROS production. On the other hand, we still observed low plasma membrane VEGFR2 expression in ATG7 KD cells and mislocalization to EEA1 + endosomes (Supp Fig. 6). Because ATG7 has been shown to be essential to regulate EndoMT, promoting decreased expression of some endothelial markers such as CD31, CDH5 or TIE1 [27], we could not exclude that plasma membrane VEGFR2 downregulation in ATG7 KD HUVECs could be linked to the phenotypic switch towards a mesenchymal phenotype. In accordance, ATG7 KD HUVECs have a more elongated “fibroblast-like” cell shape than Control or ATG5 KD HUVECs (Supp Fig. 6). Of note, Singh et al. also observed a decrease in LC3-II without P62 significant change [27]. Altogether, these results suggested that ATG5 and ATG7 may not exert fully redundant functions in endothelial cells.

### Angiogenesis defects in vascular explants of endothelial-selective ATG5-deficient mice

**Fig. 4:**
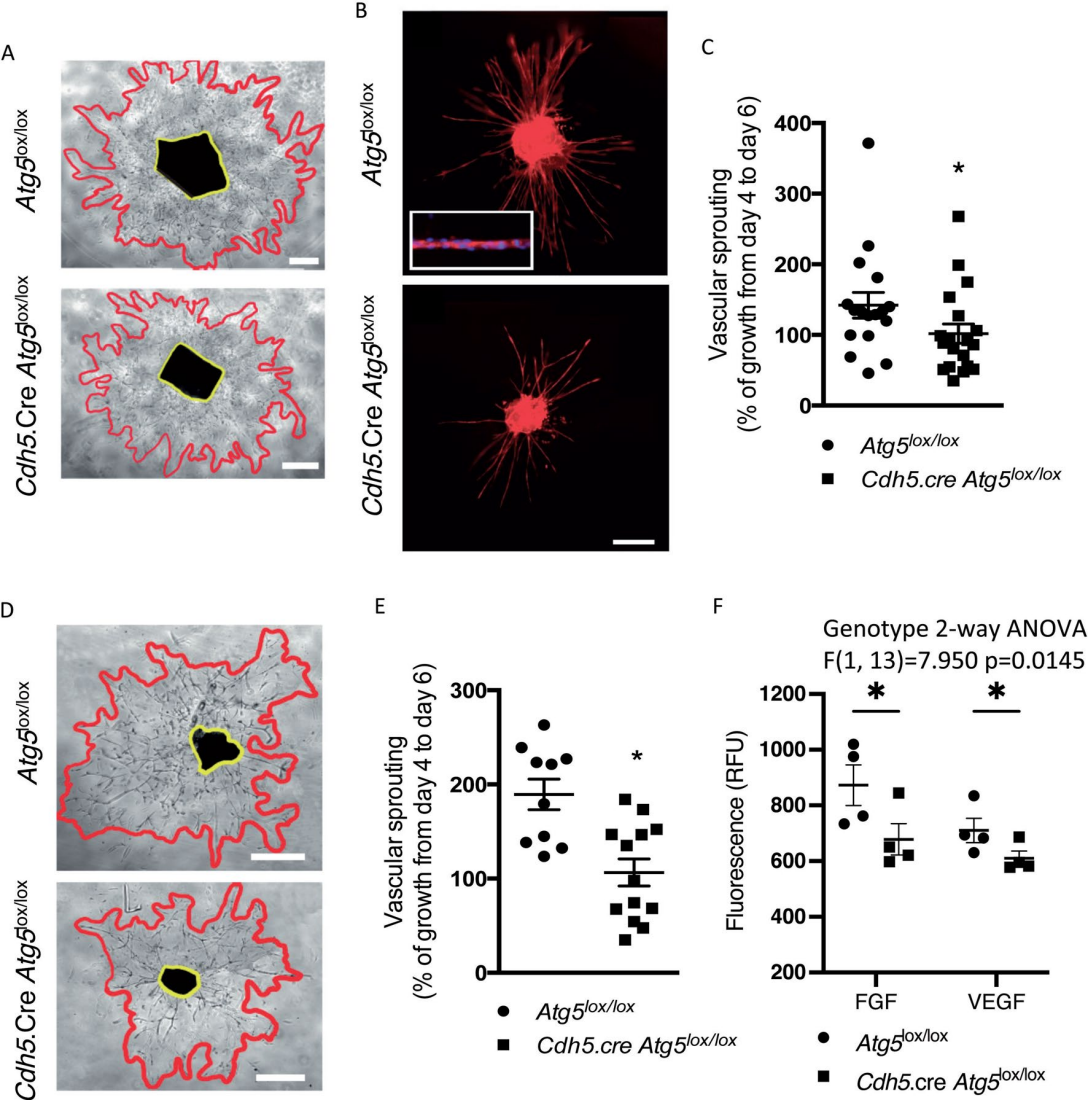
Endothelial autophagy deficiency impairs angiogenesis. **A** Representative images of aortic explants in 4 to 6-week-old *Atg5*^lox/lox^ and *Cdh5*.Cre.*Atg5*^lox/lox^ mice. Scale bar 400 mμ. **B** Representative images of TRITC-coupled isolectin showing endothelial cells forming tubes. Scale bar 200 mμ. Higher magnification is shown in the inset. **C** Aortic ring assay quantification in *Atg5*^lox/lox^ and *Cdh5*.Cre.*Atg5*^lox/lox^ mice showing decreased vascular sprouting in *Cdh5*.Cre.*Atg5*^lox/lox^ aortic explants. **D** Representative images of choroid explants in 4 to 6-week-old *Atg5*^lox/lox^ and *Cdh5*.Cre.*Atg5*^lox/lox^ mice. Scale bar 400 mμ. **E** Choroid sprouting angiogenesis assay quantification in *Atg5*^lox/lox^ and *Cdh5*.Cre.*Atg5*^lox/lox^ mice showing decreased vascular sprouting in *Cdh5*.Cre.*Atg5*^lox/lox^ choroidal explants. Values are individual values and means ± SEM of 10–14 mice. *p < 0.05. **F** In vivo DIVAA angiogenesis assay. Angioreactors containing FGF2 or VEGFA were implanted subcutaneously for 15 days. After removal, endothelial cells into angioreactors were stained with a fluorescent isolectin staining and fluorescence was quantified. Quantification of angiogenesis in angioreactors after implantation in *Atg5*^lox/lox^ and *Cdh5*.Cre.*Atg5*^lox/lox^ mice showed decreased FGF- and VEGF-induced angiogenesis in *Cdh5*.Cre.*Atg5*^lox/lox^ mice. *n* = 4 angioreactors per condition. Values are individual values and means ± SEM. 2 way-ANOVA: genotype effect: *p < 0.05, post-hoc Fisher LSD test *p < 0.05

We explored ex vivo whether endothelial ATG5 deficiency could influence angiogenesis. While extensive vascular outgrowth was observed from aortic rings isolated from *Atg5*^lox/lox^ mice, the extension of endothelial sprouts was significantly reduced in aortic rings from *Cdh5.cre-Atg5*^*lox/lox*^ mice (Fig. 4A–C). Similar observations were found in a choroid sprouting model of microvascular angiogenesis [28] (Fig. 4D–E). An in vivo angiogenesis assay using subcutaneous angioreactor implants showed reduced FGF2- and VEGFA-mediated angiogenesis in *Cdh5.cre-Atg5*^*lox/lox*^ mice, confirming impaired neo-angiogenesis in endothelial-selective ATG5-deficient mice (Fig. 4F). Together, our results demonstrate that endothelial autophagy deficiency impairs neo-angiogenesis.

### Defective endothelial autophagy results in selective loss of flow-induced vasodilation in mesenteric arteries and kidneys

**Fig. 5:**
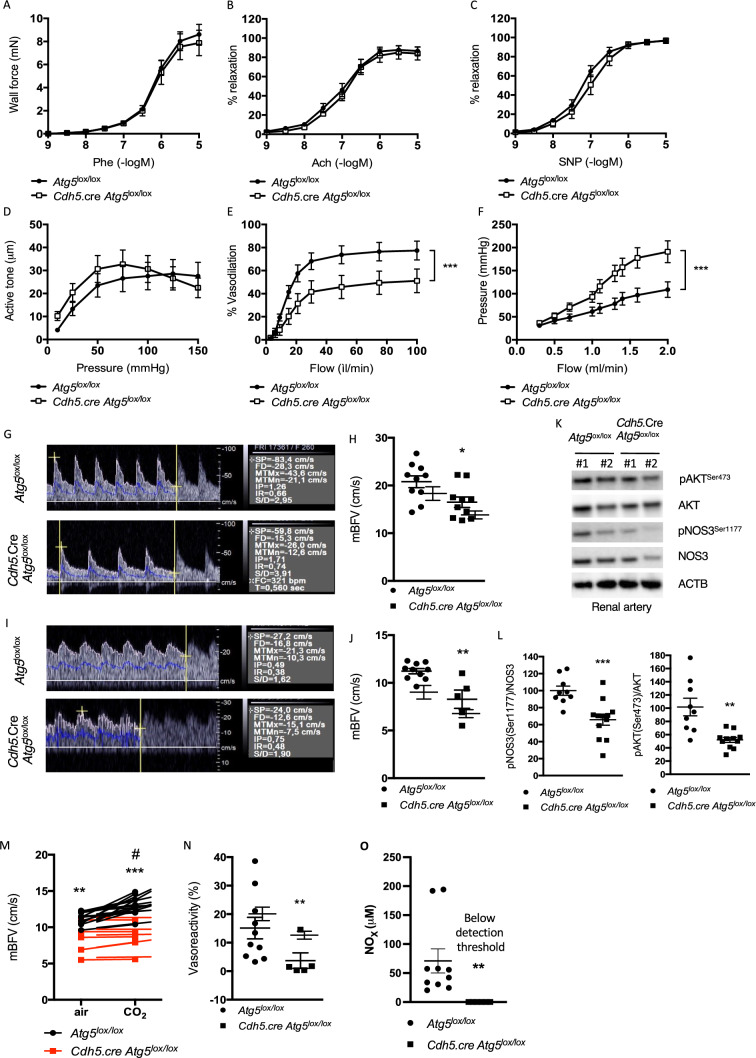
Mice with selective-endothelial autophagy deficiency present decreased peripheric blood flow velocity and endothelial dysfunction. **A-E** Changes in mesenteric artery contractility in mice with endothelial autophagy deficiency. **A** Wall force was measured in response to phenylephrine (Phe) in isolated mesenteric arteries from *Atg5*^lox/lox^ and *Cdh5*.cre-*Atg5*^lox/lox^ mice. **B**, **C** Mesenteric arteries were precontracted with Phe. Acetylcholine (Ach) (**B**) or NO donor sodium nitroprusside (SNP) (**C**) was cumulatively added after the contraction had reached a steady-state level. Data are shown as percentage of relaxation of the steady-state preconstriction. **D** Myogenic tone. **E** Flow-mediated dilation (FMD). Autophagy deficiency strongly impairs FMD in isolated mesenteric arteries. **F** Intrarenal pressure flow-relationship measured in isolated and perfused kidneys from *Atg5*^lox/lox^ and *Cdh5*.cre-*Atg5*^lox/lox^ mice. **A–F** Values are means ± SEM of *n* = 9 *Atg5*^lox/lox^ mice and *n* = 7 *Cdh5*.cre-*Atg5*^lox/lox^ mice. ***p < 0.001 (**G–J**) Echo-Doppler measurements of mean blood flow velocity (mBFV). **G**, **I** Representative BFV waveforms in the renal artery (**G**) and the basilar trunk artery (**I**) in 12 weeks-old *Atg5*^lox/lox^ and *Cdh5*.cre.*Atg5*^lox/lox^ mice at steady state. **H**, **J** Echo-Doppler measurements of mean blood flow velocity (mBFV) in the renal artery (**H**) and the basilar trunk artery (BT) (**J**). Endothelial autophagy deficiency is associated with a significant decrease in renal and cerebral mBFV velocity. *p < 0.05; ** *p* < 0.01. Values are individual values and means ± SEM. *n* = 10 mice per group except for (**J**) where *n* = 10 *Atg5*^lox/lox^ mice and *n* = 5 *Cdh5*.cre.*Atg5*^lox/lox^ mice. **K**, **L** Representative immunoblot (**K**) and quantifications (**L**) of AKT, phospho-AKT, eNOS (NOS3) and phospho-eNOS expressions in freshly isolated renal arteries from *Atg5*^lox/lox^ and *Cdh5*.cre.*Atg5*^lox/lox^ mice. β Actin (ACTB) was used as a loading control. In K, lanes #1 and #2 represent 2 different protein extracts from 2 different mice. Values are individual values and mean ± SEM of *n* = 9 *Atg5*^lox/lox^ mice and *n* = 11 *Cdh5*.cre-*Atg5*^lox/lox^ mice. ***p < 0.001; **p < 0.01. (M,N) Hypercapnia-induced vasodilatation is impaired in the BT from *Cdh5*.cre.*Atg5*^lox/lox^ mice. **M** mBFV in BT from *Atg5*^lox/lox^ and *Cdh5*.cre.*Atg5*^lox/lox^ mice after air or CO_2_ inhalation. CO2 inhalation induces a rise in mBFV in *Atg5*^lox/lox^ mice but not in *Cdh5*.cre-*Atg5*^lox/lox^ mice. (**N**) Hypercapnia-induced relative vasoreactivity in BT from *Atg5*^lox/lox^ and *Cdh5*.cre-*Atg5*^lox/lox^ mice. # CO_2_ vs. air in *Atg5*^lox/lox^ mice, * *Atg5*^lox/lox^ vs. *Cdh5*.cre-*Atg5*^lox/lox^. #*p* < 0.05; **p < 0.01 ***p < 0.001. Values are individual values and means ± SEM. *n* = 10 *Atg5*^lox/lox^ mice and *n* = 5 *Cdh5*.cre.*Atg5*^lox/lox^ mice. **O** NOx measurement in the plasma of *Atg5*^lox/lox^ and *Cdh5*.cre.*Atg5*^lox/lox^ mice. NOx level was always below detection threshold in the plasma of *Cdh5*.cre.*Atg5*^lox/lox^ mice. Values are individual values and means ± SEM. *n* = 10 *Atg5*^lox/lox^ mice and *n* = 6 *Cdh5*.cre.*Atg5*^lox/lox^ mice. **p < 0.01

Next, we explored the effects of defective endothelial autophagy on endothelium-dependent and endothelium-independent vasodilation. Ex vivo vascular responsiveness to phenylephrine and concentration–response curves to acetylcholine and NO donor sodium nitroprusside (SNP) were performed in isolated mesenteric resistance arteries from *Cdh5*.cre-*Atg5*^lox/lox^ mice and WT littermates. Endothelial deletion of *Atg5* did not alter the mesenteric arterial compliance. Phenylephrine-induced contraction, endothelium-dependent relaxation in response to acetylcholine and endothelium-independent relaxation in response to SNP (Fig. 5A–C) did not differ between *Cdh5*.cre-*Atg5*^lox/lox^ and control mice. Myogenic tone also remained intact (Fig. 5D). Thus, endothelial autophagy did not impact vessel responses to vasoconstrictive and vasodilatory molecules.

As endothelial flow sensing strongly depends on VEGFR2-NO signaling, we explored the role of endothelial autophagy on functional endothelial flow-sensing by measuring flow-mediated vasodilation (FMD) of mouse mesenteric arteries. In pre-constricted vessels isolated from *Cdh5*.cre-*Atg5*^lox/lox^ mice, FMD was strongly impaired compared to WT littermates (Fig. 5E). In line with these results, renal flow-mediated pressure was also increased in isolated perfused kidneys from *Cdh5*.cre-*Atg5*^lox/lox^ mice when compared to WT littermates (Fig. 5F). Taken together, these results indicate that endothelial ATG5 is required for FMD in resistive vascular beds.

### Endothelial autophagy deficiency increases renal and cerebral vascular resistances

Telemetry, plethysmography, and ultrasonography measurements did not reveal differences in systolic blood pressure, heart rate, or heart structure between *Cdh5*.cre-*Atg5*^lox/lox^ and WT littermates (Supp Fig. 7 and Supp Tables 6–7). Further, the vascular network in adult retinas was similar between *Cdh5*.cre-*Atg5*^lox/lox^ mice and WT (Supp Fig. 8), suggesting no significant developmental effects of endothelial-ATG5 deficiency.

As impaired FMD of resistance arteries may lead to an increase in peripheral vascular resistance, we investigated the role of endothelial autophagy on vascular resistance in kidneys and the brain. Whereas the cardiac output (CO) did not differ between *Cdh5.cre-Atg5*^*lox/lox*^ mice and littermate controls (Supp Table 7), the mean blood flow velocity (mBFV) was significantly decreased in the kidneys and basilar artery of *Cdh5.cre-Atg5*^*lox/lox*^ mice (Fig. 5G–J). Importantly, micro-computed tomography 3D angio-scanning of the kidneys revealed no difference in the characteristics of renal vascular trees of the two genotypes (Supp Fig. 9), thus suggesting that the decreased renal mBFV in *Cdh5.cre-Atg5*^*lox/lox*^ mice was the consequence of increased vascular resistance rather than abnormal renal vascularization. Correlating with such a hypothesis, we observed a decreased phosphorylation of AKT at Ser^473^ and NOS3 at Ser^1177^ in isolated renal arteries (Fig. 5K, L) from *Cdh5.cre-Atg5*^*lox/lox*^ mice. We also assessed vasodilation of basilar arteries in response to hypercapnia as an in vivo test of endothelial function [29, 30]. This was significantly impaired in *Cdh5*.cre-*Atg5*^lox/lox^ mice (Fig. 5M, N). In accordance with impaired NOS3 signaling in arteries from *Cdh5*.cre-*Atg5*^lox/lox^ mice and in HUVECs ATG5 KD, we found decreased NOx levels in the plasma of *Cdh5*.cre-*Atg5*^lox/lox^ mice when compared to plasma from WT mice (Fig. 5O), thus showing that endothelial ATG5 deficiency leads to impaired vascular NO production. These results are consistent with the ex vivo explorations of FMD and demonstrate that endothelial ATG5 is required for endothelial mechanosensing in another important vascular bed.

#### Endothelial ATG5 deficiency exacerbates vascular dysfunction in stressed mice

We next addressed if stress conditions would exacerbate cardiovascular dysfunction triggered by impaired endothelial autophagy.

**Fig. 6:**
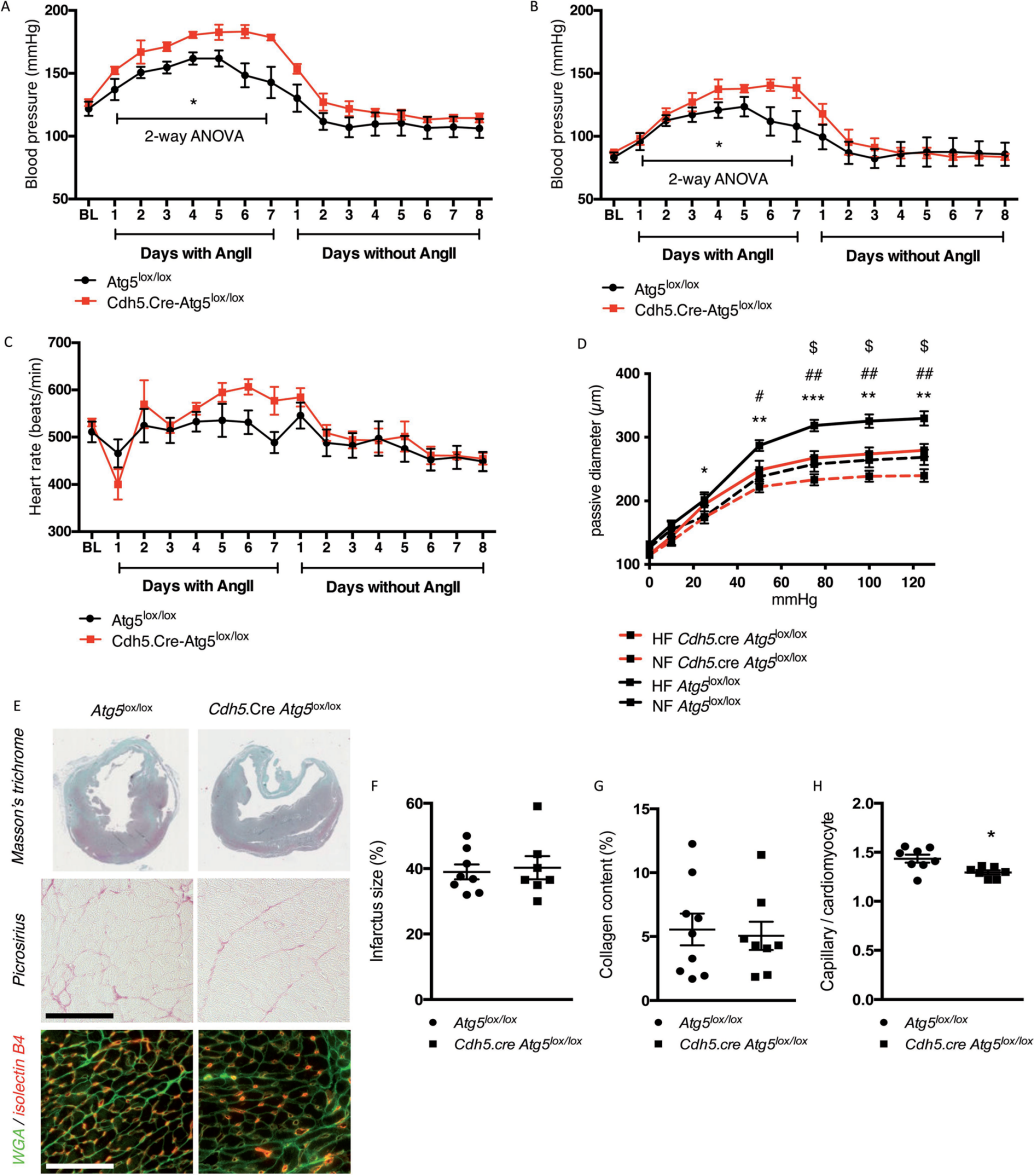
Endothelial autophagy deficiency impairs flow-mediated vascular remodeling. **A–C** *Cdh5*.cre *Atg5*^lox/lox^ mice have an abnormally elevated rise in blood pressure after angiotensin II perfusion. Day measurements of systolic (**A**) and diastolic (**B**) blood pressure and heart rate (**C**) were recorded by radiotelemetry. Values are means ± SEM of *n* = 4 mice per group. **D** Endothelial autophagy deficiency impairs flow-mediated outward remodeling of mesenteric arteries. Arterial diameter was measured 1 week after arterial ligation in high-flow (HF) and normal-flow (NF) arteries isolated from *Atg5*^lox/lox^ control and *Cdh5*.cre-*Atg5*^lox/lox^ mice in response to increasing intraluminal pressure. Values are means ± SEM of *n* = 7–10 mice. 2-way ANOVA: pressure *p* < 0.0001, genotype *p* < 0.001. Fisher’s LSD test: * # $ *p* < 0.05, ** ##*p* < 0.001, ***p < 0.001. * NF vs. HF in *Atg5*^lox/lox^, $ NF vs. HF in *Atg5*^lox/lox^, # HF *Atg5*lox^/lox^ vs. HF *Cdh5*.cre *Atg5*^lox/lox^. E Representative images of Masson’s trichrome (top panel), picrosirius (middle panel) and GSA-TRITC (red)/WGA-FITC (green) (lower panel) staining of hearts from *Cdh5*.Cre *Atg5*^lox/lox^ and *Atg5*^lox/lox^ mice, 10 days after MI. Scale bar 50μ m. **F–H** Quantifications of the infarcts size (**F**), collagen content (**G**) and capillary density (**H**). Values are individual plots and means ± SEM of *n* = 8 mice. *p < 0.05

First, we found that the blood pressure rise in response to angiotensin II was significantly accentuated in endothelium-ATG5 deficient mice relative to WT, while heart rates were similar between the two groups (Fig. 6A–C).

We then tested the flow-mediated outward arterial remodeling. In response to a chronic increase in blood flow, the normal physiological adaptation involves an increase in arterial diameter until shear stress is normalized. Such flow-mediated outward remodeling requires endothelium-mediated dilation. Passive mesenteric arterial diameter in response to increasing intraluminal pressure was measured 1 week after arterial ligation in high-flow (HF) and normal-flow (NF) arteries isolated from *Cdh5.cre-Atg5*^lox/lox^ mice and controls. The passive arterial diameter was significantly higher in HF than in NF arteries in control mice, while in *Cdh5.cre-Atg5*^*lox/lox*^ mice, the passive arterial diameter was similar in HF and NF arteries (Fig. 6D). Thus, diameter enlargement did not occur in HF arteries from endothelial-selective ATG5 deficient mice, showing that ATG5 is required for flow-mediated outward arterial remodeling.

Next, we analyzed the role of endothelial autophagy in femoral artery responses to wire-induced endothelial denudation and smooth muscle injury. Of note, eNOS signaling was impaired in the femoral arteries of *Cdh5.cre-Atg5*^*lox/lox*^ mice at baseline, as shown by decreased phosphorylation of AKT and NOS3 (Supp Fig. 10), indicating a crucial role for ATG5 in the control of NOS3 activity at steady state in physiological conditions. Arterial wire injury caused the development of concentric neointimal lesions in femoral arteries from *Atg5*^lox/lox^ control mice. Endothelial ATG5 deficiency increased the neointima formation following wire injury, resulting in increased lesion volume and maximal cross-sectional area (Supp Fig. 10). These results suggest that endothelial autophagy supports the re-endothelialization process following wire injury.

Finally, functional recovery of the heart after myocardial infarction (MI) involves both reperfusion of collateral arteries upon the local increase in blood flow and angiogenesis. Endothelial selective ATG5 deficiency resulted in a significant decrease in the left ventricular shortening fraction 10 days after coronary artery ligation (Supp Table S8). The left ventricular posterior wall width was also significantly smaller in *Cdh5.cre-Atg5*^*lox/lox*^ mice, while left ventricular septum size and left ventricle size were similar in *Cdh5.cre-Atg5*^*lox/lox*^ mice and *Atg5*^lox/lox^ littermates (Supp Table S8). Neither the infarct size nor the cardiac fibrosis differed between the groups (Fig. 6E–G), but the number of capillaries per cardiomyocyte was smaller in *Cdh5.cre-Atg5*^*lox/lox*^ mice (Fig. 6E, H)**.** Together, these data suggest that while endothelial-selective ATG5 deficiency does not influence normal heart function or tissue injury induced by coronary artery ligation, it impairs functional recovery after MI.

## Discussion

Our results highlight the pivotal role for autophagy in endothelial functions of small mature vessels, impacting fundamental physiological processes, primarily tissue perfusion and blood pressure regulation. Mitochondrial recycling depends on autophagy (mitophagy), and we confirmed that ATG5 deficiency generated mitochondrial dysfunction in endothelial cells, as previously reported [16]. The global impact of ATG5 deficiency on pivotal endothelial homeostatic systems was uncovered by RNAseq and proteomic analysis, and included metabolic changes, activation of p53 (an anti-ROS countermeasure [31]), the UPR pathway (a general mediator of vascular inflammation [32]), and synthesis of prostanoids (regulators of angiogenesis, vascular inflammation and hypertension [33–35]). In ATG5 KD cells, P53 induction may be responsible for (defective) mitophagy initiation as P53 is a known regulator of BNIP3L [36].

We found ex vivo and in vivo evidence showing that autophagy is required for intact endothelial cell mechanotransduction through the VEGFR2-eNOS pathway. Autophagy is triggered by endothelial shear stress [37, 38] and may be required to ensure normal endothelial cell alignment to flow since a mild alteration of endothelial cell alignment was reported in *Cdh5*.cre *Atg5*^lox/lox^ aorta [17]. Our results suggest that autophagy involvement in flow-induced sensing may be even more critical in resistive arteries where fluid shear stress is more pronounced and mechanotransduction physiologically relevant to ensure proper tissue perfusion through the integration of FMD and endothelial maintenance and repair. Loss of endothelial ATG5 selectively affected shear stress-dependent signal transduction as shown by a reduced FMD and flow-mediated remodeling despite normal endothelium-dependent and endothelium-independent relaxation. Concordant with our findings, the treatment of spontaneously hypertensive rats with the autophagy inducer trehalose enhanced arterial vasodilatation ex vivo [39]. Shear-stress-induced NO production was previously shown to depend on autophagy in bovine endothelial cells where ATG3 deficiency led to decreased NO production in response to shear stress [40]. The same group identified that autophagy suppression led to GLUT1 downregulation, glycolysis diminution and limited purinergic-mediated activation of eNOS [41]. Our study complements these findings and suggests that there are at least two independent pathways by which shear stress induces autophagy-mediated NO production.

Here, our in vivo gene targeting strategy unambiguously uncovers the role of endothelial autophagy in FMD regulation ex vivo and in vivo. Importantly, flow-dependent vasodilation is frequently blunted in asymptomatic individuals with coronary artery disease [42] even when the acetylcholine-mediated dilation is normal [43]. Our study suggests that chronic impairment of protective autophagy, as we observed in the microvasculature of diabetic mice [15] and as reported in conditions associated with CVD [4–9, 11] could thus contribute to endothelial dysfunction and diseases.

Resistance arteries control local blood flow, and their capacity to remodel in response to chronic changes in the hemodynamic environment is necessary to maintain their full efficiency. Chronic increases in arterial blood flow occur in physiological situations, such as growth, pregnancy, or exercise. These increases in blood flow induce outward hypertrophic remodeling in resistance arteries and depend upon NO-dependent dilation [44]. Endothelial-specific ATG5 deficiency impaired not only the flow-induced vasodilation *ex-vivo* but also the outward hypertrophic remodeling in vivo, similar to that observed in aged [45], diabetic [46] or hypertensive [47] animals. Thus, endothelial ATG5 deficiency impacted not only basal endothelial function but also the adaptive arterial remodeling to flow.

Endothelial regeneration is an essential process for preventing excessive neointimal formation following endothelial denudation and for recovery after myocardial infarction. Altered shear stress response of damaged or dysfunctional endothelium stimulates neointima formation by migration and proliferation of media-derived mural smooth muscle cells. Here, flow-induced vascular remodeling following wire injury was likely impaired by endothelial *Atg5* deletion, thus favoring the formation of neointimal lesions. *Beclin 1* knockdown, mediated by siRNA systemic delivery, exacerbated neointimal formation after rat carotid injury mediated by endothelial denudation [48]. Because systemic *Beclin 1* knockdown could have impacted other crucial non-endothelial partners, our data strongly advocate for a requirement of endothelial autophagy in re-endothelialization in vivo and in the restoration of myocardial capillary density during MI recovery.

Internalized VEGFR2 in response to VEGF colocalized with autophagosomes in normal cells. ATG5 deficiency led to a decreased expression of cell surface VEGFR2 and impaired phosphorylation cascade in response to VEGF, which might be partly linked to the impaired routing of VEGFR2 to endosomes and recycling in the absence of ATG5. Recent evidence supported the role of the autophagy pathway in recycling internalized tyrosine kinase receptors: autophagy was involved in recycling altered EEA1 + endosomes, and autophagy deficiency decreased EGFR signaling in glial cells [49]. This study and our results suggest that autophagy might be a monitoring mechanism of altered endosomes allowing proper recycling of defective endosomes to ensure proper trafficking of tyrosine kinase receptors. Further studies should clarify whether this mechanism is restricted to specific receptors or is a universal mechanism to monitor endosome integrity.

Finally, autophagosomes contain extracellular and membrane proteins that play essential roles in endothelial homeostasis and mechanotransduction, such as semaphorins, netrins, extracellular matrix components, and tyrosine kinase receptors, suggesting that autophagy may also control endothelial flow sensing and homeostasis through others signaling pathways. We speculate that, beyond playing a role in the recycling of organelles and proteins, the autophagy machinery could be an essential routing platform for extracellular and membrane proteins, thereby regulating endothelial sensing to extracellular signals.

Overall, our findings unravel a fundamental role for ATG5 in endothelial function, linking cell autophagy to mechanosensing and angiogenic signaling.

## Methods

### Animals

Mice with an endothelial-specific disruption of the *Atg5* gene (*Cdh5*.Cre.*Atg5*^lox/lox^) were generated by crossing *Cdh5*.Cre mice [50] with *Atg5*^lox/lox^ mice [51]. Mice were on pure C57BL/6 J genetic background. Littermate *Atg5*^lox/lox^ male mice were used as controls in all studies. GFP-LC3 transgenic mice were previously described [52]. All mice were bred and housed in a specific pathogen-free facility and were given free access to water and standard chow. Experiments were conducted according to the French veterinary guidelines and those formulated by the European Community for experimental animal use (L358-86/609EEC) and were approved by the Institut National de la Santé et de la Recherche Médicale, local University Research Ethics Committee, and French Ministry of Research (MESR 7646 and 4898). Adult mice from 3-to-12 months of age were used in the study. For surgical procedures, mice were anesthetized with isoflurane inhalation (2,5%) or with ketamine 100 mg/kg—xylazine 10 mg/kg intraperitoneal injection. Pre-surgery and post-surgery analgesia were performed by buprenorphine (0,1 mg/kg) subcutaneous injection. Drugs were diluted in sterile phosphate buffer or saline.

#### Angiotensin-II induced hypertension

The hypertensive model was induced by subcutaneous infusion of angiotensin II (AngII) (Sigma-Aldrich) at a dose of 1 μg/kg/min for 7 days via osmotic minipumps (Alzet Corp.) in 12–14-week-old males. Pumps were implanted subcutaneously on the back between the shoulder blades and hips. Mice received salt supplementation (3% NaCl) in food. After 7 days, mice were reoperated to remove the minipump and then euthanized 7 days after.

#### Chronic increase in blood flow in mesenteric arteries

Arterial ligation in mesenteric arteries was performed as previously described [53, 54]. Ligatures were applied to four second-order arterial branches. The artery located between the two ligated arteries was designed as a high-flow (HF) artery. Arteries located at distance from the ligated arteries were used as control (normal flow (NF)).

#### Myocardial infarction

Myocardial infarction (MI) was induced in mice by permanent coronary ligation, as described previously [55]. In brief, mice were anesthetized and then intubated and ventilated with air using a small-animal respirator. The chest wall was shaved, and a left thoracotomy was performed in the fourth left intercostal space. The left ventricle (LV) was visualized, and the left coronary artery was permanently ligated with monofilament nylon 7–0 sutures (Peters surgical, 26S05F) at the site of its emergence from under the left atrium. The chest wall was closed with 7–0 nylon sutures, and the skin was closed with 6–0 nylon sutures. The sham-operated control mice underwent the same intervention, except that the ligature was left untied. Mice were euthanized 14 days after MI.

#### Radiotelemetry blood pressure (BP) recording

Mice were anesthetized with isoflurane, and a BP telemeter (model TA11PA-C10; data sciences international, St Paul, MN) was implanted in the left femoral artery. A single dose of amoxicillin (20 mg/kg ip) and ketoprofen (5 mg/kg ip) was administered after the surgery. Mice were adapted for one week, and BP values of the last 3 days of the adaptation period were averaged to define the basal BP.

#### Non-invasive ultrasound study of cardiac, cerebral and renal hemodynamics

Ultrasound examination was carried out under light anesthesia sufficient to obtain sedation without cardiorespiratory depression (0.5% Isoflurane in ambient air administered through a vaporizer) [model 100-F; Ohio Medical Instruments®, Cincinnati, OH, USA] [56]. Mice were shaved on the chest, left flank, and occiput to allow good contact between the skin and the ultrasound probe. We used an echocardiograph (Acuson S3000; Siemens®, Erlangen, Germany) equipped with a 14-MHz linear transducer (14L5 SP).

##### Measurement of cardiac output (CO) and left ventricular systolic function

A left parasternal long-axis B-mode image of the chest allowed access to the pulmonary artery for diameter measurement. A pulsed Doppler sample was placed on the longitudinal axis of the pulmonary artery, and the flow profile was recorded. Blood flow velocities (BFVs) were then measured with a correction of the angle between the long axis of the artery and the Doppler beam. CO was calculated with the following formula: CO = [(π(Dpa/2)2) x (mean-BFV × 60)], (CO in ml/min; mean-BFV, spatial-averaged-time-averaged mean BFV in cm/s; Dpa, pulmonary artery diameter in cm).

After activation of the M-mode on a long axis view of the left ventricle, wall thickness and internal diameters in systole and diastole were measured for left ventricular systolic function determination.

##### Measurements of kidney size and renal blood flow velocity (BFV)

A frontal view of the abdomen allowed measurements of the width and the height of the kidneys as previously reported [57]. Color-Doppler mode was activated for renal artery localization, pulsed Doppler sample positioning and Doppler spectrum recording. Spatial-averaged-time-averaged mean-BFVs were measured with angle correction.

##### Measurements of cerebral vasoreactivity

A horizontal B-mode image crossing through the basis of the skull and color Doppler showed the basilar trunk and the circle of Willis as previously reported [58, 59]. A pulsed Doppler sample was then placed on the longitudinal axis of the basilar artery, and the pulsed Doppler spectrum was recorded. Mean-BFVs were measured in the basilar trunk (1) under air, (2) following 4–5 min of a mixture of O2(16%), CO2(5%) and N2(79%), which acts as a cerebral vasodilator [30]. Vasoreactivity was estimated as the percentage increase in mean-BFV recorded under a gas mixture compared to mean BFV recorded under ambient air.

All reported data for each animal represent the average of three measurements.

#### Direct in vivo angiogenesis assay (DIVAA) with angioreactors

In vivo angiogenesis assay was performed using DIVAA angioreactors according to the manufacturer’s protocol (R&D systems). Briefly, angioreactors were prepared on ice with no growth factor, FGF2 (1 μg/mL) or VEGFA (1 μg/mL), then polymerized at 37 °C for 1 h. Four angioreactors per mouse were implanted subcutaneously in Cdh5.cre Atg5^lox/lox^ mice and WT littermates and removed after 14 days. After removal, the angioreactor content was resuspended and homogenized in cellsperse buffer for 1 h at 37 °C and then washed in DIVAA wash buffer. The cell pellet was resuspended in DMEM + 10% FCS and incubated at 37 °C for 1 h to allow cell surface epitope recovery. Cells were then incubated with FITC-lectin overnight at 4 °C. After washes, fluorescence was read on a fluorometer (ex 485 nm, em 510 nm). Data are expressed as relative fluorescence units (RFU).

### Flow- and pressure-dependent tone in isolated mesenteric arteries

Mesenteric artery segments were isolated and cannulated at both ends and mounted in a video-monitored perfusion system as previously described [60] using a pressure arteriograph from Living System Instrumentation Inc. (Burlington, VT). The artery segment was bathed in a 5-ml organ bath containing physiological salt solution of the following composition (mM): 135.0, NaCl; 15.0, NaHCO3; 4.6, KCl; 1.5, CaCl2; 1.2, MgSO4; 11.0, glucose; and 10.0, N-2-hydroxy-ethylpiperazine-N-2-ethylsulfonic acid. The pH was 7.4 and was monitored continuously. The pO2 was maintained at a value of 160 mmHg, and the pCO2 was maintained at a value of 37 mmHg. The pressure in both ends of the artery segment was monitored using pressure transducers. Flow in the vessel could be generated through the distal pipette with a peristaltic pump. Pressure in the proximal end of the vessel was controlled by a servo perfusion system. The arterial diameter was measured and recorded continuously using a video monitoring system. Pressure and flow rate could be changed independently. Equilibrium diameter changes were measured in each segment when intraluminal pressure was set between 10 and 150 mmHg. Flow-mediated dilation was studied by increasing the flow rate by steps from 3 to 150 µl/min when intraluminal pressure was 75 mmHg.

The flow was then stopped, and intraluminal pressure was increased by steps (10–150 mmHg) in order to determine active arterial diameter. Arteries were subsequently perfused and superfused with a calcium-free physiological salt solution containing EGTA (2 mM) and sodium nitroprusside (SNP) (10 μM), and the pressure steps (10–150 mmHg) were repeated to determine the passive diameter of the vessel, i.e., in the absence of smooth muscle tone. Diameters measured in normal physiological salt solution were considered as diameters under active tone or active diameters. Pressure and diameter measurements were collected by a Biopac data acquisition system (MP 100; Biopac, La Jolla, CA), recorded, and analyzed using the Acqknowledge® software (Biopac). Results are given in microns for artery diameters. Myogenic tone was calculated as the percentage of passive diameter (measured diameter/passive diameter). Flow-mediated dilatation was expressed as percent dilation of active tone.

### Pharmacological profile of isolated mesenteric arteries

Four segments of first-order mesenteric arteries were dissected and mounted on a wire myograph (DMT, Aarhus, DK) as previously described [61]. Briefly, two tungsten wires (40 µm diameter) were inserted into the lumen of the arteries and fixed to a force transducer and a micrometer, respectively. Arteries were bathed in a PSS as described above. Wall tension was applied as described previously. After 45 min, arterial segments viability was tested using a potassium-rich solution (KCl, 80 mmol/L). Endothelial function was then tested using acetylcholine (ACh, 10^–6^ mol/L) after precontraction with phenylephrine (Phe, 10^–6^ mol/L). After washout, a cumulative concentration–response curve to Phe (10^–9^ to 10^–5^ mol/L) was performed. A cumulative concentration–response curve to Ach (10^–9^ to 10^–5^ mol/L) was then performed after precontraction of the artery with Phe to 50% of the maximal response. A cumulative concentration–response curve to SNP (10^–9^ to 10^–5^ mol/L) was then performed in the same conditions.

### Perfused isolated kidney

As previously described [62], the right renal artery was cannulated with a polyethylene catheter (PE-10, 0.28 mm internal diameter, 0.61 mm external diameter, Intramedic) blunt disposable needle passed through the superior mesenteric artery. The kidney was then excised. It was perfused without interruption of kidney flow at 37 °C with PSS. The perfusion solution was dialyzed to reduce contamination, and the pH was adjusted to 7.4. Perfusion rate was 600 µl/min, and perfusion pressure was measured upstream of the renal artery using a pressure transducer (PT-F, Living System, Burlington, VT). Vascular reactivity of the renal vasculature was tested using KCl (40 mmol/L). Endothelium-mediated dilatation was tested using ACh (1 µmol/L) after precontraction with Phe (1 µmol/L). Perfusion flow was then increased by step from 0.3 to 2.0 ml/min.

### Aortic ring and choroid sprouting assay

Aortae from adult *Atg5*^lox/lox^ and *Cdh5*.Cre-*Atg5*^lox/lox^ were cut into 1-mm-thick rings as previously described [63]. Peripheral choroids from adult *Atg5*^lox/lox^ and *Cdh5*.Cre-*Atg5*^lox/lox^ were cut into 1-mm^2^ pieces and cultured as previously described [28]. Briefly, aortic rings and choroidal explants were covered with 20 μL of Matrigel (BD Biosciences, 356,234) and cultured for 6 days in Dulbecco’s modified Eagle’s medium (DMEM, Invitrogen, 10,566–016) containing 10% FCS, 1% penicillin/streptomycin, and 0.2% amphotericin B. The surface covered by the aortic ring or the choroidal explant and the vascular sprouts was measured on day 4 and day 6. Vascular sprouting was expressed as the increase in vascular area between day 4 and day 6 (expressed as percentage). Aortic explants were stained with TRITC-coupled isolectin to stain endothelial cells.

### Mouse lung endothelial cells isolation

Lungs from 8-week adult *Atg5*^lox/lox^ and *Cdh5*.Cre-*Atg5*^lox/lox^ mice were harvested and incubated in 5 mL Dulbecco’s modified Eagle’s medium containing 2 mg/mL collagenase I (Invitrogen, 17,100–017) for 45 min at 37 °C with shaking every 15 min followed by filtering through a 40-μm nylon mesh (BD Falcon, 352,340). The cells were then centrifuged at 1,000* g* for 5 min at 4 °C, re-suspended in buffer 1 (0.1% bovine serum albumin, 2 mM EDTA, pH 7.4, in PBS), and incubated with anti-rat immunoglobulin G-coated magnetic beads (Invitrogen, 1103) pre-coupled with rat anti-mouse platelet/endothelial cell adhesion molecule-1 (PECAM-1; MEC13.3, BD Pharmingen,550,274) for 30 min at 4 °C in an over-head shaker. Beads were separated from the solution with a magnetic particle concentrator (Dynal MPC-S, Invitrogen). The beads were washed five times with buffer 1 and centrifuged for 5 min at 1000* g*, and the supernatant was removed. The purified endothelial cells were then cultured in complemented EGM-2 (PromoCell, 22,010). For western blot analysis, lung endothelial cells (2 × 10^5^) were seeded in 60-mm dishes and cultured for 24 h in ECGM-2 at 37 °C and 5% CO_2,_ followed by 8-h starvation in EBM (PromoCell) before 100 ng/ml VEGF-A treatment when indicated.

### Lentiviral transduction of HUVEC cells

HUVECs (PromoCell) were cultured in ECGM medium (PromoCell) with 1% penicillin/streptomycin (Gibco). Production of HIV1 delta U3 SIN lentiviral particles with VSV-G envelop was carried out by the VVTG facility platform (Necker faculty) by using the lentiviral vector pLVTH (Addgene), containing ATG5-specific shRNA (small-hairpin RNA):

GATCCCCGGCATTATCCAATTGGTTTTTCAAGAGAAAACCAATTGGATAATGCCTTTTTAAGCT

A GFP-coding scramble shRNA vector was used as a control. For lentiviral infection, early passages HUVECs were cultivated in six-well plates. Upon reaching ∼70% confluence, a culture medium containing lentiviral particles to the amount of 10 MOI (multiplicity of infection) was added. At 24 h after infection, the medium was changed. At 10 days after infection, ATG5 knockdown HUVECs and controls were stimulated with recombinant human VEGFA (100 ng/mL) in ECGM medium.

When mentioned, cells were treated with Bafilomycin A1 (Enzo life, 100 nM) for 2 h.

### Measure of the active mitochondrial pool

Staining of mitochondria was performed using Mitotracker Red CMXRos (Invitrogen #M7512, 300 nM) diluted in culture medium and incubated for 30 min at 37 °C. After 3 washes with culture medium, cells were fixed with paraformaldehyde diluted at 4% in phosphate buffer saline (PBS). Images were captured with a SP8 Leica confocal microscope with a lighting module. Measurement of mitochondria footprint and branch length was performed on FIJI (NIH) thanks to the semi-automatic plugin MiNA (Mitochondrial Network Analysis) (https://imagej.net/plugins/mina) [64].

For flow cytometry analysis, cells were incubated 30 min at 37 °C with 300 nM of Mitotracker (Invitrogen #M7512) or 100 nM of Tetramethylrhodamine, Methyl Ester, Perchlorate (TMRM, Invitrogen #T668) diluted in culture medium. After washing with PBS and detachment with Trypsin–EDTA (0.05%) (Gibco #25300096), cells were centrifuged at 1000 rpm for 5 min and resuspended in 100 µL of PBS supplemented with 10% FCS. Measurement of the mitochondrial pool was performed by Flow Cytometry using BD LSRFortessa X-20 Cell Analyzer (BD Biosciences) acquired on BD FACSDiva Software (BD Biosciences) and analyzed on FlowJo software (BD Biosciences).

### RNA sequencing and ingenuity pathway analysis

Total RNA was isolated from freshly sorted lung endothelial cells (LEC) by Trizol isolation method and reverse-transcribed using the Quantitect Reverse Transcription kit (Qiagen) according to the manufacturer’s protocol. RNA sequencing (> 75 M reads, pair ends) was carried out using Illumina TruSeq Stranded mRNA Library Prep Kit and 3 independent mice providing LEC per condition. Differential expression analysis: we used the Bioconductor DESeq2 package [65] to import raw HTSeq counts into R statistical software, obtain size factors and dispersion estimates, and tested for differential expression. We tested genes expressed in at least one sample (FPKM ≥ 0.1) to improve the statistical power of the analysis. We applied a p-value threshold of ≤ 0.05 to define differentially expressed genes. For each supervised analysis, we used the hypergeometric test to identify gene sets overrepresented among significantly deregulated genes. We tested all the gene sets referenced in the MSigDB database from the Broad institute, an extensive collection of annotated gene sets comprising hallmark pathways, curated gene sets, GO gene sets, and computational gene sets defined by previous transcriptional studies. The differentially expressed genes were subjected to Ingenuity Pathway Analysis (Qiagen, Hilden, Germany) to decipher the major biological pathways and regulators emphasized by the significantly deregulated genes (with a *p*-value < 0.05 and fold change > 1.2). Gene Ontology was performed using DAVID Bioinformatics Resources, a high-throughput and integrated data-mining environment, to analyze gene lists derived from high-throughput genomic experiments [66, 67].

### Autophagosome isolation

LC3-positive membrane-bound vesicles were isolated as previously described by Yao et al. [68]. Briefly, primary lung endothelial cells from GFP-LC3 mice were grown on T75 flasks until confluence. Cells were starved in EBM for 4 h, and bafilomycin A1 (100 nM) was added 4 h before autophagosome isolation to enrich cells in autophagosomes. When mentioned, VEGFA (100 ng/mL) treatment was applied. Cells were then washed in cold PBS twice and homogenized in 1 mL cold separation buffer (250 mM sucrose, 1 mM EDTA, 10 mM HEPES + protease inhibitors (Roche)). After centrifugation at 1 000 g for 10 min at 4 °C, the post-nuclear fraction was further centrifuged at 17 000 g for 20 min et 4 °C. The supernatant was discarded and washed/centrifuged twice in cold PBS. The pellet fraction was resuspended in 1 mL separation buffer with 50μL of μMACS anti-GFP magnetic beads (Miltenyi Biotec) and incubated on ice for 1 h. The lysate-beads mixture was applied to μcolumns placed on a μMACS separator and washed 5 times with separation buffer. Autophagosome fraction was then eluted with 20μL of elution buffer, heated at 70 °C for 5 min and stored at – 80 °C until proteomic analysis.

### Nitric oxide assay

Nitric oxide was measured on fresh plasma using an improved Griess method according to manufacturer’s protocol (ab272517, Abcam, The Netherlands). Deproteination was performed prior to assay according to manufacturer’s protocol.

### Proteomic profiling of autophagosomal content and whole proteome

Samples were taken up in Laemmli sample buffer, reduced with 1 mM DTT for 10 min at 75 °C and alkylated using 5.5 mM iodoacetamide for 10 min at RT. Same amount of each sample was loaded on 4–12% gradients gels, gel lanes were cut into small pieces (four fractions for autophagosomal content and five fractions for whole proteome of WT and ATG5 KO samples) and proteins therein were in-gel digested with trypsin (Promega). Tryptic peptides were purified by STAGE tips prior LC–MS/MS measurements. The LC–MS/MS measurements were taken on a QExactive plus mass spectrometer coupled to an EasyLC 1000 nanoflow-HPLC. Peptides were separated on fused silica HPLC-column tip (I.D. 75 μm, New Objective, self-packed with reprosil-Pur 120 C18-AQ, 1.9 μm [Dr. Maisch] to a length of 20 cm) using a gradient of A (0.1% formic acid in water) and B (0.1% formic acid in 80% acetonitrile in water). MaxQuant software (version 1.6.2.10) [69] was used for analyzing the MS raw files for peak detection, peptide quantification and identification using Uniprot mouse database (version April 2016 UniProt, full length) and common contaminants. Carbamidomethylcysteine was used as fixed modification, and oxidation of methionine was set as variable modification. The MS/MS tolerance was set to 20 ppm, and two missed cleavages were allowed for Trypsin/P as enzyme specificity. Based on a forward–reverse database, protein and peptide FDR were set to 0.01, minimum peptide length was set to seven, and at least one unique peptide had to be identified. The match-between run option was set to 0.7 min. MaxQuant results were analyzed using Perseus software (version 1.6.6.0) [70]. Ingenuity Pathway Analysis was performed using a fold-change of |1.2| and a *p* < 0.05 for the whole proteome and a fold-change of |2| and a p < 0.01 for the autophagosome content. As IPA does not tolerate missing values, we imputed a FC value of 25 and 35 for proteomic and autophagosomic data, respectively, when proteins were not detected in any of the 3 replicates.

### Detection of TCA intermediates using a non-targeted metabolomic approach

HUVECs were cultured on T75 flasks in ECGM complete medium (PromoCell) until confluence. After removal of the medium, cells were rinsed twice with 0.22% NaCl aqueous solution, quenched with cold MeOH (Optima LC/MS grade) and stored at − 80 °C until analysis. Briefly, TCA cycle intermediates were extracted by adding to 1 million cells aliquots a mixture of cold H_2_O/MeOH. After centrifugation, supernatants were evaporated and reconstituted in an aqueous solution with 2% of MeOH prior to the UHPLC-HRMS (Ultra High-Pressure Liquid Chromatography-High Resolution Mass Spectrometry) analysis. This was performed on a Thermo Scientific Q Exactive mass spectrometer (Thermo Fisher Scientific, Bremen, Germany), operating with a heated electrospray ionization source in negative mode, coupled to a Dionex UltiMate® 3000 UHPLC (Dionex, Sunnyvale, CA, U.S.A.) equipped with a Phenomenex Luna Omega Polar C18 (150 mm × 2.10 mm, 1.6 µm, 100 Å) UHPLC column. Ionization conditions and MS parameters were identical to those previously described as well as chromatographic settings [71–74]. Furthermore, internal quality controls (QCs, generated by mixing all the samples together before extraction and treated like other samples) were used to validate the analysis and injected every 5 biological samples. Finally, obtained data were normalized to sample DNA content (NanoDrop 2000, Thermo Fisher Scientific, Waltham, MA, USA).

### Histopathology and immunofluorescence

#### Aortas

Aortas were isolated from Atg5^lox/lox^ and Cdh5.Cre-Atg5^lox/lox^ mice following intracardial injection of 2% PFA prepared in 1 × PBS^Ca2+Mg2+^. After dissection, the thoracic aortas were flat-mounted and fixed for 30 min in PFA 4% at RT. Following PBS washes, aortas were blocked and permeabilized in TNBT (0.1 M Tris pH 7.4; NaCl 150 mM; 0.5% blocking reagent from Perkin Elmer, 0.5% Triton X-100) or TBST + 3% BSA for 4 h at room temperature. Aortas were incubated with anti-VEGFR2, anti-SQSTM1 and anti-VE-Cadherin primary antibodies (Supp Table S10) diluted in blocking buffer overnight at 4 °C, washed in TNT buffer (0.1 M Tris pH 7.4; NaCl 150 mM, 0.5% Triton X-100) or TBST and incubated with appropriate secondary antibody (coupled with Alexa 488/555, Life Technologies, 1:200) diluted in TNB Triton buffer overnight at 4 °C. Tissues were washed and mounted on slides in fluorescent mounting medium (Dako or Fluoromount). Images were acquired using a Leica TCS SP8 confocal microscope with lightning module.

#### Hearts

Hearts were immersed in 10% formalin and embedded in paraffin. For heart Sects. (4 μm-thick), cell membranes were stained using a wheat germ agglutinin (WGA) coupled to FITC (Vector laboratories, FL1021) and capillaries were stained with Griffonia simplicifolia lectin-I coupled to TRITC (Vector laboratories, RL1102). The nuclei were stained with DAPI. Heart sections were also processed for Masson’s trichrome and picro-sirius stainings. Photomicrographs were taken with an Axiophot Zeiss photomicroscope.

#### Primary lung endothelial cells and HUVECs

Cells were grown on ibiTreat 18 well slides (Ibidi). Cells were fixed in ice-cold methanol for 20 min, washed twice in PBS, and stored at 4 °C until use. For VEGFR2 membrane staining, cells were starved in endothelial basal medium (EBM) (PromoCell) for 4 h, then placed on ice, and washed twice with cold PBS before incubation with anti-VEGFR2 antibody diluted in EBM for 30 min. After two washes with cold PBS, cells were either immediately fixed in formol 10% for 20 min or incubated with VEGFA (100 ng/mL) for 20 min in EBM at 37 °C before fixation in formol 10% for 20 min. After washes in PBS, cells were stored at 4 °C until use. Cells were incubated in blocking buffer (TBS/T + 3%BSA) for 30 min and then incubated with primary antibodies diluted in blocking buffer overnight at 4 °C. The primary antibodies are listed in Supp Table S9. After washings in TBS-T and incubation with appropriate secondary antibody (coupled with Alexa 488/555, Life Technologies, 1:200) diluted in blocking buffer at room temperature for 2 h, nuclei were stained with Hoechst. Cells were washed and mounted with fluorescent mounting medium (ibidi). Images were acquired using a Leica TCS SP8 confocal microscope with a lightning module. Cell surface VEGFR2 area quantification was performed on FIJI. Colocalization analysis was performed on FIJI using JACOP plugin (https://imagej.nih.gov/ij/plugins/track/jacop2.html) with Mander's coefficient and manual thresholding [75].

### Western blot

Protein extraction was performed by using RIPA extraction buffer containing phosphatase and protease inhibitors (Roche). Proteins were separated by 10% or 12% SDS-PAGE and transferred to nitrocellulose or polyvinylidene difluoride membranes. Membranes were incubated overnight with the primary antibody before incubation with the appropriate peroxidase-labeled secondary antibody for 1 h. The primary antibodies are listed in Supp Table S9. The detection of specific signals was visualized by ECL detection according to the manufacturer’s instructions (Bio-Rad, 170–5061). Membranes were stripped with 0.1 M glycine pH = 2.8 for 1 h before re-blotting. We imaged membranes using the Bio-Rad ChemiDOC + imaging system or LAS4000 imaging system.

### Statistics

Data are expressed as individual plots and mean ± SEM. In vivo experiments were performed on at least 5 mice per group. Statistical analyses were calculated with GraphPad Prism (GraphPad software). Comparison between two groups was made with a Student’s *t test* or Mann–Whitney U test. Comparisons between multiple groups were made with one-way ANOVA or two-way ANOVA when appropriate, followed by Fisher’s LSD *post-hoc* test. A* P* value < 0.05 was considered statistically significant.

## Supplementary Information

Below is the link to the electronic supplementary material.

Supplementary file1 (PDF 17787 KB)

Supplementary file2 (PDF 353 KB)

Supplementary file3 (PDF 40 KB)

Supplementary file4 (PDF 96 KB)

Supplementary file5 (PDF 25 KB)

Supplementary file6 (PDF 181 KB)

Supplementary file7 (PDF 65 KB)

Supplementary file8 (DOCX 33 KB)

Supplementary file9 (PDF 6097 KB)

## Data Availability

The mass spectrometry proteomics data have been deposited to the ProteomeXchange Consortium via the PRIDE [76] partner repository with the dataset identifier PXD042431. The transcriptomic data have been deposited to GEO repository with the dataset identifier GSE235785.
