# Supplementary material for: Autophagy protein 5 controls flow-dependent endothelial functions
Source: Cell Mol Life Sci. 2023 Jul 18;80(8):210. doi: 10.1007/s00018-023-04859-9 (PMC10352428; doi:10.1007/s00018-023-04859-9)
Supplement: Supplementary file 1 — Supplementary file1 (PDF 17787 KB) [file 18_2023_4859_MOESM1_ESM.pdf]

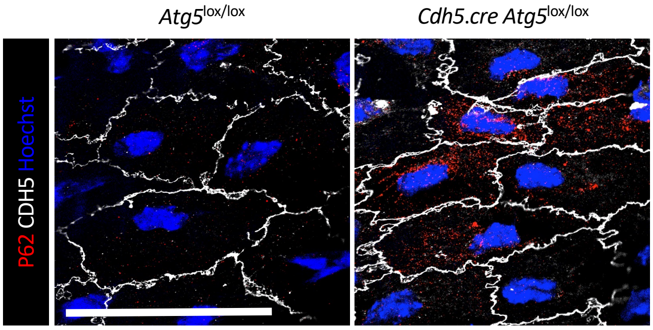

Supplementary Figure S1: Endothelial-selective Atg5 deletion induces endothelial autophagy deficiency. Immunofluorescence of P62 (red) and CDH5 (white) in flat mount aorta from *Atg5*$^{lox/lox}$ and *Cdh5.cre Atg5*$^{lox/lox}$ mice showing P62 accumulation in endothelial cells. Nuclei were stained with hoechst (blue). n=3 mice per genotype. Scale bar 25µm.

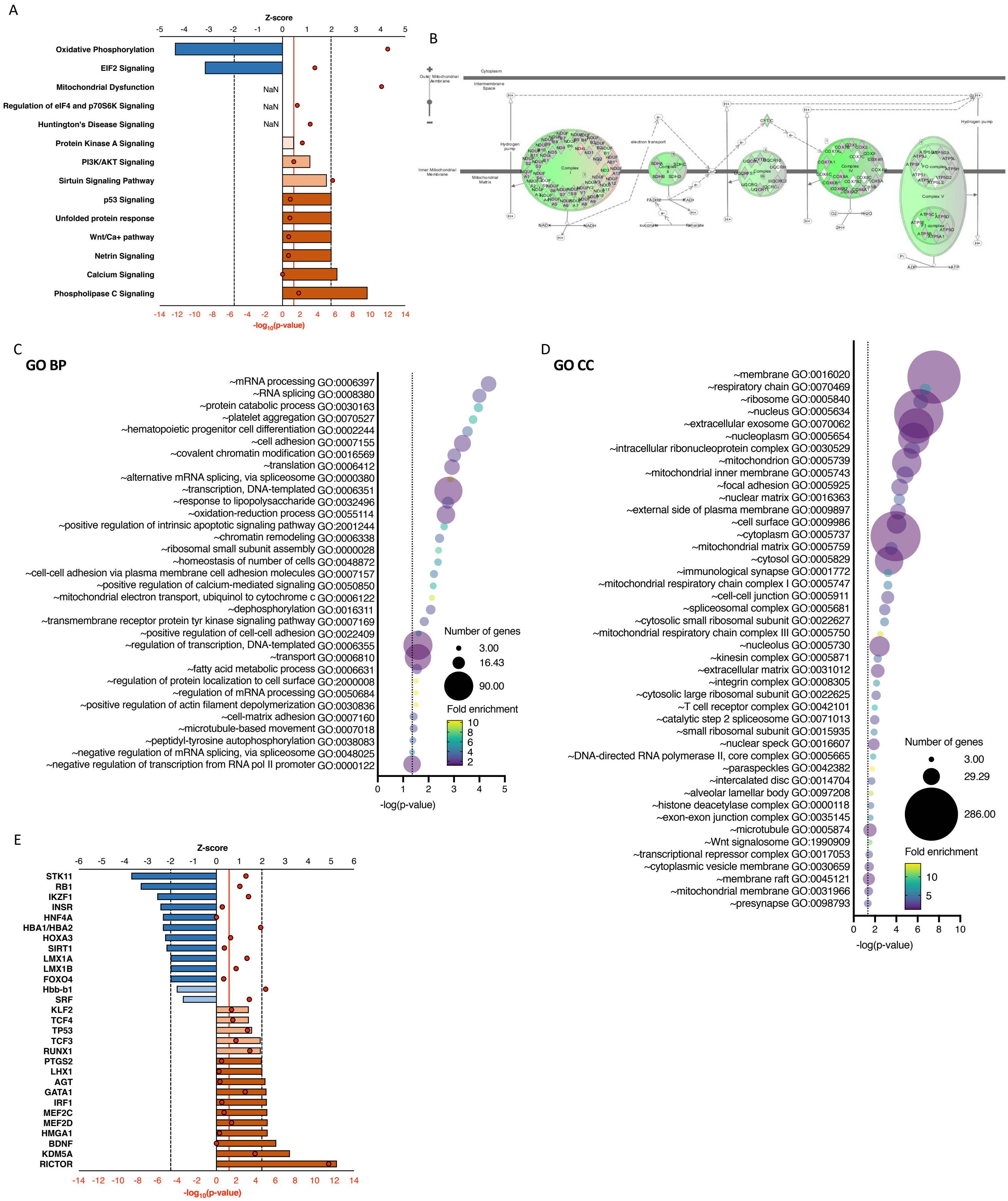

Supplementary Figure S2: IPA and GO analysis of DEGs in ATG5-deficient primary endothelial cells. RNA sequencing data from lung primary endothelial cells isolated from *Atg5*$^{lox/lox}$ and *Cdh5.cre Atg5*$^{lox/lox}$ mice were analyzed using IPA and GO. (A) Z-score and p-value of selected canonical pathways downregulated or upregulated in ATG5-deficient endothelial cells. (B) Representation of genes of the oxidative phosphprylation pathway differentially expressed in ATG5-deficient endothelial cells. (C-D) GO analysis of some biological processes (GO BP, C) and cellular components (GO CC, D) differentially regulated in ATG5-deficient endothelial cells. (E)) Z-score and p-value of upstream regulators predicted by IPA to be downregulated or upregulated in ATG5-deficient endothelial cells.

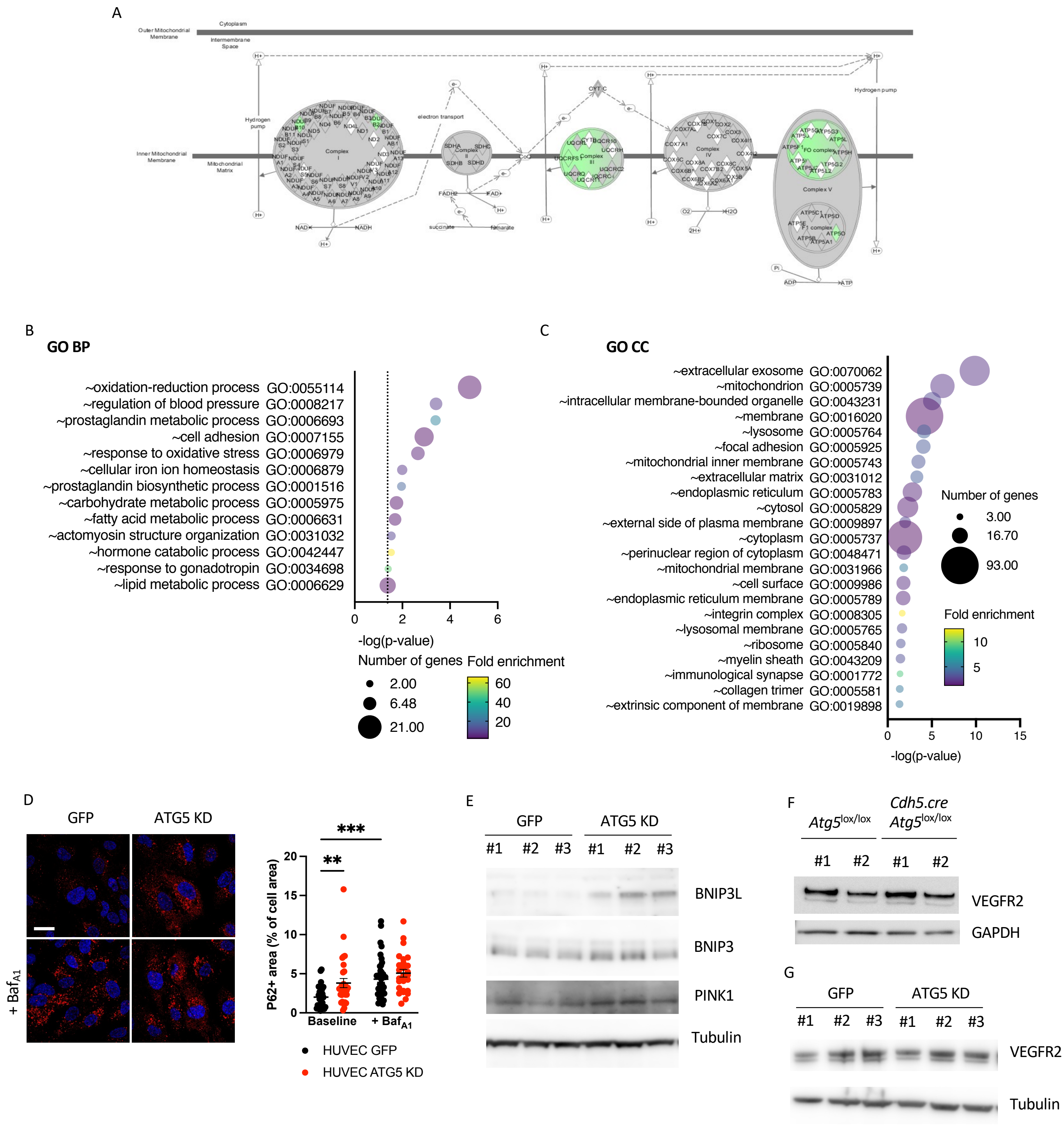

Supplementary Figure S3: IPA and GO analysis of the differentially expressed proteins in ATG5-deficient primary endothelial cells. Proteomic data from lung primary endothelial cells isolated from *Atg5*$^{lox/lox}$ and *Cdh5.cre Atg5*$^{lox/lox}$ mice were analyzed using IPA and GO. (A) Representation of proteins of the oxidative phosphprylation pathway differentially expressed in ATG5-deficient endothelial cells.(B-C) GO analysis of some biological processes (GO BP, B) and cellular components (GO CC, C) differentially regulated in ATG5-deficient endothelial cells. (D) P62 immunofluorescence (red) in control and ATG5 KD HUVECs treated or not with bafilomycin A1 (100nM) for 2 hours. Nuclei were stained with Hoechst (blue). n=3 replicates. Scale bar 25 µm. 2-way ANOVA: ATG5 KD effect: p=0.005, Bafilomycin A1 effect: p= 0.0001. *Post-hoc* Fisher LSD's test: Baseline:HUVEC GFP vs. Baseline:HUVEC ATG5 KD: p=0.0064, Baseline:HUVEC GFP vs. + Baf$_{A1}$:HUVEC GFP : p=0.0002, Baseline:HUVEC ATG5 KD vs. Baf$_{A1}$:HUVEC ATG5 KD: p=0.059, Baf$_{A1}$:HUVEC GFP vs. + Baf$_{A1}$:HUVEC ATG5 KD : p=0.22 (E) Immunoblot of BNIP3L, BNIP3 and PINK1 expression in HUVECs transduced with a lentivirus coding for a shRNA against ATG5 or a scramble shRNA. (F,G) Immunoblot of VEGFR2 expression in lung primary endothelial cells isolated from *Atg5*$^{lox/lox}$ and *Cdh5.cre Atg5*$^{lox/lox}$ mice (representative of n=4 mice) (F) and HUVECs transduced with a lentivirus coding for a shRNA against ATG5 or a scramble shRNA (G). (E-G) Lanes #1, #2 and #3 represent different replicates. GAPDH or Tubulin were used as loading control.

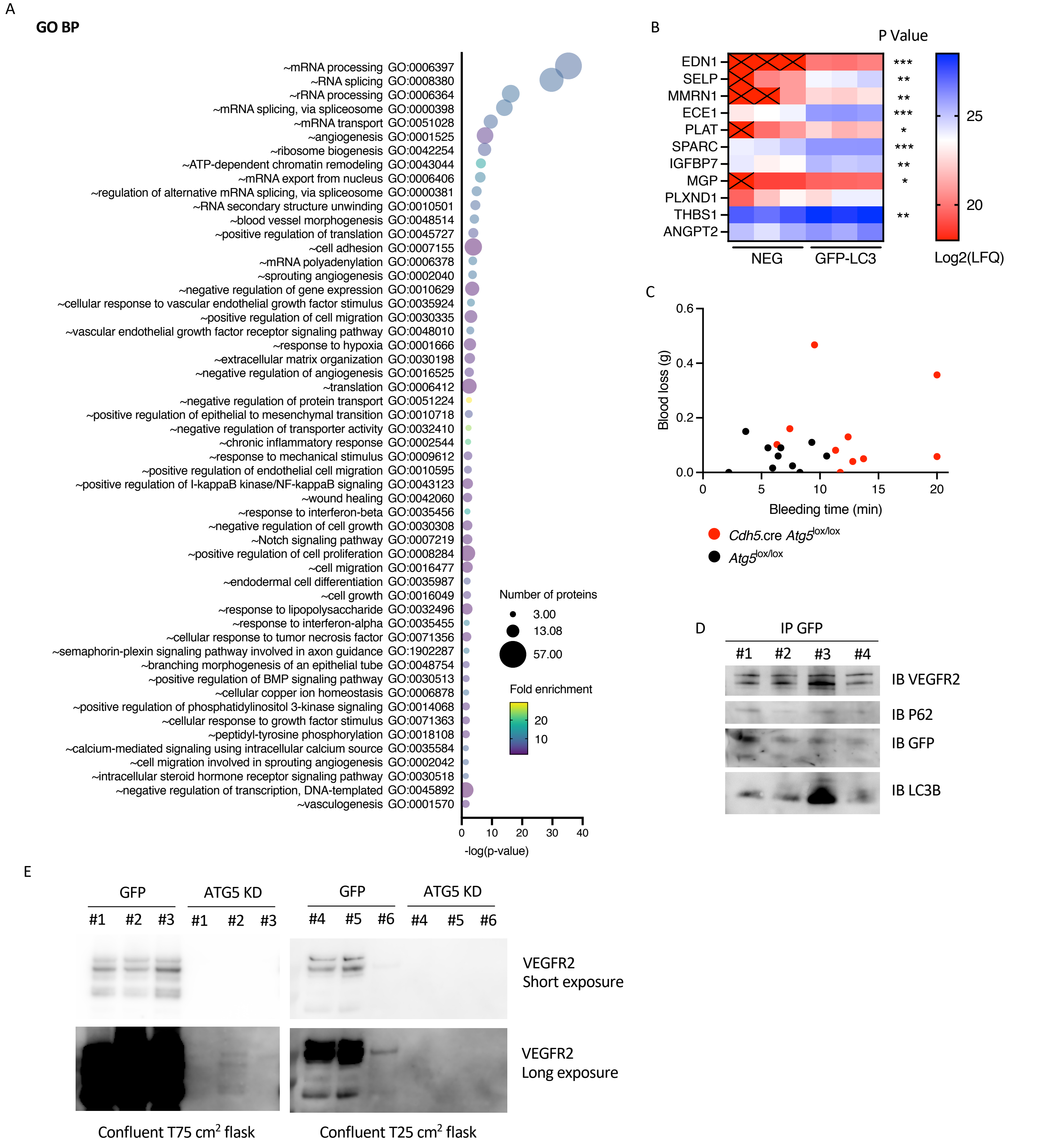

Supplementary Figure S4: Enriched proteins in autophagosomes. Proteomic data from autophagosomes isolated from lung primary endothelial cells isolated of GFP-LC3 mice. (A) GO analysis of some biological processes (GO BP) enriched in autophagosomes from endothelial cells. (B) Heatmap showing proteins of the Weibel palade bodies enriched in autophagosomes. * p<0.05, ** p<0.01, *** p<0.001 between GFP-immunoprecipitated autophagosomes and negative isolation. (C) Bleeding assay in *Atg5*$^{lox/lox}$ and *Cdh5.cre Atg5*$^{lox/lox}$ mice showing increased bleeding time and blood loss in *Cdh5.cre Atg5*$^{lox/lox}$ mice. n=10 mice per genotype. D. Immunoblot analysis of VEGFR2, P62, GFP and LC3B expression in autophagosomes isolated by GFP immunoprecipitation. Lanes #1, #2, #3 and #4 represent 4 different protein extracts from 4 different experiments. E. Immunoblot analysis of VEGFR2 expression in plasma membrane extracts from control or ATG5 KD HUVECs after 6h of starvation in EBM medium without supplements. For each of the 6 replicates, the whole lysate was loaded on the gels. For replicates 1 to 3, plasma membrane extracts were prepared from a T75 confluent flask and for replicates 4 to 6 from a T25 confluent flask.

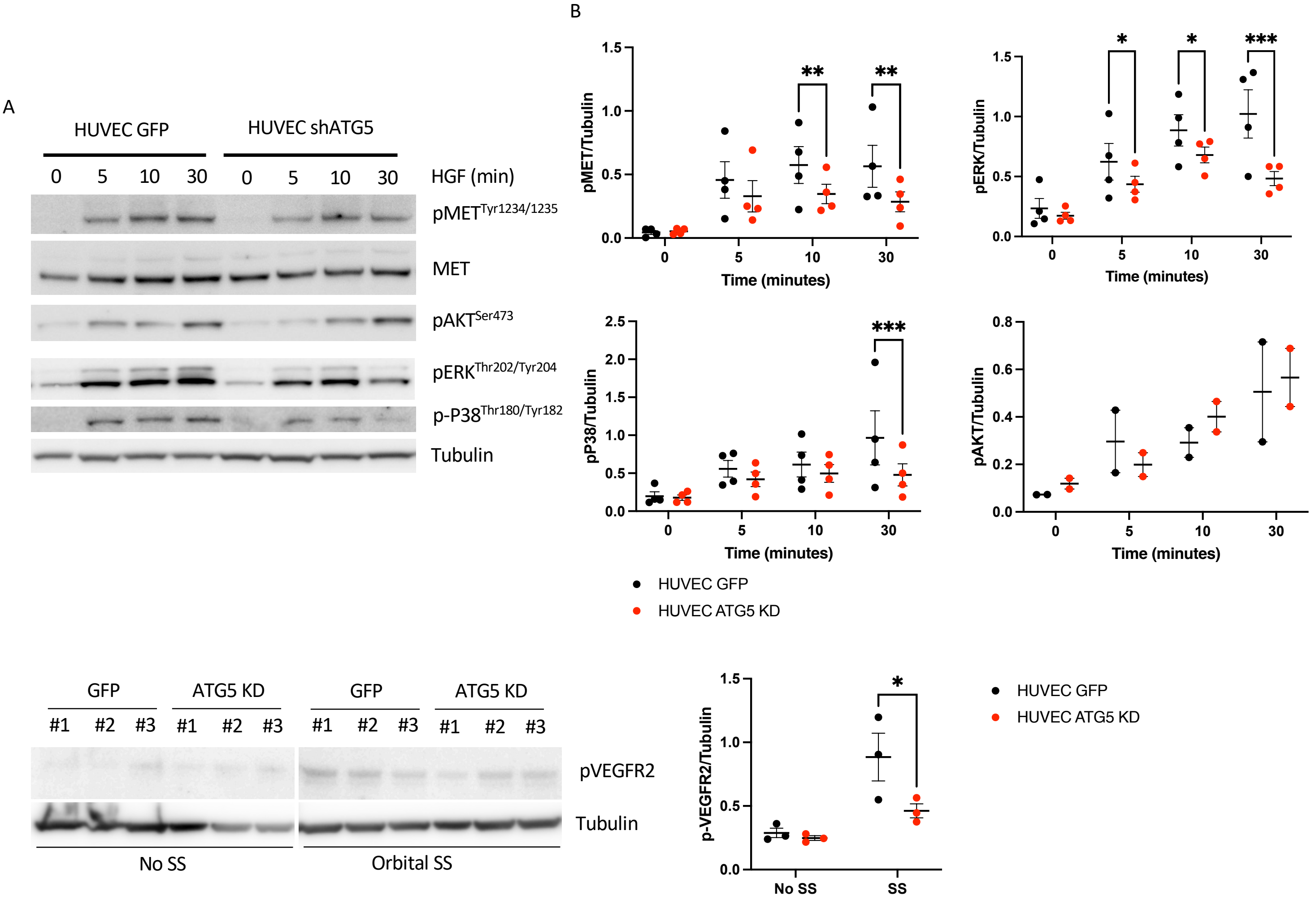

Supplementary Figure S5: MET and VEGFR2 signaling in ATG5-deficient endothelial cells. (A) Western blot analysis of phospho-MET, MET, phospho-AKT, phospho-ERK and phospho-P38 expression in control and ATG5 KD HUVECs after HGF treatment. n=4 replicates. Tubulin was used as loading control (B) Associated quantifications. 2-way ANOVA were used to analyze effects of ATG5 KD on HGF response over time. pMET: time effect p=0.0037, ATG5 KD effect p=0.0542, time x ATG5 KD effect p=0.0521. pERK: time effect p<0.001, ATG5 KD effect p=0.0762, time x ATG5 KD effect p=0.0151. pP38: time effect p=0.021, ATG5 KD effect p=0.0841, time x ATG5 KD effect p=0.0542. pAKT: time effect p=0.086, ATG5 KD effect p=0.75, time x ATG5 KD effect p=0.44. Significant Fisher's LSD post-hoc results are shown on the graphs: * p<0.05, ** p<0.01, *** p<0.001. n=4 replicates except for pAKT were n=2 replicates. (C) Western blot analysis of phospho-VEGFR2 in control and ATG5 KD HUVECs after orbital shear-stress for 10min or in static condition. n=3 replicates. Tubulin was used as loading control.

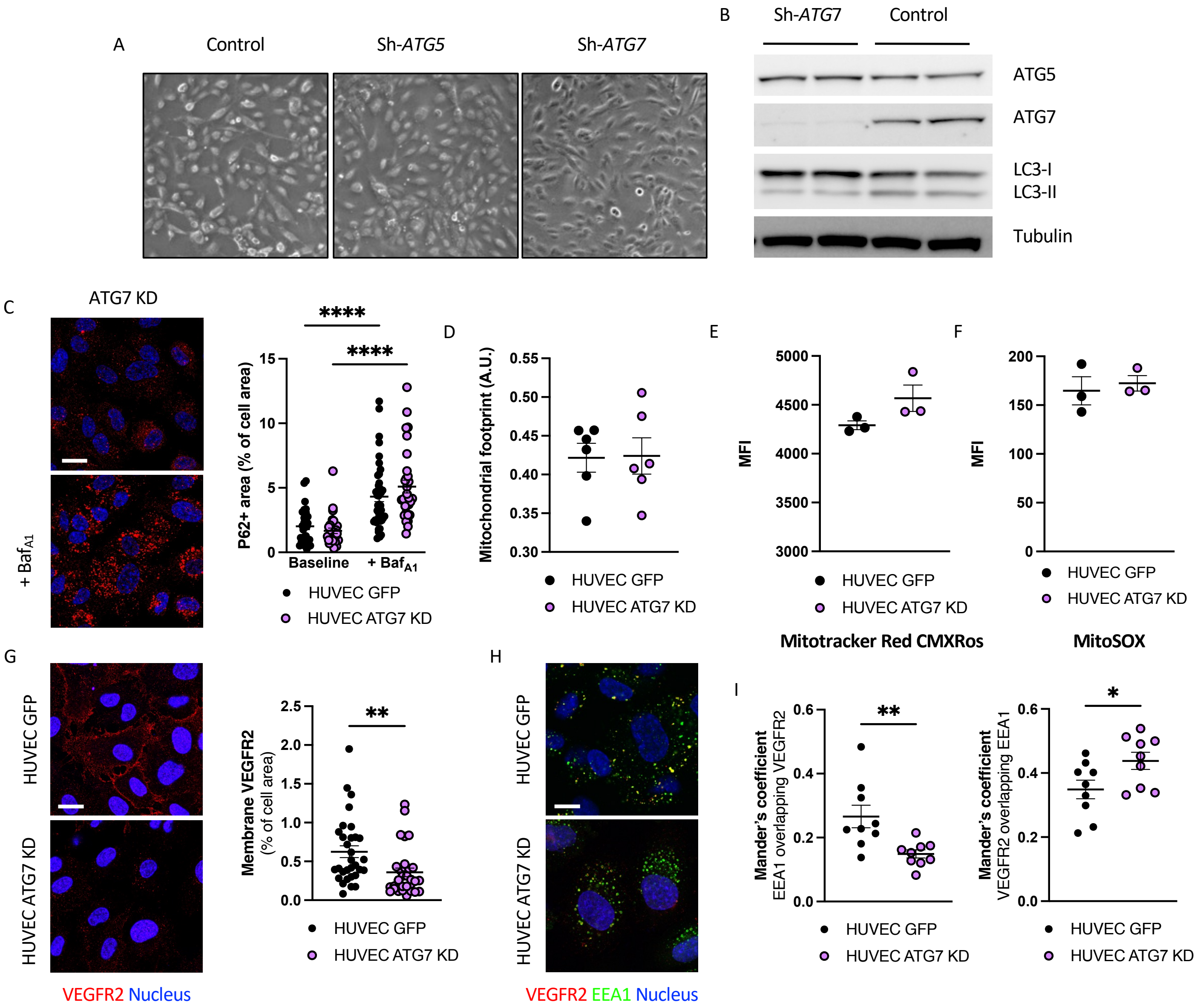

Supplementary Figure S6: ATG7 KD HUVECs have no mitochndrial abnormality and decreased plasma membrane VEGFR2 expression. (A) Representative pictures of Control, ATG5 KD and ATG7 KD HUVECs. (B) Western blot analysis of ATG5, ATG7 and LC3-B expression in HUVECs transduced with a scramble shRNA (Control) or a sh-ATG7. Tubulin was used as loading control. n=3 replicates. (C) P62 immunofluorescence (red) in ATG7 KD HUVECs treated or not with bafilomycin A1 (100nM) for 2 hours. Nuclei were stained using DAPI (blue). n=3 replicates. Scale bar 25µm (D) Quantification of the mitochondrial footprint in control and ATG7 KD HUVECs after incubation with mitotracker Red CMXRos. (E,F) Flow cytometry analysis of the active pool of mitochondria in control and ATG7 KD HUVECs using mitotracker Red CMXRos (E) and mitochondrial ROS production using MitoSOX (F) stainings. n=3 experimental replicates. (G) Immunofluorescence of cell surface VEGFR2 (red) expression in control and ATG7 KD HUVECs. Nuclei were stained with Hoechst. n=3 replicates. Scale bar 25µm. (H) Immunofluorescence of VEGFR2 (red) and EEA1 (green) expression in control and ATG7 KD HUVECs after 20min of VEGFA treatment. Cell surface VEGFR2 was stained to visualize internalization upon VEGFA treatment. Nuclei were stained with Hoechst. n=3 replicates. Scale bar 25µm. (I) Quantification of VEGFR2 staining overlapping EEA1 staining and EEA1 staining overlapping VEGFR2 staining in control and ATG7 KD HUVECs after 20min of VEGFA treatment.

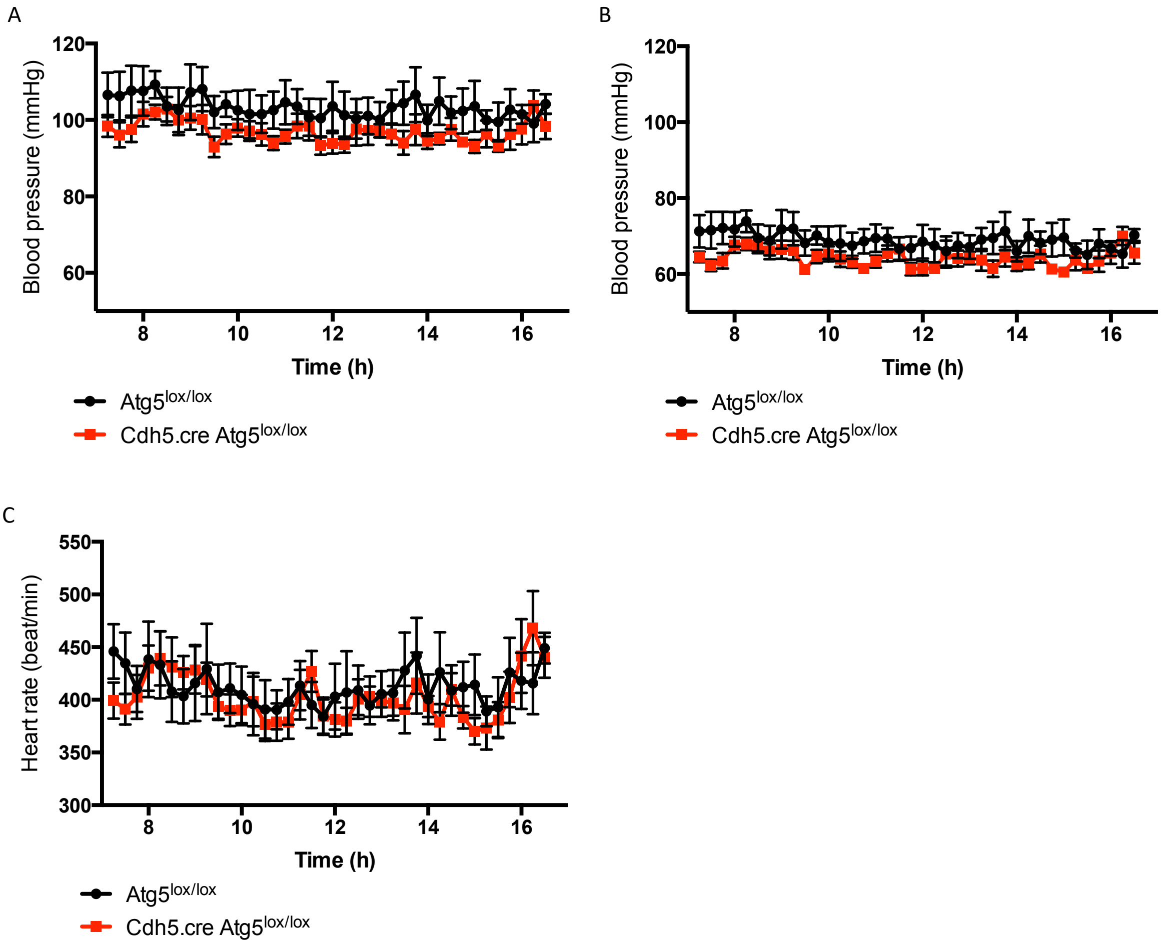

Supplementary Figure S7: *Cdh5*.cre-*Atg5*$^{lox/lox}$ mice have normal blood pressure in adult. (A-C) Representatives graph of telemetry recording in 12 weeks-old *Atg5*$^{lox/lox}$ and *Cdh5*.cre-*Atg5*$^{lox/lox}$ mice. Day measurements of systolic (A) and diastolic (B) blood pressure and heart rate (C). Values are means ± SEM of n=6 mice.

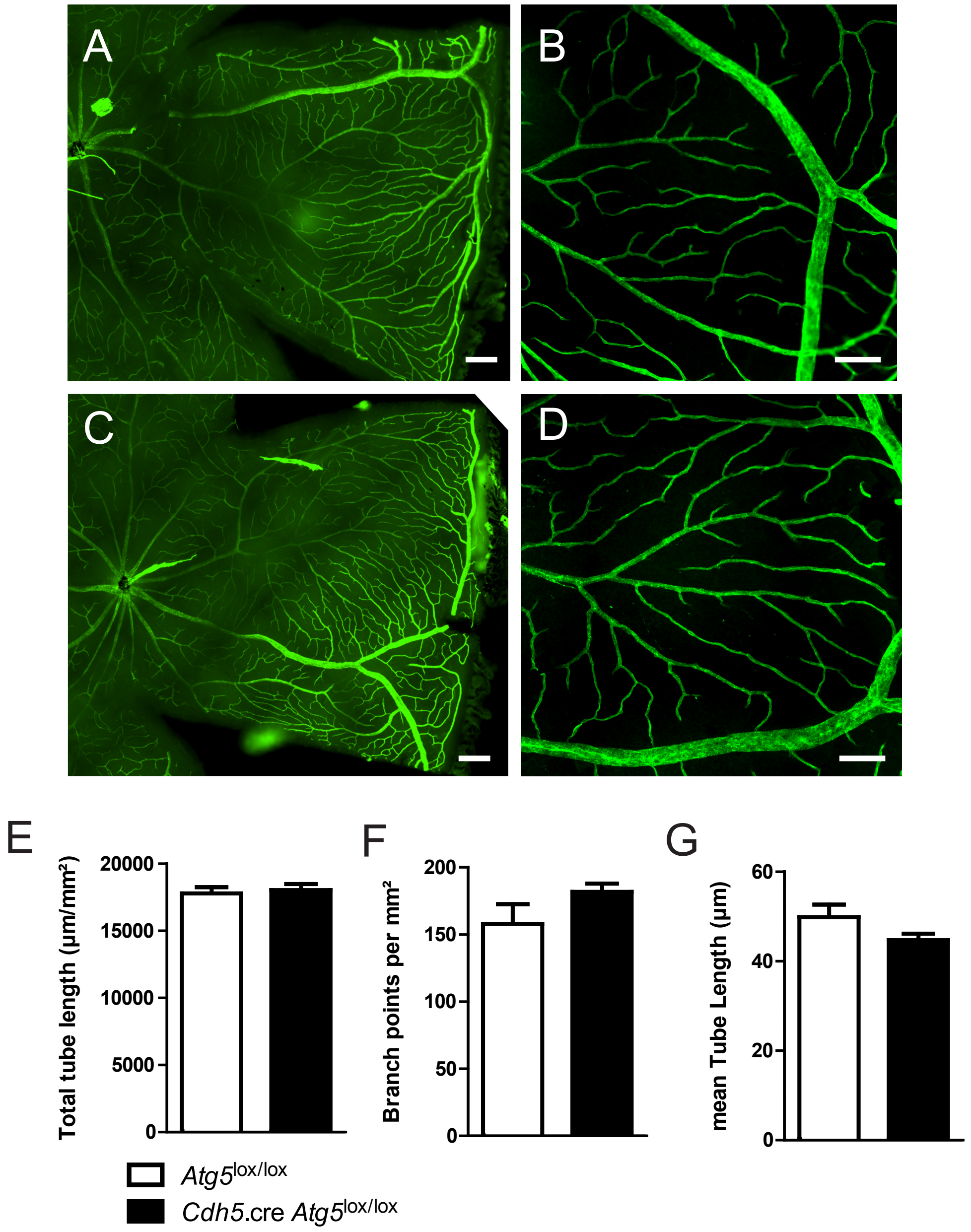

Supplementary Figure S8: *Cdh5*.cre-*Atg5*$^{lox/lox}$ mice have normal retinal vascularization in adult. (A-D) Representatives images of the vascular density (isolectin B4 in green) in adult retinas from 12 weeks-old *Atg5*$^{lox/lox}$ (A, B) and *Cdh5*.cre-*Atg5*$^{lox/lox}$ (C, D) mice. Scale bar 200µm (left panel) and 50µm (right panel). Quantification of total tube length (E), number of branch points per mm^2 (F) and mean tube length (G). Values are means ± SEM of n=4 mice.

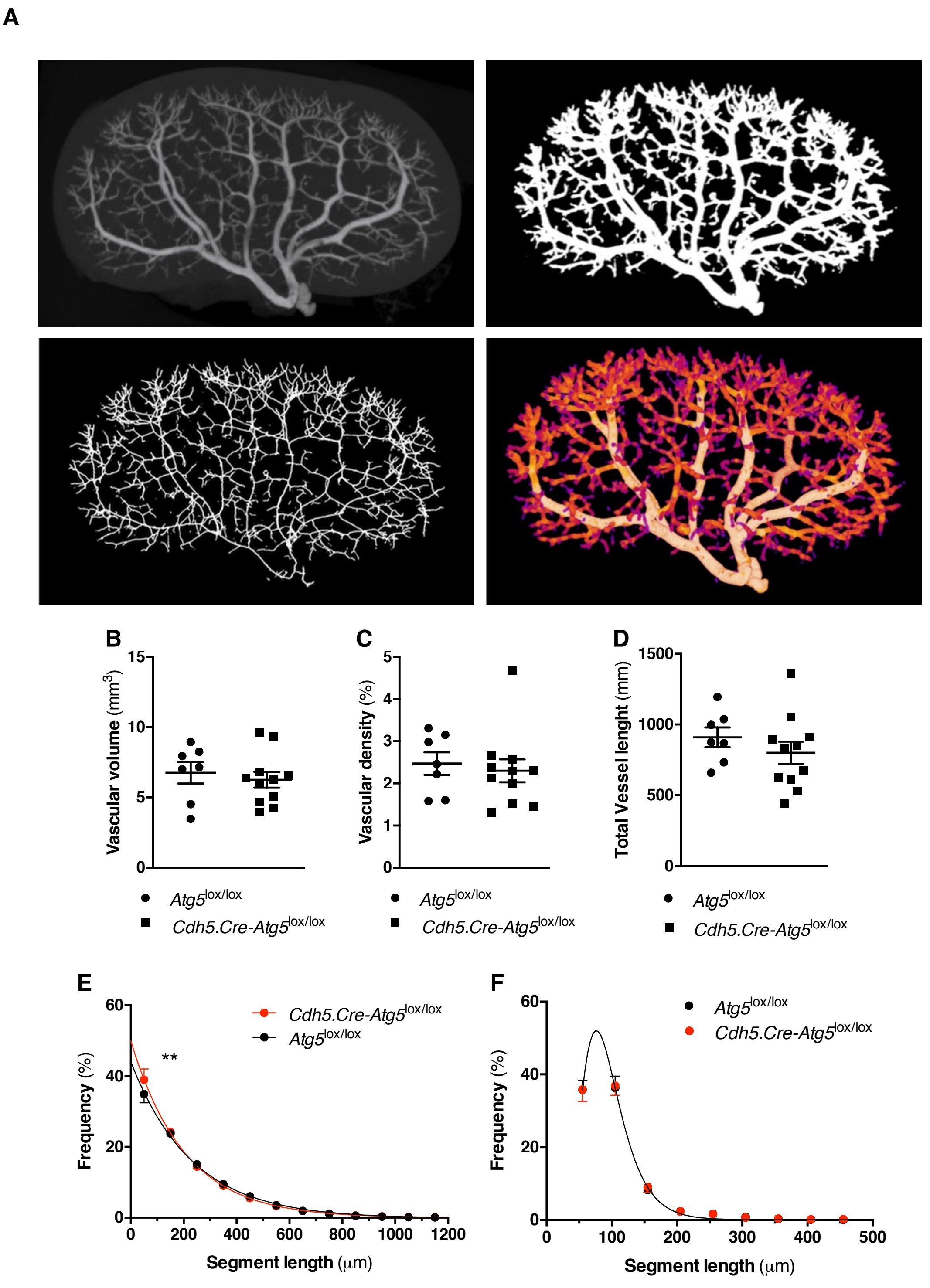

Supplementary Figure S9: Microangioscanner assessment of renal vascularisation do not identified differences in mice with selective-endothelial autophagy deficiency. Images were analyzed by FIJI software from the 3D microscanner acquisition (A). A mask is determined from the selected pixels and allow to determine the fractal dimension. The skeleton is then extracted from the mask and allow to measures branch length. A diameter map is extracted from the mask and allow to measure vessel diameters. Measurement of the vascular volume (B), vascular density normalized to renal volume (C) and total vessel length (D) in Atg5lox/lox and Cdh5-cre Atg5lox/lox mice. (E) Segment length frequency distribution. Abscissa: center of segment length intervals (100 µm range). Ordinate: number of segments per interval, in % of total number. Dots and error bars are mean value and SEM. Lines: non linear fits by one-order exponential decay. Statistical comparison by non linear regression (F test) using GraphPad Prism. ** P<0.01 (F) Segment diameter frequency distribution. Abscissa: center of segment diameter intervals (50 µm range). Ordinate: number of segments per interval, in % of total number. Dots and error bars are mean value and SEM. Line: non linear fits by Log Gaussian law. Statistical comparison by non linear regression (F test) using GraphPad Prism non significant (P>0.05)

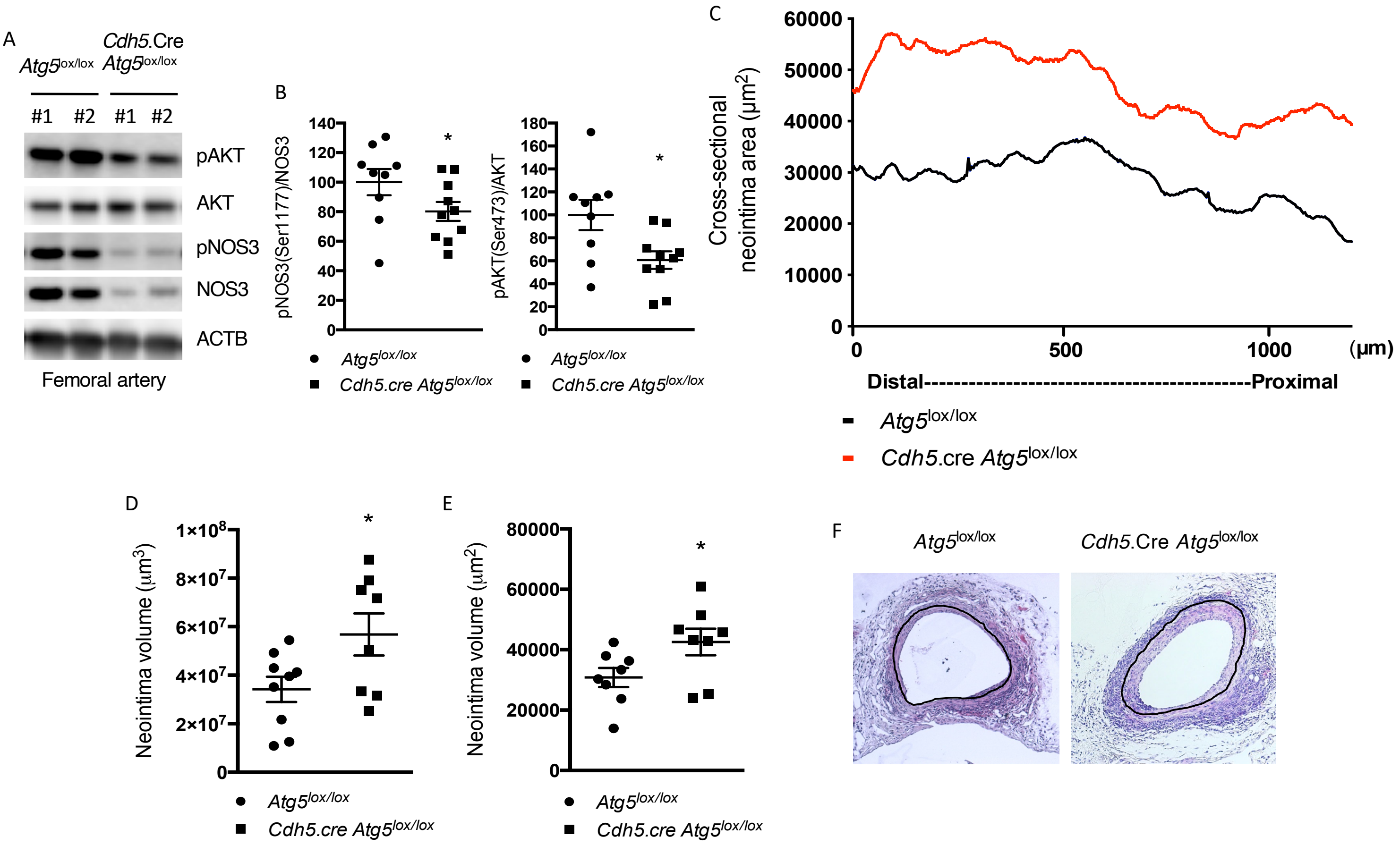

Supplementary Figure S10: *Cdh5*.Cre-*Atg5*$^{lox/lox}$ mice have increased neointimal lesions in a model of intraluminal injury. (A, B) Representative immunoblot (A) and quantifications (B) of AKT, phospho-AKT, eNOS (NOS3) and phospho-eNOS expression in freshly isolated femoral arteries from *Atg5*$^{lox/lox}$ and *Cdh5*.cre.*Atg5*$^{lox/lox}$ mice. β Actin (ACTB) was used as loading control. In A, lanes #1 and #2 represent 2 different protein extracts from 2 different mice. Values are individual values and mean ± SEM of n=9 *Atg5*$^{lox/lox}$ mice and n=11 *Cdh5*.cre-*Atg5*$^{lox/lox}$ mice. * $p < 0.05$. (C-F) Endothelial autophagy deficiency increases neointimal lesions in a model of intraluminal injury. In femoral arteries from *Atg5*$^{lox/lox}$ and *Cdh5*.Cre-*Atg5*$^{lox/lox}$ mice subjected to wire injury, neointimal lesion distribution (C) and neointimal volume (D) were determined by OPT. Maximal cross-sectional narrowing (E) was measured in serial sections stained with Masson's trichrome. Values are means ± SEM of n=8 *Cdh5*.Cre-*Atg5*$^{lox/lox}$ and n=9 *Atg5*$^{lox/lox}$ mice. * $p<0.05$ vs. *Atg5*$^{lox/lox}$ controls. (F) Representatives images of hematoxylin/eosin coloration on femoral cross section from *Atg5*$^{lox/lox}$ and *Cdh5*.Cre-*Atg5*$^{lox/lox}$ mice 10 days after wire injury. The dark line shows the neointimal lesion.
