# Supplementary material for: Autophagy protein 5 controls flow-dependent endothelial functions
Source: Cell Mol Life Sci. 2023 Jul 18;80(8):210. doi: 10.1007/s00018-023-04859-9 (PMC10352428; doi:10.1007/s00018-023-04859-9)
Supplement: Supplementary file 2 — Supplementary file2 (PDF 353 KB) [file 18_2023_4859_MOESM2_ESM.pdf]

ID	Symbol	Expr p-value	Expr FDR (q-value)	Expr Log Ratio	Entrez Gene Name	Location	Type(s)
**ENSMUSG00000035202**	**LARS2**	**4,61E-27**	**2,21E-23**	**-1,696**	**leucyl-tRNA synthetase 2, mitochondrial**	**Cytoplasm**	**enzyme**
**ENSMUSG00000018008**	**CYTH4**	**2,95E-05**	**2,49E-02**	**1,04**	**cytohesin 4**	**Cytoplasm**	**other**
ENSMUSG00000058626	CAPN11	7,26E-05	1,00E+00	-4,609	calpain 11	Cytoplasm	peptidase
ENSMUSG00000064358	MT-CO3	2,03E-04	9,83E-02	-0,781	cytochrome c oxidase III	Cytoplasm	enzyme
ENSMUSG00000024194	CUTA	2,06E-04	9,83E-02	-0,355	cutA divalent cation tolerance homolog	Cytoplasm	other
ENSMUSG00000037795	N4BP2	2,12E-04	9,83E-02	0,56	NEDD4 binding protein 2	Cytoplasm	enzyme
ENSMUSG00000027804	PPID	2,48E-04	1,02E-01	0,416	peptidylprolyl isomerase D	Cytoplasm	enzyme
ENSMUSG00000000275	TRIM25	2,65E-04	1,03E-01	0,351	tripartite motif containing 25	Cytoplasm	transcription regulator
ENSMUSG00000000409	LCK	2,73E-04	1,03E-01	1,503	LCK proto-oncogene, Src family tyrosine kinase	Cytoplasm	kinase
ENSMUSG00000022551	CYC1	3,58E-04	1,22E-01	-0,34	cytochrome c1	Cytoplasm	enzyme
ENSMUSG00000020946	GOSR2	4,80E-04	1,46E-01	-0,357	golgi SNAP receptor complex member 2	Cytoplasm	transporter
ENSMUSG00000028071	SH2D2A	5,20E-04	1,48E-01	0,945	SH2 domain containing 2A	Cytoplasm	other
ENSMUSG00000020444	GUK1	5,22E-04	1,48E-01	-0,338	guanylate kinase 1	Cytoplasm	kinase
ENSMUSG00000042625	SAFB2	5,53E-04	1,48E-01	0,586	scaffold attachment factor B2	Cytoplasm	other
ENSMUSG00000026941	MAMDC4	5,65E-04	1,48E-01	0,902	MAM domain containing 4	Cytoplasm	other
ENSMUSG00000080902	YWHAQ	6,40E-04	1,56E-01	1,025	tyrosine 3-monooxygenase/tryptophan 5-monooxygenase activation protein theta	Cytoplasm	other
ENSMUSG00000041642	KIF21B	8,21E-04	1,81E-01	0,746	kinesin family member 21B	Cytoplasm	other
ENSMUSG00000002699	LCP2	1,07E-03	2,25E-01	0,969	lymphocyte cytosolic protein 2	Cytoplasm	other
ENSMUSG00000025135	ANAPC11	1,25E-03	2,36E-01	-0,385	anaphase promoting complex subunit 11	Cytoplasm	enzyme
ENSMUSG00000027602	MAP1LC3A	1,42E-03	2,57E-01	-0,593	microtubule associated protein 1 light chain 3 alpha	Cytoplasm	other
ENSMUSG00000011263	EXOC3L2	1,55E-03	2,64E-01	-0,5	exocyst complex component 3 like 2	Cytoplasm	other
ENSMUSG00000024182	AXIN1	1,58E-03	2,64E-01	0,382	axin 1	Cytoplasm	other
ENSMUSG00000060376	BCKDHA	1,62E-03	2,64E-01	-0,289	branched chain keto acid dehydrogenase E1 subunit alpha	Cytoplasm	enzyme
ENSMUSG00000014602	KIF1A	1,63E-03	1,00E+00	1,501	kinesin family member 1A	Cytoplasm	other
ENSMUSG00000026790	ODF2	1,65E-03	2,65E-01	0,3	outer dense fiber of sperm tails 2	Cytoplasm	other
ENSMUSG00000025330	PADI4	1,87E-03	1,00E+00	1,749	peptidyl arginine deiminase 4	Cytoplasm	enzyme
ENSMUSG00000025486	SIRT3	1,90E-03	2,83E-01	-0,502	sirtuin 3	Cytoplasm	enzyme
ENSMUSG00000020395	ITK	1,99E-03	2,92E-01	0,86	IL2 inducible T cell kinase	Cytoplasm	kinase
ENSMUSG00000058076	SDHC	2,14E-03	3,04E-01	-0,572	succinate dehydrogenase complex subunit C	Cytoplasm	enzyme
ENSMUSG00000039450	DCXR	2,18E-03	3,04E-01	-0,482	dicarbonyl and L-xylulose reductase	Cytoplasm	enzyme
ENSMUSG00000034892	RPS29	2,37E-03	3,14E-01	-0,472	ribosomal protein S29	Cytoplasm	other
ENSMUSG00000040225	PRRC2C	2,45E-03	3,16E-01	0,318	proline rich coiled-coil 2C	Cytoplasm	other
ENSMUSG00000037112	SIK2	2,54E-03	3,17E-01	0,382	salt inducible kinase 2	Cytoplasm	kinase
ENSMUSG00000003477	INMT	2,67E-03	3,19E-01	-0,623	indolethylamine N-methyltransferase	Cytoplasm	enzyme
ENSMUSG00000042632	PLA2G6	2,74E-03	3,25E-01	0,415	phospholipase A2 group VI	Cytoplasm	enzyme
ENSMUSG00000006360	CRIP1	2,81E-03	3,25E-01	-0,439	cysteine rich protein 1	Cytoplasm	other
ENSMUSG00000032172	OLFM2	2,85E-03	3,25E-01	0,5	olfactomedin 2	Cytoplasm	other
ENSMUSG00000031389	ARHGAP4	3,51E-03	3,62E-01	0,695	Rho GTPase activating protein 4	Cytoplasm	other
ENSMUSG00000019054	FIS1	3,93E-03	3,81E-01	-0,301	fission, mitochondrial 1	Cytoplasm	other
ENSMUSG00000017969	PTGIS	4,02E-03	3,82E-01	-0,545	prostaglandin I2 synthase	Cytoplasm	enzyme
ENSMUSG00000042349	IKBKE	4,21E-03	3,92E-01	0,708	inhibitor of nuclear factor kappa B kinase subunit epsilon	Cytoplasm	kinase
ENSMUSG00000049760	MICOS13	4,74E-03	4,03E-01	-0,322	mitochondrial contact site and cristae organizing system subunit 13	Cytoplasm	other
ENSMUSG00000029030	TPRG1L	5,01E-03	4,03E-01	-0,268	tumor protein p63 regulated 1 like	Cytoplasm	other
ENSMUSG00000014195	DNAJC7	5,05E-03	4,03E-01	0,284	DnaJ heat shock protein family (Hsp40) member C7	Cytoplasm	other
ENSMUSG00000002395	USE1	5,08E-03	4,03E-01	-0,278	unconventional SNARE in the ER 1	Cytoplasm	other
ENSMUSG00000035559	MPV17L2	5,22E-03	4,03E-01	-0,348	MPV17 mitochondrial inner membrane protein like 2	Cytoplasm	other
ENSMUSG00000050708	FTL	5,23E-03	4,03E-01	-0,266	ferritin light chain	Cytoplasm	enzyme
ENSMUSG00000027347	RASGRP1	5,33E-03	4,04E-01	0,733	RAS guanyl releasing protein 1	Cytoplasm	other
ENSMUSG00000038650	RNH1	5,35E-03	4,04E-01	-0,511	ribonuclease/angiogenin inhibitor 1	Cytoplasm	other
ENSMUSG00000038286	BPHL	5,58E-03	4,08E-01	-0,646	biphenyl hydrolase like	Cytoplasm	enzyme
ENSMUSG00000090877	Hspa1b	5,70E-03	4,11E-01	0,671	heat shock protein 1B	Cytoplasm	other
ENSMUSG00000027530	FABP12	5,87E-03	1,00E+00	-2,373	fatty acid binding protein 12	Cytoplasm	other
ENSMUSG00000044894	UQCRQ	6,07E-03	4,26E-01	-0,388	ubiquinol-cytochrome c reductase complex III subunit VII	Cytoplasm	enzyme
ENSMUSG00000020340	CYFIP2	6,14E-03	4,26E-01	0,604	cytoplasmic FMR1 interacting protein 2	Cytoplasm	other
ENSMUSG00000091971	HSPA1A/HSPA1B	6,14E-03	4,26E-01	0,615	heat shock protein family A (Hsp70) member 1A	Cytoplasm	enzyme
ENSMUSG00000036880	ACAA2	6,18E-03	4,27E-01	-0,387	acetyl-CoA acyltransferase 2	Cytoplasm	enzyme
ENSMUSG00000018995	NARS2	6,28E-03	4,30E-01	-0,439	asparaginyl-tRNA synthetase 2, mitochondrial	Cytoplasm	enzyme
ENSMUSG00000056071	S100A9	6,39E-03	4,30E-01	1,093	S100 calcium binding protein A9	Cytoplasm	other
ENSMUSG00000062908	ACADM	6,39E-03	4,30E-01	0,464	acyl-CoA dehydrogenase medium chain	Cytoplasm	enzyme
ENSMUSG00000063646	JAKMIP1	6,49E-03	4,30E-01	1,123	janus kinase and microtubule interacting protein 1	Cytoplasm	translation regulator
ENSMUSG00000026213	STK11IP	6,53E-03	4,30E-01	0,496	serine/threonine kinase 11 interacting protein	Cytoplasm	other
ENSMUSG00000065947	MT-ND4L	6,53E-03	4,30E-01	0,32	NADH dehydrogenase, subunit 4L (complex I)	Cytoplasm	enzyme
ENSMUSG00000035439	HAUS8	6,74E-03	4,34E-01	0,531	HAUS augmin like complex subunit 8	Cytoplasm	other
ENSMUSG00000038011	DNAH10	6,79E-03	4,34E-01	0,761	dynein axonemal heavy chain 10	Cytoplasm	other
ENSMUSG00000040883	TMEM205	6,90E-03	4,34E-01	-0,446	transmembrane protein 205	Cytoplasm	other
ENSMUSG00000039208	METRNL	7,36E-03	4,55E-01	-0,611	meteorin like, glial cell differentiation regulator	Cytoplasm	other
ENSMUSG00000021693	KIF2A	7,62E-03	4,62E-01	0,367	kinesin family member 2A	Cytoplasm	other
ENSMUSG00000046330	RPL37A	7,78E-03	4,65E-01	-0,512	ribosomal protein L37a	Cytoplasm	other
ENSMUSG00000022427	TOMM22	7,91E-03	4,71E-01	-0,38	translocase of outer mitochondrial membrane 22	Cytoplasm	transporter
ENSMUSG00000008601	RAB25	8,11E-03	4,75E-01	-0,534	RAB25, member RAS oncogene family	Cytoplasm	enzyme
ENSMUSG00000016255	TUBB1	8,14E-03	4,75E-01	0,826	tubulin beta 1 class VI	Cytoplasm	other
ENSMUSG00000020297	NSG2	8,33E-03	4,80E-01	1,053	neuronal vesicle trafficking associated 2	Cytoplasm	other
ENSMUSG00000012848	RPS5	8,61E-03	4,82E-01	-0,379	ribosomal protein S5	Cytoplasm	other
ENSMUSG00000046727	Cystm1	9,02E-03	4,88E-01	-0,403	cysteine-rich transmembrane module containing 1	Cytoplasm	other
ENSMUSG00000009927	RPS25	9,32E-03	4,92E-01	-0,334	ribosomal protein S25	Cytoplasm	other
ENSMUSG00000030045	MRPL19	9,35E-03	4,92E-01	-0,477	mitochondrial ribosomal protein L19	Cytoplasm	other
ENSMUSG00000064360	MT-ND3	9,35E-03	4,92E-01	-0,586	NADH dehydrogenase, subunit 3 (complex I)	Cytoplasm	enzyme
ENSMUSG00000028234	RPS20	9,43E-03	4,92E-01	-0,561	ribosomal protein S20	Cytoplasm	other
ENSMUSG00000001642	AKR1B1	9,53E-03	4,92E-01	-0,277	aldo-keto reductase family 1 member B	Cytoplasm	enzyme
ENSMUSG00000033940	BRK1	9,59E-03	4,92E-01	-0,367	BRICK1 subunit of SCAR/WAVE actin nucleating complex	Cytoplasm	other
ENSMUSG00000020190	MKNK2	9,64E-03	4,92E-01	-0,302	MAPK interacting serine/threonine kinase 2	Cytoplasm	kinase
ENSMUSG00000069744	PSMB3	9,79E-03	4,95E-01	-0,394	proteasome subunit beta 3	Cytoplasm	peptidase
ENSMUSG00000046314	STXBP6	1,01E-02	4,96E-01	0,398	syntaxin binding protein 6	Cytoplasm	other
ENSMUSG00000026276	SEPTIN2	1,03E-02	4,97E-01	0,335	septin 2	Cytoplasm	enzyme
ENSMUSG00000037089	SLC35B2	1,04E-02	5,01E-01	-0,367	solute carrier family 35 member B2	Cytoplasm	transporter
ENSMUSG00000032908	SGPP2	1,05E-02	5,01E-01	-0,466	sphingosine-1-phosphate phosphatase 2	Cytoplasm	phosphatase
ENSMUSG00000035697	ARHGAP45	1,05E-02	5,01E-01	0,386	Rho GTPase activating protein 45	Cytoplasm	transporter
ENSMUSG00000018924	ALOX15	1,07E-02	1,00E+00	1,405	arachidonate 15-lipoxygenase	Cytoplasm	enzyme
ENSMUSG00000025651	UQCRC1	1,07E-02	5,03E-01	-0,28	ubiquinol-cytochrome c reductase core protein 1	Cytoplasm	enzyme
ENSMUSG00000023259	SLC26A6	1,09E-02	5,04E-01	-0,488	solute carrier family 26 member 6	Cytoplasm	transporter
ENSMUSG00000008540	MGST1	1,10E-02	5,04E-01	-0,287	microsomal glutathione S-transferase 1	Cytoplasm	enzyme
ENSMUSG00000026825	DNM1	1,11E-02	5,04E-01	0,541	dynamin 1	Cytoplasm	enzyme
ENSMUSG00000020386	SAR1B	1,11E-02	5,04E-01	-0,338	secretion associated Ras related GTPase 1B	Cytoplasm	enzyme
ENSMUSG00000003355	FKBP11	1,14E-02	5,04E-01	-0,588	FKBP prolyl isomerase 11	Cytoplasm	enzyme
ENSMUSG00000052142	RASAL3	1,14E-02	5,04E-01	0,836	RAS protein activator like 3	Cytoplasm	other
ENSMUSG00000028367	TXN	1,14E-02	5,04E-01	-0,328	thioredoxin	Cytoplasm	enzyme
ENSMUSG00000029254	STAP1	1,15E-02	5,04E-01	1,059	signal transducing adaptor family member 1	Cytoplasm	other
ENSMUSG00000022516	NUDT16L1	1,16E-02	5,04E-01	-0,331	nudix hydrolase 16 like 1	Cytoplasm	other
ENSMUSG00000032330	COX7A2	1,18E-02	5,04E-01	-0,354	cytochrome c oxidase subunit 7A2	Cytoplasm	enzyme
ENSMUSG00000028710	ATPAF1	1,20E-02	5,08E-01	-0,331	ATP synthase mitochondrial F1 complex assembly factor 1	Cytoplasm	other
ENSMUSG00000059534	UQCR10	1,22E-02	5,12E-01	-0,375	ubiquinol-cytochrome c reductase, complex III subunit X	Cytoplasm	enzyme
ENSMUSG00000015750	APH1A	1,22E-02	5,12E-01	-0,297	aph-1 homolog A, gamma-secretase subunit	Cytoplasm	peptidase
ENSMUSG00000029632	NDUFA4	1,26E-02	5,19E-01	-0,377	NDUFA4 mitochondrial complex associated	Cytoplasm	enzyme
ENSMUSG00000048755	MCAT	1,27E-02	5,22E-01	-0,457	malonyl-CoA-acyl carrier protein transacylase	Cytoplasm	enzyme
ENSMUSG00000024608	RPS14	1,30E-02	5,22E-01	-0,654	ribosomal protein S14	Cytoplasm	translation regulator
ENSMUSG00000034875	NUDT19	1,32E-02	5,22E-01	-0,397	nudix hydrolase 19	Cytoplasm	enzyme
ENSMUSG00000033671	CEP350	1,32E-02	5,22E-01	0,276	centrosomal protein 350	Cytoplasm	other
ENSMUSG00000024782	AK3	1,32E-02	5,22E-01	-0,267	adenylate kinase 3	Cytoplasm	kinase
ENSMUSG00000001052	SEC24B	1,34E-02	5,22E-01	0,305	SEC24 homolog B, COPII coat complex component	Cytoplasm	transporter

ENSMUSG00000015806	QDPR	1,35E-02	5,22E-01	-0,388	quinoid dihydropteridine reductase	Cytoplasm	enzyme
ENSMUSG00000059447	HADHB	1,40E-02	5,28E-01	-0,292	hydroxyacyl-CoA dehydrogenase trifunctional multienzyme complex subunit beta	Cytoplasm	enzyme
ENSMUSG00000022370	MRPL13	1,44E-02	5,34E-01	-0,265	mitochondrial ribosomal protein L13	Cytoplasm	other
ENSMUSG00000031255	SYTL4	1,45E-02	5,34E-01	0,545	synaptotagmin like 4	Cytoplasm	transporter
ENSMUSG00000032114	SLC37A4	1,45E-02	5,34E-01	-0,423	solute carrier family 37 member 4	Cytoplasm	transporter
ENSMUSG00000042426	DHX29	1,49E-02	5,40E-01	0,362	DExH-box helicase 29	Cytoplasm	enzyme
ENSMUSG00000024099	NDUFV2	1,52E-02	5,48E-01	-0,288	NADH:ubiquinone oxidoreductase core subunit V2	Cytoplasm	enzyme
ENSMUSG00000040952	RPS19	1,53E-02	5,48E-01	-0,509	ribosomal protein S19	Cytoplasm	other
ENSMUSG00000028779	PEF1	1,64E-02	5,59E-01	-0,36	penta-EF-hand domain containing 1	Cytoplasm	other
ENSMUSG00000002379	NDUFA11	1,67E-02	5,61E-01	-0,351	NADH:ubiquinone oxidoreductase subunit A11	Cytoplasm	enzyme
ENSMUSG00000030409	DMPK	1,67E-02	5,61E-01	0,3	DM1 protein kinase	Cytoplasm	kinase
ENSMUSG00000055493	EPM2A	1,68E-02	5,61E-01	-0,356	EPM2A glucan phosphatase, laforin	Cytoplasm	phosphatase
ENSMUSG00000056054	S100A8	1,70E-02	5,62E-01	1,207	S100 calcium binding protein A8	Cytoplasm	other
ENSMUSG00000074754	Smim26	1,70E-02	5,62E-01	-0,71	small integral membrane protein 26	Cytoplasm	other
ENSMUSG00000021951	EEF1AKMT1	1,70E-02	5,62E-01	-0,37	EEF1A lysine methyltransferase 1	Cytoplasm	enzyme
ENSMUSG00000021794	GLUD1	1,73E-02	5,64E-01	-0,363	glutamate dehydrogenase 1	Cytoplasm	enzyme
ENSMUSG00000078490	CFAP74	1,75E-02	5,64E-01	0,601	cilia and flagella associated protein 74	Cytoplasm	other
ENSMUSG00000031641	CBR4	1,75E-02	5,64E-01	-0,368	carbonyl reductase 4	Cytoplasm	enzyme
ENSMUSG00000014453	BLK	1,77E-02	5,64E-01	1,035	BLK proto-oncogene, Src family tyrosine kinase	Cytoplasm	kinase
ENSMUSG00000022403	ST13	1,77E-02	5,64E-01	0,444	ST13 Hsp70 interacting protein	Cytoplasm	other
ENSMUSG00000004266	PTPN6	1,78E-02	5,64E-01	0,557	protein tyrosine phosphatase non-receptor type 6	Cytoplasm	phosphatase
ENSMUSG00000039981	ZC3H12D	1,79E-02	5,64E-01	1,184	zinc finger CCCH-type containing 12D	Cytoplasm	other
ENSMUSG00000079641	RPL39	1,80E-02	5,64E-01	-0,43	ribosomal protein L39	Cytoplasm	other
ENSMUSG00000079557	MARCHF2	1,80E-02	5,64E-01	-0,274	membrane associated ring-CH-type finger 2	Cytoplasm	enzyme
ENSMUSG00000041736	TSPO	1,81E-02	5,64E-01	-0,453	translocator protein	Cytoplasm	transmembrane receptor
ENSMUSG00000063953	AMD1	1,82E-02	5,64E-01	-0,572	adenosylmethionine decarboxylase 1	Cytoplasm	enzyme
ENSMUSG00000021996	ESD	1,83E-02	5,64E-01	-0,328	esterase D	Cytoplasm	enzyme
ENSMUSG00000027579	SRMS	1,86E-02	5,64E-01	-0,386	src-related kinase lacking C-terminal regulatory tyrosine and N-terminal myristylation sites	Cytoplasm	kinase
ENSMUSG00000021594	SRD5A1	1,87E-02	5,64E-01	-0,484	steroid 5 alpha-reductase 1	Cytoplasm	enzyme
ENSMUSG00000028393	ALAD	1,88E-02	5,65E-01	-0,768	aminolevulinate dehydratase	Cytoplasm	enzyme
ENSMUSG00000041881	NDUFA7	1,90E-02	5,67E-01	-0,418	NADH:ubiquinone oxidoreductase subunit A7	Cytoplasm	enzyme
ENSMUSG00000027890	GSTM4	1,91E-02	5,67E-01	-0,481	glutathione S-transferase mu 4	Cytoplasm	enzyme
ENSMUSG00000068874	SELENBP1	1,94E-02	5,73E-01	-0,51	selenium binding protein 1	Cytoplasm	other
ENSMUSG00000016252	Atp5e	1,99E-02	5,78E-01	-0,643	ATP synthase, H+ transporting, mitochondrial F1 complex, epsilon subunit	Cytoplasm	transporter
ENSMUSG00000049109	THEMIS	2,01E-02	5,82E-01	1,032	thymocyte selection associated	Cytoplasm	other
ENSMUSG00000035413	TMEM98	2,01E-02	5,82E-01	-0,291	transmembrane protein 98	Cytoplasm	other
ENSMUSG00000071866	PPIA	2,01E-02	5,82E-01	-0,292	peptidylprolyl isomerase A	Cytoplasm	enzyme
ENSMUSG00000031165	WAS	2,03E-02	5,82E-01	0,604	WASP actin nucleation promoting factor	Cytoplasm	other
ENSMUSG00000040687	MADD	2,03E-02	5,82E-01	0,298	MAP kinase activating death domain	Cytoplasm	other
ENSMUSG00000087260	LAMTOR5	2,04E-02	5,83E-01	-0,365	late endosomal/lysosomal adaptor, MAPK and MTOR activator 5	Cytoplasm	other
ENSMUSG00000093674	RPL41	2,04E-02	5,83E-01	-0,382	ribosomal protein L41	Cytoplasm	other
ENSMUSG00000039163	CMC1	2,09E-02	5,89E-01	-0,376	C-X9-C motif containing 1	Cytoplasm	other
ENSMUSG00000025224	GBF1	2,14E-02	5,98E-01	0,313	golgi brefeldin A resistant guanine nucleotide exchange factor 1	Cytoplasm	other
ENSMUSG00000055652	KLHL25	2,15E-02	6,00E-01	-0,378	kelch like family member 25	Cytoplasm	other
ENSMUSG00000028996	RBP7	2,22E-02	6,03E-01	0,87	retinol binding protein 7	Cytoplasm	other
ENSMUSG00000098274	RPL24	2,22E-02	6,03E-01	-0,536	ribosomal protein L24	Cytoplasm	other
ENSMUSG00000082035	RPL17	2,23E-02	6,03E-01	-0,438	ribosomal protein L17	Cytoplasm	other
ENSMUSG00000005779	PSMB4	2,27E-02	6,03E-01	-0,39	proteasome subunit beta 4	Cytoplasm	peptidase
ENSMUSG00000032254	KIF23	2,29E-02	6,03E-01	0,791	kinesin family member 23	Cytoplasm	other
ENSMUSG00000030872	GGA2	2,29E-02	6,03E-01	-0,341	golgi associated, gamma adaptin ear containing, ARF binding protein 2	Cytoplasm	transporter
ENSMUSG00000030811	FBXL19	2,30E-02	6,03E-01	0,292	F-box and leucine rich repeat protein 19	Cytoplasm	enzyme
ENSMUSG00000051343	RAB11FIP5	2,34E-02	6,03E-01	-0,354	RAB11 family interacting protein 5	Cytoplasm	other
ENSMUSG00000060126	TPT1	2,36E-02	6,03E-01	-0,278	tumor protein, translationally-controlled 1	Cytoplasm	other
ENSMUSG00000000320	ALOX12	2,37E-02	6,03E-01	0,698	arachidonate 12-lipoxygenase, 12S type	Cytoplasm	enzyme
ENSMUSG00000026317	CLN8	2,37E-02	6,03E-01	-0,401	CLN8 transmembrane ER and ERGIC protein	Cytoplasm	other
ENSMUSG00000030220	ARHGDIB	2,39E-02	6,03E-01	0,372	Rho GDP dissociation inhibitor beta	Cytoplasm	enzyme
ENSMUSG00000000171	SDHD	2,39E-02	6,03E-01	-0,296	succinate dehydrogenase complex subunit D	Cytoplasm	enzyme
ENSMUSG00000032064	DIXDC1	2,41E-02	6,03E-01	-0,462	DIX domain containing 1	Cytoplasm	other
ENSMUSG00000003233	DVL3	2,41E-02	6,03E-01	0,274	dishevelled segment polarity protein 3	Cytoplasm	other
ENSMUSG00000030483	CYP2B6	2,41E-02	6,03E-01	-0,406	cytochrome P450 family 2 subfamily B member 6	Cytoplasm	enzyme
ENSMUSG00000026411	TMEM9	2,48E-02	6,05E-01	-0,294	transmembrane protein 9	Cytoplasm	other
ENSMUSG00000014294	NDUFA2	2,51E-02	6,05E-01	-0,335	NADH:ubiquinone oxidoreductase subunit A2	Cytoplasm	enzyme
ENSMUSG00000024997	PRDX3	2,52E-02	6,05E-01	-0,322	peroxiredoxin 3	Cytoplasm	enzyme
ENSMUSG00000008682	RPL10	2,53E-02	6,05E-01	-0,391	ribosomal protein L10	Cytoplasm	translation regulator
ENSMUSG00000006057	ATP5MC1	2,55E-02	6,05E-01	-0,399	ATP synthase membrane subunit c locus 1	Cytoplasm	transporter
ENSMUSG00000027843	PTPN22	2,58E-02	6,05E-01	0,741	protein tyrosine phosphatase non-receptor type 22	Cytoplasm	phosphatase
ENSMUSG00000071547	NT5DC2	2,59E-02	6,05E-01	-0,31	5'-nucleotidase domain containing 2	Cytoplasm	other
ENSMUSG00000028572	HOOK1	2,60E-02	6,05E-01	0,474	hook microtubule tethering protein 1	Cytoplasm	other
ENSMUSG00000027959	SASS6	2,61E-02	6,05E-01	0,438	SAS-6 centriolar assembly protein	Cytoplasm	other
ENSMUSG00000002083	BBC3	2,62E-02	6,05E-01	0,492	BCL2 binding component 3	Cytoplasm	other
ENSMUSG00000052889	PRKCB	2,70E-02	6,08E-01	0,696	protein kinase C beta	Cytoplasm	kinase
ENSMUSG00000024862	KLC2	2,71E-02	6,08E-01	0,326	kinesin light chain 2	Cytoplasm	other
ENSMUSG00000007891	CTSD	2,76E-02	6,15E-01	-0,32	cathepsin D	Cytoplasm	peptidase
ENSMUSG00000035242	OAZ1	2,78E-02	6,18E-01	-0,346	ornithine decarboxylase antizyme 1	Cytoplasm	enzyme
ENSMUSG00000057322	RPL38	2,81E-02	6,20E-01	-0,326	ribosomal protein L38	Cytoplasm	other
ENSMUSG00000041161	OTUD3	2,82E-02	6,20E-01	0,39	OTU deubiquitinase 3	Cytoplasm	peptidase
ENSMUSG00000028256	ODF2L	2,84E-02	6,20E-01	0,27	outer dense fiber of sperm tails 2 like	Cytoplasm	other
ENSMUSG00000038268	OVCA2	2,84E-02	6,20E-01	-0,288	OVCA2 serine hydrolase domain containing	Cytoplasm	other
ENSMUSG00000005161	PRDX2	2,85E-02	6,20E-01	-0,292	peroxiredoxin 2	Cytoplasm	enzyme
ENSMUSG00000079426	ARPC4	2,87E-02	6,20E-01	-0,345	actin related protein 2/3 complex subunit 4	Cytoplasm	other
ENSMUSG00000029804	HERC3	2,88E-02	6,20E-01	0,408	HECT and RLD domain containing E3 ubiquitin protein ligase 3	Cytoplasm	enzyme
ENSMUSG00000030498	GAS2	2,89E-02	6,20E-01	-0,58	growth arrest specific 2	Cytoplasm	other
ENSMUSG00000030263	LRMP	2,89E-02	6,20E-01	0,706	lymphoid restricted membrane protein	Cytoplasm	other
ENSMUSG00000029073	CPTP	2,93E-02	6,26E-01	-0,314	ceramide-1-phosphate transfer protein	Cytoplasm	transporter
ENSMUSG00000002804	NUDT14	2,96E-02	6,28E-01	-0,309	nudix hydrolase 14	Cytoplasm	phosphatase
ENSMUSG00000019761	Krt10	3,00E-02	6,31E-01	-0,602	keratin 10	Cytoplasm	other
ENSMUSG00000006526	STIMATE-MUSTN1	3,01E-02	6,33E-01	-0,277	STIMATE-MUSTN1 readthrough	Cytoplasm	other
ENSMUSG00000033728	LRRC14	3,08E-02	6,36E-01	-0,304	leucine rich repeat containing 14	Cytoplasm	other
ENSMUSG00000024875	YIF1A	3,11E-02	6,39E-01	-0,27	Yip1 interacting factor homolog A, membrane trafficking protein	Cytoplasm	other
ENSMUSG00000028527	AK4	3,12E-02	6,39E-01	0,638	adenylate kinase 4	Cytoplasm	kinase
ENSMUSG00000031320	RPS4Y1	3,12E-02	6,39E-01	-0,321	ribosomal protein S4 Y-linked 1	Cytoplasm	other
ENSMUSG00000070880	GAD1	3,14E-02	1,00E+00	1,743	glutamate decarboxylase 1	Cytoplasm	enzyme
ENSMUSG00000018585	ATOX1	3,16E-02	6,41E-01	-0,42	antioxidant 1 copper chaperone	Cytoplasm	transporter
ENSMUSG00000004285	ATP6V1F	3,16E-02	6,41E-01	-0,313	ATPase H+ transporting V1 subunit F	Cytoplasm	enzyme
ENSMUSG00000008892	VDAC3	3,21E-02	6,43E-01	-0,304	voltage dependent anion channel 3	Cytoplasm	ion channel
ENSMUSG00000021290	ATP5MPL	3,24E-02	6,43E-01	-0,542	ATP synthase membrane subunit 6.8PL	Cytoplasm	other
ENSMUSG00000021040	SLIRP	3,26E-02	6,43E-01	-0,397	SRA stem-loop interacting RNA binding protein	Cytoplasm	other
ENSMUSG00000026260	NDUFA10	3,29E-02	6,43E-01	-0,311	NADH:ubiquinone oxidoreductase subunit A10	Cytoplasm	transporter
ENSMUSG00000006315	TMEM147	3,30E-02	6,43E-01	-0,269	transmembrane protein 147	Cytoplasm	other
ENSMUSG00000003814	CALR	3,30E-02	6,43E-01	0,28	calreticulin	Cytoplasm	transcription regulator
ENSMUSG00000041444	ARHGAP32	3,31E-02	6,43E-01	-0,277	Rho GTPase activating protein 32	Cytoplasm	other
ENSMUSG00000025353	ORMDL2	3,35E-02	6,43E-01	-0,329	ORMDL sphingolipid biosynthesis regulator 2	Cytoplasm	other
ENSMUSG00000113902	NDUFB1	3,35E-02	6,43E-01	-0,347	NADH:ubiquinone oxidoreductase subunit B1	Cytoplasm	enzyme
ENSMUSG00000026701	PRDX6	3,35E-02	6,43E-01	-0,464	peroxiredoxin 6	Cytoplasm	enzyme
ENSMUSG00000033793	ATP6V1H	3,38E-02	6,43E-01	-0,265	ATPase H+ transporting V1 subunit H	Cytoplasm	transporter
ENSMUSG00000058317	UBE2E2	3,39E-02	6,43E-01	-0,287	ubiquitin conjugating enzyme E2 E2	Cytoplasm	enzyme
ENSMUSG00000039497	DSE	3,40E-02	6,43E-01	-0,315	dermatan sulfate epimerase	Cytoplasm	enzyme
ENSMUSG00000022940	PIGP	3,42E-02	6,43E-01	-0,326	phosphatidylinositol glycan anchor biosynthesis class P	Cytoplasm	enzyme
ENSMUSG00000078238	Gm12854/S100a11	3,43E-02	6,43E-01	-1,269	S100 calcium binding protein A11	Cytoplasm	other
ENSMUSG00000021905	DPH3	3,43E-02	6,43E-01	-0,34	diphthamide biosynthesis 3	Cytoplasm	other

ENSMUSG00000018965	YWHAH	3,46E-02	6,46E-01	0,268	tyrosine 3-monooxygenase/tryptophan 5-monooxygenase activation protein eta	Cytoplasm	transcription regulator
ENSMUSG00000032177	PDE4A	3,49E-02	6,47E-01	0,304	phosphodiesterase 4A	Cytoplasm	enzyme
ENSMUSG00000058586	SERHL2	3,50E-02	6,47E-01	-0,362	serine hydrolase like 2	Cytoplasm	enzyme
ENSMUSG00000038784	CNOT4	3,51E-02	6,48E-01	0,265	CCR4-NOT transcription complex subunit 4	Cytoplasm	enzyme
ENSMUSG00000022519	SRL	3,52E-02	1,00E+00	0,896	sarcalumenin	Cytoplasm	other
ENSMUSG00000022400	Rbx1	3,56E-02	6,48E-01	-0,311	ring-box 1	Cytoplasm	enzyme
ENSMUSG00000019102	ALDH3A1	3,62E-02	6,53E-01	-0,777	aldehyde dehydrogenase 3 family member A1	Cytoplasm	enzyme
ENSMUSG00000062515	FABP4	3,64E-02	6,53E-01	0,507	fatty acid binding protein 4	Cytoplasm	transporter
ENSMUSG00000048007	TIMM8A	3,65E-02	6,53E-01	-0,375	translocase of inner mitochondrial membrane 8A	Cytoplasm	transporter
ENSMUSG00000035674	NDUFA3	3,66E-02	6,53E-01	-0,416	NADH:ubiquinone oxidoreductase subunit A3	Cytoplasm	enzyme
ENSMUSG00000053040	Aph1c	3,68E-02	6,56E-01	-0,527	aph1 homolog C, gamma secretase subunit	Cytoplasm	peptidase
ENSMUSG00000037860	AIM2	3,72E-02	6,57E-01	0,576	absent in melanoma 2	Cytoplasm	other
ENSMUSG00000057375	YIPF1	3,78E-02	6,57E-01	-0,302	Yip1 domain family member 1	Cytoplasm	other
ENSMUSG00000029762	AKR1B10	3,79E-02	6,57E-01	-0,404	aldo-keto reductase family 1 member B10	Cytoplasm	enzyme
ENSMUSG00000021182	CCDC88C	3,81E-02	6,58E-01	0,554	coiled-coil domain containing 88C	Cytoplasm	other
ENSMUSG00000025465	ECHS1	3,81E-02	6,58E-01	-0,352	enoyl-CoA hydratase, short chain 1	Cytoplasm	enzyme
ENSMUSG00000036781	RPS27L	3,84E-02	6,60E-01	-0,375	ribosomal protein S27 like	Cytoplasm	translation regulator
ENSMUSG00000025150	Cbr2	3,86E-02	6,62E-01	-0,427	carbonyl reductase 2	Cytoplasm	enzyme
ENSMUSG00000021947	CRYL1	3,93E-02	6,69E-01	-0,422	crystallin lambda 1	Cytoplasm	enzyme
ENSMUSG00000022671	MZT2A	3,97E-02	6,71E-01	-0,309	mitotic spindle organizing protein 2A	Cytoplasm	other
ENSMUSG00000041498	KIF14	4,02E-02	1,00E+00	1,297	kinesin family member 14	Cytoplasm	enzyme
ENSMUSG00000029449	RHOF	4,07E-02	6,73E-01	0,465	ras homolog family member F, filopodia associated	Cytoplasm	enzyme
ENSMUSG00000039264	GIMAP1-GIMAP5	4,07E-02	6,73E-01	0,607	GIMAP1-GIMAP5 readthrough	Cytoplasm	other
ENSMUSG00000012405	RPL15	4,07E-02	6,73E-01	-0,281	ribosomal protein L15	Cytoplasm	other
ENSMUSG00000020766	GALK1	4,09E-02	6,73E-01	-0,275	galactokinase 1	Cytoplasm	kinase
ENSMUSG00000026786	APBB1IP	4,11E-02	6,73E-01	0,587	amyloid beta precursor protein binding family B member 1 interacting protein	Cytoplasm	other
ENSMUSG00000031200	CMC4	4,13E-02	6,73E-01	0,324	C-X9-C motif containing 4	Cytoplasm	other
ENSMUSG00000062825	ACTG1	4,16E-02	6,73E-01	-0,316	actin gamma 1	Cytoplasm	other
ENSMUSG00000014301	PAM16	4,18E-02	6,73E-01	-0,318	presequence translocase associated motor 16	Cytoplasm	other
ENSMUSG00000059743	FDPS	4,22E-02	6,73E-01	-0,36	farnesyl diphosphate synthase	Cytoplasm	enzyme
ENSMUSG00000039670	OXLD1	4,33E-02	6,73E-01	0,539	oxidoreductase like domain containing 1	Cytoplasm	other
ENSMUSG00000026154	SDHAF4	4,61E-02	6,80E-01	-0,525	succinate dehydrogenase complex assembly factor 4	Cytoplasm	enzyme
ENSMUSG00000020120	PLEK	4,61E-02	6,80E-01	0,408	pleckstrin	Cytoplasm	other
ENSMUSG00000021906	OXNAD1	4,62E-02	6,80E-01	0,332	oxidoreductase NAD binding domain containing 1	Cytoplasm	transporter
ENSMUSG00000031974	ABCB10	4,66E-02	6,80E-01	-0,288	ATP binding cassette subfamily B member 10	Cytoplasm	transporter
ENSMUSG00000029798	HERC6	4,67E-02	6,80E-01	0,296	HECT and RLD domain containing E3 ubiquitin protein ligase family member 6	Cytoplasm	enzyme
ENSMUSG00000040964	ARHGEF10L	4,70E-02	6,80E-01	0,375	Rho guanine nucleotide exchange factor 10 like	Cytoplasm	enzyme
ENSMUSG00000054675	TMEM119	4,70E-02	6,80E-01	-0,314	transmembrane protein 119	Cytoplasm	other
ENSMUSG00000008668	RPS18	4,73E-02	6,80E-01	-0,304	ribosomal protein S18	Cytoplasm	other
ENSMUSG00000038412	HIGD1A	4,77E-02	6,80E-01	-0,445	HIG1 hypoxia inducible domain family member 1A	Cytoplasm	other
ENSMUSG00000009876	COX4I2	4,78E-02	6,80E-01	-0,375	cytochrome c oxidase subunit 4I2	Cytoplasm	enzyme
ENSMUSG00000049775	Tmsb4x (includes others)	4,79E-02	6,80E-01	-0,368	thymosin, beta 4, X chromosome	Cytoplasm	other
ENSMUSG00000025792	SLC25A10	4,80E-02	6,80E-01	-0,39	solute carrier family 25 member 10	Cytoplasm	transporter
ENSMUSG00000044986	TST	4,83E-02	6,80E-01	-0,382	thiosulfate sulfurtransferase	Cytoplasm	enzyme
ENSMUSG00000004931	APBA3	4,84E-02	6,80E-01	-0,279	amyloid beta precursor protein binding family A member 3	Cytoplasm	transporter
ENSMUSG00000057058	SKAP1	4,90E-02	6,80E-01	0,674	src kinase associated phosphoprotein 1	Cytoplasm	kinase
ENSMUSG00000032563	MRPL3	4,91E-02	6,80E-01	-0,308	mitochondrial ribosomal protein L3	Cytoplasm	other
ENSMUSG00000071014	NDUFB6	4,94E-02	6,80E-01	-0,279	NADH:ubiquinone oxidoreductase subunit B6	Cytoplasm	enzyme
ENSMUSG00000047888	TNRC6B	5,00E-02	6,80E-01	0,319	trinucleotide repeat containing adaptor 6B	Cytoplasm	other
ENSMUSG00000024053	EMILIN2	2,44E-04	1,02E-01	1,055	elastin microfibril interfacer 2	Extracellular Space	other
ENSMUSG00000006014	Prg4	3,11E-04	1,11E-01	1,197	proteoglycan 4 (megakaryocyte stimulating factor, articular superficial zone protein)	Extracellular Space	other
ENSMUSG00000027857	TSHB	6,30E-04	1,56E-01	-0,833	thyroid stimulating hormone subunit beta	Extracellular Space	other
ENSMUSG00000026987	BAZ2B	1,21E-03	2,36E-01	0,312	bromodomain adjacent to zinc finger domain 2B	Extracellular Space	other
ENSMUSG00000042379	ESM1	1,51E-03	2,64E-01	0,575	endothelial cell specific molecule 1	Extracellular Space	growth factor
ENSMUSG00000049521	CDC42EP1	2,12E-03	3,04E-01	-0,268	CDC42 effector protein 1	Extracellular Space	other
ENSMUSG00000000489	PDGFB	2,61E-03	3,19E-01	-0,399	platelet derived growth factor subunit B	Extracellular Space	growth factor
ENSMUSG00000042050	WDR60	3,06E-03	3,41E-01	0,474	WD repeat domain 60	Extracellular Space	other
ENSMUSG00000000693	LOXL3	3,33E-03	3,57E-01	-0,474	lysyl oxidase like 3	Extracellular Space	enzyme
ENSMUSG00000062929	CFL2	3,61E-03	3,62E-01	-0,342	cofilin 2	Extracellular Space	other
ENSMUSG00000041445	MMRN2	4,79E-03	4,03E-01	-0,316	multimerin 2	Extracellular Space	other
ENSMUSG00000032484	Ngp	4,98E-03	4,03E-01	2,322	neutrophilic granule protein	Extracellular Space	other
ENSMUSG00000000743	CHMP1A	5,56E-03	4,08E-01	-0,384	charged multivesicular body protein 1A	Extracellular Space	peptidase
ENSMUSG00000039109	F13A1	5,93E-03	4,23E-01	0,83	coagulation factor XIII A chain	Extracellular Space	enzyme
ENSMUSG00000029838	PTN	8,06E-03	4,75E-01	0,633	pleiotrophin	Extracellular Space	growth factor
ENSMUSG00000032554	TF	9,19E-03	4,91E-01	-0,38	transferrin	Extracellular Space	transporter
ENSMUSG00000037965	ZC3H7A	9,97E-03	4,96E-01	0,301	zinc finger CCCH-type containing 7A	Extracellular Space	other
ENSMUSG00000055632	HMCN2	1,05E-02	1,00E+00	-2,246	hemicentin 2	Extracellular Space	other
ENSMUSG00000029811	AOC1	1,08E-02	1,00E+00	-2,237	amine oxidase copper containing 1	Extracellular Space	enzyme
ENSMUSG00000030583	SIPA1L3	1,32E-02	5,22E-01	0,331	signal induced proliferation associated 1 like 3	Extracellular Space	other
ENSMUSG00000030116	MFAP5	1,41E-02	5,28E-01	-0,456	microfibril associated protein 5	Extracellular Space	other
ENSMUSG00000054169	Ceacam10	1,43E-02	1,00E+00	-1,973	carcinoembryonic antigen-related cell adhesion molecule 10	Extracellular Space	other
ENSMUSG00000004814	CCL24	1,58E-02	1,00E+00	2,105	C-C motif chemokine ligand 24	Extracellular Space	cytokine
ENSMUSG00000002204	NAPSA	1,70E-02	5,62E-01	-0,486	napsin A aspartic peptidase	Extracellular Space	peptidase
ENSMUSG00000028226	MMP16	1,77E-02	5,64E-01	0,708	matrix metallopeptidase 16	Extracellular Space	peptidase
ENSMUSG00000031137	FGF13	1,93E-02	1,00E+00	1,342	fibroblast growth factor 13	Extracellular Space	growth factor
ENSMUSG00000043613	MMP3	2,19E-02	6,03E-01	-0,81	matrix metallopeptidase 3	Extracellular Space	peptidase
ENSMUSG00000050447	LYPD6	2,24E-02	1,00E+00	1,644	LY6/PLAUR domain containing 6	Extracellular Space	other
ENSMUSG00000017754	PLTP	2,27E-02	6,03E-01	-0,267	phospholipid transfer protein	Extracellular Space	enzyme
ENSMUSG00000062345	SERPINB2	2,28E-02	1,00E+00	1,38	serpin family B member 2	Extracellular Space	other
ENSMUSG00000017723	WFDC2	2,36E-02	6,03E-01	-0,53	WAP four-disulfide core domain 2	Extracellular Space	other
ENSMUSG00000026579	F5	2,38E-02	6,03E-01	0,8	coagulation factor V	Extracellular Space	other
ENSMUSG00000021508	CXCL14	2,45E-02	6,04E-01	-0,412	C-X-C motif chemokine ligand 14	Extracellular Space	cytokine
ENSMUSG00000039307	HEXD	2,64E-02	6,06E-01	-0,339	hexosaminidase D	Extracellular Space	enzyme
ENSMUSG00000056370	SFTPB	2,67E-02	6,06E-01	-0,498	surfactant protein B	Extracellular Space	other
ENSMUSG00000029417	Cxcl9	2,72E-02	6,09E-01	1,645	chemokine (C-X-C motif) ligand 9	Extracellular Space	cytokine
ENSMUSG00000016637	IFT27	3,07E-02	6,36E-01	-0,333	intraflagellar transport 27	Extracellular Space	enzyme
ENSMUSG00000062778	CHIA	3,07E-02	6,36E-01	-0,842	chitinase acidic	Extracellular Space	enzyme
ENSMUSG00000062329	CYTL1	3,35E-02	6,43E-01	-0,38	cytokine like 1	Extracellular Space	cytokine
ENSMUSG00000029373	PF4	3,70E-02	6,57E-01	0,866	platelet factor 4	Extracellular Space	cytokine
ENSMUSG00000030800	PRSS8	3,80E-02	6,58E-01	-0,335	serine protease 8	Extracellular Space	peptidase
ENSMUSG00000030111	A2M	4,22E-02	1,00E+00	-1,892	alpha-2-macroglobulin	Extracellular Space	transporter
ENSMUSG00000022097	SFTPC	4,42E-02	6,77E-01	-0,512	surfactant protein C	Extracellular Space	other
ENSMUSG00000029005	DRAXIN	4,55E-02	6,80E-01	0,997	dorsal inhibitory axon guidance protein	Extracellular Space	other
ENSMUSG00000018593	SPARC	4,60E-02	6,80E-01	-0,331	secreted protein acidic and cysteine rich	Extracellular Space	other
ENSMUSG00000007987	IFT22	4,75E-02	6,80E-01	-0,283	intraflagellar transport 22	Extracellular Space	other
ENSMUSG00000021765	FST	4,83E-02	6,80E-01	-0,49	follistatin	Extracellular Space	other
**ENSMUSG00000060036**	**RPL3**	**1,20E-06**	**2,15E-03**	**-0,555**	**ribosomal protein L3**	**Nucleus**	**other**
**ENSMUSG00000048251**	**BCL11B**	**1,14E-05**	**1,18E-02**	**1,403**	**BAF chromatin remodeling complex subunit BCL11B**	**Nucleus**	**transcription regulator**
**ENSMUSG00000031229**	**ATRX**	**1,32E-05**	**1,27E-02**	**0,448**	**ATRX chromatin remodeler**	**Nucleus**	**transcription regulator**
**ENSMUSG00000032621**	**SREK1**	**4,82E-05**	**3,64E-02**	**0,423**	**splicing regulatory glutamic acid and lysine rich protein 1**	**Nucleus**	**other**
ENSMUSG00000020863	LUC7L3	8,73E-05	5,71E-02	0,439	LUC7 like 3 pre-mRNA splicing factor	Nucleus	other
ENSMUSG00000030680	PAGR1a	8,76E-05	5,71E-02	-2,391	PAXIP1 associated glutamate rich protein 1	Nucleus	other
ENSMUSG00000019982	MYB	1,60E-04	8,86E-02	2,144	MYB proto-oncogene, transcription factor	Nucleus	transcription regulator
ENSMUSG00000022148	FYB	1,97E-04	9,83E-02	1,015	FYN binding protein 1	Nucleus	other
ENSMUSG00000042772	SMG7	2,37E-04	1,02E-01	0,297	SMG7 nonsense mediated mRNA decay factor	Nucleus	other
ENSMUSG00000023927	SATB1	2,78E-04	1,03E-01	0,982	SATB homeobox 1	Nucleus	transcription regulator
ENSMUSG00000055436	SRSF11	2,79E-04	1,03E-01	0,324	serine and arginine rich splicing factor 11	Nucleus	other
ENSMUSG00000063273	NAA15	3,75E-04	1,25E-01	0,327	N(alpha)-acetyltransferase 15, NatA auxiliary subunit	Nucleus	transcription regulator
ENSMUSG00000004698	HDAC9	5,69E-04	1,48E-01	0,409	histone deacetylase 9	Nucleus	transcription regulator
ENSMUSG00000023150	IVNS1ABP	6,10E-04	1,56E-01	0,343	influenza virus NS1A binding protein	Nucleus	other

ENSMUSG00000057110	CNTRL	6,89E-04	1,62E-01	0,339	centriolin	Nucleus	transcription regulator
ENSMUSG00000071637	CEBPD	7,25E-04	1,65E-01	0,738	CCAAT enhancer binding protein delta	Nucleus	transcription regulator
ENSMUSG00000025261	HUWE1	1,10E-03	2,28E-01	0,44	HECT, UBA and WWE domain containing E3 ubiquitin protein ligase 1	Nucleus	transcription regulator
ENSMUSG00000035649	ZCCHC7	1,24E-03	2,36E-01	0,463	zinc finger CCHC-type containing 7	Nucleus	other
ENSMUSG00000033933	VHL	1,32E-03	2,45E-01	-0,416	von Hippel-Lindau tumor suppressor	Nucleus	transcription regulator
ENSMUSG00000003778	BRD8	1,59E-03	2,64E-01	0,367	bromodomain containing 8	Nucleus	transcription regulator
ENSMUSG00000071054	SAFB	1,76E-03	2,77E-01	0,325	scaffold attachment factor B	Nucleus	other
ENSMUSG00000039218	Srrm2	2,16E-03	3,04E-01	0,282	serine/arginine repetitive matrix 2	Nucleus	other
ENSMUSG00000028248	PNISR	2,20E-03	3,04E-01	0,314	PNN interacting serine and arginine rich protein	Nucleus	other
ENSMUSG00000048154	KMT2D	2,30E-03	3,12E-01	0,408	lysine methyltransferase 2D	Nucleus	transcription regulator
ENSMUSG00000037608	BCLAF1	2,47E-03	3,17E-01	0,329	BCL2 associated transcription factor 1	Nucleus	transcription regulator
ENSMUSG00000065990	AURKAIP1	2,54E-03	3,17E-01	-0,309	aurora kinase A interacting protein 1	Nucleus	enzyme
ENSMUSG00000038116	PHF20	2,79E-03	3,25E-01	0,293	PHD finger protein 20	Nucleus	transcription regulator
ENSMUSG00000024325	RING1	2,85E-03	3,25E-01	0,323	ring finger protein 1	Nucleus	transcription regulator
ENSMUSG00000050605	Zfp61	2,95E-03	3,33E-01	0,378	zinc finger protein 61	Nucleus	peptidase
ENSMUSG00000038538	UBN2	3,06E-03	3,41E-01	0,283	ubinuclein 2	Nucleus	other
ENSMUSG00000022185	ACIN1	3,20E-03	3,48E-01	0,309	apoptotic chromatin condensation inducer 1	Nucleus	enzyme
ENSMUSG00000035021	BAZ1A	3,32E-03	3,57E-01	0,318	bromodomain adjacent to zinc finger domain 1A	Nucleus	other
ENSMUSG00000032053	POU2AF1	3,36E-03	3,57E-01	1,051	POU class 2 homeobox associating factor 1	Nucleus	transcription regulator
ENSMUSG00000044807	ZNF354C	3,54E-03	3,62E-01	0,356	zinc finger protein 354C	Nucleus	transcription regulator
ENSMUSG00000029594	RBM19	3,80E-03	3,76E-01	0,361	RNA binding motif protein 19	Nucleus	other
ENSMUSG00000036893	EHMT1	3,84E-03	3,78E-01	0,439	euchromatic histone lysine methyltransferase 1	Nucleus	transcription regulator
ENSMUSG00000047810	CCDC88B	3,90E-03	3,80E-01	1,264	coiled-coil domain containing 88B	Nucleus	enzyme
ENSMUSG00000004980	HNRNPA2B1	4,05E-03	3,82E-01	0,298	heterogeneous nuclear ribonucleoprotein A2/B1	Nucleus	other
ENSMUSG00000029415	SDAD1	4,16E-03	3,90E-01	0,377	SDA1 domain containing 1	Nucleus	other
ENSMUSG00000070283	NDUFAF3	4,36E-03	4,01E-01	-0,4	NADH:ubiquinone oxidoreductase complex assembly factor 3	Nucleus	other
ENSMUSG00000024498	TCERG1	4,62E-03	4,03E-01	0,336	transcription elongation regulator 1	Nucleus	transcription regulator
ENSMUSG00000021413	PRPF4B	4,86E-03	4,03E-01	0,326	pre-mRNA processing factor 4B	Nucleus	kinase
ENSMUSG00000021963	SAP18	4,87E-03	4,03E-01	-0,273	Sin3A associated protein 18	Nucleus	transcription regulator
ENSMUSG00000038628	POLR3K	4,98E-03	4,03E-01	-0,292	RNA polymerase III subunit K	Nucleus	transcription regulator
ENSMUSG00000017499	CDC6	5,03E-03	4,03E-01	-0,972	cell division cycle 6	Nucleus	other
ENSMUSG00000028483	SNAPC3	5,19E-03	4,03E-01	0,324	small nuclear RNA activating complex polypeptide 3	Nucleus	other
ENSMUSG00000032212	SLTM	5,22E-03	4,03E-01	0,294	SAFB like transcription modulator	Nucleus	other
ENSMUSG00000040865	INO80D	5,51E-03	4,08E-01	0,332	INO80 complex subunit D	Nucleus	other
ENSMUSG00000065037	RN7SK	5,58E-03	4,08E-01	-0,84	RNA component of 7SK nuclear ribonucleoprotein	Nucleus	other
ENSMUSG00000029439	SFSWAP	5,63E-03	4,08E-01	0,27	splicing factor SWAP	Nucleus	other
ENSMUSG00000020074	CCAR1	6,34E-03	4,30E-01	0,319	cell division cycle and apoptosis regulator 1	Nucleus	transcription regulator
ENSMUSG00000033166	DIS3	6,51E-03	4,30E-01	0,294	DIS3 homolog, exosome endoribonuclease and 3'-5' exoribonuclease	Nucleus	enzyme
ENSMUSG00000056919	CEP162	6,74E-03	4,34E-01	0,368	centrosomal protein 162	Nucleus	other
ENSMUSG00000037572	WDHD1	6,95E-03	4,36E-01	0,569	WD repeat and HMG-box DNA binding protein 1	Nucleus	transcription regulator
ENSMUSG00000055531	CPSF6	7,21E-03	4,50E-01	0,264	cleavage and polyadenylation specific factor 6	Nucleus	other
ENSMUSG00000020721	HELZ	7,43E-03	4,58E-01	0,286	helicase with zinc finger	Nucleus	enzyme
ENSMUSG00000028820	SFPQ	7,57E-03	4,62E-01	0,358	splicing factor proline and glutamine rich	Nucleus	transcription regulator
ENSMUSG00000070544	TOP1	7,58E-03	4,62E-01	0,349	DNA topoisomerase I	Nucleus	enzyme
ENSMUSG00000003847	NFAT5	8,12E-03	4,75E-01	0,322	nuclear factor of activated T cells 5	Nucleus	transcription regulator
ENSMUSG00000032727	MIER3	8,36E-03	4,80E-01	0,315	MIER family member 3	Nucleus	transcription regulator
ENSMUSG00000040721	ZFHX2	9,73E-03	4,93E-01	0,464	zinc finger homeobox 2	Nucleus	transcription regulator
ENSMUSG00000090258	CHURC1	1,01E-02	4,96E-01	-0,531	churchill domain containing 1	Nucleus	transcription regulator
ENSMUSG00000035696	RNF38	1,06E-02	5,03E-01	0,339	ring finger protein 38	Nucleus	enzyme
ENSMUSG00000000708	KAT2B	1,06E-02	5,03E-01	0,276	lysine acetyltransferase 2B	Nucleus	transcription regulator
ENSMUSG00000000339	RTCA	1,08E-02	5,03E-01	-0,275	RNA 3'-terminal phosphate cyclase	Nucleus	enzyme
ENSMUSG00000030180	KDM5A	1,08E-02	5,03E-01	0,288	lysine demethylase 5A	Nucleus	transcription regulator
ENSMUSG00000043962	THRAP3	1,08E-02	5,03E-01	0,28	thyroid hormone receptor associated protein 3	Nucleus	transcription regulator
ENSMUSG00000029120	PPP2R2C	1,11E-02	5,04E-01	0,545	protein phosphatase 2 regulatory subunit Bgamma	Nucleus	phosphatase
ENSMUSG00000039384	DUSP10	1,11E-02	5,04E-01	0,402	dual specificity phosphatase 10	Nucleus	phosphatase
ENSMUSG00000064141	ZFP69	1,13E-02	5,04E-01	0,388	ZFP69 zinc finger protein	Nucleus	transcription regulator
ENSMUSG00000017550	ATAD5	1,15E-02	5,04E-01	0,461	ATPase family AAA domain containing 5	Nucleus	enzyme
ENSMUSG00000032253	PHIP	1,15E-02	5,04E-01	0,265	pleckstrin homology domain interacting protein	Nucleus	other
ENSMUSG00000014039	PRDM15	1,16E-02	5,04E-01	0,447	PR/SET domain 15	Nucleus	transcription regulator
ENSMUSG00000063511	SNRNP70	1,18E-02	5,04E-01	0,335	small nuclear ribonucleoprotein U1 subunit 70	Nucleus	other
ENSMUSG00000082029	H3C14	1,25E-02	5,17E-01	-0,568	H3 clustered histone 14	Nucleus	other
ENSMUSG00000079487	MED12	1,32E-02	5,22E-01	0,31	mediator complex subunit 12	Nucleus	transcription regulator
ENSMUSG00000097814	Panct2	1,35E-02	5,22E-01	0,895	pluripotency-associated noncoding transcript 2	Nucleus	other
ENSMUSG00000022983	SCAF4	1,38E-02	5,28E-01	0,272	SR-related CTD associated factor 4	Nucleus	other
ENSMUSG00000022178	AJUBA	1,40E-02	5,28E-01	-0,307	ajuba LIM protein	Nucleus	transcription regulator
ENSMUSG00000020719	DDX5	1,42E-02	5,29E-01	0,366	DEAD-box helicase 5	Nucleus	enzyme
ENSMUSG00000026275	PPP1R7	1,47E-02	5,37E-01	-0,379	protein phosphatase 1 regulatory subunit 7	Nucleus	phosphatase
ENSMUSG00000061360	PHF5A	1,53E-02	5,48E-01	-0,338	PHD finger protein 5A	Nucleus	transcription regulator
ENSMUSG00000018168	IKZF3	1,54E-02	5,48E-01	0,818	IKAROS family zinc finger 3	Nucleus	transcription regulator
ENSMUSG00000030256	Bhlhe41	1,57E-02	5,54E-01	-0,892	basic helix-loop-helix family, member e41	Nucleus	transcription regulator
ENSMUSG00000040734	PPP1R13L	1,59E-02	5,54E-01	0,58	protein phosphatase 1 regulatory subunit 13 like	Nucleus	transcription regulator
ENSMUSG00000042524	SUN2	1,61E-02	5,58E-01	-0,279	Sad1 and UNC84 domain containing 2	Nucleus	other
ENSMUSG00000041297	CDK13	1,62E-02	5,58E-01	0,337	cyclin dependent kinase 13	Nucleus	kinase
ENSMUSG00000039183	NUBP2	1,65E-02	5,60E-01	-0,311	nucleotide binding protein 2	Nucleus	other
ENSMUSG00000018697	AATF	1,68E-02	5,61E-01	0,295	apoptosis antagonizing transcription factor	Nucleus	transcription regulator
ENSMUSG00000027510	RBM38	1,79E-02	5,64E-01	0,408	RNA binding motif protein 38	Nucleus	other
ENSMUSG00000009076	ZMAT5	1,80E-02	5,64E-01	-0,308	zinc finger matrin-type 5	Nucleus	other
ENSMUSG00000082585	Gm15387/Hmgb1-ps7	1,86E-02	5,64E-01	0,451	high-mobility group high mobility group box 1, pseudogene 7	Nucleus	other
ENSMUSG00000052707	TNRC6A	1,86E-02	5,64E-01	0,268	trinucleotide repeat containing adaptor 6A	Nucleus	other
ENSMUSG00000041126	H2AZ2	1,87E-02	5,64E-01	-0,27	H2A.Z variant histone 2	Nucleus	other
ENSMUSG00000053007	CREB5	1,88E-02	5,65E-01	0,557	cAMP responsive element binding protein 5	Nucleus	transcription regulator
ENSMUSG00000028809	Srrm1	1,90E-02	5,67E-01	0,305	serine/arginine repetitive matrix 1	Nucleus	other
ENSMUSG00000042496	PRDM10	1,95E-02	5,74E-01	0,284	PR/SET domain 10	Nucleus	transcription regulator
ENSMUSG00000063488	ZKSCAN7	2,02E-02	5,82E-01	0,439	zinc finger with KRAB and SCAN domains 7	Nucleus	transcription regulator
ENSMUSG00000018654	IKZF1	2,02E-02	5,82E-01	0,701	IKAROS family zinc finger 1	Nucleus	transcription regulator
ENSMUSG00000003437	PAF1	2,19E-02	6,03E-01	0,443	PAF1 homolog, Paf1/RNA polymerase II complex component	Nucleus	other
ENSMUSG00000016409	NKAP	2,21E-02	6,03E-01	0,275	NFKB activating protein	Nucleus	transcription regulator
ENSMUSG00000015804	MED28	2,24E-02	6,03E-01	-0,338	mediator complex subunit 28	Nucleus	other
ENSMUSG00000030750	NSMCE1	2,26E-02	6,03E-01	-0,277	NSE1 homolog, SMC5-SMC6 complex component	Nucleus	transporter
ENSMUSG00000028654	MYCL	2,30E-02	1,00E+00	-0,976	MYCL proto-oncogene, bHLH transcription factor	Nucleus	transcription regulator
ENSMUSG00000029290	ZNF326	2,32E-02	6,03E-01	0,279	zinc finger protein 326	Nucleus	transcription regulator
ENSMUSG00000059995	ATXN7L3	2,35E-02	6,03E-01	-0,3	ataxin 7 like 3	Nucleus	transcription regulator
ENSMUSG00000024045	AKAP8	2,35E-02	6,03E-01	0,27	A-kinase anchoring protein 8	Nucleus	other
ENSMUSG00000039852	RERE	2,41E-02	6,03E-01	0,341	arginine-glutamic acid dipeptide repeats	Nucleus	transcription regulator
ENSMUSG00000078887	ZNF442	2,43E-02	6,04E-01	-0,575	zinc finger protein 442	Nucleus	transcription regulator
ENSMUSG00000052798	NUP107	2,45E-02	6,04E-01	0,263	nucleoporin 107	Nucleus	other
ENSMUSG00000039910	CITED2	2,45E-02	6,04E-01	0,364	Cbp/p300 interacting transactivator with Glu/Asp rich carboxy-terminal domain 2	Nucleus	transcription regulator
ENSMUSG00000036959	BCORL1	2,47E-02	6,05E-01	0,345	BCL6 corepressor like 1	Nucleus	transcription regulator
ENSMUSG00000049470	AFF4	2,47E-02	6,05E-01	0,417	AF4/FMR2 family member 4	Nucleus	transcription regulator
ENSMUSG00000022941	RIPPLY3	2,52E-02	6,05E-01	-0,331	ripply transcriptional repressor 3	Nucleus	other
ENSMUSG00000036931	NFKBID	2,55E-02	6,05E-01	0,466	NFKB inhibitor delta	Nucleus	transcription regulator
ENSMUSG00000020069	HNRNPH3	2,57E-02	6,05E-01	0,27	heterogeneous nuclear ribonucleoprotein H3	Nucleus	other
ENSMUSG00000019738	POLR2I	2,59E-02	6,05E-01	-0,293	RNA polymerase II subunit I	Nucleus	transcription regulator
ENSMUSG00000031821	GINS2	2,62E-02	6,05E-01	-0,758	GINS complex subunit 2	Nucleus	other
ENSMUSG00000032316	CLK3	2,65E-02	6,06E-01	0,386	CDC like kinase 3	Nucleus	kinase
ENSMUSG00000073490	IFI16	2,70E-02	6,08E-01	0,674	interferon gamma inducible protein 16	Nucleus	transcription regulator
ENSMUSG00000033020	POLR2F	2,82E-02	6,20E-01	-0,333	RNA polymerase II subunit F	Nucleus	enzyme
ENSMUSG00000036281	SNAPC4	2,83E-02	6,20E-01	0,277	small nuclear RNA activating complex polypeptide 4	Nucleus	transcription regulator
ENSMUSG00000026094	STK17B	2,83E-02	6,20E-01	0,423	serine/threonine kinase 17b	Nucleus	kinase
ENSMUSG00000067870	Rpl31-ps14 (includes others)	2,83E-02	6,20E-01	-0,371	ribosomal protein L31, pseudogene 8	Nucleus	other

ENSMUSG00000051495	IRF2BP2	2,90E-02	6,21E-01	0,434	interferon regulatory factor 2 binding protein 2	Nucleus	transcription regulator
ENSMUSG00000029603	DTX1	2,94E-02	6,27E-01	0,428	deltex E3 ubiquitin ligase 1	Nucleus	transcription regulator
ENSMUSG00000024002	Brd4	2,96E-02	6,28E-01	0,344	bromodomain containing 4	Nucleus	kinase
ENSMUSG00000042557	SIN3A	2,97E-02	6,29E-01	0,313	SIN3 transcription regulator family member A	Nucleus	transcription regulator
ENSMUSG00000026565	POU2F1	3,02E-02	6,33E-01	0,37	POU class 2 homeobox 1	Nucleus	transcription regulator
ENSMUSG00000037007	ZNF3	3,03E-02	6,34E-01	0,285	zinc finger protein 3	Nucleus	transcription regulator
ENSMUSG00000019564	ARID3A	3,05E-02	6,36E-01	0,484	AT-rich interaction domain 3A	Nucleus	transcription regulator
ENSMUSG00000032077	BUD13	3,14E-02	6,40E-01	0,354	BUD13 homolog	Nucleus	other
ENSMUSG00000036572	UPF3B	3,21E-02	6,43E-01	0,369	UPF3B regulator of nonsense mediated mRNA decay	Nucleus	transporter
ENSMUSG00000037197	RBM17	3,31E-02	6,43E-01	0,266	RNA binding motif protein 17	Nucleus	other
ENSMUSG00000049396	GEMIN4	3,35E-02	6,43E-01	-0,609	gem nuclear organelle associated protein 4	Nucleus	other
ENSMUSG00000005378	BUD23	3,36E-02	6,43E-01	0,276	BUD23 rRNA methyltransferase and ribosome maturation factor	Nucleus	enzyme
ENSMUSG00000035236	SCAI	3,41E-02	6,43E-01	0,338	suppressor of cancer cell invasion	Nucleus	transcription regulator
ENSMUSG00000109324	PRMT1	3,42E-02	6,43E-01	-0,415	protein arginine methyltransferase 1	Nucleus	enzyme
ENSMUSG00000020032	NUAK1	3,44E-02	6,44E-01	-0,266	NUAK family kinase 1	Nucleus	kinase
ENSMUSG00000039197	ADK	3,49E-02	6,47E-01	0,287	adenosine kinase	Nucleus	kinase
ENSMUSG00000078773	RAD54B	3,51E-02	1,00E+00	0,823	RAD54 homolog B	Nucleus	enzyme
ENSMUSG00000069910	SPDL1	3,52E-02	6,48E-01	0,808	spindle apparatus coiled-coil protein 1	Nucleus	other
ENSMUSG00000057278	Snrpg	3,52E-02	6,48E-01	-0,465	small nuclear ribonucleoprotein polypeptide G	Nucleus	other
ENSMUSG00000021217	TSHZ3	3,53E-02	6,48E-01	0,287	teashirt zinc finger homeobox 3	Nucleus	transcription regulator
ENSMUSG00000005045	CHD5	3,55E-02	1,00E+00	1,562	chromodomain helicase DNA binding protein 5	Nucleus	enzyme
ENSMUSG00000031422	MORF4L2	3,55E-02	6,48E-01	0,415	mortality factor 4 like 2	Nucleus	other
ENSMUSG00000104896	Rnu3b4	3,72E-02	1,00E+00	-1,656	U3B small nuclear RNA 4	Nucleus	other
ENSMUSG00000070034	SP110	3,72E-02	6,57E-01	0,348	SP110 nuclear body protein	Nucleus	transcription regulator
ENSMUSG00000092192	DNAAF4	3,73E-02	1,00E+00	-1,209	dynein axonemal assembly factor 4	Nucleus	other
ENSMUSG00000002028	KMT2A	3,76E-02	6,57E-01	0,292	lysine methyltransferase 2A	Nucleus	transcription regulator
ENSMUSG00000073616	COPS9	3,78E-02	6,57E-01	-0,588	COP9 signalosome subunit 9	Nucleus	other
ENSMUSG00000019947	ARID5B	3,81E-02	6,58E-01	0,329	AT-rich interaction domain 5B	Nucleus	transcription regulator
ENSMUSG00000029430	RAN	3,82E-02	6,58E-01	0,429	RAN, member RAS oncogene family	Nucleus	enzyme
ENSMUSG00000029250	POLR2B	3,87E-02	6,62E-01	0,326	RNA polymerase II subunit B	Nucleus	enzyme
ENSMUSG00000030966	TRIM21	3,96E-02	6,71E-01	0,285	tripartite motif containing 21	Nucleus	enzyme
ENSMUSG00000061028	Clasrp	3,97E-02	6,71E-01	0,308	CLK4-associating serine/arginine rich protein	Nucleus	other
ENSMUSG00000021453	GADD45G	4,05E-02	6,73E-01	0,336	growth arrest and DNA damage inducible gamma	Nucleus	other
ENSMUSG00000007836	HNRNPA0	4,05E-02	6,73E-01	0,307	heterogeneous nuclear ribonucleoprotein A0	Nucleus	other
ENSMUSG00000034083	CCDC174	4,07E-02	6,73E-01	0,311	coiled-coil domain containing 174	Nucleus	other
ENSMUSG00000054237	FRA10AC1	4,10E-02	6,73E-01	0,287	FRA10A associated CGG repeat 1	Nucleus	other
ENSMUSG00000089694	Nat8f3 (includes others)	4,11E-02	6,73E-01	-0,771	N-acetyltransferase 8 (GCN5-related) family member 3	Nucleus	enzyme
ENSMUSG00000002625	AKAP8L	4,14E-02	6,73E-01	0,267	A-kinase anchoring protein 8 like	Nucleus	other
ENSMUSG00000018501	NCOR1	4,14E-02	6,73E-01	0,331	nuclear receptor corepressor 1	Nucleus	transcription regulator
ENSMUSG00000069305	H4C13	4,17E-02	6,73E-01	1,036	H4 clustered histone 13	Nucleus	other
ENSMUSG00000006932	CTNNB1	4,23E-02	6,73E-01	-0,313	catenin beta 1	Nucleus	transcription regulator
ENSMUSG00000079553	KIFC1	4,26E-02	6,73E-01	0,843	kinesin family member C1	Nucleus	enzyme
ENSMUSG00000014850	MSH3	4,29E-02	6,73E-01	0,355	mutS homolog 3	Nucleus	enzyme
ENSMUSG00000051550	ZNF579	4,31E-02	6,73E-01	0,314	zinc finger protein 579	Nucleus	transcription regulator
ENSMUSG00000043323	FBRSL1	4,33E-02	6,73E-01	0,337	fibrosin like 1	Nucleus	other
ENSMUSG00000000581	C1D	4,34E-02	6,73E-01	-0,397	C1D nuclear receptor corepressor	Nucleus	transcription regulator
ENSMUSG00000094066	FAM205A	4,36E-02	1,00E+00	2,078	family with sequence similarity 205 member A	Nucleus	other
ENSMUSG00000022831	HCLS1	4,43E-02	6,77E-01	0,521	hematopoietic cell-specific Lyn substrate 1	Nucleus	other
ENSMUSG00000054717	HMGB2	4,45E-02	6,77E-01	0,367	high mobility group box 2	Nucleus	transcription regulator
ENSMUSG00000084838	Gm10241/Gm13690	4,46E-02	6,77E-01	0,419	SMT3 suppressor of mif two 3 homolog 2 pseudogene	Nucleus	other
ENSMUSG00000052837	JUNB	4,47E-02	6,77E-01	0,484	JunB proto-oncogene, AP-1 transcription factor subunit	Nucleus	transcription regulator
ENSMUSG00000016526	DYRK3	4,50E-02	1,00E+00	-1,311	dual specificity tyrosine phosphorylation regulated kinase 3	Nucleus	kinase
ENSMUSG00000037262	KIN	4,60E-02	6,80E-01	0,29	Kin17 DNA and RNA binding protein	Nucleus	other
ENSMUSG00000050244	HEATR1	4,61E-02	6,80E-01	-0,34	HEAT repeat containing 1	Nucleus	other
ENSMUSG00000025427	RNF165	4,63E-02	6,80E-01	0,822	ring finger protein 165	Nucleus	enzyme
ENSMUSG00000023940	SGO1	4,64E-02	1,00E+00	1,05	shugoshin 1	Nucleus	other
ENSMUSG00000021597	SLF1	4,64E-02	6,80E-01	0,27	SMC5-SMC6 complex localization factor 1	Nucleus	transcription regulator
ENSMUSG00000048118	ARID4A	4,64E-02	6,80E-01	0,308	AT-rich interaction domain 4A	Nucleus	transcription regulator
ENSMUSG00000030699	TBX6	4,70E-02	6,80E-01	0,459	T-box transcription factor 6	Nucleus	transcription regulator
ENSMUSG00000040943	TET2	4,74E-02	6,80E-01	0,283	tet methylcytosine dioxygenase 2	Nucleus	enzyme
ENSMUSG00000010362	RDM1	4,79E-02	6,80E-01	-0,294	RAD52 motif containing 1	Nucleus	other
ENSMUSG00000070808	BICRA	4,88E-02	6,80E-01	0,407	BRD4 interacting chromatin remodeling complex associated protein	Nucleus	transcription regulator
ENSMUSG00000029275	GFI1	4,90E-02	1,00E+00	1,07	growth factor independent 1 transcriptional repressor	Nucleus	transcription regulator
ENSMUSG00000059208	HNRNPM	4,93E-02	6,80E-01	0,3	heterogeneous nuclear ribonucleoprotein M	Nucleus	other
ENSMUSG00000070780	RBM47	4,94E-02	6,80E-01	0,679	RNA binding motif protein 47	Nucleus	other
**ENSMUSG00000105361**	**AY036118**	**2,97E-29**	**4,26E-25**	**-1,852**	**cDNA sequence AY036118**	**Other**	**other**
**ENSMUSG00000084349**	**Rpl3-ps1**	**9,65E-28**	**6,92E-24**	**1,047**	**ribosomal protein L3, pseudogene 1**	**Other**	**other**
**ENSMUSG00000106106**	**Rn18s-rs5**	**4,48E-10**	**1,61E-06**	**-1,648**		**Other**	**other**
ENSMUSG00000100220	Gm29331	3,02E-09	1,00E+00	-7,697		Other	other
**ENSMUSG00000080727**	**C920021L13Rik**	**2,70E-07**	**6,46E-04**	**0,482**	**RIKEN cDNA C920021L13 gene**	**Other**	**other**
**ENSMUSG00000098650**	**Gm28048**	**4,45E-07**	**9,11E-04**	**-1,062**		**Other**	**other**
**ENSMUSG00000100131**	**Gm28439**	**4,99E-07**	**1,00E+00**	**-7,802**		**Other**	**other**
**ENSMUSG00000094103**	**Fam177a**	**1,91E-06**	**3,04E-03**	**-13,246**	**family with sequence similarity 177, member A**	**Other**	**other**
**ENSMUSG00000099021**	**Rn7s1**	**3,48E-06**	**4,54E-03**	**-1,169**	**7S RNA 1**	**Other**	**other**
**ENSMUSG00000099250**	**Rn7s2**	**3,48E-06**	**4,54E-03**	**-1,169**	**7S RNA 2**	**Other**	**other**
**ENSMUSG00000072612**	**Gm10382**	**6,59E-06**	**7,88E-03**	**1,466**		**Other**	**other**
ENSMUSG00000021908	Gm6768	1,16E-04	7,25E-02	-1,718	predicted gene 6768	Other	other
ENSMUSG00000105703	Gm43305	1,60E-04	8,86E-02	0,667	predicted gene 43305	Other	other
ENSMUSG00000097461	Gm26735	1,89E-04	9,83E-02	-0,689		Other	other
ENSMUSG00000094446	Gm6344	2,39E-04	1,02E-01	-1,891	ribosomal protein L29 pseudogene	Other	other
ENSMUSG00000048271	Rbm33	3,26E-04	1,14E-01	0,345	RNA binding motif protein 33	Other	other
ENSMUSG00000026655	FAM107B	3,94E-04	1,28E-01	0,507	family with sequence similarity 107 member B	Other	other
ENSMUSG00000100514	Gm12960	4,43E-04	1,41E-01	1,184	heterogeneous nuclear ribonucleoprotein A3 pseudogene	Other	other
ENSMUSG00000108912	E230020D15Rik	5,40E-04	1,48E-01	0,422		Other	other
ENSMUSG00000072501	PHF20L1	5,65E-04	1,48E-01	0,297	PHD finger protein 20 like 1	Other	other
ENSMUSG00000018821	AVPI1	6,20E-04	1,56E-01	-0,402	arginine vasopressin induced 1	Other	other
ENSMUSG00000112743	RP23-160A1.5	6,60E-04	1,58E-01	1,791		Other	other
ENSMUSG00000097352	C920009B18Rik	7,85E-04	1,76E-01	5,35	RIKEN cDNA C920009B18 gene	Other	other
ENSMUSG00000099931	Gm29358	8,97E-04	1,00E+00	3,621		Other	other
ENSMUSG00000102961	Gm19918	9,88E-04	1,00E+00	-3,654		Other	other
ENSMUSG00000062017	Abca14	1,17E-03	2,36E-01	-1,137	ATP-binding cassette, sub-family A (ABC1), member 14	Other	other
ENSMUSG00000062382	LOC100862446	1,21E-03	2,36E-01	-0,492	ferritin light chain 1	Other	other
ENSMUSG00000102411	Gm36936	1,25E-03	2,36E-01	0,615		Other	other
ENSMUSG00000105677	Gm43328	1,33E-03	2,45E-01	0,515		Other	other
ENSMUSG00000087396	4933407K13Rik	1,56E-03	2,64E-01	0,401	RIKEN cDNA 4933407K13 gene	Other	other
ENSMUSG00000032526	SS18L2	1,60E-03	2,64E-01	-0,551	SS18 like 2	Other	other
ENSMUSG00000068262	Gm5879	1,68E-03	1,00E+00	-3,115	60S ribosomal protein L3 related	Other	other
ENSMUSG00000031145	PRICKLE3	1,86E-03	2,82E-01	0,428	prickle planar cell polarity protein 3	Other	other
ENSMUSG00000095304	Gm9780 (includes others)	1,87E-03	2,82E-01	-1,156	predicted gene 9780	Other	other
ENSMUSG00000056211	R3HDM1	2,31E-03	3,12E-01	0,311	R3H domain containing 1	Other	other
ENSMUSG00000069520	TMEM19	2,36E-03	3,14E-01	-0,363	transmembrane protein 19	Other	other
ENSMUSG00000079065	BC005561	2,43E-03	3,16E-01	0,321	cDNA sequence BC005561	Other	other
ENSMUSG00000097336	Fendrr	2,64E-03	3,19E-01	0,292	Foxf1 adjacent non-coding developmental regulatory RNA	Other	other
ENSMUSG00000037318	TRAF3IP3	2,80E-03	3,25E-01	0,922	TRAF3 interacting protein 3	Other	other
ENSMUSG00000106099	Gm42664	3,20E-03	3,48E-01	0,46		Other	other
ENSMUSG00000110148	5830408C22Rik	3,42E-03	3,61E-01	0,771	RIKEN cDNA 5830408C22 gene	Other	other
ENSMUSG00000068240	Gm11808	3,56E-03	3,62E-01	-0,427	ubiquitin A-52 residue ribosomal protein fusion product 1 pseudogene	Other	other
ENSMUSG00000089809	RASGEF1B	3,58E-03	3,62E-01	0,466	RasGEF domain family member 1B	Other	other
ENSMUSG00000073492	Gm10521	3,72E-03	1,00E+00	2,751		Other	other
ENSMUSG00000107962	Gm43980	3,98E-03	3,81E-01	0,483		Other	other

ENSMUSG00000094737	Gm17045	4,25E-03	1,00E+00	-3,303	predicted gene 17045	Other	other
ENSMUSG00000069972	Rps13-ps2	4,42E-03	4,01E-01	-1,621	ribosomal protein S13, pseudogene 2	Other	other
ENSMUSG00000041143	TMCO4	4,51E-03	4,03E-01	-0,398	transmembrane and coiled-coil domains 4	Other	other
ENSMUSG00000036377	C530008M17Rik	4,64E-03	4,03E-01	0,925	RIKEN cDNA C530008M17 gene	Other	other
ENSMUSG00000110841	Gpx4-ps2	4,65E-03	4,03E-01	-0,379	glutathione peroxidase 4, pseudogene 2	Other	other
ENSMUSG00000038022	MINDY4	4,77E-03	4,03E-01	-0,475	MINDY lysine 48 deubiquitinase 4	Other	other
ENSMUSG00000063019	MANBAL	5,08E-03	4,03E-01	-0,308	mannosidase beta like	Other	other
ENSMUSG00000037300	TTC13	5,08E-03	4,03E-01	0,272	tetratricopeptide repeat domain 13	Other	other
ENSMUSG00000004945	TMEM242	5,15E-03	4,03E-01	-0,337	transmembrane protein 242	Other	other
ENSMUSG00000117333	Gm16386	5,31E-03	4,04E-01	0,783	zinc finger protein 946 pseudogene	Other	other
ENSMUSG00000029028	LRRC47	5,43E-03	4,06E-01	0,345	leucine rich repeat containing 47	Other	other
ENSMUSG00000034437	Gm9761	5,60E-03	4,08E-01	-0,873	splicing factor, arginine/serine-rich pseudogene	Other	other
ENSMUSG00000071531	GPRIN2	5,81E-03	1,00E+00	2,734	G protein regulated inducer of neurite outgrowth 2	Other	other
ENSMUSG00000079020	SLC45A4	5,83E-03	4,18E-01	0,496	solute carrier family 45 member 4	Other	transporter
ENSMUSG00000095865	Gm13237	5,94E-03	1,00E+00	1,918	predicted gene 13237	Other	other
ENSMUSG00000025086	TRUB1	6,02E-03	4,25E-01	0,424	TruB pseudouridine synthase family member 1	Other	enzyme
ENSMUSG00000108218	Olfr1372-ps1	6,08E-03	4,26E-01	0,434	olfactory receptor 1372, pseudogene 1	Other	other
ENSMUSG00000090625	Gm20721	6,48E-03	4,30E-01	-0,512		Other	other
ENSMUSG00000090733	Rps27/Rps27rt	6,68E-03	4,34E-01	-0,509	ribosomal protein S27	Other	other
ENSMUSG00000101162	Gm26728	6,87E-03	4,34E-01	-1,586	predicted gene, 26728	Other	other
ENSMUSG00000087412	Gm15501	6,88E-03	4,34E-01	-0,715	predicted pseudogene 15501	Other	other
ENSMUSG00000097764	Gm26635	7,12E-03	1,00E+00	2,351		Other	other
ENSMUSG00000031647	MFAP3L	7,63E-03	4,62E-01	0,429	microfibril associated protein 3 like	Other	other
ENSMUSG00000020691	METTL2A	7,67E-03	4,62E-01	0,329	methyltransferase like 2A	Other	enzyme
ENSMUSG00000024816	FRMD8	7,76E-03	4,65E-01	-0,324	FERM domain containing 8	Other	other
ENSMUSG00000113529	AC159649.3	7,99E-03	4,74E-01	0,842		Other	other
ENSMUSG00000076498	Trbc2	8,36E-03	4,80E-01	1,14	T cell receptor beta, constant 2	Other	other
ENSMUSG00000083337	Gm11539	8,41E-03	1,00E+00	-1,803	ribosomal protein L18 pseudogene	Other	other
ENSMUSG00000108614	2610306O10Rik	8,49E-03	4,82E-01	0,857		Other	other
ENSMUSG00000114003	Gm9616	8,60E-03	4,82E-01	-0,386	ribosomal protein S4, X-linked pseudogene	Other	other
ENSMUSG00000042790	RNF214	8,66E-03	4,83E-01	0,328	ring finger protein 214	Other	other
ENSMUSG00000027677	TTC14	8,77E-03	4,87E-01	0,3	tetratricopeptide repeat domain 14	Other	other
ENSMUSG00000075408	Smim41	8,80E-03	4,87E-01	-0,45	small integral membrane protein 41	Other	other
ENSMUSG00000022591	Gm9747	8,92E-03	4,88E-01	-0,716		Other	other
ENSMUSG00000071748	Gm14698	9,04E-03	4,88E-01	-0,541	serine/threonine/tyrosine interaction protein pseudogene	Other	other
ENSMUSG00000084319	Tpt1-ps3	9,22E-03	4,91E-01	-0,547	tumor protein, translationally-controlled, pseudogene 3	Other	other
ENSMUSG00000043801	Oaz1-ps	9,63E-03	4,92E-01	-0,399	ornithine decarboxylase antizyme 1, pseudogene	Other	other
ENSMUSG00000108037	Gm44597	1,03E-02	4,97E-01	0,395		Other	other
ENSMUSG00000024483	ANKHD1/ANKHD1-EIF4EBP3	1,03E-02	4,98E-01	0,387	ankyrin repeat and KH domain containing 1	Other	other
ENSMUSG00000108589	Gm45225	1,13E-02	5,04E-01	-0,757		Other	other
ENSMUSG00000003531	Dgcr6	1,13E-02	5,04E-01	-0,305	DiGeorge syndrome critical region gene 6	Other	other
ENSMUSG00000076490	Trbc1	1,14E-02	5,04E-01	1,212	T cell receptor beta, constant region 1	Other	other
ENSMUSG00000020133	C19orf25	1,15E-02	5,04E-01	-0,282	chromosome 19 open reading frame 25	Other	other
ENSMUSG00000052419	2610001J05Rik	1,17E-02	5,04E-01	-0,409	RIKEN cDNA 2610001J05 gene	Other	other
ENSMUSG00000087259	2610035D17Rik	1,23E-02	5,13E-01	-0,484	RIKEN cDNA 2610035D17 gene	Other	other
ENSMUSG00000079652	FAM71F2	1,24E-02	5,17E-01	0,363	family with sequence similarity 71 member F2	Other	other
ENSMUSG00000060989	Gm11847	1,25E-02	5,17E-01	0,751	heterogeneous nuclear ribonucleoprotein A3 pseudogene	Other	other
ENSMUSG00000039221	Rpl22l1	1,25E-02	5,17E-01	-0,78	ribosomal protein L22 like 1	Other	other
ENSMUSG00000033852	Gm28042	1,29E-02	5,22E-01	-0,466	predicted gene, 28042	Other	other
ENSMUSG00000098793	Gm27286	1,31E-02	1,00E+00	2,486		Other	other
ENSMUSG00000024677	Ms4a6b	1,32E-02	5,22E-01	0,668	membrane-spanning 4-domains, subfamily A, member 6B	Other	other
ENSMUSG00000028643	SVBP	1,33E-02	5,22E-01	-0,377	small vasohibin binding protein	Other	other
ENSMUSG00000029422	Rsrc2	1,33E-02	5,22E-01	0,295	arginine/serine-rich coiled-coil 2	Other	other
ENSMUSG00000040459	ARGLU1	1,33E-02	5,22E-01	0,321	arginine and glutamate rich 1	Other	other
ENSMUSG00000032850	RNFT2	1,34E-02	5,22E-01	0,926	ring finger protein, transmembrane 2	Other	other
ENSMUSG00000028857	TMEM222	1,36E-02	5,26E-01	-0,278	transmembrane protein 222	Other	other
ENSMUSG00000027881	PRPF38B	1,39E-02	5,28E-01	0,459	pre-mRNA processing factor 38B	Other	other
ENSMUSG00000116908	AC154232.2	1,41E-02	5,28E-01	0,395		Other	other
ENSMUSG00000110197	Gm45444	1,44E-02	1,00E+00	2,035		Other	other
ENSMUSG00000097055	Gm4419	1,44E-02	1,00E+00	1,199	predicted gene 4419	Other	other
ENSMUSG00000074800	Gm4149	1,48E-02	5,40E-01	-0,582	ribosomal protein L37a pseudogene	Other	other
ENSMUSG00000045435	TMEM60	1,53E-02	5,48E-01	-0,489	transmembrane protein 60	Other	other
ENSMUSG00000073437	D330041H03Rik	1,54E-02	5,48E-01	0,593	RIKEN cDNA D330041H03 gene	Other	other
ENSMUSG00000094989	Rpl9-ps4	1,59E-02	5,54E-01	-1,196	ribosomal protein L9, pseudogene 4	Other	other
ENSMUSG00000066724	Gm10175/Gm10231	1,60E-02	5,56E-01	-0,505	ATP synthase, H+ transporting, mitochondrial F0 complex, subunit C2 (subunit 9) pseudogene	Other	other
ENSMUSG00000014592	CAMTA1	1,61E-02	5,58E-01	-0,567	calmodulin binding transcription activator 1	Other	other
ENSMUSG00000076937	Iglc2	1,63E-02	5,58E-01	0,832	immunoglobulin lambda constant 2	Other	other
ENSMUSG00000097891	Gm3650	1,66E-02	5,61E-01	0,95	predicted gene 3650	Other	other
ENSMUSG00000022554	HGH1	1,71E-02	5,62E-01	-0,485	HGH1 homolog	Other	other
ENSMUSG00000067038	Rps12-ps3	1,76E-02	5,64E-01	-0,344	ribosomal protein S12, pseudogene 3	Other	other
ENSMUSG00000062110	SCFD2	1,79E-02	5,64E-01	-0,422	sec1 family domain containing 2	Other	transporter
ENSMUSG00000103509	Gm38372	1,83E-02	5,64E-01	0,524		Other	other
ENSMUSG00000030823	9130019O22Rik/Zfp747	1,83E-02	5,64E-01	-0,362	RIKEN cDNA 9130019O22 gene	Other	other
ENSMUSG00000051238	SWSAP1	1,87E-02	5,64E-01	-0,329	SWIM-type zinc finger 7 associated protein 1	Other	enzyme
ENSMUSG00000044600	SMIM7	1,87E-02	5,64E-01	-0,512	small integral membrane protein 7	Other	other
ENSMUSG00000115902	D730005E14Rik	1,92E-02	5,69E-01	-0,759	RIKEN cDNA D730005E14 gene	Other	other
ENSMUSG00000060096	Amd-ps3	1,94E-02	5,73E-01	-0,972	S-adenosylmethionine decarboxylase, pseudogene 3	Other	other
ENSMUSG00000090141	CXorf65	1,95E-02	1,00E+00	0,957	chromosome X open reading frame 65	Other	other
ENSMUSG00000117748	Chtf8	1,96E-02	5,74E-01	-0,947		Other	other
ENSMUSG00000093803	Ppp2r3d	1,96E-02	5,75E-01	0,369	protein phosphatase 2 (formerly 2A), regulatory subunit B'', delta	Other	phosphatase
ENSMUSG00000087367	Gm15491	2,00E-02	1,00E+00	-1,86		Other	other
ENSMUSG00000103821	D430013B06Rik	2,03E-02	1,00E+00	0,861		Other	other
ENSMUSG00000058838	Rps27a-ps2	2,07E-02	5,86E-01	-0,618	ribosomal protein S27A, pseudogene 2	Other	other
ENSMUSG00000057359	Gm17494	2,09E-02	5,89E-01	-0,423		Other	other
ENSMUSG00000096942	Rps19-ps6	2,10E-02	5,89E-01	-0,835	ribosomal protein S19, pseudogene 6	Other	other
ENSMUSG00000111082	Gm30934	2,15E-02	1,00E+00	1,302	predicted gene, 30934	Other	other
ENSMUSG00000110057	Gm2225	2,17E-02	6,03E-01	-0,715	predicted gene 2225	Other	other
ENSMUSG00000036864	Proser3	2,17E-02	6,03E-01	0,451	proline and serine rich 3	Other	other
ENSMUSG00000087067	Gm11532	2,20E-02	1,00E+00	-2,387	predicted gene 11532	Other	other
ENSMUSG00000041707	TMEM273	2,20E-02	1,00E+00	-1,728	transmembrane protein 273	Other	other
ENSMUSG00000058443	Rpl10-ps3	2,20E-02	6,03E-01	-0,453	ribosomal protein L10, pseudogene 3	Other	other
ENSMUSG00000089945	Pakap	2,22E-02	6,03E-01	0,542		Other	other
ENSMUSG00000039461	TCTA	2,23E-02	6,03E-01	-0,398	T cell leukemia translocation altered	Other	other
ENSMUSG00000026123	PLEKHB2	2,23E-02	6,03E-01	-0,277	pleckstrin homology domain containing B2	Other	other
ENSMUSG00000109829	Gm45605	2,25E-02	6,03E-01	0,59		Other	other
ENSMUSG00000054708	ANKRD24	2,28E-02	6,03E-01	0,427	ankyrin repeat domain 24	Other	other
ENSMUSG00000102982	Gm38319	2,31E-02	1,00E+00	1,258		Other	other
ENSMUSG00000085342	Gm12254	2,32E-02	6,03E-01	-0,661		Other	other
ENSMUSG00000036395	GLB1L2	2,33E-02	6,03E-01	-0,289	galactosidase beta 1 like 2	Other	other
ENSMUSG00000110397	Gm45540	2,34E-02	6,03E-01	0,516		Other	other
ENSMUSG00000097123	Gm6297	2,35E-02	6,03E-01	0,889	predicted gene 6297	Other	other
ENSMUSG00000113061	Rps18-ps5	2,36E-02	6,03E-01	-1,055	ribosomal protein S18, pseudogene 5	Other	other
ENSMUSG00000024535	SNX24	2,36E-02	6,03E-01	-0,5	sorting nexin 24	Other	transporter
ENSMUSG00000061167	Rpl15-ps3	2,38E-02	6,03E-01	-0,401	ribosomal protein L15, pseudogene 3	Other	other
ENSMUSG00000108959	Gm44697	2,39E-02	1,00E+00	2,487		Other	other
ENSMUSG00000089940	Gm4117	2,39E-02	1,00E+00	1,537	predicted gene 4117	Other	other
ENSMUSG00000105055	Gm43079	2,40E-02	6,03E-01	-0,747		Other	other
ENSMUSG00000100954	Gm10138	2,41E-02	6,03E-01	1,125	predicted gene 10138	Other	other
ENSMUSG00000112596	RP23-345J24.2	2,43E-02	6,04E-01	0,489		Other	other
ENSMUSG00000059355	WDR83OS	2,44E-02	6,04E-01	-0,4	WD repeat domain 83 opposite strand	Other	other

ENSMUSG00000086807	Platr21	2,60E-02	6,05E-01	0,746	pluripotency associated transcript 21	Other	other
ENSMUSG00000099923	1700105P06Rik	2,63E-02	1,00E+00	1,81	RIKEN cDNA 1700105P06 gene	Other	other
ENSMUSG00000030822	PRR14	2,64E-02	6,06E-01	0,276	proline rich 14	Other	other
ENSMUSG00000109998	Gm45437	2,66E-02	6,06E-01	0,534		Other	other
ENSMUSG00000080935	Got2-ps1	2,67E-02	6,06E-01	-0,784	glutamatic-oxaloacetic transaminase 2, mitochondrial, pseudogene 1	Other	other
ENSMUSG00000092329	Gm20388	2,70E-02	6,08E-01	-1,078		Other	other
ENSMUSG00000057605	Gm6136/Gm6807	2,74E-02	1,00E+00	-1,956	ribosomal protein L6 pseudogene	Other	other
ENSMUSG00000073910	MOB3B	2,76E-02	6,16E-01	0,409	MOB kinase activator 3B	Other	other
ENSMUSG00000111027	Gm2981	2,86E-02	6,20E-01	-0,84	apoptosis inhibitor 5 pseudogene	Other	other
ENSMUSG00000089736	TGFBR3L	2,91E-02	6,23E-01	0,621	transforming growth factor beta receptor 3 like	Other	other
ENSMUSG00000087266	Gm15991	2,92E-02	1,00E+00	1,539	predicted gene 15991	Other	other
ENSMUSG00000091367	Gm17711	2,92E-02	1,00E+00	-2,142		Other	other
ENSMUSG00000084416	Rpl10a-ps1	2,96E-02	6,28E-01	-0,565	ribosomal protein L10A, pseudogene 1	Other	other
ENSMUSG00000106933	Gm43621	2,98E-02	6,30E-01	0,805		Other	other
ENSMUSG00000035875	AI182371	3,01E-02	6,33E-01	0,639	expressed sequence AI182371	Other	other
ENSMUSG00000091905	Gm6395	3,04E-02	1,00E+00	-0,893	DnaJ (Hsp40) homolog, subfamily B, member 6 pseudogene	Other	other
ENSMUSG00000037731	THEMIS2	3,06E-02	6,36E-01	0,704	thymocyte selection associated family member 2	Other	other
ENSMUSG00000044854	1700056E22Rik	3,07E-02	1,00E+00	1,718	RIKEN cDNA 1700056E22 gene	Other	other
ENSMUSG00000030876	METTL9	3,11E-02	6,39E-01	-0,346	methyltransferase like 9	Other	other
ENSMUSG00000104605	Gm42922	3,12E-02	1,00E+00	0,985		Other	other
ENSMUSG00000107549	Gm43961	3,16E-02	1,00E+00	1,375		Other	other
ENSMUSG00000029461	FAM168A	3,17E-02	6,41E-01	0,286	family with sequence similarity 168 member A	Other	other
ENSMUSG00000086316	Nbdy	3,19E-02	6,42E-01	-0,389	negative regulator of P-body association	Other	other
ENSMUSG00000050592	FAM78A	3,21E-02	6,43E-01	0,3	family with sequence similarity 78 member A	Other	other
ENSMUSG00000031886	Ces2e	3,29E-02	6,43E-01	-0,52	carboxylesterase 2E	Other	other
ENSMUSG00000095649	Gm8979	3,35E-02	6,43E-01	0,772	very large inducible GTPase 1 pseudogene	Other	other
ENSMUSG00000109539	Gm44667	3,35E-02	6,43E-01	0,463		Other	other
ENSMUSG00000035530	Eif1	3,37E-02	6,43E-01	-0,538	eukaryotic translation initiation factor 1	Other	other
ENSMUSG00000094530	Gm21399	3,38E-02	1,00E+00	-0,989	peroxiredoxin pseudogene 2	Other	other
ENSMUSG00000104546	Gm43858	3,39E-02	6,43E-01	0,372		Other	other
ENSMUSG00000074071	Fam169b	3,40E-02	6,43E-01	0,798	family with sequence similarity 169, member B	Other	other
ENSMUSG00000026594	RALGPS2	3,40E-02	6,43E-01	0,376	Ral GEF with PH domain and SH3 binding motif 2	Other	other
ENSMUSG00000111605	RP24-325N9.4	3,41E-02	6,43E-01	0,725		Other	other
ENSMUSG00000034006	SLC66A2	3,41E-02	6,43E-01	-0,276	solute carrier family 66 member 2	Other	other
ENSMUSG00000038578	SUSD1	3,47E-02	6,46E-01	0,793	sushi domain containing 1	Other	other
ENSMUSG00000091537	TMA7	3,52E-02	6,48E-01	-0,367	translation machinery associated 7 homolog	Other	other
ENSMUSG00000033767	TMEM131L	3,55E-02	6,48E-01	0,292	transmembrane 131 like	Other	other
ENSMUSG00000045679	SLC66A3	3,55E-02	6,48E-01	-0,277	solute carrier family 66 member 3	Other	other
ENSMUSG00000038524	FCHSD1	3,56E-02	6,48E-01	0,542	FCH and double SH3 domains 1	Other	other
ENSMUSG00000115148	AC107711.1	3,59E-02	6,51E-01	0,744		Other	other
ENSMUSG00000072676	Tmem254a (includes others)	3,59E-02	6,51E-01	-0,657	transmembrane protein 254a	Other	other
ENSMUSG00000051736	FAM229B	3,61E-02	1,00E+00	-0,752	family with sequence similarity 229 member B	Other	other
ENSMUSG00000078915	Hsp25-ps1	3,65E-02	6,53E-01	0,555	heat shock protein 25, pseudogene 1	Other	other
ENSMUSG00000105655	Gm42659	3,69E-02	6,56E-01	0,568		Other	other
ENSMUSG00000027942	C1orf43	3,70E-02	6,57E-01	-0,283	chromosome 1 open reading frame 43	Other	other
ENSMUSG00000098943	Rnu3b1	3,72E-02	1,00E+00	-1,656	U3B small nuclear RNA 1	Other	other
ENSMUSG00000105115	Rnu3b2	3,72E-02	1,00E+00	-1,656	U3B small nuclear RNA 2	Other	other
ENSMUSG00000099291	Rnu3b3	3,72E-02	1,00E+00	-1,656	U3B small nuclear RNA 3	Other	other
ENSMUSG00000086154	Gm16196	3,74E-02	1,00E+00	-2,153	predicted gene 16196	Other	other
ENSMUSG00000097479	Gm26582	3,74E-02	6,57E-01	0,513		Other	other
ENSMUSG00000093954	Gm16867	3,75E-02	6,57E-01	0,309	predicted gene, 16867	Other	other
ENSMUSG00000030469	Zfp719	3,75E-02	6,57E-01	-0,333	zinc finger protein 719	Other	other
ENSMUSG00000081281	Gm6274	3,80E-02	1,00E+00	-1,225	ribosomal protein L7 pseudogene	Other	other
ENSMUSG00000043168	4930426D05Rik	3,83E-02	1,00E+00	1,603	RIKEN cDNA 4930426D05 gene	Other	other
ENSMUSG00000049881	2810025M15Rik	3,85E-02	6,62E-01	-0,462	RIKEN cDNA 2810025M15 gene	Other	other
ENSMUSG00000047150	1700001C19Rik	3,86E-02	1,00E+00	-1,128	RIKEN cDNA 1700001C19 gene	Other	other
ENSMUSG00000036114	RPP25L	3,86E-02	6,62E-01	-0,335	ribonuclease P/MRP subunit p25 like	Other	other
ENSMUSG00000085042	Abhd11os	3,88E-02	1,00E+00	0,867	abhydrolase domain containing 11, opposite strand	Other	other
ENSMUSG00000055188	Rbm3os	4,00E-02	1,00E+00	1,047	RNA binding motif protein 3, opposite strand	Other	other
ENSMUSG00000081087	Rps15a-ps7	4,06E-02	6,73E-01	-0,751	ribosomal protein S15A, pseudogene 7	Other	other
ENSMUSG00000092595	Gm20427	4,08E-02	6,73E-01	0,726		Other	other
ENSMUSG00000108443	Gm44510	4,09E-02	6,73E-01	-0,867		Other	other
ENSMUSG00000082536	Gm13456	4,11E-02	6,73E-01	-0,364	eukaryotic translation elongation factor 1 alpha 1 pseudogene	Other	other
ENSMUSG00000097559	D430018E03Rik	4,14E-02	1,00E+00	1,224		Other	other
ENSMUSG00000090963	Gm17655	4,16E-02	6,73E-01	-0,727	zinc finger protein pseudogene	Other	other
ENSMUSG00000092560	Gm8750	4,18E-02	6,73E-01	0,907	archain 1 pseudogene	Other	other
ENSMUSG00000091154	Proscos	4,21E-02	1,00E+00	1,919		Other	other
ENSMUSG00000014856	TMEM208	4,21E-02	6,73E-01	-0,354	transmembrane protein 208	Other	other
ENSMUSG00000076928	Trac	4,23E-02	6,73E-01	0,746	T cell receptor alpha constant	Other	other
ENSMUSG00000074449	4930467E23Rik (includes others)	4,25E-02	1,00E+00	-1,64	RIKEN cDNA 4930467E23 gene	Other	other
ENSMUSG00000085819	Ube4bos1	4,30E-02	1,00E+00	-1,455		Other	other
ENSMUSG00000043664	TMEM221	4,31E-02	6,73E-01	-0,492	transmembrane protein 221	Other	other
ENSMUSG00000091119	CCDC152	4,34E-02	6,73E-01	-0,537	coiled-coil domain containing 152	Other	other
ENSMUSG00000052369	TMEM106C	4,36E-02	6,73E-01	-0,338	transmembrane protein 106C	Other	other
ENSMUSG00000086717	Gm15655	4,38E-02	1,00E+00	1,969		Other	other
ENSMUSG00000081254	Gm12112	4,38E-02	1,00E+00	-1,278		Other	other
ENSMUSG00000030917	TMEM159	4,41E-02	6,77E-01	-0,285	transmembrane protein 159	Other	other
ENSMUSG00000045455	Gm9797	4,42E-02	1,00E+00	0,912	translocase of inner mitochondrial membrane 8 homolog a1 pseudogene	Other	other
ENSMUSG00000097405	D630044L22Rik	4,45E-02	1,00E+00	-2,162	RIKEN cDNA gene D630044L22 gene	Other	other
ENSMUSG00000056145	AI504432	4,46E-02	6,77E-01	0,373	expressed sequence AI504432	Other	other
ENSMUSG00000070315	4930581F22Rik	4,47E-02	6,77E-01	0,319	RIKEN cDNA 4930581F22 gene	Other	other
ENSMUSG00000015981	STK32C	4,48E-02	6,77E-01	-0,795	serine/threonine kinase 32C	Other	kinase
ENSMUSG00000086825	Gm15675	4,48E-02	6,77E-01	0,61		Other	other
ENSMUSG00000044694	Gm32819	4,49E-02	6,78E-01	0,826	predicted gene, 32819	Other	other
ENSMUSG00000116594	AC133488.1	4,51E-02	1,00E+00	-1,207		Other	other
ENSMUSG00000103985	Gm37446	4,52E-02	1,00E+00	1,507		Other	other
ENSMUSG00000027574	NKAIN4	4,57E-02	6,80E-01	-0,52	sodium/potassium transporting ATPase interacting 4	Other	other
ENSMUSG00000116114	Gm35853	4,58E-02	6,80E-01	-0,34	predicted gene, 35853	Other	other
ENSMUSG00000094797	Igkv6-15	4,59E-02	1,00E+00	2,004	immunoglobulin kappa variable 6-15	Other	other
ENSMUSG00000114004	AC155937.1	4,61E-02	1,00E+00	-1,128		Other	other
ENSMUSG00000028069	GPATCH4	4,63E-02	6,80E-01	0,334	G-patch domain containing 4	Other	other
ENSMUSG00000109875	Gm45456	4,64E-02	1,00E+00	-1,235		Other	other
ENSMUSG00000109638	Gm45630	4,65E-02	1,00E+00	1,634		Other	other
ENSMUSG00000110569	Gm18860	4,69E-02	1,00E+00	-1,078	CCR4-NOT transcription complex, subunit 8 pseudogene	Other	other
ENSMUSG00000074671	Tspyl3	4,69E-02	6,80E-01	-0,305	TSPY-like 3	Other	other
ENSMUSG00000019080	MFSD3	4,71E-02	6,80E-01	-0,451	major facilitator superfamily domain containing 3	Other	other
ENSMUSG00000062456	Rpl9-ps6	4,73E-02	6,80E-01	-0,373	ribosomal protein L9, pseudogene 6	Other	other
ENSMUSG00000091491	Vmn2r101 (includes others)	4,75E-02	1,00E+00	0,849	vomeronasal 2, receptor 98	Other	other
ENSMUSG00000084897	Gm14226	4,83E-02	6,80E-01	0,765		Other	other
ENSMUSG00000101188	Eif4a-ps4	4,86E-02	6,80E-01	-0,29	eukaryotic translation initiation factor 4A, pseudogene 4	Other	other
ENSMUSG00000115344	AC154627.2	4,87E-02	1,00E+00	2,607		Other	other
ENSMUSG00000096996	Gm26792	4,89E-02	6,80E-01	0,503		Other	other
ENSMUSG00000089782	Gm3531	4,90E-02	6,80E-01	-0,635	basic transcription factor 3 pseudogene	Other	other
ENSMUSG00000086638	4930405A21Rik	4,91E-02	1,00E+00	1,678	RIKEN cDNA 4930405A21 gene	Other	other
ENSMUSG00000062933	Gm10123	4,91E-02	6,80E-01	-0,311	peptidylprolyl isomerase A-like pseudogene	Other	other
ENSMUSG00000074178	Gm10638	4,92E-02	1,00E+00	0,87	predicted gene 10638	Other	other
ENSMUSG00000096681	Rps19-ps1	4,93E-02	1,00E+00	-1,792	ribosomal protein S19, pseudogene 1	Other	other
ENSMUSG00000094974	Rps19-ps2	4,93E-02	1,00E+00	-1,792	ribosomal protein S19, pseudogene 2	Other	other
ENSMUSG00000071392	ECT2L	4,94E-02	1,00E+00	1,183	epithelial cell transforming 2 like	Other	other
ENSMUSG00000083993	Gm14398	4,96E-02	1,00E+00	-1,506	predicted gene 14398	Other	other

ENSMUSG00000081344	Gm14303	4,96E-02	6,80E-01	-0,848	ribosomal protein S29 pseudogene	Other	other
ENSMUSG00000108112	Gm45193	4,99E-02	6,80E-01	1,27		Other	other
**ENSMUSG00000045114**	**PRRT2**	**1,91E-07**	**5,49E-04**	**1,379**	**proline rich transmembrane protein 2**	**Plasma Membrane**	**other**
**ENSMUSG00000013236**	**PTPRS**	**1,15E-05**	**1,18E-02**	**0,483**	**protein tyrosine phosphatase receptor type S**	**Plasma Membrane**	**phosphatase**
**ENSMUSG00000023274**	**CD4**	**2,72E-05**	**2,44E-02**	**1,301**	**CD4 molecule**	**Plasma Membrane**	**transmembrane receptor**
**ENSMUSG00000032525**	**NKTR**	**3,49E-05**	**2,78E-02**	**0,396**	**natural killer cell triggering receptor**	**Plasma Membrane**	**enzyme**
ENSMUSG00000050357	CARMIL2	7,58E-05	5,43E-02	0,947	capping protein regulator and myosin 1 linker 2	Plasma Membrane	other
ENSMUSG00000022505	EMP2	1,60E-04	8,86E-02	-0,342	epithelial membrane protein 2	Plasma Membrane	other
ENSMUSG00000041801	PHLDA3	4,55E-04	1,42E-01	-0,514	pleckstrin homology like domain family A member 3	Plasma Membrane	other
ENSMUSG00000029530	CCR9	5,50E-04	1,48E-01	2,407	C-C motif chemokine receptor 9	Plasma Membrane	G-protein coupled receptor
ENSMUSG00000026073	IL1R2	7,05E-04	1,63E-01	1,201	interleukin 1 receptor type 2	Plasma Membrane	transmembrane receptor
ENSMUSG00000039137	WHRN	8,42E-04	1,83E-01	1,173	whirlin	Plasma Membrane	other
ENSMUSG00000001588	ACAP1	1,17E-03	2,36E-01	0,901	ArfGAP with coiled-coil, ankyrin repeat and PH domains 1	Plasma Membrane	other
ENSMUSG00000026944	ABCA2	1,43E-03	2,57E-01	0,448	ATP binding cassette subfamily A member 2	Plasma Membrane	transporter
ENSMUSG00000026395	PTPRC	1,58E-03	2,64E-01	0,7	protein tyrosine phosphatase receptor type C	Plasma Membrane	phosphatase
ENSMUSG00000073414	MPIG6B	1,71E-03	2,73E-01	0,997	megakaryocyte and platelet inhibitory receptor G6b	Plasma Membrane	other
ENSMUSG00000042747	KRTCAP2	1,78E-03	2,78E-01	-0,35	keratinocyte associated protein 2	Plasma Membrane	enzyme
ENSMUSG00000029713	GNB2	2,04E-03	2,95E-01	-0,272	G protein subunit beta 2	Plasma Membrane	enzyme
ENSMUSG00000039115	ITGA9	2,45E-03	3,16E-01	0,382	integrin subunit alpha 9	Plasma Membrane	other
ENSMUSG00000022372	SLA	2,54E-03	3,17E-01	0,844	Src like adaptor	Plasma Membrane	other
ENSMUSG00000026068	IL18RAP	2,59E-03	3,19E-01	1,276	interleukin 18 receptor accessory protein	Plasma Membrane	transmembrane receptor
ENSMUSG00000020838	SLC6A4	2,64E-03	3,19E-01	0,748	solute carrier family 6 member 4	Plasma Membrane	transporter
ENSMUSG00000019464	PTGER1	3,09E-03	3,41E-01	0,502	prostaglandin E receptor 1	Plasma Membrane	G-protein coupled receptor
ENSMUSG00000003153	SLC2A3	3,62E-03	3,62E-01	0,71	solute carrier family 2 member 3	Plasma Membrane	transporter
ENSMUSG00000026000	LANCL1	3,63E-03	3,62E-01	-0,346	LanC like 1	Plasma Membrane	enzyme
ENSMUSG00000024670	CD6	3,98E-03	3,81E-01	0,926	CD6 molecule	Plasma Membrane	transmembrane receptor
ENSMUSG00000013033	ADGRL1	4,25E-03	3,94E-01	0,404	adhesion G protein-coupled receptor L1	Plasma Membrane	G-protein coupled receptor
ENSMUSG00000001943	VSIG2	4,41E-03	4,01E-01	0,365	V-set and immunoglobulin domain containing 2	Plasma Membrane	other
ENSMUSG00000051790	NLGN2	4,73E-03	4,03E-01	0,298	neuroligin 2	Plasma Membrane	enzyme
ENSMUSG00000048163	SELPLG	4,77E-03	4,03E-01	0,763	selectin P ligand	Plasma Membrane	other
ENSMUSG00000028465	TLN1	4,89E-03	4,03E-01	0,394	talin 1	Plasma Membrane	other
ENSMUSG00000026117	ZAP70	5,05E-03	4,03E-01	0,918	zeta chain of T cell receptor associated protein kinase 70	Plasma Membrane	kinase
ENSMUSG00000046822	SLC39A3	5,18E-03	4,03E-01	-0,275	solute carrier family 39 member 3	Plasma Membrane	transporter
ENSMUSG00000003420	FCGRT	5,27E-03	4,04E-01	-0,389	Fc fragment of IgG receptor and transporter	Plasma Membrane	transmembrane receptor
ENSMUSG00000052013	BTLA	5,96E-03	4,23E-01	0,837	B and T lymphocyte associated	Plasma Membrane	other
ENSMUSG00000027863	CD2	6,32E-03	4,30E-01	0,606	CD2 molecule	Plasma Membrane	transmembrane receptor
ENSMUSG00000026012	CD28	6,75E-03	4,34E-01	0,723	CD28 molecule	Plasma Membrane	transmembrane receptor
ENSMUSG00000020994	PNN	6,75E-03	4,34E-01	0,298	pinin, desmosome associated protein	Plasma Membrane	other
ENSMUSG00000027009	ITGA4	7,34E-03	4,55E-01	0,508	integrin subunit alpha 4	Plasma Membrane	transmembrane receptor
ENSMUSG00000029699	SSC4D	7,58E-03	1,00E+00	-1,667	scavenger receptor cysteine rich family member with 4 domains	Plasma Membrane	transmembrane receptor
ENSMUSG00000050777	TMEM37	8,42E-03	4,81E-01	-0,453	transmembrane protein 37	Plasma Membrane	ion channel
ENSMUSG00000028955	VAMP3	8,58E-03	4,82E-01	-0,339	vesicle associated membrane protein 3	Plasma Membrane	other
ENSMUSG00000050921	P2RY10	8,94E-03	4,88E-01	0,684	P2Y receptor family member 10	Plasma Membrane	G-protein coupled receptor
ENSMUSG00000034656	CACNA1A	8,95E-03	4,88E-01	0,663	calcium voltage-gated channel subunit alpha1 A	Plasma Membrane	ion channel
ENSMUSG00000021298	GPR132	9,23E-03	4,91E-01	0,682	G protein-coupled receptor 132	Plasma Membrane	G-protein coupled receptor
ENSMUSG00000090136	SEC61G	9,54E-03	4,92E-01	-0,755	SEC61 translocon gamma subunit	Plasma Membrane	transporter
ENSMUSG00000030745	IL21R	9,72E-03	4,93E-01	0,736	interleukin 21 receptor	Plasma Membrane	transmembrane receptor
ENSMUSG00000050075	GPR171	9,92E-03	4,96E-01	0,815	G protein-coupled receptor 171	Plasma Membrane	G-protein coupled receptor
ENSMUSG00000001029	ICAM2	9,95E-03	4,96E-01	-0,265	intercellular adhesion molecule 2	Plasma Membrane	other
ENSMUSG00000071068	TREML2	1,01E-02	4,96E-01	0,825	triggering receptor expressed on myeloid cells like 2	Plasma Membrane	other
ENSMUSG00000066684	PILRB	1,09E-02	5,04E-01	-1,174	paired immunoglobin like type 2 receptor beta	Plasma Membrane	other
ENSMUSG00000026399	CD55	1,14E-02	5,04E-01	0,356	CD55 molecule (Cromer blood group)	Plasma Membrane	other
ENSMUSG00000030742	LAT	1,17E-02	5,04E-01	0,893	linker for activation of T cells	Plasma Membrane	other
ENSMUSG00000047139	Cd24a	1,19E-02	5,08E-01	-0,285	CD24a antigen	Plasma Membrane	other
ENSMUSG00000022146	OSMR	1,20E-02	5,08E-01	0,282	oncostatin M receptor	Plasma Membrane	transmembrane receptor
ENSMUSG00000056290	Ms4a4b (includes others)	1,29E-02	5,22E-01	0,723	membrane-spanning 4-domains, subfamily A, member 4B	Plasma Membrane	other
ENSMUSG00000034463	SCARA3	1,33E-02	5,22E-01	-0,82	scavenger receptor class A member 3	Plasma Membrane	transmembrane receptor
ENSMUSG00000027220	SYT13	1,37E-02	1,00E+00	2,347	synaptotagmin 13	Plasma Membrane	transporter
ENSMUSG00000022231	SEMA5A	1,37E-02	5,27E-01	-0,431	semaphorin 5A	Plasma Membrane	transmembrane receptor
ENSMUSG00000034028	CD226	1,40E-02	5,28E-01	0,754	CD226 molecule	Plasma Membrane	other
ENSMUSG00000044199	S1PR4	1,44E-02	5,34E-01	-0,288	sphingosine-1-phosphate receptor 4	Plasma Membrane	G-protein coupled receptor
ENSMUSG00000066043	PHACTR4	1,44E-02	5,34E-01	0,32	phosphatase and actin regulator 4	Plasma Membrane	other
ENSMUSG00000029761	Cald1	1,48E-02	5,40E-01	0,363	caldesmon 1	Plasma Membrane	other
ENSMUSG00000024669	CD5	1,55E-02	5,48E-01	0,906	CD5 molecule	Plasma Membrane	transmembrane receptor
ENSMUSG00000036353	P2RY12	1,57E-02	5,54E-01	0,998	purinergic receptor P2Y12	Plasma Membrane	G-protein coupled receptor
ENSMUSG00000041046	RAMP3	1,62E-02	5,58E-01	-0,64	receptor activity modifying protein 3	Plasma Membrane	other
ENSMUSG00000022817	ITGB5	1,63E-02	5,58E-01	-0,267	integrin subunit beta 5	Plasma Membrane	other
ENSMUSG00000028613	LRP8	1,65E-02	1,00E+00	1,166	LDL receptor related protein 8	Plasma Membrane	transmembrane receptor
ENSMUSG00000041078	GRID1	1,68E-02	5,62E-01	-2,078	glutamate ionotropic receptor delta type subunit 1	Plasma Membrane	ion channel
ENSMUSG00000027800	TM4SF1	1,70E-02	5,62E-01	-0,672	transmembrane 4 L six family member 1	Plasma Membrane	other
ENSMUSG00000053977	CD8A	1,73E-02	5,64E-01	1,62	CD8a molecule	Plasma Membrane	other
ENSMUSG00000017412	CACNB4	1,77E-02	5,64E-01	1,639	calcium voltage-gated channel auxiliary subunit beta 4	Plasma Membrane	ion channel
ENSMUSG00000050931	SGMS2	1,82E-02	5,64E-01	0,767	sphingomyelin synthase 2	Plasma Membrane	enzyme
ENSMUSG00000034664	ITGA2B	1,84E-02	5,64E-01	0,661	integrin subunit alpha 2b	Plasma Membrane	transmembrane receptor
ENSMUSG00000018569	CLDN7	1,90E-02	5,67E-01	-0,782	claudin 7	Plasma Membrane	other
ENSMUSG00000051043	GPRC5C	1,97E-02	5,75E-01	-0,511	G protein-coupled receptor class C group 5 member C	Plasma Membrane	G-protein coupled receptor
ENSMUSG00000043953	CCRL2	2,05E-02	5,84E-01	-0,298	C-C motif chemokine receptor like 2	Plasma Membrane	G-protein coupled receptor
ENSMUSG00000047414	FLRT2	2,06E-02	5,85E-01	0,606	fibronectin leucine rich transmembrane protein 2	Plasma Membrane	other
ENSMUSG00000047953	GP5	2,21E-02	6,03E-01	0,844	glycoprotein V platelet	Plasma Membrane	other
ENSMUSG00000002981	CLPTM1	2,26E-02	6,03E-01	-0,331	CLPTM1 regulator of GABA type A receptor forward trafficking	Plasma Membrane	other
ENSMUSG00000031775	PLLP	2,29E-02	6,03E-01	-0,29	plasmolipin	Plasma Membrane	transporter
ENSMUSG00000053310	Nrgn	2,33E-02	6,03E-01	1,233	neurogranin	Plasma Membrane	other
ENSMUSG00000028618	TMEM59	2,39E-02	6,03E-01	-0,495	transmembrane protein 59	Plasma Membrane	peptidase
ENSMUSG00000026581	SELL	2,40E-02	6,03E-01	0,692	selectin L	Plasma Membrane	transmembrane receptor
ENSMUSG00000030054	GP9	2,46E-02	6,05E-01	0,79	glycoprotein IX platelet	Plasma Membrane	other
ENSMUSG00000033917	GDE1	2,48E-02	6,05E-01	-0,287	glycerophosphodiester phosphodiesterase 1	Plasma Membrane	enzyme
ENSMUSG00000037031	TSPAN15	2,50E-02	6,05E-01	-0,437	tetraspanin 15	Plasma Membrane	other
ENSMUSG00000038319	KCNH2	2,51E-02	6,05E-01	-0,513	potassium voltage-gated channel subfamily H member 2	Plasma Membrane	ion channel
ENSMUSG00000028854	SLC9A1	2,51E-02	6,05E-01	0,282	solute carrier family 9 member A1	Plasma Membrane	ion channel
ENSMUSG00000015316	SLAMF1	2,53E-02	6,05E-01	1,029	signaling lymphocytic activation molecule family member 1	Plasma Membrane	transmembrane receptor
ENSMUSG00000030365	Clec2d	2,54E-02	6,05E-01	1,065	C-type lectin domain family 2, member d	Plasma Membrane	transmembrane receptor
ENSMUSG00000040860	CROCC	2,56E-02	6,05E-01	0,454	ciliary rootlet coiled-coil, rootletin	Plasma Membrane	other
ENSMUSG00000058153	SEZ6L	2,75E-02	1,00E+00	1,502	seizure related 6 homolog like	Plasma Membrane	other
ENSMUSG00000050675	GP1BA	2,84E-02	6,20E-01	0,704	glycoprotein Ib platelet subunit alpha	Plasma Membrane	transmembrane receptor
ENSMUSG00000014158	TRPV4	2,84E-02	6,20E-01	-0,29	transient receptor potential cation channel subfamily V member 4	Plasma Membrane	ion channel
ENSMUSG00000021303	GNG4	3,02E-02	1,00E+00	-1,276	G protein subunit gamma 4	Plasma Membrane	enzyme
ENSMUSG00000044279	Crb3	3,13E-02	6,39E-01	-0,425	crumbs family member 3	Plasma Membrane	other
ENSMUSG00000038607	GNG10	3,35E-02	6,43E-01	-0,35	G protein subunit gamma 10	Plasma Membrane	other
ENSMUSG00000076617	IGHM	3,39E-02	6,43E-01	0,66	immunoglobulin heavy constant mu	Plasma Membrane	transmembrane receptor
ENSMUSG00000003882	IL7R	3,40E-02	6,43E-01	0,571	interleukin 7 receptor	Plasma Membrane	transmembrane receptor
ENSMUSG00000026131	Dst	3,54E-02	6,48E-01	0,283	dystonin	Plasma Membrane	other
ENSMUSG00000027737	SLC7A11	3,63E-02	6,53E-01	1,039	solute carrier family 7 member 11	Plasma Membrane	transporter
ENSMUSG00000098112	BIN2	3,94E-02	6,69E-01	0,599	bridging integrator 2	Plasma Membrane	other
ENSMUSG00000006641	SLC5A6	3,94E-02	6,69E-01	-0,414	solute carrier family 5 member 6	Plasma Membrane	transporter
ENSMUSG00000031239	ITM2A	4,10E-02	6,73E-01	-0,427	integral membrane protein 2A	Plasma Membrane	other
ENSMUSG00000031561	TENM3	4,15E-02	6,73E-01	0,871	teneurin transmembrane protein 3	Plasma Membrane	other
ENSMUSG00000022416	CACNA1I	4,16E-02	6,73E-01	0,927	calcium voltage-gated channel subunit alpha1 I	Plasma Membrane	ion channel
ENSMUSG00000026791	SLC2A8	4,21E-02	6,73E-01	0,36	solute carrier family 2 member 8	Plasma Membrane	transporter
ENSMUSG00000047880	CXCR5	4,25E-02	6,73E-01	0,967	C-X-C motif chemokine receptor 5	Plasma Membrane	G-protein coupled receptor
ENSMUSG00000033470	CYSLTR2	4,27E-02	6,73E-01	0,562	cysteinyl leukotriene receptor 2	Plasma Membrane	G-protein coupled receptor

ENSMUSG00000053141	PTPRT	4,31E-02	6,73E-01	0,791	protein tyrosine phosphatase receptor type T	Plasma Membrane	phosphatase
ENSMUSG00000025492	IFITM3	4,31E-02	6,73E-01	-0,263	interferon induced transmembrane protein 3	Plasma Membrane	other
ENSMUSG00000073002	VAMP5	4,35E-02	6,73E-01	-0,287	vesicle associated membrane protein 5	Plasma Membrane	transporter
ENSMUSG00000030854	PTPN5	4,40E-02	1,00E+00	-1,539	protein tyrosine phosphatase non-receptor type 5	Plasma Membrane	phosphatase
ENSMUSG00000042265	TREM1	4,45E-02	6,77E-01	0,688	triggering receptor expressed on myeloid cells 1	Plasma Membrane	transmembrane receptor
ENSMUSG00000079227	CCR5	4,45E-02	6,77E-01	-0,892	C-C motif chemokine receptor 5 (gene/pseudogene)	Plasma Membrane	G-protein coupled receptor
ENSMUSG00000030830	ITGAL	4,62E-02	6,80E-01	0,52	integrin subunit alpha L	Plasma Membrane	transmembrane receptor
ENSMUSG00000002033	CD3G	4,69E-02	6,80E-01	0,883	CD3g molecule	Plasma Membrane	transmembrane receptor
ENSMUSG00000034863	ANO8	4,70E-02	6,80E-01	0,311	anoctamin 8	Plasma Membrane	ion channel
ENSMUSG00000005540	FCER2	4,73E-02	6,80E-01	0,915	Fc fragment of IgE receptor II	Plasma Membrane	transmembrane receptor
ENSMUSG00000004655	AQP1	4,77E-02	6,80E-01	-0,347	aquaporin 1 (Colton blood group)	Plasma Membrane	transporter
ENSMUSG00000021070	BDKRB2	4,81E-02	6,80E-01	0,316	bradykinin receptor B2	Plasma Membrane	G-protein coupled receptor
ENSMUSG00000039155	CDH26	4,83E-02	6,80E-01	1,128	cadherin 26	Plasma Membrane	other
