# Supplementary material for: Autophagy protein 5 controls flow-dependent endothelial functions
Source: Cell Mol Life Sci. 2023 Jul 18;80(8):210. doi: 10.1007/s00018-023-04859-9 (PMC10352428; doi:10.1007/s00018-023-04859-9)
Supplement: Supplementary file 3 — Supplementary file3 (PDF 40 KB) [file 18_2023_4859_MOESM3_ESM.pdf]

Categories	Functions	Diseases or Functions Annotation	p-value	Activation z-score	Molecules	# Molecules
Cell-To-Cell Signaling and Interaction,Hematological System Development and Function	activation	Activation of blood cells	1,13E-02	0,956	CCDC88B,CD4,GP5,HDAC9,IGHM,IL18RAP,LCP2,P2RY12,PILRB,PLEK,PRKCB,PTPN22,RASGRP1,SATB1,WAS	15
Cell-To-Cell Signaling and Interaction	activation	Activation of cells	1,19E-02	0,956	CCDC88B,Cd24a,CD4,GP5,HDAC9,IGHM,IL18RAP,LCP2,P2RY12,PILRB,PLEK,PRKCB,PTPN22,RASGRP1,SATB1,WAS	16
Cell Signaling	activation	Activation of enzyme	2,17E-02		AVPI1,AXIN1,BBC3,FABP4,GADD45G,IGHM,ITK,KAT2B,LRP8,MADD,PHACTR4,PRDX2,PTPRC,S1PR4,SLC37A4,TF	16
Cell Signaling,Post-Translational Modification	activation	Activation of MAP kinase	1,79E-02		AVPI1,IGHM,MADD,PRDX2,PTPRC	5
Cell Signaling,Post-Translational Modification	activation	Activation of Protein kinase	6,88E-03		AVPI1,AXIN1,FABP4,GADD45G,IGHM,KAT2B,LRP8,MADD,PRDX2,PTPRC,TF	11
Cell-To-Cell Signaling and Interaction,Hematological System Development and Function	aggregation	Aggregation of blood cells	3,13E-03		ALOX12,Cd24a,PLEK,PTPN6,SLC7A11	5
Cell-To-Cell Signaling and Interaction,Hematological System Development and Function,Inflammatory Response	aggregation	Aggregation of blood platelets	1,39E-02		ALOX12,PLEK,PTPN6,SLC7A11	4
Cell-To-Cell Signaling and Interaction,Hematological System Development and Function	aggregation	Aggregation of red blood cells	3,65E-02		Cd24a	1
Cell Death and Survival	apoptosis	Initiation of apoptosis	2,03E-02		BBC3,Cd24a,CD28,CD5	4
Nucleic Acid Metabolism,Small Molecule Biochemistry	biosynthesis	Biosynthesis of dATP	3,65E-02		ADK	1
Lipid Metabolism,Small Molecule Biochemistry,Vitamin and Mineral Metabolism	biosynthesis	Biosynthesis of vitamin E	3,65E-02		PLTP	1
Carbohydrate Metabolism,Small Molecule Biochemistry	catabolism	Catabolism of chitin	3,65E-02		CHIA	1
Free Radical Scavenging,Small Molecule Biochemistry	catabolism	Catabolism of hydrogen peroxide	2,48E-02		PRDX2,PRDX3	2
Drug Metabolism	catabolism	Catabolism of xenobiotic	3,65E-02		GSTM4	1
Cell Cycle	cell cycle progression	Cell cycle progression	2,09E-02	2,236	AATF,CALR,CITED2,EPM2A,GADD45G,JUNB,MADD,PHACTR4,PHIP,PTPRC,RPL24,SFPQ,SLC6A4,TUBB1	14
Cellular Movement	chemotaxis	Chemotaxis	1,74E-02	-0,200	CCL24,CCR9,CXCR5,ITGA9,PTN,S100A9,SEMA5A,SLAMF1,SLC37A4,STAP1,TREM1,TRPV4	12
Cell Death and Survival,Cellular Function and Maintenance,Hematological System Development and Function	clearance	Clearance of red blood cells	3,65E-02		Cd24a	1
Hematological System Development and Function,Organismal Functions	coagulation	Coagulation of blood	7,25E-03		F13A1,F5,GP1BA,GP5,MPIG6B,P2RY12,PLEK	7
Post-Translational Modification	dephosphorylation	Dephosphorylation of protein	3,35E-03		DUSP10,Ppp2r3d,PTPN22,PTPN5,PTPN6,PTPRC,PTPRT	7
Cellular Assembly and Organization,Cellular Compromise	depolymerization	Depolymerization of actin filaments	8,34E-03		CFL2,PLEK,SEMA5A	3
Cellular Assembly and Organization,Cellular Compromise	depolymerization	Depolymerization of filaments	2,03E-02		CFL2,FGF13,PLEK,SEMA5A	4
Cellular Assembly and Organization	development	Development of cytoplasm	8,17E-03		AATF,ALOX15,Clec2d (includes others),CTSD,EPM2A,FCHSD1,FGF13,FIS1,ITGB5,SLIRP,Tmsb4x (includes others),WAS	12
Cellular Development,Cellular Growth and Proliferation,Hematological System Development and Function,Hematopoiesis,Tissue Development	differentiation	Differentiation of hematopoietic progenitor cells	1,21E-02		Cd24a,CEBPD,CITED2,GPATCH4,HERC6,PRRC2C,PTPN6,RBM47,SIN3A,SIPA1L3,SLC37A4,TET2	12
Cellular Development	differentiation	Differentiation of stem cells	1,28E-02		A2M,Cd24a,CDK13,CEBPD,CITED2,GPATCH4,HERC6,PRRC2C,PTN,PTPN6,RBM47,SIN3A,SIPA1L3,SLC37A4,TET2	15
Cellular Assembly and Organization,Cellular Function and Maintenance,Cellular Movement,Nervous System Development and Function	endocytosis	Endocytosis of synaptic vesicles	3,96E-02		ACTG1,Cd24a,DNM1	3
Cell Cycle,Cellular Assembly and Organization	fission	Fission of peroxisomes	3,65E-02		FIS1	1
Cellular Function and Maintenance	homeostasis	Cellular homeostasis	1,91E-02	1,534	AQP1,ATOX1,BCL11B,CACNA1A,CACNB4,CARMIL2,Cd24a,CD28,CD4,CD8A,CFL2,CTNNB1,DMPK,DTX1,EPM2A,GADD45G,IKZF1,IL7R,ITK,KCNH2,LAT,LCK,NCOR1,NKAP,PRDX2,PRKCB,PTPN22,PTPRC,SLAMF1,SLC37A4,SLC39A3,SLC9A1,SRL,TET2,TMEM98,TRPV4,ZAP70	37
Cellular Function and Maintenance	homeostasis	Homeostasis of blood cells	3,16E-05	1,942	BCL11B,CARMIL2,CD28,CD4,CD8A,CTNNB1,DTX1,GADD45G,IKZF1,IL7R,ITK,LAT,LCK,NCOR1,NKAP,PRDX2,PTPN22,PTPRC,SLAMF1,SLC37A4,SLC39A3,TET2,TMEM98,ZAP70	24
Cellular Function and Maintenance	maintenance	Maintenance of cells	3,57E-03		CLN8,CROCC,CTNNB1,MED12,MED28,NKAP,PADI4,PAF1,Panct2,TPT1	10
Cellular Function and Maintenance	maintenance	Maintenance of stem cells	2,97E-03		CTNNB1,MED12,MED28,NKAP,PADI4,PAF1,Panct2,TPT1	8
Cell Signaling,Nucleic Acid Metabolism,Small Molecule Biochemistry	metabolism	Metabolism of cyclic AMP	3,65E-02		CACNB4	1
Carbohydrate Metabolism,Small Molecule Biochemistry	metabolism	Metabolism of D-xylulose	3,65E-02		DCXR	1
Carbohydrate Metabolism,Small Molecule Biochemistry	metabolism	Metabolism of galactitol	3,65E-02		GALK1	1
Small Molecule Biochemistry	metabolism	Metabolism of nitrobenzene	3,65E-02		GSTM4	1
Lipid Metabolism,Small Molecule Biochemistry	metabolism	Conversion of fatty acid	1,80E-02	-0.900	ALDH3A1,ALOX15,FABP4,PDGFB	4
Lipid Metabolism,Small Molecule Biochemistry	metabolism	Folding of lipid	1,33E-03		SFTPB,SFTPC	2
Amino Acid Metabolism,Post-Translational Modification,Small Molecule Biochemistry	metabolism	Phosphorylation of L-tyrosine	6,57E-03	1.532	Cd24a,CD4,CLK3,DYRK3,IGHM,LCK,LRP8,PDGFB,PTPRC,ZAP70	10
Lipid Metabolism,Small Molecule Biochemistry	metabolism	Production of ketone body	1,24E-02		SIRT3,VHL	2
Lipid Metabolism,Small Molecule Biochemistry	metabolism	Synthesis of 12(S)-hydroxyeicosatetraenoic acid	1,24E-02		ALOX12,ALOX15	2
Lipid Metabolism,Small Molecule Biochemistry	metabolism	Synthesis of arachidonic acid	1,31E-02		ALOX12,ALOX15,CTNNB1	3
Lipid Metabolism,Small Molecule Biochemistry	metabolism	Synthesis of prostaglandin F	8,34E-03		AKR1B1,IGHM,PTGIS	3
Carbohydrate Metabolism,Molecular Transport	metabolism	Transport of dehydroascorbic acid	1,24E-02		SLC2A3,SLC2A8	2
Amino Acid Metabolism,Small Molecule Biochemistry	metabolism	Inhibition of amino acids	1,24E-02		CXCL14,TSHB	2
Cellular Assembly and Organization,Cellular Function and Maintenance	organization	Organization of actin cytoskeleton	6,61E-03		BRK1,CALR,CFL2,DIXDC1,Pakap,PDGFB,PHACTR4,RAN,S100A9,TF,TLN1,Tmsb4x (includes others)	12
Cell Cycle,Cellular Assembly and Organization,Cellular Function and Maintenance,DNA Replication, Recombination, and Repair	organization	Organization of chromosomes	4,67E-02		HDAC9,PADI4,SATB1,SFPQ,WDHD1	5
Cellular Assembly and Organization,Cellular Function and Maintenance	organization	Organization of cytoplasmic microtubule	6,43E-03		AXIN1,Dst,HOOK1	3
Cellular Assembly and Organization,Cellular Function and Maintenance	organization	Organization of cytoskeleton	4,60E-02	-2,000	AJUBA,ATRX,AXIN1,BCL11B,BRK1,CACNA1A,CALR,Cd24a,CEP350,CFL2,CLN8,CYFIP2,DIXDC1,DRAXIN,Dst,FGF13,HOOK1,Krt10,LRP8,P2RY12,Pakap,PDGFB,PHACTR4,PTN,PTPRS,RAN,RERE,RNF165,S100A9,SEMA5A,SEPTIN2,SIN3A,SIPA1L3,TF,TLN1,Tmsb4x (includes others),YWHAH	37
Cellular Assembly and Organization,Cellular Function and Maintenance	organization	Organization of filaments	3,04E-02		AXIN1,CFL2,CLN8,Dst,HOOK1,Krt10,MFAP5,Pakap,TF	9
Cellular Assembly and Organization,Cellular Function and Maintenance	organization	Organization of nucleus	2,09E-02		DMPK,HDAC9,IRAG2,PADI4,SATB1,SFPQ,WDHD1	7
Cellular Assembly and Organization	organization	Organization of organelle	4,18E-02		AXIN1,CFL2,CLN8,CTNNB1,DMPK,Dst,EPM2A,HDAC9,HOOK1,IFT27,IRAG2,Krt10,MFAP5,PADI4,Pakap,PTPRS,SATB1,SFPQ,SLC7A11,TF,USE1,WDHD1,WHRN	23
Cell Signaling	osmosensory signaling pathway	Osmosensory signaling pathway	3,65E-02		TRPV4	1
Amino Acid Metabolism,Post-Translational Modification,Small Molecule Biochemistry	phosphorylation	Phosphorylation of L-tyrosine	4,42E-03	1,195	Cd24a,CD4,CLK3,DYRK3,IGHM,LCK,LRP8,PDGFB,PTPRC,ZAP70	10
Cellular Development,Cellular Growth and Proliferation	proliferation	Proliferation of blood cells	3,42E-05	1,452	ATAD5,BTLA,CARMIL2,CCDC88B,Cd24a,CD28,Clec2d (includes others),CTNNB1,IGHM,IKZF1,IKZF3,IL7R,ITGAL,NKAP,PRDX2,Prg4,PTPN6,PTPRC,SATB1,SLAMF1,ZAP70,ZC3H12D	22
Protein Trafficking	recruitment	Recruitment of protein	1,21E-02		CARMIL2,IKZF1,KIF14,WHRN	4
Cell Cycle,Cellular Assembly and Organization,DNA Replication, Recombination, and Repair	remodeling	Remodeling of chromatin	4,13E-02		BAZ1A,PADI4,RERE,SATB1	4
Amino Acid Metabolism,Lipid Metabolism,Molecular Transport,Small Molecule Biochemistry	secretion	Secretion of GABA	1,81E-02		CACNA1A,CACNB4	2
Cell Signaling,Vitamin and Mineral Metabolism	signaling	Signaling of Ca2+	4,74E-04	2,000	CD4,CD8A,ITGAL,ZAP70	4
Cell Signaling,Cellular Function and Maintenance,Molecular Transport,Vitamin and Mineral Metabolism	signaling	Flux of Ca2+	7,06E-03	1.287	A2M,CCL24,CCR5,CD226,CD28,CD4,CD5,IGHM,ITK,JUNB,LAT,LCP2,MYB,PRKCB,SRL,TRPV4,WAS,ZAP70	18
Cell Signaling,Cellular Function and Maintenance,Molecular Transport,Vitamin and Mineral Metabolism	signaling	Influx of Ca2+	1,33E-02	0.722	A2M,CD28,CD5,IGHM,ITK,JUNB,LAT,MYB,PRKCB,SRL,TRPV4,WAS,ZAP70	13
Cell Signaling,Molecular Transport,Vitamin and Mineral Metabolism	signaling	Mobilization of Ca2+	1,95E-05	1.786	A2M,CCR5,CCR9,CD2,CD28,CD4,CD5,CD6,CXCR5,CYSLTR2,FCER2,GNB2,ITK,LAT,LCK,LCP2,PTGER1,PTPN6,PTPRC,SH2D2A,SRL,TREM1,ZAP70	23
Cell Signaling,Vitamin and Mineral Metabolism	signaling	Signaling of Ca2+	8,22E-05	1.890	A2M,CD4,CD8A,CTNNB1,Cxcl9,ITGAL,ZAP70	7
RNA Post-Transcriptional Modification	splicing	Splicing of mRNA	4,67E-02		CDK13,DDX5,HNRNPA2B1,SFSWAP,Srrm1	5
Lipid Metabolism,Small Molecule Biochemistry	synthesis	Synthesis of lipoxin A4	3,65E-02		ALOX15	1
Small Molecule Biochemistry	synthesis	Synthesis of spermidine	3,65E-02		AMD1	1
Small Molecule Biochemistry	synthesis	Synthesis of spermine	3,65E-02		AMD1	1
Drug Metabolism,Molecular Transport	transmembrane transport	Transmembrane transport of glutathione	3,65E-02		SLC7A11	1
Carbohydrate Metabolism,Molecular Transport,Small Molecule Biochemistry	transport	Transport of D-fructose	3,65E-02		SLC2A8	1
Carbohydrate Metabolism,Molecular Transport	transport	Transport of dehydroascorbic acid	7,61E-03		SLC2A3,SLC2A8	2
Carbohydrate Metabolism,Molecular Transport	transport	Transport of formic acid	3,65E-02		SLC26A6	1
Carbohydrate Metabolism,Molecular Transport,Small Molecule Biochemistry	transport	Transport of galactose	3,65E-02		SLC2A8	1
Molecular Transport	transport	Transport of inorganic cation	1,10E-02		AQP1,ATOX1,Atp5e,CACNA1A,CACNB4,DMPK,EPM2A,FGF13,KCNH2,MYB,PRKCB,PRSS8,SLC39A3,SLC9A1,TF,YWHAH	16
Molecular Transport	transport	Transport of ion	4,37E-02		AQP1,ATOX1,Atp5e,CACNA1A,CACNB4,DMPK,EPM2A,FGF13,KCNH2,MYB,P2RY12,PRKCB,PRSS8,SLC26A6,SLC39A3,SLC9A1,TF,YWHAH	18
Amino Acid Metabolism,Molecular Transport,Small Molecule Biochemistry	transport	Transport of L-glutamic acid	4,94E-02		EPM2A,SEPTIN2	2
Molecular Transport	transport	Transport of metal ion	1,00E-02		AQP1,ATOX1,CACNA1A,CACNB4,DMPK,EPM2A,FGF13,KCNH2,MYB,PRKCB,PRSS8,SLC39A3,SLC9A1,TF,YWHAH	15
Molecular Transport	transport	Transport of Na+	4,67E-02		DMPK,FGF13,PRSS8,SLC9A1,YWHAH	5
Cellular Assembly and Organization	transport	Transport of vesicles	1,36E-02		ACTG1,Cd24a,CTNNB1,DNM1,KIFC1,RASGRP1	6
