# Supplementary material for: Autophagy protein 5 controls flow-dependent endothelial functions
Source: Cell Mol Life Sci. 2023 Jul 18;80(8):210. doi: 10.1007/s00018-023-04859-9 (PMC10352428; doi:10.1007/s00018-023-04859-9)
Supplement: Supplementary file 4 — Supplementary file4 (PDF 96 KB) [file 18_2023_4859_MOESM4_ESM.pdf]

Expr p-value	-Log(p-value)	Expr FDR (q-value)	Expr Fold Change	ID	Symbol	Entrez Gene Name	Location	Type(s)
3,23E-02	1,49E+00	1,64E-02	-1,401	Q9D358	ACP1	acid phosphatase 1	Cytoplasm	phosphatase
6,60E-03	2,18E+00	3,99E-03	1,591	Q8CG76	AKR7A2	aldo-keto reductase family 7 member A2	Cytoplasm	enzyme
1,14E-02	1,94E+00	6,16E-03	-2,008	A2AH22	AMBRA1	autophagy and beclin 1 regulator 1	Cytoplasm	other
6,50E-03	2,19E+00	2,10E-03	-1,513	Q9CPQ8	ATP5MG	ATP synthase membrane subunit g	Cytoplasm	enzyme
4,53E-02	1,34E+00	8,44E-03	-1,216	Q9DB20	ATP5PO	ATP synthase peripheral stalk subunit OSCP	Cytoplasm	transporter
3,55E-02	1,45E+00	3,60E-02	1,797	P70295	AUP1	AUP1 lipid droplet regulating VLDL assembly factor	Cytoplasm	other
1,90E-02	1,72E+00	1,32E-02	-2,171	O35127	C12orf57	chromosome 12 open reading frame 57	Cytoplasm	other
9,99E-03	2,00E+00	9,99E-03	25	Q8WUR0	C19orf12	chromosome 19 open reading frame 12	Cytoplasm	other
1,70E-02	1,77E+00	4,76E-02	-1,3	O88456	CAPNS1	calpain small subunit 1	Cytoplasm	peptidase
7,52E-03	2,12E+00	1,16E-03	-1,278	Q9D1I2	CARD19	caspase recruitment domain family member 19	Cytoplasm	other
9,99E-03	2,00E+00	9,99E-03	25	O08736	Casp12	caspase 12	Cytoplasm	peptidase
9,99E-03	2,00E+00	2,96E-02	1,79	Q8BT07	CEP55	centrosomal protein 55	Cytoplasm	other
8,65E-03	2,06E+00	3,04E-02	1,312	Q9QXG2	CHM	CHM Rab escort protein	Cytoplasm	enzyme
9,42E-03	2,03E+00	3,45E-02	-1,395	Q60680	CHUK	component of inhibitor of nuclear factor kappa B kinase complex	Cytoplasm	kinase
1,13E-02	1,95E+00	1,60E-02	-1,815	Q8BXK9	CLIC5	chloride intracellular channel 5	Cytoplasm	ion channel
3,84E-02	1,42E+00	4,28E-02	1,914	Q9CWN7	CNOT11	CCR4-NOT transcription complex subunit 11	Cytoplasm	other
1,17E-02	1,93E+00	3,76E-03	1,252	Q9DAU1	CNPY3	canopy FGF signaling regulator 3	Cytoplasm	other
1,86E-02	1,73E+00	2,41E-02	1,94	P10605	CTSB	cathepsin B	Cytoplasm	peptidase
4,79E-02	1,32E+00	2,66E-02	1,853	Q9WUU7	CTSZ	cathepsin Z	Cytoplasm	peptidase
4,84E-02	1,32E+00	4,97E-02	-1,244	Q9CQX2	CYB5B	cytochrome b5 type B	Cytoplasm	enzyme
2,90E-04	3,54E+00	4,52E-04	-2,075	P12791	CYP2B6	cytochrome P450 family 2 subfamily B member 6	Cytoplasm	enzyme
2,22E-02	1,65E+00	3,61E-02	-1,26	Q9CQ62	DECR1	2,4-dienoyl-CoA reductase 1	Cytoplasm	enzyme
2,61E-03	2,58E+00	4,95E-02	-1,334	A2RT67	DENND3	DENN domain containing 3	Cytoplasm	other
3,16E-03	2,50E+00	3,92E-02	-1,268	Q9D7X3	DUSP3	dual specificity phosphatase 3	Cytoplasm	phosphatase
4,84E-02	1,32E+00	1,17E-02	-1,269	Q9WVL6	EXTL3	exostosin like glycosyltransferase 3	Cytoplasm	enzyme
4,19E-02	1,38E+00	1,72E-02	1,29	Q00612	G6PD	glucose-6-phosphate dehydrogenase	Cytoplasm	enzyme
9,99E-03	2,00E+00	9,99E-03	25	P54818	GALC	galactosylceramidase	Cytoplasm	enzyme
3,19E-02	1,50E+00	4,96E-03	1,235	Q6P5E6	GGA2	golgi associated, gamma adaptin ear containing, ARF binding protein 2	Cytoplasm	transporter
3,47E-03	2,46E+00	3,03E-02	1,742	P23336	Ggta1	glycoprotein galactosyltransferase alpha 1, 3	Cytoplasm	other
3,71E-02	1,43E+00	3,97E-02	1,225	Q91VC9	GHITM	growth hormone inducible transmembrane protein	Cytoplasm	other
2,47E-02	1,61E+00	1,03E-02	-1,258	Q9JK38	GNPNAT1	glucosamine-phosphate N-acetyltransferase 1	Cytoplasm	enzyme
2,99E-02	1,52E+00	4,12E-02	-1,493	Q91XR9	GPX4	glutathione peroxidase 4	Cytoplasm	enzyme
8,86E-03	2,05E+00	4,33E-02	-1,916	O54865	GUCY1B1	guanylate cyclase 1 soluble subunit beta 1	Cytoplasm	enzyme
4,57E-02	1,34E+00	3,38E-02	1,606	P12265	GUSB	glucuronidase beta	Cytoplasm	enzyme
3,04E-02	1,52E+00	1,92E-02	2,051	P20060	HEXB	hexosaminidase subunit beta	Cytoplasm	enzyme
1,75E-02	1,76E+00	2,60E-02	-1,881	Q8VCC1	HPGD	15-hydroxyprostaglandin dehydrogenase	Cytoplasm	enzyme
4,40E-02	1,36E+00	4,72E-02	1,731	Q9JHF7	HPGDS	hematopoietic prostaglandin D synthase	Cytoplasm	enzyme
9,75E-03	2,01E+00	2,92E-02	-1,238	P50172	HSD11B1	hydroxysteroid 11-beta dehydrogenase 1	Cytoplasm	enzyme
2,14E-02	1,67E+00	4,90E-02	-1,212	O35632	HYAL2	hyaluronidase 2	Cytoplasm	enzyme
3,19E-02	1,50E+00	6,03E-03	-2,648	P85094	ISOC2	isochorismatase domain containing 2	Cytoplasm	enzyme
3,39E-02	1,47E+00	3,29E-03	-1,267	Q3TFD2	LPCAT1	lysophosphatidylcholine acyltransferase 1	Cytoplasm	enzyme
2,56E-02	1,59E+00	9,68E-03	1,701	Q6NXK9	MAN1C1	mannosidase alpha class 1C member 1	Cytoplasm	enzyme
3,95E-02	1,40E+00	1,56E-02	1,505	Q8VCF0	MAVS	mitochondrial antiviral signaling protein	Cytoplasm	other
2,50E-02	1,60E+00	9,79E-03	-1,547	Q9CPU4	MGST3	microsomal glutathione S-transferase 3	Cytoplasm	enzyme
1,98E-02	1,70E+00	2,92E-02	1,472	Q8CD10	MICU2	mitochondrial calcium uptake 2	Cytoplasm	other
7,19E-04	3,14E+00	9,41E-03	1,585	Q99J99	MPST	mercaptopyruvate sulfurtransferase	Cytoplasm	enzyme
9,99E-03	2,00E+00	9,99E-03	-25	Q9DCI9	MRPL32	mitochondrial ribosomal protein L32	Cytoplasm	translation regulator
2,46E-02	1,61E+00	3,57E-02	-1,235	Q99N89	MRPL43	mitochondrial ribosomal protein L43	Cytoplasm	translation regulator
4,43E-02	1,35E+00	2,29E-02	-4,142	P13541	MYH3	myosin heavy chain 3	Cytoplasm	enzyme
4,38E-02	1,36E+00	2,77E-02	-1,223	Q8VDD5	MYH9	myosin heavy chain 9	Cytoplasm	enzyme
9,99E-03	2,00E+00	9,99E-03	25	Q6A037	N4BP1	NEDD4 binding protein 1	Cytoplasm	other
1,45E-02	1,84E+00	8,07E-03	-1,461	Q9DCS9	NDUFB10	NADH:ubiquinone oxidoreductase subunit B10	Cytoplasm	enzyme
4,10E-02	1,39E+00	1,13E-02	-1,442	Q9CPU2	NDUFB2	NADH:ubiquinone oxidoreductase subunit B2	Cytoplasm	enzyme
1,08E-03	2,97E+00	3,96E-02	1,733	Q9Z1J3	NFS1	NFS1 cysteine desulfurase	Cytoplasm	enzyme
2,53E-03	2,60E+00	1,31E-02	2,621	Q9DCJ9	NPL	N-acetylneuraminate pyruvate lyase	Cytoplasm	enzyme
9,99E-03	2,00E+00	9,99E-03	25	Q9CWD8	NUBPL	nucleotide binding protein like	Cytoplasm	other
2,62E-02	1,58E+00	2,08E-02	-1,286	Q505D7	OPA3	outer mitochondrial membrane lipid metabolism regulator OPA3	Cytoplasm	other
2,07E-02	1,68E+00	2,19E-02	1,475	A2A8Z1	OSBPL9	oxysterol binding protein like 9	Cytoplasm	other
2,62E-02	1,58E+00	1,24E-02	2,115	Q8C0L6	PAOX	polyamine oxidase	Cytoplasm	enzyme
1,39E-02	1,86E+00	3,76E-03	-1,206	Q91ZA3	PCCA	propionyl-CoA carboxylase subunit alpha	Cytoplasm	enzyme
2,00E-02	1,70E+00	2,19E-02	1,706	Q8K183	PDXK	pyridoxal kinase	Cytoplasm	kinase
4,31E-02	1,37E+00	3,83E-02	1,496	Q3UUQ7	PGAP1	post-GPI attachment to proteins inositol deacylase 1	Cytoplasm	enzyme
3,48E-04	3,46E+00	5,89E-04	-1,568	Q6ZPQ6	PITPNM2	phosphatidylinositol transfer protein membrane associated 2	Cytoplasm	enzyme
1,94E-03	2,71E+00	3,23E-03	-2,261	Q99KR7	PPIF	peptidylprolyl isomerase F	Cytoplasm	enzyme
5,81E-03	2,24E+00	3,16E-02	1,71	O88531	PPT1	palmitoyl-protein thioesterase 1	Cytoplasm	enzyme
5,68E-03	2,25E+00	3,82E-02	-1,245	Q61171	PRDX2	peroxiredoxin 2	Cytoplasm	enzyme
8,04E-03	2,09E+00	3,97E-02	2,737	Q922R0	PRKX	protein kinase X-linked	Cytoplasm	kinase
1,91E-02	1,72E+00	3,75E-02	-1,33	P22437	PTGS1	prostaglandin-endoperoxide synthase 1	Cytoplasm	enzyme
5,33E-03	2,27E+00	2,50E-03	-1,267	P35293	RAB18	RAB18, member RAS oncogene family	Cytoplasm	enzyme
2,81E-02	1,55E+00	1,73E-02	4,642	Q8VDV3	RAB3IL1	RAB3A interacting protein like 1	Cytoplasm	other
2,74E-02	1,56E+00	3,88E-02	1,455	Q62172	RALBP1	ralA binding protein 1	Cytoplasm	enzyme
2,50E-02	1,60E+00	4,86E-02	1,656	Q9WUB0	RBCK1	RANBP2-type and C3HC4-type zinc finger containing 1	Cytoplasm	transcription regulator
1,89E-02	1,72E+00	1,67E-02	2,068	Q9CQE5	RGS10	regulator of G protein signaling 10	Cytoplasm	enzyme
6,31E-04	3,20E+00	2,77E-03	-1,572	P63325	RPS10	ribosomal protein S10	Cytoplasm	other
3,23E-04	3,49E+00	3,52E-03	-1,394	P63276	RPS17	ribosomal protein S17	Cytoplasm	other
2,63E-02	1,58E+00	1,13E-02	1,347	P62274	RPS29	ribosomal protein S29	Cytoplasm	other
9,99E-03	2,00E+00	9,99E-03	25	Q8BLK9	RPS6KC1	ribosomal protein S6 kinase C1	Cytoplasm	kinase
1,01E-02	2,00E+00	2,44E-02	-1,23	O08547	SEC22B	SEC22 homolog B, vesicle trafficking protein (gene/pseudogene)	Cytoplasm	other
6,02E-04	3,22E+00	1,95E-03	1,892	Q8VE96	SLC35F6	solute carrier family 35 member F6	Cytoplasm	other
6,84E-03	2,16E+00	2,87E-02	2,056	Q9WU81	SLC37A2	solute carrier family 37 member 2	Cytoplasm	transporter
4,71E-02	1,33E+00	3,48E-02	1,421	Q8CFD4	SNX8	sorting nexin 8	Cytoplasm	transporter
7,96E-04	3,10E+00	3,40E-02	-1,391	P08228	SOD1	superoxide dismutase 1	Cytoplasm	enzyme
1,92E-02	1,72E+00	1,63E-02	-1,393	Q9D6K5	SYNJ2BP	synaptojanin 2 binding protein	Cytoplasm	other
6,44E-03	2,19E+00	2,11E-02	1,688	Q8BH24	TM9SF4	transmembrane 9 superfamily member 4	Cytoplasm	transporter
4,47E-02	1,35E+00	3,74E-02	1,833	Q9JLV2	TRPC4AP	transient receptor potential cation channel subfamily C member 4 associate	Cytoplasm	transporter
2,53E-03	2,60E+00	4,33E-02	-1,464	P50637	TSPO	translocator protein	Cytoplasm	transmembrane receptor
1,84E-03	2,74E+00	2,29E-02	1,219	Q9Z0P5	TWF2	twinfilin actin binding protein 2	Cytoplasm	kinase
9,99E-03	2,00E+00	9,99E-03	25	A2RSX7	TYW5	tRNA-yW synthesizing protein 5	Cytoplasm	enzyme
1,49E-02	1,83E+00	4,03E-02	1,31	Q9Z1F9	UBA2	ubiquitin like modifier activating enzyme 2	Cytoplasm	enzyme
7,87E-03	2,10E+00	6,98E-03	1,948	Q8VCW4	UNC93B1	unc-93 homolog B1, TLR signaling regulator	Cytoplasm	other
1,32E-02	1,88E+00	1,11E-02	-1,392	Q9CR68	UQCRFS1	ubiquinol-cytochrome c reductase, Rieske iron-sulfur polypeptide 1	Cytoplasm	enzyme
5,55E-03	2,26E+00	1,69E-02	-1,217	Q99LG0	USP16	ubiquitin specific peptidase 16	Cytoplasm	peptidase
7,82E-03	2,11E+00	9,64E-03	1,293	Q8R307	VPS18	VPS18 core subunit of CORVET and HOPS complexes	Cytoplasm	transporter
9,99E-03	2,00E+00	9,99E-03	25	Q9CR39	WDR45B	WD repeat domain 45B	Cytoplasm	other
1,52E-03	2,82E+00	3,03E-03	1,297	P56695	WFS1	wolframin ER transmembrane glycoprotein	Cytoplasm	enzyme
7,01E-03	2,15E+00	1,59E-02	1,388	Q8BZZ3	WWP1	WW domain containing E3 ubiquitin protein ligase 1	Cytoplasm	enzyme
2,80E-03	2,55E+00	1,42E-02	2,744	B2RW38	CFAP58	cilia and flagella associated protein 58	Extracellular Space	other
2,29E-03	2,64E+00	2,96E-03	-2,124	Q04857	COL6A1	collagen type VI alpha 1 chain	Extracellular Space	other
3,06E-03	2,51E+00	1,23E-03	-2,13	Q02788	COL6A2	collagen type VI alpha 2 chain	Extracellular Space	other
3,57E-02	1,45E+00	2,58E-03	-2,001	E9PWQ3	COL6A3	collagen type VI alpha 3 chain	Extracellular Space	other
1,23E-02	1,91E+00	1,12E-02	-1,482	Q99K41	EMILIN1	elastin microfibril interfacer 1	Extracellular Space	other
7,55E-03	2,12E+00	2,52E-02	-1,305	Q8BYW9	EOGT	EGF domain specific O-linked N-acetylglucosamine transferase	Extracellular Space	enzyme
2,60E-02	1,59E+00	3,96E-02	3,329	Q8BH61	F13A1	coagulation factor XIII A chain	Extracellular Space	enzyme
9,99E-03	2,00E+00	9,74E-04	-3,16	Q684R7	FREM1	FRAS1 related extracellular matrix 1	Extracellular Space	other
2,60E-02	1,59E+00	2,07E-02	-1,624	Q3TNL8	ITPRIP	inositol 1,4,5-trisphosphate receptor interacting protein	Extracellular Space	other
2,11E-02	1,68E+00	4,58E-02	-1,301	O88322	NID2	nidogen 2	Extracellular Space	other
4,94E-02	1,31E+00	2,25E-02	1,403	E1U8D0	SOGA1	suppressor of glucose, autophagy associated 1	Extracellular Space	other
4,93E-03	2,31E+00	4,11E-02	-2,192	Q9D809	2200002D01Rik	RIKEN cDNA 2200002D01 gene	Nucleus	other
9,99E-03	2,00E+00	9,99E-03	25	O70200	AIF1	allograft inflammatory factor 1	Nucleus	other
9,99E-03	2,00E+00	9,99E-03	25	Q3TSG4	ALKBH5	alkB homolog 5, RNA demethylase	Nucleus	enzyme
2,22E-02	1,65E+00	3,66E-02	1,314	E9Q6J5	BOD1L1	biorientation of chromosomes in cell division 1 like 1	Nucleus	other
9,99E-03	2,00E+00	9,99E-03	25	Q76KJ5	CD3EAP	CD3e molecule associated protein	Nucleus	other
2,10E-05	4,68E+00	2,55E-02	-1,208	P97315	CSRP1	cysteine and glycine rich protein 1	Nucleus	other
1,24E-02	1,91E+00	1,82E-02	-1,405	P97314	CSRP2	cysteine and glycine rich protein 2	Nucleus	other
3,66E-02	1,44E+00	3,66E-02	-1,736	Q6NZQ2	DDX31	DEAD-box helicase 31	Nucleus	enzyme

9,99E-03	2,00E+00	9,99E-03	-25	Q80VY9	DHX33	DEAH-box helicase 33	Nucleus	enzyme
9,99E-03	2,00E+00	9,99E-03	25	Q9CS74	ECD	ecdysoneless cell cycle regulator	Nucleus	transcription regulator
5,35E-04	3,27E+00	2,77E-03	1,262	P42128	FOXK1	forkhead box K1	Nucleus	transcription regulator
3,56E-02	1,45E+00	3,72E-02	2,661	O35601	FYB1	FYN binding protein 1	Nucleus	other
9,99E-03	2,00E+00	9,99E-03	25	Q99LZ3	GINS4	GINS complex subunit 4	Nucleus	other
4,00E-02	1,40E+00	8,21E-03	2,329	P0DOV2	IFI16	interferon gamma inducible protein 16	Nucleus	transcription regulator
2,32E-02	1,63E+00	2,32E-02	1,437	Q9D168	INTS12	integrator complex subunit 12	Nucleus	other
4,52E-02	1,34E+00	4,88E-02	-1,304	Q99PI5	LPIN2	lipin 2	Nucleus	phosphatase
9,99E-03	2,00E+00	9,99E-03	-25	Q5SWW4	MED13	mediator complex subunit 13	Nucleus	transcription regulator
6,69E-03	2,17E+00	1,96E-02	1,2	Q8K1R7	NEK9	NIMA related kinase 9	Nucleus	kinase
3,38E-02	1,47E+00	3,38E-02	1,555	Q02780	NFIA	nuclear factor I A	Nucleus	transcription regulator
2,23E-02	1,65E+00	1,29E-02	2,567	E9Q8I7	NFXL1	nuclear transcription factor, X-box binding like 1	Nucleus	transcription regulator
3,82E-02	1,42E+00	2,26E-02	-1,237	Q01768	NME2	NME/NM23 nucleoside diphosphate kinase 2	Nucleus	kinase
2,85E-02	1,55E+00	6,37E-03	-1,286	Q9WU28	PFDN5	prefoldin subunit 5	Nucleus	transcription regulator
9,99E-03	2,00E+00	9,99E-03	25	Q9CQT5	POMP	proteasome maturation protein	Nucleus	other
8,79E-03	2,06E+00	1,44E-03	1,897	Q9CXG3	PPIL4	peptidylprolyl isomerase like 4	Nucleus	enzyme
4,77E-02	1,32E+00	3,36E-02	1,367	Q8BX09	RBBP5	RB binding protein 5, histone lysine methyltransferase complex subunit	Nucleus	transcription regulator
4,49E-02	1,35E+00	1,41E-02	2,038	P97868	RBBP6	RB binding protein 6, ubiquitin ligase	Nucleus	enzyme
9,99E-03	2,00E+00	9,99E-03	-25	Q0VG62	RBIS	ribosomal biogenesis factor	Nucleus	other
3,59E-02	1,44E+00	3,38E-02	1,25	Q9JJT0	RCL1	RNA terminal phosphate cyclase like 1	Nucleus	enzyme
3,52E-02	1,45E+00	2,30E-02	-1,826	Q64374	RGN	regucalcin	Nucleus	enzyme
1,85E-02	1,73E+00	2,98E-02	-2,339	Q9CQ71	RPA3	replication protein A3	Nucleus	other
1,34E-02	1,87E+00	3,64E-02	-1,218	P35979	RPL12	ribosomal protein L12	Nucleus	other
2,82E-02	1,55E+00	1,96E-02	1,232	E9PWW9	RSF1	remodeling and spacing factor 1	Nucleus	transcription regulator
2,30E-02	1,64E+00	2,34E-02	2,438	Q99K95	RTF2	replication termination factor 2	Nucleus	other
6,64E-03	2,18E+00	1,82E-03	-1,273	Q5M8N4	SDR39U1	short chain dehydrogenase/reductase family 39U member 1	Nucleus	other
3,64E-02	1,44E+00	2,34E-02	1,845	Q3UHX9	SPOUT1	SPOUT domain containing methyltransferase 1	Nucleus	other
4,73E-02	1,33E+00	4,56E-02	-3,162	Q7TMY4	THOC7	THO complex 7	Nucleus	other
2,74E-03	2,56E+00	7,59E-03	2,019	Q8BU11	TOX4	TOX high mobility group box family member 4	Nucleus	other
9,99E-03	2,00E+00	9,99E-03	25	E9Q444	ZBTB21	zinc finger and BTB domain containing 21	Nucleus	transcription regulator
3,49E-02	1,46E+00	4,21E-02	-5,752	Q9JJN2	ZFHX4	zinc finger homeobox 4	Nucleus	transcription regulator
2,11E-02	1,68E+00	3,21E-02	1,699	Q8VC28	Akr1c12/Akr1c13	aldo-keto reductase family 1, member C13	Other	enzyme
1,62E-02	1,79E+00	1,24E-02	1,275	B2RXR6	ANKRD44	ankyrin repeat domain 44	Other	other
1,18E-02	1,93E+00	1,23E-02	1,298	A6H630	ARMT1	acidic residue methyltransferase 1	Other	enzyme
9,99E-03	2,00E+00	9,99E-03	-25	F6XLV1	CROCC2	ciliary rootlet coiled-coil, rootletin family member 2	Other	other
9,99E-03	2,00E+00	9,99E-03	25	Q3UE31	KIAA0930	KIAA0930	Other	other
1,47E-02	1,83E+00	6,14E-03	1,554	Q9DBS5	KLC4	kinesin light chain 4	Other	other
9,99E-03	2,00E+00	9,99E-03	25	Q9ERD6	RALGPS2	Ral GEF with PH domain and SH3 binding motif 2	Other	other
9,99E-03	2,00E+00	9,99E-03	25	D3Z0K6	RSBN1L	round spermatid basic protein 1 like	Other	other
9,99E-03	2,00E+00	9,99E-03	-25	Q8C3S2	TANGO6	transport and golgi organization 6 homolog	Other	other
9,99E-03	2,00E+00	9,99E-03	25	E9Q723	TTC13	tetratricopeptide repeat domain 13	Other	other
3,35E-02	1,47E+00	1,09E-03	-1,292	E9Q6P5	TTC7B	tetratricopeptide repeat domain 7B	Other	other
9,99E-03	2,00E+00	4,30E-02	-1,895	Q99JW1	ABHD17A	abhydrolase domain containing 17A, depalmitoylase	Plasma Membrane	enzyme
9,14E-05	4,04E+00	5,00E-02	-1,446	Q7M759	ABHD17B	abhydrolase domain containing 17B, depalmitoylase	Plasma Membrane	peptidase
1,80E-02	1,74E+00	4,95E-02	-1,38	P09470	ACE	angiotensin I converting enzyme	Plasma Membrane	peptidase
4,77E-02	1,32E+00	5,13E-03	-1,373	Q9CQW2	ARL8B	ADP ribosylation factor like GTPase 8B	Plasma Membrane	enzyme
2,06E-02	1,69E+00	3,20E-02	-1,571	Q9WVC3	CAV2	caveolin 2	Plasma Membrane	other
2,92E-03	2,53E+00	4,05E-02	-1,21	Q4PZA2	ECE1	endothelin converting enzyme 1	Plasma Membrane	peptidase
1,28E-02	1,89E+00	1,63E-02	1,248	Q03173	ENAH	ENAH actin regulator	Plasma Membrane	other
4,01E-02	1,40E+00	3,72E-02	-1,603	Q63961	ENG	endoglin	Plasma Membrane	transmembrane receptor
2,29E-02	1,64E+00	3,92E-02	2,035	Q9WV92	EPB41L3	erythrocyte membrane protein band 4.1 like 3	Plasma Membrane	other
9,99E-03	2,00E+00	9,99E-03	-25	Q8BGS1	EPB41L5	erythrocyte membrane protein band 4.1 like 5	Plasma Membrane	other
4,26E-03	2,37E+00	1,04E-02	-1,262	A2AKG8	FOCAD	focadhesin	Plasma Membrane	other
3,93E-03	2,41E+00	8,98E-03	-1,476	Q8BHL4	GPRC5A	G protein-coupled receptor class C group 5 member A	Plasma Membrane	G-protein coupled receptor
9,99E-03	2,00E+00	9,99E-03	25	P70387	HFE	homeostatic iron regulator	Plasma Membrane	transmembrane receptor
3,46E-02	1,46E+00	9,99E-03	-25	P20040	HLA-DRB5	major histocompatibility complex, class II, DR beta 5	Plasma Membrane	transmembrane receptor
1,08E-02	1,97E+00	2,43E-04	-1,464	P13597	ICAM1	intercellular adhesion molecule 1	Plasma Membrane	transmembrane receptor
9,99E-03	2,00E+00	9,99E-03	-25	P01872	IGHM	immunoglobulin heavy constant mu	Plasma Membrane	transmembrane receptor
2,00E-02	1,70E+00	7,18E-03	-1,34	Q62470	ITGA3	integrin subunit alpha 3	Plasma Membrane	other
5,91E-03	2,23E+00	3,03E-02	-1,635	B8JK39	ITGA9	integrin subunit alpha 9	Plasma Membrane	other
4,18E-02	1,38E+00	3,18E-02	1,301	Q6WVG3	KCTD12	potassium channel tetramerization domain containing 12	Plasma Membrane	ion channel
1,37E-02	1,86E+00	2,93E-02	2,165	Q01965	LY9	lymphocyte antigen 9	Plasma Membrane	other
9,99E-03	2,00E+00	9,99E-03	25	P08553	Nefm	neurofilament, medium polypeptide	Plasma Membrane	other
1,82E-03	2,74E+00	3,76E-02	-1,346	P18293	NPR1	natriuretic peptide receptor 1	Plasma Membrane	enzyme
1,35E-03	2,87E+00	1,42E-02	-1,917	Q61503	NT5E	5'-nucleotidase ecto	Plasma Membrane	phosphatase
4,73E-02	1,33E+00	3,85E-02	-1,241	P56380	NUDT2	nudix hydrolase 2	Plasma Membrane	phosphatase
3,15E-02	1,50E+00	1,59E-02	1,681	Q9JJX6	P2RX4	purinergic receptor P2X 4	Plasma Membrane	ion channel
1,91E-02	1,72E+00	2,94E-02	-1,587	Q9R0M4	Podxl	podocalyxin-like	Plasma Membrane	other
3,86E-03	2,41E+00	2,32E-03	1,224	Q6P2K6	PPP4R3A	protein phosphatase 4 regulatory subunit 3A	Plasma Membrane	other
2,57E-03	2,59E+00	7,55E-04	-2,277	P60060	SEC61G	SEC61 translocon subunit gamma	Plasma Membrane	transporter
2,79E-03	2,55E+00	3,37E-02	1,423	P53986	SLC16A1	solute carrier family 16 member 1	Plasma Membrane	transporter
1,06E-02	1,97E+00	1,66E-02	1,61	Q61609	SLC20A1	solute carrier family 20 member 1	Plasma Membrane	transporter
2,69E-02	1,57E+00	1,56E-02	2,286	O55100	SYNGR1	synaptogyrin 1	Plasma Membrane	transporter
4,52E-02	1,34E+00	4,26E-02	2,593	P36423	TBXAS1	thromboxane A synthase 1	Plasma Membrane	enzyme
1,94E-03	2,71E+00	4,73E-03	1,366	Q62351	TFRC	transferrin receptor	Plasma Membrane	transporter
1,25E-02	1,90E+00	2,06E-02	-1,401	Q06806	TIE1	tyrosine kinase with immunoglobulin like and EGF like domains 1	Plasma Membrane	kinase
5,53E-05	4,26E+00	1,04E-02	-1,476	Q9CQG9	TMEM100	transmembrane protein 100	Plasma Membrane	other
2,82E-02	1,55E+00	3,68E-02	-1,562	Q9CQN6	TMEM14C	transmembrane protein 14C	Plasma Membrane	other
9,26E-03	2,03E+00	2,62E-02	-1,503	P58771	Tpm1	tropomyosin 1, alpha	Plasma Membrane	other
9,99E-03	2,00E+00	9,99E-03	25	Q8BGB2	TTC7A	tetratricopeptide repeat domain 7A	Plasma Membrane	other
9,99E-03	2,00E+00	9,99E-03	25	Q3V0C5	USP48	ubiquitin specific peptidase 48	Plasma Membrane	peptidase
8,91E-04	3,05E+00	1,73E-02	1,3	Q5ND34	WDR81	WD repeat domain 81	Plasma Membrane	other
