# Supplementary material for: Autophagy protein 5 controls flow-dependent endothelial functions
Source: Cell Mol Life Sci. 2023 Jul 18;80(8):210. doi: 10.1007/s00018-023-04859-9 (PMC10352428; doi:10.1007/s00018-023-04859-9)
Supplement: Supplementary file 5 — Supplementary file5 (PDF 25 KB) [file 18_2023_4859_MOESM5_ESM.pdf]

Categories	Functions	Diseases or Functions Annotation	p-value	B-H p-value	Molecules	# Molecules
Cell Signaling,Post-Translational Modification	activation	Activation of MAP kinase	9.24E-03	1.53E-01	IGHM,PRDX2,SOD1	3
Cellular Assembly and Organization	assembly	Assembly of parallel actin bundles	3.06E-02	1.78E-01	AIF1	1
Small Molecule Biochemistry	biosynthesis	Biosynthesis of hydrogen sulfide	1.03E-02	1.53E-01	MPST	1
Nucleic Acid Metabolism,Small Molecule Biochemistry	biosynthesis	Biosynthesis of NADP	1.03E-02	1.53E-01	G6PD	1
Carbohydrate Metabolism	biosynthesis	Biosynthesis of pentose	2.05E-02	1.61E-01	G6PD	1
Cardiovascular System Development and Function,Hematological System Development and Function	blood pressure	Blood pressure	1.49E-02	1.61E-01	ACE,PTGS1,SOD1	3
DNA Replication, Recombination, and Repair,Nucleic Acid Metabolism,Small Molecule Biochemistry	catabolism	Catabolism of AMP	1.03E-02	1.53E-01	NT5E	1
Carbohydrate Metabolism	catabolism	Catabolism of carbohydrate	3.58E-03	1.53E-01	HEXB,HYAL2,NPL	3
Small Molecule Biochemistry	catabolism	Catabolism of carboxylic acid	1.61E-02	1.61E-01	HYAL2,NPL	2
Lipid Metabolism,Small Molecule Biochemistry	catabolism	Catabolism of galactosylceramide	2.05E-02	1.61E-01	GALC	1
Lipid Metabolism,Small Molecule Biochemistry	catabolism	Catabolism of ganglioside	3.06E-02	1.78E-01	HEXB	1
Lipid Metabolism,Small Molecule Biochemistry	catabolism	Catabolism of glycosphingolipid	7.66E-03	1.53E-01	GALC,HEXB	2
Endocrine System Development and Function,Small Molecule Biochemistry	catabolism	Catabolism of hormone	4.06E-02	1.90E-01	ACE	1
Carbohydrate Metabolism,Drug Metabolism,Small Molecule Biochemistry	catabolism	Catabolism of hyaluronic acid	3.06E-02	1.78E-01	HYAL2	1
Carbohydrate Metabolism,Small Molecule Biochemistry	catabolism	Catabolism of N-acetylneuraminic acid	4.06E-02	1.90E-01	NPL	1
Carbohydrate Metabolism	catabolism	Catabolism of oligosaccharide	1.55E-03	1.45E-01	HEXB,NPL	2
Small Molecule Biochemistry	catabolism	Catabolism of polyamines	3.06E-02	1.78E-01	PAOX	1
Cardiovascular System Development and Function,Organ Development,Organ Morphology	contraction	Contraction of heart	3.20E-02	1.78E-01	ACE,SOD1,Tpm1	3
Post-Translational Modification	cross-linkage	Cross-linkage of peptide	4.06E-02	1.90E-01	F13A1	1
Post-Translational Modification	depalmitoylation	Depalmitoylation of protein	2.85E-03	1.52E-01	ABHD17B,PPT1	2
Cell Death and Survival	ferroptosis	Ferroptosis	1.03E-02	1.53E-01	GPX4	1
Cellular Assembly and Organization,Cellular Function and Maintenance	formation	Formation of caveolae	3.06E-02	1.78E-01	CAV2	1
Cell-To-Cell Signaling and Interaction,Cellular Assembly and Organization,Cellular Function and Maintenance	formation	Formation of focal adhesions	2.72E-02	1.78E-01	DUSP3,EPB41L5	2
Cellular Assembly and Organization,Cellular Function and Maintenance,Tissue Development	formation	Formation of intermediate filaments	4.06E-02	1.90E-01	Nefm	1
Cell-To-Cell Signaling and Interaction,Cellular Assembly and Organization	fusion	Fusion of plasma membrane	3.06E-02	1.78E-01	TIE1	1
Cellular Function and Maintenance,Molecular Transport,Small Molecule Biochemistry	homeostasis	Homeostasis of iron ion	5.00E-04	1.33E-01	HFE,SOD1,TFRC,TTC7A	4
Cellular Function and Maintenance,Small Molecule Biochemistry	homeostasis	Homeostasis of metal ion	1.78E-02	1.61E-01	HEXB,HFE,SOD1,TFRC,TTC7A	5
Cell Cycle	localization	Localization of meiotic spindles	4.06E-02	1.90E-01	MYH9	1
Carbohydrate Metabolism	metabolism	Metabolism of carbohydrate	1.27E-02	1.61E-01	G6PD,GNPNAT1,GUSB,HEXB,HYAL2,NPL,RGN	7
Drug Metabolism,Protein Synthesis	metabolism	Metabolism of glutathione	1.78E-02	1.61E-01	G6PD,SOD1	2
Carbohydrate Metabolism,Small Molecule Biochemistry	metabolism	Metabolism of glycosaminoglycan	1.78E-02	1.61E-01	HEXB,HYAL2	2
Endocrine System Development and Function,Small Molecule Biochemistry	metabolism	Metabolism of hormone	2.17E-03	1.45E-01	ACE,CTSB,ECE1,HFE	4
Free Radical Scavenging,Small Molecule Biochemistry	metabolism	Metabolism of hydrogen peroxide	1.30E-02	1.61E-01	PRDX2,SOD1	2
Lipid Metabolism,Small Molecule Biochemistry	metabolism	Metabolism of prostaglandin	1.61E-02	1.61E-01	HPGDS,PTGS1	2
Cellular Assembly and Organization	nuclear import	Nuclear import	4.06E-02	1.90E-01	RAB18	1
Cellular Assembly and Organization,Cellular Function and Maintenance	organization	Organization of lysosome	3.14E-02	1.78E-01	HEXB,PPT1	2
Cellular Assembly and Organization	organization	Organization of organelle	1.35E-02	1.61E-01	CAV2,ENAH,GPX4,HEXB,MYH9,Nefm,PPT1,SOD1,VPS18,WDR81	10
Cell Morphology,Cellular Assembly and Organization,Cellular Function and Maintenance	permeability	Permeability of mitochondrial membrane	3.06E-02	1.78E-01	PPIF	1
Free Radical Scavenging	removal	Removal of superoxide	2.15E-03	1.45E-01	PRDX2,SOD1	2
DNA Replication, Recombination, and Repair,Nucleic Acid Metabolism,Small Molecule Biochemistry	synthesis	Synthesis of adenosine	1.03E-02	1.53E-01	NT5E	1
Carbohydrate Metabolism,Small Molecule Biochemistry,Vitamin and Mineral Metabolism	synthesis	Synthesis of ascorbic acid	3.06E-02	1.78E-01	RGN	1
Endocrine System Development and Function,Small Molecule Biochemistry	synthesis	Synthesis of hormone	1.30E-02	1.61E-01	CTSB,HFE	2
Carbohydrate Metabolism,Nucleic Acid Metabolism,Small Molecule Biochemistry	synthesis	Synthesis of UDP-N-acetylglucosamine	2.05E-02	1.61E-01	GNPNAT1	1
Molecular Transport	transport	Transport of metal	4.92E-02	2.11E-01	HFE,MICU2,P2RX4,SOD1,TFRC,TTC7A	6
