# Supplementary material for: Autophagy protein 5 controls flow-dependent endothelial functions
Source: Cell Mol Life Sci. 2023 Jul 18;80(8):210. doi: 10.1007/s00018-023-04859-9 (PMC10352428; doi:10.1007/s00018-023-04859-9)
Supplement: Supplementary file 6 — Supplementary file6 (PDF 181 KB) [file 18_2023_4859_MOESM6_ESM.pdf]

Expr p-value	-Log(p-value)	Expr FDR (q-value)	Expr Fold Change	In GFP+ Only	ID	Symbol	Entrez Gene Name	Location	Type(s)	Other localization
4,31E-03	2,37E+00	1,11E-01	2,389	0	P58742	AAAS	aladin WD repeat nucleoporin	Nucleus	other	
5,67E-04	3,25E+00	5,56E-02	5,563	0	Q9JKX4	AATF	apoptosis antagonizing transcription factor	Nucleus	transcription regulator	
1,04E-04	3,98E+00	3,05E-02	8,795	0	Q8R2Y0	ABHD6	abhydrolase domain containing 6, acylglycerol lipase	Cytoplasm	enzyme	mitochondria
9,99E-03	2,00E+00	9,99E-03	35	1	P56485	ACKR3	atypical chemokine receptor 3	Plasma Membrane	G-protein coupled receptor	
4,84E-05	4,32E+00	3,05E-02	2,24	0	Q91V92	ACLY	ATP citrate lyase	Cytoplasm	enzyme	mitochondria
9,99E-03	2,00E+00	9,99E-03	35	1	P54987	ACOD1	aconitate decarboxylase 1	Cytoplasm	enzyme	mitochondria
5,55E-03	2,26E+00	1,18E-01	3,672	0	Q9CZW4	ACSL3	acyl-CoA synthetase long chain family member 3	Cytoplasm	enzyme	mitochondria
9,99E-03	2,00E+00	9,99E-03	35	1	Q61271	ACVR1B	activin A receptor type 1B	Plasma Membrane	kinase	
1,48E-03	2,83E+00	8,06E-02	4,302	0	Q61824	ADAM12	ADAM metallopeptidase domain 12	Plasma Membrane	peptidase	
1,21E-03	2,92E+00	7,86E-02	13,926	0	P97857	ADAMTS1	ADAM metallopeptidase with thrombospondin type 1 motif 1	Extracellular Space	peptidase	
9,99E-03	2,00E+00	9,99E-03	35	1	Q91ZS8	ADARB1	adenosine deaminase RNA specific B1	Nucleus	enzyme	
2,72E-03	2,57E+00	9,42E-02	2,016	0	G5E8Q8	ADGRF5	adhesion G protein-coupled receptor F5	Plasma Membrane	G-protein coupled receptor	
7,77E-03	2,11E+00	1,40E-01	5,638	0	O08715	AKAP1	A-kinase anchoring protein 1	Cytoplasm	other	mitochondria
1,44E-03	2,84E+00	8,06E-02	3,034	0	Q9Z110	ALDH18A1	aldehyde dehydrogenase 18 family member A1	Cytoplasm	kinase	mitochondria
1,22E-03	2,91E+00	7,86E-02	2,176	0	O08583	Alyref	Aly/REF export factor	Nucleus	other	
4,57E-03	2,34E+00	1,12E-01	5,136	0	Q9JHZ2	ANKH	ANKH inorganic pyrophosphate transport regulator	Plasma Membrane	transporter	
3,35E-03	2,47E+00	1,02E-01	10,626	0	Q9CZ52	ANTXR1	ANTXR cell adhesion molecule 1	Plasma Membrane	transmembrane receptor	
4,34E-03	2,36E+00	1,11E-01	4,923	0	Q06335	APLP2	amyloid beta precursor like protein 2	Cytoplasm	other	
9,75E-04	3,01E+00	7,40E-02	2,232	0	P12023	APP	amyloid beta precursor protein	Plasma Membrane	other	mitochondria
9,99E-03	2,00E+00	9,99E-03	35	1	Q9D0J4	ARL2	ADP ribosylation factor like GTPase 2	Cytoplasm	enzyme	mitochondria
9,99E-03	2,00E+00	9,99E-03	35	1	Q6A058	ARMCX2	armadillo repeat containing X-linked 2	Other	other	mitochondria
2,82E-03	2,55E+00	9,42E-02	2,373	0	Q8BSY0	ASPH	aspartate beta-hydroxylase	Cytoplasm	enzyme	
3,92E-03	2,41E+00	1,08E-01	4,782	0	Q8R087	B4GALT7	beta-1,4-galactosyltransferase 7	Cytoplasm	enzyme	
5,00E-03	2,30E+00	1,15E-01	2,475	0	Q8K019	BCLAF1	BCL2 associated transcription factor 1	Nucleus	transcription regulator	
9,99E-03	2,00E+00	9,99E-03	35	1	O35153	BET1L	Bet1 golgi vesicular membrane trafficking protein like	Cytoplasm	transporter	
6,73E-04	3,17E+00	6,30E-02	3,08	0	O35607	BMPR2	bone morphogenetic protein receptor type 2	Plasma Membrane	kinase	
3,38E-05	4,47E+00	2,60E-02	5,676	0	Q8R2Q8	Bst2	bone marrow stromal cell antigen 2	Plasma Membrane	other	
4,46E-03	2,35E+00	1,11E-01	2,422	0	Q810Q5	C15orf48	chromosome 15 open reading frame 48	Nucleus	other	
1,06E-04	3,97E+00	3,05E-02	9,472	0	Q8K201	C5orf15	chromosome 5 open reading frame 15	Other	other	
9,99E-03	2,00E+00	9,99E-03	35	1	Q9CXL3	C7orf50	chromosome 7 open reading frame 50	Other	other	
5,51E-03	2,26E+00	1,18E-01	5,009	0	Q8CGU1	CALCOCO1	calcium binding and coiled-coil domain 1	Nucleus	transcription regulator	
9,99E-03	2,00E+00	9,99E-03	35	1	Q61686	CBX5	chromobox 5	Nucleus	transcription regulator	
2,00E-03	2,70E+00	8,66E-02	2,09	0	Q8CH18	CCAR1	cell division cycle and apoptosis regulator 1	Nucleus	transcription regulator	
9,99E-03	2,00E+00	9,99E-03	35	1	P10148	Ccl2	chemokine (C-C motif) ligand 2	Extracellular Space	cytokine	
1,63E-03	2,79E+00	8,06E-02	2,893	0	P29268	CCN2	cellular communication network factor 2	Extracellular Space	growth factor	
9,99E-03	2,00E+00	9,99E-03	35	1	O88874	CCNK	cyclin K	Nucleus	kinase	
9,99E-03	2,00E+00	9,99E-03	35	1	Q76KJ5	CD3EAP	CD3e molecule associated protein	Nucleus	other	
2,16E-03	2,67E+00	8,66E-02	2,447	0	Q6A068	CDC5L	cell division cycle 5 like	Nucleus	transcription regulator	
1,35E-04	3,87E+00	3,42E-02	4,54	0	P55284	CDH5	cadherin 5	Plasma Membrane	other	
9,63E-03	2,02E+00	1,44E-01	2,268	0	Q04899	CDK18	cyclin dependent kinase 18	Cytoplasm	kinase	
3,47E-03	2,46E+00	1,03E-01	2,303	0	Q6PDQ2	CHD4	chromodomain helicase DNA binding protein 4	Nucleus	enzyme	
9,99E-03	2,00E+00	9,99E-03	35	1	Q99LI9	CLP1	cleavage factor polyribonucleotide kinase subunit 1	Nucleus	kinase	
4,36E-03	2,36E+00	1,11E-01	4,306	0	Q9D486	CMIP	c-Maf inducing protein	Cytoplasm	other	
6,97E-03	2,16E+00	1,34E-01	2,071	0	Q8BMA3	CNKSR3	CNKSR family member 3	Plasma Membrane	kinase	
2,44E-03	2,61E+00	9,20E-02	2,837	0	P39061	COL18A1	collagen type XVIII alpha 1 chain	Extracellular Space	other	
4,94E-03	2,31E+00	1,15E-01	2,795	0	P08122	COL4A2	collagen type IV alpha 2 chain	Extracellular Space	other	
1,77E-05	4,75E+00	1,92E-02	5,272	0	Q8K4Q8	COLEC12	collectin subfamily member 12	Plasma Membrane	transmembrane receptor	
2,12E-03	2,67E+00	8,66E-02	2,354	0	Q9QXK7	CPSF3	cleavage and polyadenylation specific factor 3	Nucleus	enzyme	
2,17E-03	2,66E+00	8,66E-02	3,595	0	Q6NVF9	CPSF6	cleavage and polyadenylation specific factor 6	Nucleus	other	
9,99E-03	2,00E+00	9,99E-03	35	1	Q8BTV2	CPSF7	cleavage and polyadenylation specific factor 7	Nucleus	other	

**9,99E-03**	**2,00E+00**	**9,99E-03**	**35**	**1**	**Q01147**	**CREB1**	**cAMP responsive element binding protein 1**	**Nucleus**	**transcription regulator**	mitochondria
**6,41E-05**	**4,19E+00**	**3,05E-02**	**5,422**	**0**	**Q9JLL0**	**CRIM1**	**cysteine rich transmembrane BMP regulator 1**	**Extracellular Space**	**kinase**	
7,89E-04	3,10E+00	6,98E-02	2,404	0	Q8BIQ5	CSTF2	cleavage stimulation factor subunit 2	Nucleus	other	
**9,99E-03**	**2,00E+00**	**9,99E-03**	**35**	**1**	**Q8VIH7**	**CYYR1**	**cysteine and tyrosine rich 1**	**Other**	**other**	
1,63E-03	2,79E+00	8,06E-02	2,133	0	Q8BPM0	DAAM1	dishevelled associated activator of morphogenesis 1	Cytoplasm	other	
9,62E-03	2,02E+00	1,44E-01	5,251	0	Q91ZV3	DCBLD2	discoidin, CUB and LCCL domain containing 2	Plasma Membrane	other	
5,12E-03	2,29E+00	1,15E-01	2,358	0	Q501J6	DDX17	DEAD-box helicase 17	Nucleus	enzyme	
2,58E-03	2,59E+00	9,41E-02	5,058	0	Q8K363	DDX18	DEAD-box helicase 18	Nucleus	enzyme	
**2,93E-04**	**3,53E+00**	**4,36E-02**	**3,814**	**0**	**Q9JIK5**	**DDX21**	**DExD-box helicase 21**	**Nucleus**	**enzyme**	mitochondria
**9,99E-03**	**2,00E+00**	**9,99E-03**	**35**	**1**	**Q9ESV0**	**DDX24**	**DEAD-box helicase 24**	**Nucleus**	**enzyme**	
8,60E-04	3,07E+00	7,21E-02	5,158	0	Q8VDW0	DDX39A	DExD-box helicase 39A	Nucleus	enzyme	
7,03E-03	2,15E+00	1,34E-01	5,674	0	Q810A7	DDX42	DEAD-box helicase 42	Cytoplasm	enzyme	
1,66E-03	2,78E+00	8,15E-02	2,808	0	Q61656	DDX5	DEAD-box helicase 5	Nucleus	enzyme	
2,42E-03	2,62E+00	9,18E-02	2,057	0	Q8VCH6	DHCR24	24-dehydrocholesterol reductase	Cytoplasm	enzyme	
**9,99E-03**	**2,00E+00**	**9,99E-03**	**35**	**1**	**Q99KU1**	**DHDDS**	**dehydrodolichyl diphosphate synthase subunit**	**Cytoplasm**	**enzyme**	
3,08E-03	2,51E+00	9,86E-02	2,154	0	O35286	DHX15	DEAH-box helicase 15	Nucleus	enzyme	
**4,33E-04**	**3,36E+00**	**4,97E-02**	**9,371**	**0**	**Q9ESX5**	**DKC1**	**dyskerin pseudouridine synthase 1**	**Nucleus**	**enzyme**	
1,61E-03	2,79E+00	8,06E-02	3,023	0	Q9QYI5	DNAJB2	DnaJ heat shock protein family (Hsp40) member B2	Nucleus	other	
8,86E-03	2,05E+00	1,42E-01	2,796	0	Q80TN4	DNAJC16	DnaJ heat shock protein family (Hsp40) member C16	Other	other	
**9,99E-03**	**2,00E+00**	**9,99E-03**	**35**	**1**	**Q8R3P2**	**DTX2**	**deltex E3 ubiquitin ligase 2**	**Nucleus**	**other**	
5,09E-04	3,29E+00	5,34E-02	5,329	0	Q4PZA2	ECE1	endothelin converting enzyme 1	Plasma Membrane	peptidase	
**9,99E-03**	**2,00E+00**	**9,99E-03**	**35**	**1**	**P22387**	**EDN1**	**endothelin 1**	**Extracellular Space**	**cytokine**	
4,64E-03	2,33E+00	1,13E-01	2,997	0	P57776	EEF1D	eukaryotic translation elongation factor 1 delta	Cytoplasm	translation regulator	
5,34E-04	3,27E+00	5,43E-02	2,169	0	Q91VC3	EIF4A3	eukaryotic translation initiation factor 4A3	Nucleus	enzyme	
1,77E-03	2,75E+00	8,24E-02	2,328	0	Q03145	EPHA2	EPH receptor A2	Plasma Membrane	kinase	
4,24E-03	2,37E+00	1,11E-01	5,666	0	P54761	EPHB4	EPH receptor B4	Plasma Membrane	kinase	
**9,99E-03**	**2,00E+00**	**9,99E-03**	**35**	**1**	**P81270**	**ERG**	**ETS transcription factor ERG**	**Nucleus**	**transcription regulator**	
3,02E-03	2,52E+00	9,82E-02	2,27	0	Q9ERY9	ERG28	ergosterol biosynthesis 28 homolog	Cytoplasm	other	
**8,87E-05**	**4,05E+00**	**3,05E-02**	**3,011**	**0**	**P84089**	**ERH**	**ERH mRNA splicing and mitosis factor**	**Nucleus**	**other**	
2,78E-03	2,56E+00	9,42E-02	2,044	0	Q61545	Ewsr1	Ewing sarcoma breakpoint region 1	Nucleus	other	
8,56E-03	2,07E+00	1,42E-01	2,01	0	Q8C163	EXOG	exo/endonuclease G	Cytoplasm	enzyme	mitochondria
7,83E-03	2,11E+00	1,40E-01	3,545	0	Q9CRA8	EXOSC5	exosome component 5	Nucleus	enzyme	
9,01E-03	2,05E+00	1,42E-01	3,135	0	P70428	EXT2	exostosin glycosyltransferase 2	Cytoplasm	enzyme	
**3,58E-04**	**3,45E+00**	**4,78E-02**	**5,182**	**0**	**Q922J9**	**FAR1**	**fatty acyl-CoA reductase 1**	**Cytoplasm**	**enzyme**	
9,61E-03	2,02E+00	1,44E-01	4,262	0	P35550	FBL	fibrillarin	Nucleus	enzyme	
**9,99E-03**	**2,00E+00**	**9,99E-03**	**35**	**1**	**P20491**	**FCER1G**	**Fc fragment of IgE receptor Ig**	**Plasma Membrane**	**transmembrane receptor**	
8,82E-04	3,05E+00	7,21E-02	14,359	0	P16092	FGFR1	fibroblast growth factor receptor 1	Plasma Membrane	kinase	
1,62E-03	2,79E+00	8,06E-02	7,22	0	Q9D824	Fip1l1	FIP1 like 1 (S. cerevisiae)	Nucleus	other	
7,87E-03	2,10E+00	1,40E-01	2,866	0	Q8BLU0	FLRT2	fibronectin leucine rich transmembrane protein 2	Plasma Membrane	other	
2,65E-03	2,58E+00	9,41E-02	9,504	0	P35969	FLT1	fms related receptor tyrosine kinase 1	Plasma Membrane	kinase	
1,07E-03	2,97E+00	7,50E-02	4,508	0	P35917	FLT4	fms related receptor tyrosine kinase 4	Plasma Membrane	transmembrane receptor	
9,83E-03	2,01E+00	1,45E-01	2,436	0	P11276	FN1	fibronectin 1	Extracellular Space	enzyme	
**9,99E-03**	**2,00E+00**	**9,99E-03**	**35**	**1**	**P47930**	**FOSL2**	**FOS like 2, AP-1 transcription factor subunit**	**Nucleus**	**transcription regulator**	
9,77E-03	2,01E+00	1,45E-01	2,461	0	Q9DBE9	FTSJ3	FtsJ RNA 2'-O-methyltransferase 3	Nucleus	enzyme	
5,31E-03	2,27E+00	1,17E-01	2,039	0	Q9D6K8	FUNDC2	FUN14 domain containing 2	Cytoplasm	other	mitochondria
**3,95E-04**	**3,40E+00**	**4,78E-02**	**2,689**	**0**	**P60521**	**GABARAPL2**	**GABA type A receptor associated protein like 2**	**Cytoplasm**	**other**	autophagosome
2,13E-03	2,67E+00	8,66E-02	2,144	0	Q9CY66	Gar1	GAR1 ribonucleoprotein	Nucleus	other	
2,00E-03	2,70E+00	8,66E-02	4,395	0	Q8CHY6	GATAD2A	GATA zinc finger domain containing 2A	Nucleus	transcription regulator	
2,37E-03	2,63E+00	9,06E-02	3,729	0	Q8VHR5	GATAD2B	GATA zinc finger domain containing 2B	Nucleus	transcription regulator	
9,23E-03	2,03E+00	1,42E-01	2,089	0	Q9QYC7	GGCX	gamma-glutamyl carboxylase	Cytoplasm	enzyme	
**9,99E-03**	**2,00E+00**	**9,99E-03**	**35**	**1**	**Q9D7M1**	**GID8**	**GID complex subunit 8 homolog**	**Nucleus**	**other**	

3,16E-03	2,50E+00	9,99E-02	8,424	0	P23242	GJA1	gap junction protein alpha 1	Plasma Membrane	transporter	mitochondria
1,93E-03	2,71E+00	8,66E-02	2,085	0	Q61543	GLG1	golgi glycoprotein 1	Cytoplasm	other	
9,99E-03	2,00E+00	9,99E-03	35	1	Q6NSU3	GLT8D1	glycosyltransferase 8 domain containing 1	Cytoplasm	enzyme	
9,99E-03	2,00E+00	9,99E-03	35	1	Q3UED7	Gm4951	predicted gene 4951	Other	other	
8,94E-03	2,05E+00	1,42E-01	2,335	0	Q921M4	GOLGA2	golgin A2	Cytoplasm	other	
1,98E-03	2,70E+00	8,66E-02	2,61	0	Q9QYE6	GOLGA5	golgin A5	Cytoplasm	kinase	
6,75E-03	2,17E+00	1,32E-01	2,2	0	O88630	GOSR1	golgi SNAP receptor complex member 1	Cytoplasm	transporter	
4,13E-03	2,38E+00	1,11E-01	2,416	0	Q9WTK3	GPAA1	glycosylphosphatidylinositol anchor attachment 1	Cytoplasm	enzyme	mitochondria
9,99E-03	2,00E+00	9,99E-03	35	1	Q8BUV8	GPR107	G protein-coupled receptor 107	Plasma Membrane	G-protein coupled receptor	
1,00E-03	3,00E+00	7,41E-02	2,519	0	Q923Z0	GPRC5B	G protein-coupled receptor class C group 5 member B	Plasma Membrane	G-protein coupled receptor	
7,87E-03	2,10E+00	1,40E-01	4,593	0	Q9CX99	GRAP	GRB2 related adaptor protein	Cytoplasm	other	
8,95E-03	2,05E+00	1,42E-01	3,683	0	Gvin1	Gvin1	GTPase, very large interferon inducible 1	Nucleus	other	
9,99E-03	2,00E+00	9,99E-03	35	1	P70288	HDAC2	histone deacetylase 2	Nucleus	transcription regulator	
8,04E-04	3,09E+00	7,00E-02	2,439	0	G3X9B1	HEATR1	HEAT repeat containing 1	Nucleus	other	mitochondria
9,99E-03	2,00E+00	9,99E-03	35	1	Q9DC33	HMG20A	high mobility group 20A	Nucleus	transcription regulator	
1,95E-03	2,71E+00	8,66E-02	2,855	0	Hmga1	HMGA1	high mobility group AT-hook 1	Nucleus	transcription regulator	
5,08E-03	2,29E+00	1,15E-01	5,152	0	P52927	Hmga2	high mobility group AT-hook 2	Nucleus	enzyme	
4,19E-03	2,38E+00	1,11E-01	4,467	0	P49312	Hnrnpa1	heterogeneous nuclear ribonucleoprotein A1	Nucleus	other	
6,96E-03	2,16E+00	1,34E-01	2,393	0	O88569	HNRNPA2B1	heterogeneous nuclear ribonucleoprotein A2/B1	Nucleus	other	
8,52E-03	2,07E+00	1,42E-01	3,127	0	Q8BG05	Hnrnpa3	heterogeneous nuclear ribonucleoprotein A3	Nucleus	transporter	
1,62E-03	2,79E+00	8,06E-02	2,713	0	Q9Z204	HNRNPC	heterogeneous nuclear ribonucleoprotein C	Nucleus	other	
4,32E-03	2,36E+00	1,11E-01	2,023	0	Q60668	HNRNPD	heterogeneous nuclear ribonucleoprotein D	Nucleus	transcription regulator	
9,19E-03	2,04E+00	1,42E-01	3,037	0	O35737	HNRNPH1	heterogeneous nuclear ribonucleoprotein H1	Nucleus	other	
6,14E-03	2,21E+00	1,28E-01	2,111	0	Q8R081	HNRNPL	heterogeneous nuclear ribonucleoprotein L	Nucleus	other	
3,98E-03	2,40E+00	1,08E-01	2,488	0	Q9D0E1	HNRNPM	heterogeneous nuclear ribonucleoprotein M	Nucleus	other	
8,36E-03	2,08E+00	1,42E-01	2,697	0	Q8VEK3	HNRNPU	heterogeneous nuclear ribonucleoprotein U	Nucleus	transporter	
8,70E-03	2,06E+00	1,42E-01	3,027	0	O88736	HSD17B7	hydroxysteroid 17-beta dehydrogenase 7	Cytoplasm	enzyme	
9,99E-03	2,00E+00	9,99E-03	35	1	Q8R5F7	IFIH1	interferon induced with helicase C domain 1	Nucleus	enzyme	mitochondria
1,25E-03	2,90E+00	7,96E-02	8,343	0	Q64282	IFIT1B	interferon induced protein with tetratricopeptide repeats 1B	Cytoplasm	other	
2,09E-03	2,68E+00	8,66E-02	3,793	0	Q99J93	IFITM2	interferon induced transmembrane protein 2	Cytoplasm	other	
8,23E-03	2,08E+00	1,42E-01	4,07	0	Q9CQW9	IFITM3	interferon induced transmembrane protein 3	Plasma Membrane	other	
9,99E-03	2,00E+00	9,99E-03	35	1	P33896	IFNAR1	interferon alpha and beta receptor subunit 1	Plasma Membrane	transmembrane receptor	
9,99E-03	2,00E+00	9,99E-03	35	1	P47879	IGFBP4	insulin like growth factor binding protein 4	Extracellular Space	other	
4,02E-03	2,40E+00	1,08E-01	2,65	0	Q61581	IGFBP7	insulin like growth factor binding protein 7	Extracellular Space	transporter	
2,66E-03	2,58E+00	9,41E-02	2,556	0	Q9Z1M8	IK	IK cytokine	Extracellular Space	cytokine	
8,99E-03	2,05E+00	1,42E-01	2,321	0	Q9DBZ1	IKBIP	IKBKB interacting protein	Cytoplasm	other	
1,12E-03	2,95E+00	7,66E-02	3,184	0	Q61730	IL1RAP	interleukin 1 receptor accessory protein	Plasma Membrane	transmembrane receptor	
5,02E-04	3,30E+00	5,34E-02	12,764	0	P34902	IL2RG	interleukin 2 receptor subunit gamma	Plasma Membrane	transmembrane receptor	
1,41E-03	2,85E+00	8,06E-02	3,353	0	P16382	IL4R	interleukin 4 receptor	Plasma Membrane	transmembrane receptor	
4,90E-03	2,31E+00	1,15E-01	3,506	0	Q9Z1X4	ILF3	interleukin enhancer binding factor 3	Nucleus	transcription regulator	mitochondria
9,99E-03	2,00E+00	9,99E-03	35	1	Q8VHZ7	IMP4	IMP U3 small nucleolar ribonucleoprotein 4	Nucleus	other	
9,99E-03	2,00E+00	9,99E-03	35	1	Q8QZV7	INTS13	integrator complex subunit 13	Cytoplasm	other	
9,99E-03	2,00E+00	9,99E-03	35	1	Q80UK8	INTS2	integrator complex subunit 2	Nucleus	other	
9,99E-03	2,00E+00	9,99E-03	35	1	Q7TPD0	INTS3	integrator complex subunit 3	Nucleus	other	
9,99E-03	2,00E+00	9,99E-03	35	1	Q8CHT3	INTS5	integrator complex subunit 5	Nucleus	other	
9,99E-03	2,00E+00	9,99E-03	35	1	Q6PCM2	INTS6	integrator complex subunit 6	Nucleus	enzyme	
2,10E-04	3,68E+00	3,91E-02	6,142	0	O89051	ITM2B	integral membrane protein 2B	Plasma Membrane	other	
1,44E-05	4,84E+00	1,92E-02	7,926	0	Q91VK4	ITM2C	integral membrane protein 2C	Cytoplasm	other	
1,68E-03	2,77E+00	8,15E-02	6,423	0	Q3UV16	ITPRIPL2	ITPRIP like 2	Other	other	
8,98E-03	2,05E+00	1,42E-01	3,523	0	Q9QXX0	JAG1	jagged canonical Notch ligand 1	Extracellular Space	growth factor	
9,99E-03	2,00E+00	9,99E-03	35	1	P09450	JUNB	JunB proto-oncogene, AP-1 transcription factor subunit	Nucleus	transcription regulator	

1,42E-04	3,85E+00	3,42E-02	16,684	0	P35918	KDR	kinase insert domain receptor	Plasma Membrane	kinase	
8,15E-03	2,09E+00	1,41E-01	3,028	0	Q9Z2X8	KEAP1	kelch like ECH associated protein 1	Cytoplasm	transcription regulator	
7,19E-03	2,14E+00	1,35E-01	2,208	0	Q3U0V1	KHSRP	KH-type splicing regulatory protein	Nucleus	enzyme	
1,98E-04	3,70E+00	3,91E-02	3,086	0	Q91X21	KIAA2013	KIAA2013	Other	other	
7,38E-03	2,13E+00	1,36E-01	3,587	0	Q80W68	KIRREL1	kirre like nephrin family adhesion molecule 1	Plasma Membrane	other	
8,37E-06	5,08E+00	1,92E-02	2,585	0	P97927	LAMA4	laminin subunit alpha 4	Extracellular Space	enzyme	
2,64E-03	2,58E+00	9,41E-02	5,036	0	Q60961	LAPTM4A	lysosomal protein transmembrane 4 alpha	Cytoplasm	other	
9,33E-03	2,03E+00	1,43E-01	2,095	0	Q6A0A2	LARP4B	La ribonucleoprotein 4B	Cytoplasm	other	
6,25E-03	2,20E+00	1,29E-01	4,702	0	P35951	LDLR	low density lipoprotein receptor	Plasma Membrane	transporter	
9,99E-03	2,00E+00	9,99E-03	35	1	Q9WU40	LEMD3	LEM domain containing 3	Nucleus	other	
2,10E-03	2,68E+00	8,66E-02	2,758	0	Q9JL15	LGALS8	galectin 8	Extracellular Space	other	
9,99E-03	2,00E+00	9,99E-03	35	1	Q7TQH7	LRP10	LDL receptor related protein 10	Plasma Membrane	transmembrane receptor	
9,99E-03	2,00E+00	9,99E-03	35	1	Q8BUJ9	LRP12	LDL receptor related protein 12	Plasma Membrane	transmembrane receptor	
9,99E-03	2,00E+00	9,99E-03	35	1	Q924X6	LRP8	LDL receptor related protein 8	Plasma Membrane	transmembrane receptor	
7,51E-03	2,12E+00	1,37E-01	4,809	0	G3XA59	LRRC32	leucine rich repeat containing 32	Plasma Membrane	other	
9,99E-03	2,00E+00	9,99E-03	35	1	Q08288	LYAR	Ly1 antibody reactive	Plasma Membrane	other	
3,90E-04	3,41E+00	4,78E-02	3,49	0	Q9CQV6	MAP1LC3B	microtubule associated protein 1 light chain 3 beta	Cytoplasm	other	autophagosome
9,99E-03	2,00E+00	9,99E-03	35	1	Q9Z2D8	MBD3	methyl-CpG binding domain protein 3	Nucleus	other	
9,99E-03	2,00E+00	9,99E-03	35	1	Q8VCD5	MED17	mediator complex subunit 17	Nucleus	transcription regulator	
8,46E-05	4,07E+00	3,05E-02	5,485	0	Q922T2	MFAP3	microfibril associated protein 3	Extracellular Space	other	
8,62E-03	2,06E+00	1,42E-01	2,203	0	Q6PCP5	Mff	mitochondrial fission factor	Cytoplasm	other	mitochondria
2,81E-03	2,55E+00	9,42E-02	5,325	0	P27808	MGAT1	alpha-1,3-mannosyl-glycoprotein 2-beta-N-acetylglucosaminyltransferase	Cytoplasm	enzyme	
1,60E-03	2,80E+00	8,06E-02	3,328	0	Q921V5	MGAT2	alpha-1,6-mannosyl-glycoprotein 2-beta-N-acetylglucosaminyltransferase	Cytoplasm	enzyme	
1,52E-04	3,82E+00	3,42E-02	8,662	0	Q812F8	MGAT4B	alpha-1,3-mannosyl-glycoprotein 4-beta-N-acetylglucosaminyltransferase B	Cytoplasm	enzyme	
9,41E-04	3,03E+00	7,29E-02	2,594	0	P19788	MGP	matrix Gla protein	Extracellular Space	other	
7,89E-03	2,10E+00	1,40E-01	2,389	0	Q8VDV8	MITD1	microtubule interacting and trafficking domain containing 1	Cytoplasm	other	
2,68E-05	4,57E+00	2,41E-02	7,107	0	P53690	MMP14	matrix metallopeptidase 14	Extracellular Space	peptidase	
5,54E-03	2,26E+00	1,18E-01	2,795	0	A6H6E2	MMRN2	multimerin 2	Extracellular Space	other	
9,99E-03	2,00E+00	9,99E-03	35	1	Q810V0	MPHOSPH10	M-phase phosphoprotein 10	Nucleus	other	
2,30E-03	2,64E+00	8,88E-02	2,651	0	Q9JLB0	MPP6	membrane palmitoylated protein 6	Plasma Membrane	kinase	
2,10E-03	2,68E+00	8,66E-02	5,267	0	Q3TEW6	MPZL1	myelin protein zero like 1	Plasma Membrane	other	
9,33E-04	3,03E+00	7,29E-02	4,308	0	Q9CPR5	MRPL15	mitochondrial ribosomal protein L15	Cytoplasm	other	mitochondria
9,99E-03	2,00E+00	9,99E-03	35	1	Q9CPX7	MRPS16	mitochondrial ribosomal protein S16	Cytoplasm	other	mitochondria
8,83E-03	2,05E+00	1,42E-01	2,024	0	Q8VE22	MRPS23	mitochondrial ribosomal protein S23	Cytoplasm	other	mitochondria
9,99E-03	2,00E+00	9,99E-03	35	1	Q9CY16	MRPS28	mitochondrial ribosomal protein S28	Cytoplasm	other	mitochondria
3,97E-03	2,40E+00	1,08E-01	2,295	0	Q9D0I8	MRTO4	MRT4 homolog, ribosome maturation factor	Cytoplasm	other	
9,99E-03	2,00E+00	9,99E-03	35	1	Q9CRA4	MSMO1	methylsterol monooxygenase 1	Cytoplasm	enzyme	
3,46E-03	2,46E+00	1,03E-01	2,868	0	Q8K4B0	MTA1	metastasis associated 1	Nucleus	transcription regulator	
7,20E-03	2,14E+00	1,35E-01	2,499	0	Q9R190	MTA2	metastasis associated 1 family member 2	Nucleus	transcription regulator	
4,78E-03	2,32E+00	1,14E-01	3,355	0	Q9CZU3	MTREX	Mtr4 exosome RNA helicase	Nucleus	enzyme	
8,38E-05	4,08E+00	3,05E-02	9,383	0	Q9DBV4	MXRA8	matrix remodeling associated 8	Cytoplasm	other	
5,63E-03	2,25E+00	1,19E-01	2,512	0	Q7TPV4	MYBBP1A	MYB binding protein 1a	Nucleus	transcription regulator	
9,61E-06	5,02E+00	1,92E-02	2,614	0	Q8R411	MYCT1	MYC target 1	Nucleus	other	
6,37E-03	2,20E+00	1,30E-01	2,669	0	Q8C854	MYEF2	myelin expression factor 2	Nucleus	transcription regulator	
5,39E-03	2,27E+00	1,17E-01	9,43	0	Q8K224	NAT10	N-acetyltransferase 10	Nucleus	enzyme	
2,46E-03	2,61E+00	9,20E-02	4,695	0	P97432	NBR1	NBR1 autophagy cargo receptor	Cytoplasm	other	autophagosome
1,44E-03	2,84E+00	8,06E-02	2,391	0	P09405	NCL	nucleolin	Nucleus	other	
1,96E-04	3,71E+00	3,91E-02	3,256	0	Q5U4H9	NCOA4	nuclear receptor coactivator 4	Nucleus	transcription regulator	mitochondria
3,98E-04	3,40E+00	4,78E-02	3,184	0	Q91W39	NCOA5	nuclear receptor coactivator 5	Nucleus	other	
2,40E-04	3,62E+00	4,17E-02	5,451	0	Q8R0W6	NDFIP1	Nedd4 family interacting protein 1	Cytoplasm	other	mitochondria
5,61E-05	4,25E+00	3,05E-02	7,778	0	Q91ZP6	NDFIP2	Nedd4 family interacting protein 2	Cytoplasm	other	

**9,99E-03**	**2,00E+00**	**9,99E-03**	**35**	**1**	**P25233**	**NDN**	**necdin, MAGE family member**	**Nucleus**	**transcription regulator**	
2,78E-03	2,56E+00	9,42E-02	3,756	0	Q9CRB2	NHP2	NHP2 ribonucleoprotein	Nucleus	other	
8,56E-03	2,07E+00	1,42E-01	2,694	0	P10493	NID1	nidogen 1	Extracellular Space	other	
6,42E-03	2,19E+00	1,30E-01	4,635	0	Q9CXK8	NIP7	nucleolar pre-rRNA processing protein NIP7	Nucleus	other	
**9,99E-03**	**2,00E+00**	**9,99E-03**	**35**	**1**	**Q8R5K4**	**NOL6**	**nucleolar protein 6**	**Nucleus**	**other**	mitochondria
3,38E-03	2,47E+00	1,02E-01	5,046	0	Q9CQS2	NOP10	NOP10 ribonucleoprotein	Nucleus	other	
9,03E-03	2,04E+00	1,42E-01	4,081	0	Q01705	NOTCH1	notch receptor 1	Plasma Membrane	transcription regulator	
6,98E-03	2,16E+00	1,34E-01	3,852	0	O35516	NOTCH2	notch receptor 2	Plasma Membrane	transcription regulator	
8,69E-04	3,06E+00	7,21E-02	2,55	0	Q61937	NPM1	nucleophosmin 1	Nucleus	transcription regulator	
**9,99E-03**	**2,00E+00**	**9,99E-03**	**35**	**1**	**Q62443**	**NPTX1**	**neuronal pentraxin 1**	**Extracellular Space**	**other**	mitochondria
**2,99E-04**	**3,52E+00**	**4,36E-02**	**4,723**	**0**	**O35375**	**NRP2**	**neuropilin 2**	**Plasma Membrane**	**kinase**	
**9,99E-03**	**2,00E+00**	**9,99E-03**	**35**	**1**	**Q62092**	**NSG1**	**neuronal vesicle trafficking associated 1**	**Cytoplasm**	**other**	
3,16E-03	2,50E+00	9,99E-02	2,561	0	Q91X76	NT5DC2	5'-nucleotidase domain containing 2	Cytoplasm	other	
**9,99E-03**	**2,00E+00**	**9,99E-03**	**35**	**1**	**O09118**	**NTN1**	**netrin 1**	**Extracellular Space**	**growth factor**	
1,03E-03	2,99E+00	7,50E-02	2,194	0	Q9JI33	NTN4	netrin 4	Extracellular Space	other	
2,74E-03	2,56E+00	9,42E-02	2,686	0	Q8BH74	NUP107	nucleoporin 107	Nucleus	other	
6,42E-03	2,19E+00	1,30E-01	3,825	0	Q8R4R6	NUP35	nucleoporin 35	Nucleus	transporter	
9,49E-03	2,02E+00	1,44E-01	2,236	0	Q8CEC0	NUP88	nucleoporin 88	Nucleus	transporter	
9,45E-04	3,02E+00	7,29E-02	4,005	0	Q6PFD9	NUP98	nucleoporin 98 and 96 precursor	Nucleus	transporter	
4,47E-03	2,35E+00	1,11E-01	4,264	0	Q9DBY8	NVL	nuclear VCP like	Nucleus	other	
5,10E-03	2,29E+00	1,15E-01	2,274	0	Q8BWG9	ORAI1	ORAI calcium release-activated calcium modulator 1	Plasma Membrane	ion channel	
9,55E-03	2,02E+00	1,44E-01	3,878	0	Q8CCS6	PABPN1	poly(A) binding protein nuclear 1	Nucleus	enzyme	
9,12E-03	2,04E+00	1,42E-01	2,899	0	Q99JB8	PACSIN3	protein kinase C and casein kinase substrate in neurons 3	Cytoplasm	other	
9,63E-03	2,02E+00	1,44E-01	4,934	0	Q9DCE5	PAK1IP1	PAK1 interacting protein 1	Nucleus	other	
**4,26E-04**	**3,37E+00**	**4,97E-02**	**14,691**	**0**	**P97467**	**PAM**	**peptidylglycine alpha-amidating monooxygenase**	**Plasma Membrane**	**enzyme**	
**9,99E-03**	**2,00E+00**	**9,99E-03**	**35**	**1**	**O55134**	**PCDH12**	**protocadherin 12**	**Plasma Membrane**	**other**	
**9,99E-03**	**2,00E+00**	**9,99E-03**	**35**	**1**	**E9PXF0**	**PCDH17**	**protocadherin 17**	**Plasma Membrane**	**other**	
**9,99E-03**	**2,00E+00**	**9,99E-03**	**35**	**1**	**Q91VD8**	**PCDHB16**	**protocadherin beta 16**	**Other**	**other**	
3,92E-03	2,41E+00	1,08E-01	4,82	0	Q91XX1	PCDHGC3	protocadherin gamma subfamily C, 3	Plasma Membrane	other	
5,54E-04	3,26E+00	5,53E-02	3,842	0	Q6NS46	PDCD11	programmed cell death 11	Nucleus	other	
4,56E-03	2,34E+00	1,12E-01	3,271	0	Q9DBD5	PELP1	proline, glutamate and leucine rich protein 1	Nucleus	other	
5,18E-03	2,29E+00	1,15E-01	2,435	0	Q9R0A0	PEX14	peroxisomal biogenesis factor 14	Cytoplasm	transcription regulator	
**9,99E-03**	**2,00E+00**	**9,99E-03**	**35**	**1**	**B2RQG2**	**PHF3**	**PHD finger protein 3**	**Nucleus**	**other**	
**3,90E-04**	**3,41E+00**	**4,78E-02**	**3,262**	**0**	**P83870**	**PHF5A**	**PHD finger protein 5A**	**Nucleus**	**other**	
1,72E-03	2,76E+00	8,22E-02	5,786	0	Q8CBQ5	PI4K2B	phosphatidylinositol 4-kinase type 2 beta	Cytoplasm	kinase	
7,17E-03	2,14E+00	1,35E-01	2,615	0	Q9QZC7	PLEKHB2	pleckstrin homology domain containing B2	Other	other	
9,52E-03	2,02E+00	1,44E-01	2,888	0	Q91VC4	PLVAP	plasmalemma vesicle associated protein	Plasma Membrane	other	
7,56E-03	2,12E+00	1,38E-01	2,646	0	Q80UG2	PLXNA4	plexin A4	Plasma Membrane	transmembrane receptor	
8,92E-03	2,05E+00	1,42E-01	3,696	0	Q8BG81	POLDIP3	DNA polymerase delta interacting protein 3	Nucleus	other	
**9,99E-03**	**2,00E+00**	**9,99E-03**	**35**	**1**	**P04095**	**Prl2c2**	**prolactin family 2, subfamily c, member 2**	**Extracellular Space**	**growth factor**	
8,91E-03	2,05E+00	1,42E-01	5,062	0	Q08761	PROS1	protein S	Extracellular Space	other	
4,24E-03	2,37E+00	1,11E-01	2,463	0	Q922U1	PRPF3	pre-mRNA processing factor 3	Nucleus	other	
3,21E-03	2,49E+00	1,01E-01	5,312	0	Q9DAW6	PRPF4	pre-mRNA processing factor 4	Nucleus	other	
5,02E-03	2,30E+00	1,15E-01	3,691	0	Q9R1C7	PRPF40A	pre-mRNA processing factor 40 homolog A	Nucleus	other	
2,03E-03	2,69E+00	8,66E-02	4,167	0	P17225	PTBP1	polypyrimidine tract binding protein 1	Nucleus	enzyme	
**2,00E-04**	**3,70E+00**	**3,91E-02**	**2,519**	**0**	**B2RU80**	**PTPRB**	**protein tyrosine phosphatase receptor type B**	**Plasma Membrane**	**phosphatase**	
7,82E-03	2,11E+00	1,40E-01	2,263	0	Q922Q4	PYCR2	pyrroline-5-carboxylate reductase 2	Cytoplasm	enzyme	mitochondria
7,37E-03	2,13E+00	1,36E-01	2,005	0	P70388	RAD50	RAD50 double strand break repair protein	Nucleus	enzyme	
1,17E-03	2,93E+00	7,78E-02	3,089	0	Q9CW46	RAVER1	ribonucleoprotein, PTB binding 1	Nucleus	other	
7,88E-03	2,10E+00	1,40E-01	4,722	0	Q60973	RBBP7	RB binding protein 7, chromatin remodeling factor	Nucleus	transcription regulator	
2,66E-03	2,58E+00	9,41E-02	9,814	0	Q8BP71	RBFOX2	RNA binding fox-1 homolog 2	Nucleus	transcription regulator	

3,84E-03	2,42E+00	1,08E-01	2,733	0	Q8C2Q3	RBM14	RNA binding motif protein 14	Nucleus	transcription regulator	
8,71E-03	2,06E+00	1,42E-01	5,534	0	Q8JZX4	RBM17	RNA binding motif protein 17	Nucleus	other	
**9,99E-03**	**2,00E+00**	**9,99E-03**	**35**	**1**	**Q8R3C6**	**RBM19**	**RNA binding motif protein 19**	**Nucleus**	**other**	
3,27E-03	2,49E+00	1,01E-01	11,024	0	Q8CGC6	RBM28	RNA binding motif protein 28	Nucleus	other	
1,33E-03	2,88E+00	8,06E-02	3,974	0	Q9CWZ3	RBM8A	RNA binding motif protein 8A	Nucleus	other	
1,79E-03	2,75E+00	8,24E-02	3,493	0	Q9WV02	RBMX	RNA binding motif protein X-linked	Nucleus	other	
1,08E-03	2,97E+00	7,50E-02	3,213	0	Q8VE37	RCC1	regulator of chromosome condensation 1	Cytoplasm	other	
**2,08E-04**	**3,68E+00**	**3,91E-02**	**2,731**	**0**	**Q9QYF1**	**RDH11**	**retinol dehydrogenase 11**	**Cytoplasm**	**enzyme**	
5,24E-03	2,28E+00	1,16E-01	3,678	0	Q8BYK4	RDH12	retinol dehydrogenase 12	Cytoplasm	enzyme	
**9,99E-03**	**2,00E+00**	**9,99E-03**	**35**	**1**	**Q60841**	**RELN**	**reelin**	**Extracellular Space**	**peptidase**	
**9,99E-03**	**2,00E+00**	**9,99E-03**	**35**	**1**	**Q9JJF3**	**RIOX1**	**ribosomal oxygenase 1**	**Nucleus**	**enzyme**	
**9,99E-03**	**2,00E+00**	**9,99E-03**	**35**	**1**	**Q3UJU9**	**RMDN3**	**regulator of microtubule dynamics 3**	**Cytoplasm**	**other**	mitochondria
3,53E-03	2,45E+00	1,04E-01	3,198	0	E9Q555	RNF213	ring finger protein 213	Cytoplasm	enzyme	
**9,99E-03**	**2,00E+00**	**9,99E-03**	**35**	**1**	**Q3U319**	**RNF40**	**ring finger protein 40**	**Cytoplasm**	**enzyme**	
3,71E-03	2,43E+00	1,08E-01	4,05	0	Q8C310	ROBO4	roundabout guidance receptor 4	Plasma Membrane	other	
9,19E-03	2,04E+00	1,42E-01	2,229	0	P19253	RPL13A	ribosomal protein L13a	Cytoplasm	other	
5,99E-03	2,22E+00	1,25E-01	2,839	0	Q9CPR4	RPL17	ribosomal protein L17	Cytoplasm	other	
4,60E-03	2,34E+00	1,12E-01	2,614	0	P62717	RPL18A	ribosomal protein L18a	Cytoplasm	other	
3,29E-03	2,48E+00	1,02E-01	2,249	0	P61358	RPL27	ribosomal protein L27	Cytoplasm	other	
1,51E-03	2,82E+00	8,06E-02	3,527	0	P14115	RPL27A	ribosomal protein L27a	Cytoplasm	other	
6,49E-03	2,19E+00	1,30E-01	2,6	0	P62911	Rpl32	ribosomal protein L32	Cytoplasm	other	
2,54E-03	2,60E+00	9,38E-02	3,702	0	Q6ZWV7	RPL35	ribosomal protein L35	Cytoplasm	other	
3,09E-03	2,51E+00	9,86E-02	2,568	0	O55142	RPL35A	ribosomal protein L35a	Cytoplasm	other	mitochondria
8,93E-03	2,05E+00	1,42E-01	2,927	0	P47964	Rpl36	ribosomal protein L36	Nucleus	other	
3,34E-03	2,48E+00	1,02E-01	3,34	0	P47962	RPL5	ribosomal protein L5	Cytoplasm	other	
**9,99E-03**	**2,00E+00**	**9,99E-03**	**35**	**1**	**Q9D8M4**	**RPL7L1**	**ribosomal protein L7 like 1**	**Cytoplasm**	**transcription regulator**	
6,56E-03	2,18E+00	1,30E-01	2,168	0	P51410	RPL9	ribosomal protein L9	Nucleus	other	
3,97E-03	2,40E+00	1,08E-01	2,178	0	P62281	RPS11	ribosomal protein S11	Cytoplasm	other	
6,53E-03	2,19E+00	1,30E-01	3,234	0	Q6ZWY3	RPS27L	ribosomal protein S27 like	Cytoplasm	translation regulator	
**9,99E-03**	**2,00E+00**	**9,99E-03**	**35**	**1**	**Q9CYX7**	**RRP15**	**ribosomal RNA processing 15 homolog**	**Nucleus**	**other**	
**9,99E-03**	**2,00E+00**	**9,99E-03**	**35**	**1**	**Q91YK2**	**RRP1B**	**ribosomal RNA processing 1B**	**Nucleus**	**transcription regulator**	
**9,99E-03**	**2,00E+00**	**9,99E-03**	**35**	**1**	**Q9DB85**	**RRP8**	**ribosomal RNA processing 8**	**Nucleus**	**enzyme**	
**1,51E-04**	**3,82E+00**	**3,42E-02**	**6,928**	**0**	**Q91WM3**	**RRP9**	**ribosomal RNA processing 9, U3 small nucleolar RNA binding protein**	**Nucleus**	**other**	
6,51E-03	2,19E+00	1,30E-01	2,134	0	Q99LF4	RTCB	RNA 2',3'-cyclic phosphate and 5'-OH ligase	Cytoplasm	enzyme	
9,87E-04	3,01E+00	7,40E-02	2,789	0	D3YXK2	SAFB	scaffold attachment factor B	Nucleus	other	
2,76E-03	2,56E+00	9,42E-02	2,065	0	Q9D1J3	SARNP	SAP domain containing ribonucleoprotein	Nucleus	transcription regulator	
6,77E-04	3,17E+00	6,30E-02	3,733	0	Q5ND28	SCARF1	scavenger receptor class F member 1	Plasma Membrane	transmembrane receptor	
3,37E-03	2,47E+00	1,02E-01	17,823	0	P13516	SCD	stearoyl-CoA desaturase	Cytoplasm	enzyme	
9,79E-03	2,01E+00	1,45E-01	2,866	0	Q8VCL2	SCO2	synthesis of cytochrome C oxidase 2	Cytoplasm	enzyme	mitochondria
5,14E-04	3,29E+00	5,34E-02	2,889	0	O08992	SDCBP	syndecan binding protein	Plasma Membrane	enzyme	mitochondria
**2,73E-04**	**3,56E+00**	**4,32E-02**	**4,868**	**0**	**Q99JZ0**	**SDCBP2**	**syndecan binding protein 2**	**Cytoplasm**	**other**	
1,39E-03	2,86E+00	8,06E-02	17,757	0	Q01102	SELP	selectin P	Plasma Membrane	transmembrane receptor	
**9,99E-03**	**2,00E+00**	**9,99E-03**	**35**	**1**	**O88632**	**SEMA3F**	**semaphorin 3F**	**Extracellular Space**	**other**	
6,92E-03	2,16E+00	1,34E-01	4,089	0	Q9QUR8	SEMA7A	semaphorin 7A (John Milton Hagen blood group)	Plasma Membrane	transmembrane receptor	
1,62E-03	2,79E+00	8,06E-02	4,844	0	Q9CY58	SERBP1	SERPINE1 mRNA binding protein 1	Cytoplasm	other	
4,96E-03	2,30E+00	1,15E-01	6,676	0	Q9QZI8	SERINC1	serine incorporator 1	Plasma Membrane	transporter	
5,52E-03	2,26E+00	1,18E-01	4,438	0	Q9QZI9	SERINC3	serine incorporator 3	Cytoplasm	transporter	
3,81E-03	2,42E+00	1,08E-01	2,51	0	Q62203	Sf3a2	splicing factor 3a, subunit 2	Nucleus	other	
4,66E-03	2,33E+00	1,13E-01	2,684	0	Q9D554	SF3A3	splicing factor 3a subunit 3	Nucleus	other	
**3,42E-04**	**3,47E+00**	**4,74E-02**	**2,185**	**0**	**Q99NB9**	**SF3B1**	**splicing factor 3b subunit 1**	**Nucleus**	**other**	
**9,32E-05**	**4,03E+00**	**3,05E-02**	**4,042**	**0**	**Q3UJB0**	**SF3B2**	**splicing factor 3b subunit 2**	**Nucleus**	**other**	

9,63E-03	2,02E+00	1,44E-01	2,369	0	Q8VIJ6	SFPQ	splicing factor proline and glutamine rich	Nucleus	other	
5,06E-03	2,30E+00	1,15E-01	2,675	0	O70258	SGCE	sarcoglycan epsilon	Plasma Membrane	other	
9,99E-03	2,00E+00	9,99E-03	35	1	Q8VCQ6	SGMS1	sphingomyelin synthase 1	Cytoplasm	enzyme	
6,69E-03	2,17E+00	1,31E-01	5,936	0	P98083	SHC1	SHC adaptor protein 1	Cytoplasm	other	mitochondria
6,90E-04	3,16E+00	6,31E-02	4,168	0	Q9CZN7	SHMT2	serine hydroxymethyltransferase 2	Cytoplasm	enzyme	mitochondria
7,69E-03	3,11E+00	6,92E-02	8,282	0	Q61609	SLC20A1	solute carrier family 20 member 1	Plasma Membrane	transporter	
9,99E-03	2,00E+00	9,99E-03	35	1	O35149	SLC30A4	solute carrier family 30 member 4	Cytoplasm	transporter	
2,70E-04	3,57E+00	4,32E-02	12,939	0	Q91ZN5	SLC35B2	solute carrier family 35 member B2	Cytoplasm	transporter	
1,44E-04	3,84E+00	3,42E-02	4,063	0	Q5I012	SLC38A10	solute carrier family 38 member 10	Cytoplasm	other	
4,98E-03	2,30E+00	1,15E-01	2,18	0	Q9D8T7	SLIRP	SRA stem-loop interacting RNA binding protein	Cytoplasm	other	mitochondria
1,27E-03	2,90E+00	8,00E-02	2,513	0	Q91ZW3	SMARCA5	SWI/SNF related, matrix associated, actin dependent regulator of chromatin, subfamily a, member 5	Nucleus	transcription regulator	
6,63E-03	2,18E+00	1,31E-01	5,443	0	O54941	SMARCE1	SWI/SNF related, matrix associated, actin dependent regulator of chromatin, subfamily e, member 1	Nucleus	transcription regulator	
3,67E-03	2,44E+00	1,07E-01	2,159	0	Q8BGT7	SMNDC1	survival motor neuron domain containing 1	Nucleus	other	
1,51E-03	2,82E+00	8,06E-02	8,807	0	Q3UKJ7	SMU1	SMU1 DNA replication regulator and spliceosomal factor	Nucleus	other	
2,88E-03	2,54E+00	9,52E-02	2,182	0	P57784	SNRPA1	small nuclear ribonucleoprotein polypeptide A'	Nucleus	other	
3,23E-03	2,49E+00	1,01E-01	2,203	0	P62317	SNRPD2	small nuclear ribonucleoprotein D2 polypeptide	Nucleus	other	
4,47E-03	2,35E+00	1,11E-01	2,366	0	P62320	SNRPD3	small nuclear ribonucleoprotein D3 polypeptide	Nucleus	other	
3,95E-04	3,40E+00	4,78E-02	4,123	0	Q9D0T1	SNU13	small nuclear ribonucleoprotein 13	Nucleus	other	
6,57E-04	3,18E+00	6,30E-02	2,63	0	Q9CSN1	SNW1	SNW domain containing 1	Nucleus	transcription regulator	
1,72E-03	2,76E+00	8,22E-02	3,03	0	Q9QX47	SON	SON DNA and RNA binding protein	Nucleus	other	
2,00E-03	2,70E+00	8,66E-02	2,963	0	P07214	SPARC	secreted protein acidic and cysteine rich	Extracellular Space	other	
1,32E-03	2,88E+00	8,06E-02	3,299	0	Q64337	SQSTM1	sequestosome 1	Cytoplasm	transcription regulator	autophagosome
7,40E-03	2,13E+00	1,36E-01	3,636	0	O70551	SRPK1	SRSF protein kinase 1	Nucleus	kinase	
1,92E-03	2,72E+00	8,66E-02	3,011	0	Q9DBG7	SRPRA	SRP receptor subunit alpha	Cytoplasm	other	
3,25E-04	3,49E+00	4,62E-02	3,336	0	Q8BTI8	Srrm2	serine/arginine repetitive matrix 2	Nucleus	other	
5,01E-03	2,30E+00	1,15E-01	5,35	0	Q9R0U0	SRSF10	serine and arginine rich splicing factor 10	Nucleus	other	
9,99E-03	2,00E+00	9,99E-03	35	1	Q64692	ST8SIA4	ST8 alpha-N-acetyl-neuraminide alpha-2,8-sialyltransferase 4	Cytoplasm	enzyme	
2,80E-04	3,55E+00	4,32E-02	4,047	0	Q8K1E0	STX5	syntaxin 5	Cytoplasm	transporter	
1,43E-03	2,84E+00	8,06E-02	2,822	0	P63166	SUMO1	small ubiquitin like modifier 1	Nucleus	enzyme	
9,99E-03	2,00E+00	9,99E-03	35	1	P70279	SURF6	surfeit 6	Nucleus	other	
9,99E-03	2,00E+00	9,99E-03	35	1	Q9D198	SYF2	SYF2 pre-mRNA splicing factor	Nucleus	other	
2,14E-03	2,67E+00	8,66E-02	2,262	0	Q8K0Z7	TACO1	translational activator of cytochrome c oxidase I	Cytoplasm	other	mitochondria
1,35E-03	2,87E+00	8,06E-02	2,693	0	Q921F2	TARDBP	TAR DNA binding protein	Nucleus	transcription regulator	mitochondria
5,22E-03	2,28E+00	1,16E-01	4,591	0	Q3UKC1	TAX1BP1	Tax1 binding protein 1	Cytoplasm	other	mitophagy
8,19E-05	4,09E+00	3,05E-02	2,437	0	Q8BYH7	TBC1D17	TBC1 domain family member 17	Cytoplasm	other	mitophagy
8,45E-03	2,07E+00	1,42E-01	2,664	0	Q9WUN2	TBK1	TANK binding kinase 1	Cytoplasm	kinase	mitophagy
1,74E-03	2,76E+00	8,24E-02	2,367	0	Q8CGF7	TCERG1	transcription elongation regulator 1	Nucleus	transcription regulator	
9,87E-03	2,01E+00	1,45E-01	7,327	0	O88968	TCN2	transcobalamin 2	Extracellular Space	transporter	
6,70E-03	2,17E+00	1,31E-01	2,646	0	O08784	TCOF1	treacle ribosome biogenesis factor 1	Nucleus	transporter	
9,99E-03	2,00E+00	9,99E-03	35	1	Q5SSK3	TEFM	transcription elongation factor, mitochondrial	Cytoplasm	transcription regulator	mitochondria
9,99E-03	2,00E+00	9,99E-03	35	1	P27090	TGFB2	transforming growth factor beta 2	Extracellular Space	growth factor	
5,92E-03	2,23E+00	1,24E-01	8,197	0	Q62312	TGFBR2	transforming growth factor beta receptor 2	Plasma Membrane	kinase	
4,21E-03	2,38E+00	1,11E-01	2,143	0	P35441	THBS1	thrombospondin 1	Extracellular Space	other	
9,99E-03	2,00E+00	9,99E-03	35	1	Q03350	THBS2	thrombospondin 2	Extracellular Space	other	
5,33E-03	2,27E+00	1,17E-01	3,3	0	Q8R3N6	THOC1	THO complex 1	Nucleus	transcription regulator	
9,99E-03	2,00E+00	9,99E-03	35	1	Q7TMY4	THOC7	THO complex 7	Nucleus	other	
1,08E-03	2,97E+00	7,50E-02	2,748	0	Q569Z6	THRAP3	thyroid hormone receptor associated protein 3	Nucleus	transcription regulator	
1,17E-05	4,93E+00	1,92E-02	5,459	0	P39876	TIMP3	TIMP metallopeptidase inhibitor 3	Extracellular Space	peptidase	
2,95E-03	2,53E+00	9,66E-02	3,543	0	Q99JR5	TINAGL1	tubulointerstitial nephritis antigen like 1	Extracellular Space	transporter	
2,30E-03	2,64E+00	8,88E-02	5,3	0	Q9DBU0	TM9SF1	transmembrane 9 superfamily member 1	Plasma Membrane	transporter	
3,78E-03	2,42E+00	1,08E-01	2,311	0	Q3V009	TMED1	transmembrane p24 trafficking protein 1	Extracellular Space	transporter	

**9,99E-03**	**2,00E+00**	**9,99E-03**	**35**	**1**	**Q9WUH1**	**Tmem115**	**transmembrane protein 115**	**Other**	**other**	
**9,99E-03**	**2,00E+00**	**9,99E-03**	**35**	**1**	**Q8BGP5**	**TMEM127**	**transmembrane protein 127**	**Other**	**other**	
**9,99E-03**	**2,00E+00**	**9,99E-03**	**35**	**1**	**Q9D938**	**TMEM160**	**transmembrane protein 160**	**Cytoplasm**	**other**	mitochondria
**9,99E-03**	**2,00E+00**	**9,99E-03**	**35**	**1**	**Q8VCA6**	**TMEM161A**	**transmembrane protein 161A**	**Other**	**other**	
1,59E-03	2,80E+00	8,06E-02	2,54	0	Q9DCS1	TMEM176A	transmembrane protein 176A	Other	other	
3,77E-03	2,42E+00	1,08E-01	2,456	0	Q9R1Q6	TMEM176B	transmembrane protein 176B	Other	other	
**9,99E-03**	**2,00E+00**	**9,99E-03**	**35**	**1**	**Q8BRG8**	**TMEM209**	**transmembrane protein 209**	**Other**	**other**	
**1,07E-04**	**3,97E+00**	**3,05E-02**	**4,519**	**0**	**Q9QY73**	**TMEM59**	**transmembrane protein 59**	**Plasma Membrane**	**peptidase**	
1,18E-03	2,93E+00	7,78E-02	7,186	0	Q80YX1	TNC	tenascin C	Extracellular Space	other	
9,66E-03	2,02E+00	1,44E-01	2,749	0	Q9WUU8	TNIP1	TNFAIP3 interacting protein 1	Nucleus	other	
2,89E-03	2,54E+00	9,52E-02	2,333	0	Q921T2	TOR1AIP1	torsin 1A interacting protein 1	Nucleus	other	
**9,99E-03**	**2,00E+00**	**9,99E-03**	**35**	**1**	**Q8BU11**	**TOX4**	**TOX high mobility group box family member 4**	**Nucleus**	**other**	
**9,99E-03**	**2,00E+00**	**9,99E-03**	**35**	**1**	**E9Q987**	**TRIM12C**	**tripartite motif containing 5**	**Cytoplasm**	**enzyme**	
**9,99E-03**	**2,00E+00**	**9,99E-03**	**35**	**1**	**Q99PP6**	**TRIM6-TRIM34**	**TRIM6-TRIM34 readthrough**	**Cytoplasm**	**other**	
**9,99E-03**	**2,00E+00**	**9,99E-03**	**35**	**1**	**Q78WZ7**	**TWISTNB**	**TWIST neighbor**	**Nucleus**	**other**	
8,79E-03	2,06E+00	1,42E-01	4,398	0	P26369	U2AF2	U2 small nuclear RNA auxiliary factor 2	Nucleus	other	
**9,99E-03**	**2,00E+00**	**9,99E-03**	**35**	**1**	**Q9DC60**	**UBIAD1**	**UbiA prenyltransferase domain containing 1**	**Nucleus**	**enzyme**	mitochondria
**9,99E-03**	**2,00E+00**	**9,99E-03**	**35**	**1**	**E9Q7L1**	**URB2**	**URB2 ribosome biogenesis homolog**	**Nucleus**	**other**	
9,58E-03	2,02E+00	1,44E-01	3,033	0	Q9CZJ1	UTP11	UTP11 small subunit processome component	Nucleus	other	
**9,99E-03**	**2,00E+00**	**9,99E-03**	**35**	**1**	**Q9JI13**	**UTP3**	**UTP3 small subunit processome component**	**Nucleus**	**other**	
9,07E-03	2,04E+00	1,42E-01	2,823	0	Q8R2N2	UTP4	UTP4 small subunit processome component	Nucleus	other	
6,86E-03	2,16E+00	1,33E-01	4,379	0	Q91XL3	UXS1	UDP-glucuronate decarboxylase 1	Cytoplasm	enzyme	mitochondria
2,28E-03	2,64E+00	8,88E-02	2,948	0	P29533	VCAM1	vascular cell adhesion molecule 1	Plasma Membrane	transmembrane receptor	
9,46E-04	3,02E+00	7,29E-02	2,459	0	P20152	VIM	vimentin	Cytoplasm	other	
**9,99E-03**	**2,00E+00**	**9,99E-03**	**35**	**1**	**Q8BGW2**	**WBP1L**	**WW domain binding protein 1 like**	**Other**	**other**	
3,48E-03	2,46E+00	1,03E-01	4,84	0	Q4VBE8	WDR18	WD repeat domain 18	Nucleus	other	
2,67E-03	2,57E+00	9,41E-02	4,586	0	Q3TAQ9	WDR36	WD repeat domain 36	Extracellular Space	other	
**2,48E-04**	**3,61E+00**	**4,18E-02**	**6,127**	**0**	**Q6ZQL4**	**WDR43**	**WD repeat domain 43**	**Nucleus**	**transcription regulator**	
1,16E-03	2,94E+00	7,78E-02	5,373	0	Q9DBH0	WWP2	WW domain containing E3 ubiquitin protein ligase 2	Cytoplasm	enzyme	
8,82E-04	3,05E+00	7,21E-02	3,197	0	Q9DCD2	XAB2	XPA binding protein 2	Nucleus	other	
**9,99E-03**	**2,00E+00**	**9,99E-03**	**35**	**1**	**B7ZMP1**	**Xpnpep3**	**X-prolyl aminopeptidase 3, mitochondrial**	**Cytoplasm**	**peptidase**	mitochondria
**9,99E-03**	**2,00E+00**	**9,99E-03**	**35**	**1**	**Q924Z6**	**XPO6**	**exportin 6**	**Cytoplasm**	**other**	
5,89E-03	2,23E+00	1,24E-01	2,028	0	Q9JKB3	YBX3	Y-box binding protein 3	Nucleus	transcription regulator	
4,30E-03	2,37E+00	1,11E-01	4,801	0	Q9DB43	ZFPL1	zinc finger protein like 1	Cytoplasm	other	
4,45E-03	2,35E+00	1,11E-01	4,79	0	O88291	ZNF326	zinc finger protein 326	Nucleus	transcription regulator	
