# Supplementary material for: Autophagy protein 5 controls flow-dependent endothelial functions
Source: Cell Mol Life Sci. 2023 Jul 18;80(8):210. doi: 10.1007/s00018-023-04859-9 (PMC10352428; doi:10.1007/s00018-023-04859-9)
Supplement: Supplementary file 7 — Supplementary file7 (PDF 65 KB) [file 18_2023_4859_MOESM7_ESM.pdf]

Supp Tables

Mice	*Atg5*$^{lox/lox}$	*Cdh5*.cre.*Atg5*$^{lox/lox}$
Body weight (g)	29.7±0+6	28.2±0.7
Heart rate (beat/min)	600±28	638±24
Heart-to-body weight ratio (%)	0.57±0.03	0.60±0.03
Kidney mean length (mm)	11.4±0.7	11.6±0.6
Kidney mean size (mm3)	274.9±19	283.5±19

Supp Table S6: Cdh5.cre-Atg5lox/lox mice physiological parameters

Mice	*Atg5*$^{lox/lox}$	*Cdh5.cre-Atg5*$^{lox/lox}$
Cardiac output (mL/min)	10.8±0.7	10.5±0.7
Shortening fraction (%)	34.6±1.1	35.8±0.9
IVSd (mm)	0.86±0.06	0.97±0.06
LVDd (mm)	4.01±0.14	3.89±0.10
LVPWd (mm)	0.85±0.07	0.86±0.03
IVSs (mm)	1.24±0.09	1.29±0.08
LVDs (mm)	2.62±0.08	2.47±0.08
LVPWs (mm)	1.10±0.07	1.15±0.04

Supp Table S7: Cdh5.cre-Atg5lox/lox mice cardiac function and parameters.

Mice	*Atg5*$^{lox/lox}$	*Cdh5.cre-Atg5*$^{lox/lox}$
Shortening fraction (%)	24.9±1.6	19.3±1.7 [a]
IVSd (mm)	0.77±0.06	0.77±0.06
LVDd (mm)	5.18±0.14	5.10±0.13

LVPWd (mm)	0.77±0.04	0.70±0.03
IVSs (mm)	1.07±0.11	0.97±0.08
LVDs (mm)	3.95±0.22	4.11±0.17
LVPWs (mm)	1.09±0.05	0.91±0.05 [a]

Supp Table S8: Cdh5.cre-Atg5lox/lox cardiac function and parameters after myocardial infarction.

IVS: Interventricular septum, LVD: Left ventricular dimension, LVPW: Left ventricular posterior wall, d:diastole, s:systole. Values are means ± SEM of n=9 mice, [a] $p<0.05$

Antigen	Species	Reactivity	Application	Reference	Provider
**AKT**	Rb	Hu/Ms	WB	4691	Cell Signaling Technology
**Phospho-AKT (ser473)**	Rb	Hu/Ms	WB	4060	Cell Signaling Technology
**anti-mouse IgG HRP conjugate**	Goat	Ms	WB	7076	Cell Signaling Technology
**anti-rabbit IgG HRP conjugate**	Goat	Rb	WB	7074	Cell Signaling Technology
**ATG16L1**	Rb	Hu/Ms	IF	8089	Cell Signaling Technology
**ATG5**	Rb	Hu/Ms	WB	12994	Cell Signaling Technology
**Beta-actin**	Rb	Hu/Ms	WB	A5441	Sigma-Aldrich
**BNIP3**	Rb	Hu	WB	44060	Cell Signaling Technology
**BNIP3L**	Rb	Hu/Ms	WB	12396	Cell Signaling Technology
**CDH5**	Goat	Ms	IF	AF1002	R&D Systems
**COX4**	Ms	Hu/Ms	WB	ab33985	Abcam
**eNOS**	Ms	Hu/Ms	WB	610296	BD biosciences
**eNOS**	Rb	Hu/Ms	WB	32027	Cell Signaling Technology
**GAPDH**	Ms	Hu/Ms	WB	AB2302	Millipore
**GAPDH**	Rb	Hu/Ms	WB	sc-25778	Santa cruz biotechnology
**GFP**	Rb		IF	Ab290	Abcam
**GFP**	Ms		IF	11814460001	Roche
**LC3B**	Rb	Hu/Ms	WB, IF	3868	Cell Signaling Technology
**MET**	Rb	Hu/Ms	WB	8198	Cell Signaling Technology
**OXPHOS**	Ms	Hu	WB	ab110411	Abcam
**PINK1**	Rb	Hu	WB	6946	Cell Signaling Technology
**Phospho-eNOS (ser1177)**	Ms	Hu/Ms	WB	612392	BD biosciences

**Phospho-eNOS (ser1177)**	Rb	Hu	WB	9570	Cell Signaling Technology
**Phospho-ERK1/2 (Thr202/Tyr204)**	Rb	Hu/Ms	WB	4370	Cell Signaling Technology
**Phospho-MET (Tyr1234-1235)**	Rb	Hu/Ms	WB	3077	Cell Signaling Technology
**Phospho-p38/MAPK (Thr180/Tyr182)**	Rb	Hu/Ms	WB	9211	Cell Signaling Technology
**Phospho-VEGFR2 (Tyr 1175)**	Rb	Hu/Ms	WB	3770	Cell Signaling Technology
**SDHA**	Ms	Hu/Ms	WB	ab14715	Abcam
**SDHB**	Rb	Hu/Ms	WB	HPA002868	Sigma Aldrich
**SQSTM1**	GP	Hu/Ms	IF	GP-62C	Progen
**Tubulin**	Rb	Hu/Ms	WB	ab6160	Abcam
**VEGFR2**	Goat	Ms	IF	AF644	R&D Systems
**VEGFR2**	Rb	Hu/Ms	WB/IF	9698	Cell Signaling Technology
**VEGFR2**	Goat	Hu	IF	AF357	R&D Systems

Supp Table S9: Antibodies
