# Supplementary material for: Autophagy protein 5 controls flow-dependent endothelial functions
Source: Cell Mol Life Sci. 2023 Jul 18;80(8):210. doi: 10.1007/s00018-023-04859-9 (PMC10352428; doi:10.1007/s00018-023-04859-9)
Supplement: Supplementary file 8 — Supplementary file8 (DOCX 33 KB) [file 18_2023_4859_MOESM8_ESM.docx]

**Supplementary Methods**

**Bleeding assay**

Mice were anesthetized with Ketamine-Xylazine and weighed on a precision balance. The tail was then cut with a scalpel 2mm from the end and placed in a 50mL Falcon tube with warm (37°C) PBS. Once the tail was in PBS, the timer was immediately started. Bleeding time was measured as the time from when the tail was placed in the tube until no more blood flowed from the wound to the tail. Bleeding was stopped after 20min if the bleeding did not stop spontaneously. Mice were then weighed again on the precision balance with the tail piece removed, and the weight of blood lost was calculated as the initial weight minus the weight after bleeding.

**Retinas immunofluorescence staining:**

Retinas were isolated from Atg5^lox/lox^ and Cdh5.Cre Atg5^lox/lox^ mice following intracardial injection of 2% PFA prepared in 1x PBS^Ca2+Mg2+^. Retinas were stained with FITC-coupled isolectin B4 overnight at 4°C. Tissues were washed and mounted on slides in fluorescent mounting medium (Dako). Images were acquired using a Leica TCS SP8 confocal microscope.

***Angio microscanner***

Each mouse received an intraperitoneal injection of an anticoagulant solution (heparin Choay®), 300 IU/kg), and, 2 hours later, received an infusion of isosorbide dinitrate (Risordan®, Medisol) to induce vasodilation. The mouse was then euthanized by sodium pentobarbital (Exagon®, Axience). A median sternotomy was performed and the trunk of the brachiocephalic artery was cannulated under binocular control. Once a right draining atriotomy had been done, the vasculature was washed out under a pressure of 80 mmHg with an isotonic NaCl solution containing heparin and isosorbide dinitrate. When the washing solution became clear, a second infusion, at the same pressure, with 4% formaldehyde (neutral buffered formalin 10%, DiaPath), was made to fix the tissues. A mixture of 80% Neoprene Latex (Neoprene Latex Dispersion 671 A, Dupont, France) and barium sulphate powdered to 1 μm (3 g/mL, MicrOpaque® oral solution, Guerbet, France) in distilled water was then injected gradually under the same pressure, ensuring perfusion of arteries and arterioles down to a diameter of 20 µm. The animal was then put into formic acid for the latex to polymerize, and, after dissection, the kidneys were fixed overnight in 4% paraformaldehyde at 4°C.

The kidneys were scanned with a high-resolution micro-CT imaging system (Skyscan® 1174, Brucker). The microscanner consists of an X-ray source (50 KV, 800 µA) and a 1.3 million-pixel CCD camera coupled to a P43 scintillator for detection. The acquisition protocol consisted of 360 views (180° rotation with 0.5°step) with a voxel volume of 14.4x14.4x14.4 μm^3^. At each step, 9 images are acquired and averaged to obtain a single image with minimal noise. The 2D radiographs were then processed using the NRecon® software (Brucker) for 256 gray level 3D image reconstruction. For extraction of the 2 structures of interest (whole kidney and kidney arterial vasculature), a segmentation technique based on 2 grayscale-defined thresholds was applied. For each kidney, the threshold values were determined by statistical analysis of the frequency distribution of the pixel grayscale values and Gaussian non-linear regression of the pixel population corresponding to non-perfused renal tissues, using Prism® software (GraphPad). In the image of the arterial network obtained after segmentation, a median filter, set at 3 pixels, was applied to remove isolated pixels due to background digital noise, and then the skeletonization of the vascular network image was performed, using FIJI® software. The image of the skeleton was then fused with the image of the vascular network, associating each segment of the skeleton, identified by its orthonormal coordinates, with its 3D image. Distance mapping, allowing determining the mean radius of each segment, was then combined with skeleton analysis to obtain, for each segment, its length, and diameter.

The density of the vascular network, expressed in %, was defined as the ratio of the volume of the arterial network on the volume of the whole kidney. Both volumes were calculated using Imaris® software on the images obtained after segmentation and filtering. The fractal dimension of the arterial network was obtained from the skeletonized image of each kidney vasculature by the “box-counting” Minkowski-Bouligand method using FIJI® software. Total vessel length was calculated by summing the lengths of the segments. Vascular density, fractal dimension and total vessel length were compared between the 2 samples using Mann-Whitney statistical test (Prism® software). Lengths and diameters of the vascular segments of each kidney were analyzed by nonlinear regression of their frequency distribution. For this, frequency classes were defined, and the percentage of branches belonging to each class was calculated. The frequency distribution thus obtained was adjusted by nonlinear regression with a decreasing first-order exponential equation for segment lengths and a log Gaussian equation for diameters and then compared between samples by non-linear regression analysis and Fisher’s F test (Prism® software).

## Femoral Artery Injury

Intra-luminal wire injury of the left femoral artery was performed as described ^1^. Briefly, mice were anesthetized using isoflurane inhalation, with buprenorphine administered to ensure postoperative analgesia. A straight-sprung angioplasty guidewire (0.014” diameter) was advanced ~1.5cm into the femoral artery via the popliteal branch. After 30 seconds, the guidewire was withdrawn and the popliteal branch ligated to allow reperfusion of the injured femoral artery. Mice were allowed to recover for 21 days before trans-cardiac perfusion fixation under terminal anesthesia (pentobarbital). The left femoral arteries were then isolated for analysis.

Femoral arteries were processed for OPT, as described ^2^. Each artery segment was briefly washed in phosphate-buffered saline and embedded in an agarose gel column (1.5% agarose in H_2_O). The samples were dehydrated in methanol, optically cleared with BABB (Benzyl benzoate: Benzyl alcohol, 2:1), and scanned using Bioptonics 3001 OPT tomograph (Skyscan, Belgium). To describe the overall neointima formation, longitudinal lesion distribution and total neointimal volume in the first 1.2mm segment of the artery were analyzed using CTAn software (Skyscan, Belgium). Following OPT, the arteries were processed to paraffin for histology, sectioned and stained using the United States trichrome as described ^3^.

**Lentiviral transduction of HUVEC cells with sh-ATG7**

HUVECs (Promocell) were cultured in ECGM medium (Promocell) with 1% penicillin/streptomycin (Gibco). Production of HIV1 delta U3 SIN lentiviral particles with VSV-G envelop was carried out by the VVTG facility platform (Necker faculty) by using the lentiviral vector pLVTH (Addgene), containing ATG7-specific shRNA (small-hairpin RNA):

GATCCCCGGAGTCACAGCTCTTCCTTTTCAAGAGAAAGGAAGAGCTGTGACTCCTTTTTAAGCT, and GFP.

**Orbital shear-stress :**

HUVECs were seed in 6-well plates until confluence. Cells were then starved in EBM medium without supplements for 6 hours and placed on an orbital shaker (VWR microplate shaker, radius of the orbit =1.5mm) at 210 rpm for 10 minutes at 37°C then cells were washed in ice cold PBS and protein were extracted in RIPA buffer containing phosphatase and protease inhibitors. This condition of orbital rotation was shown to generate low shear stress in the centre of the well and high shear stress at the periphery ^4-6^.

**Plasma membrane isolation**

Plasma membrane proteins were isolated using a plasma membrane protein extraction kit (ab65400, Abcam) according to manufacturer’s protocol. Plasma membrane pellets was diluted in RIPA buffer containing phosphatase and protease inhibitors and kept at -80°C before gel loading.

1. Sata, M. *et al.* A mouse model of vascular injury that induces rapid onset of medial cell apoptosis followed by reproducible neointimal hyperplasia. *J Mol Cell Cardiol* **32**, 2097-2104 (2000).

2. Kirkby, N.S. *et al.* Quantitative 3-dimensional imaging of murine neointimal and atherosclerotic lesions by optical projection tomography. *PLoS One* **6**, e16906 (2011).

3. Hadoke, P., Wainwright, C.L., Wadsworth, R.M., Butler, K. & Giddings, M.J. Characterization of the morphological and functional alterations in rabbit subclavian artery subjected to balloon angioplasty. *Coron Artery Dis* **6**, 403-415 (1995).

4. Dardik, A. *et al.* Differential effects of orbital and laminar shear stress on endothelial cells. *J Vasc Surg* **41**, 869-880 (2005).

5. Mahmoud, M.M. *et al.* Shear stress induces endothelial-to-mesenchymal transition via the transcription factor Snail. *Sci Rep* **7**, 3375 (2017).

6. Warboys, C.M. *et al.* Disturbed flow promotes endothelial senescence via a p53-dependent pathway. *Arterioscler Thromb Vasc Biol* **34**, 985-995 (2014).
