# Supplementary material for: Autophagy protein 5 controls flow-dependent endothelial functions
Source: Cell Mol Life Sci. 2023 Jul 18;80(8):210. doi: 10.1007/s00018-023-04859-9 (PMC10352428; doi:10.1007/s00018-023-04859-9)
Supplement: Supplementary file 9 — Supplementary file9 (PDF 6097 KB) [file 18_2023_4859_MOESM9_ESM.pdf]

Fig 1C

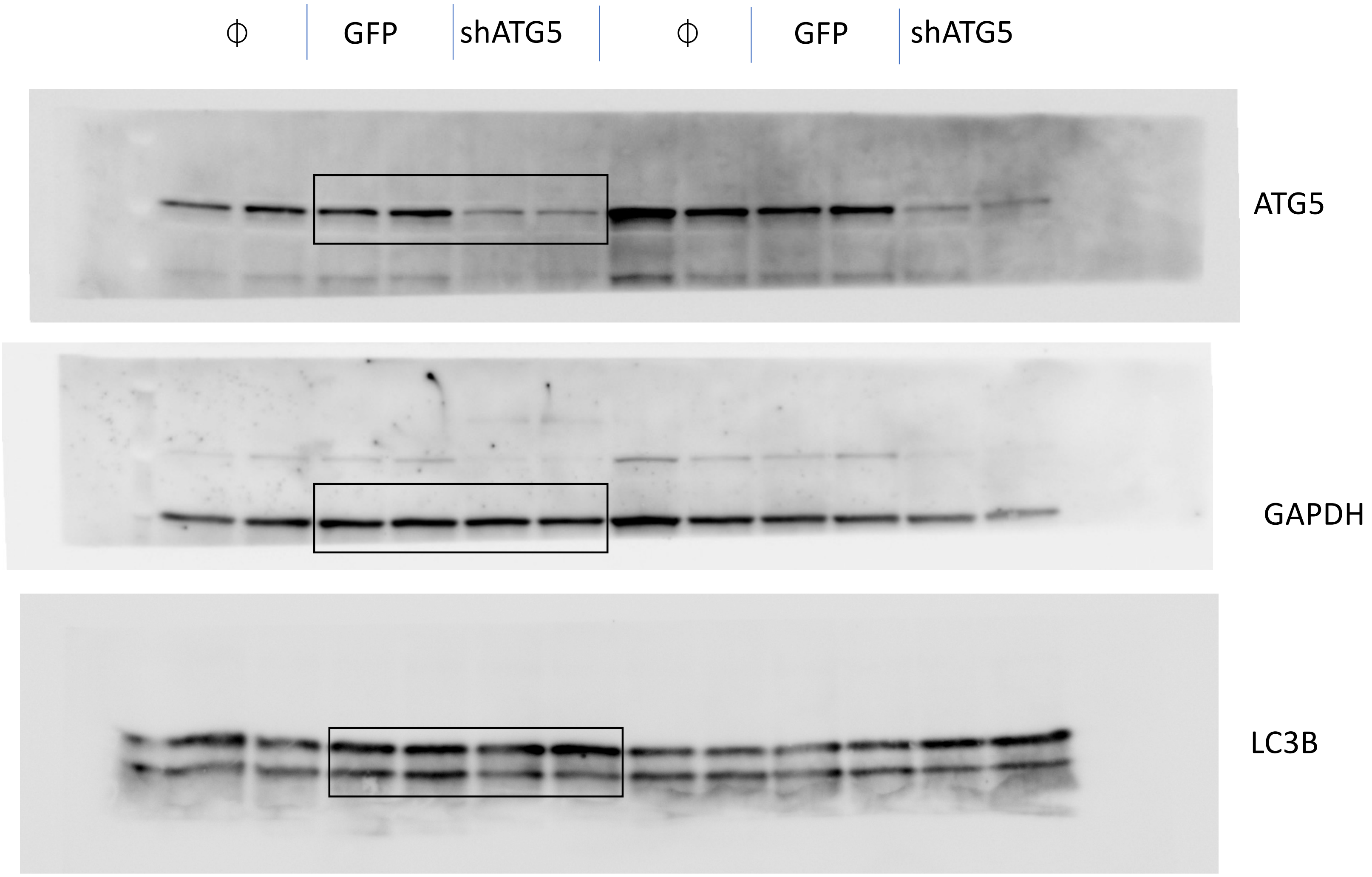

Fig 1F

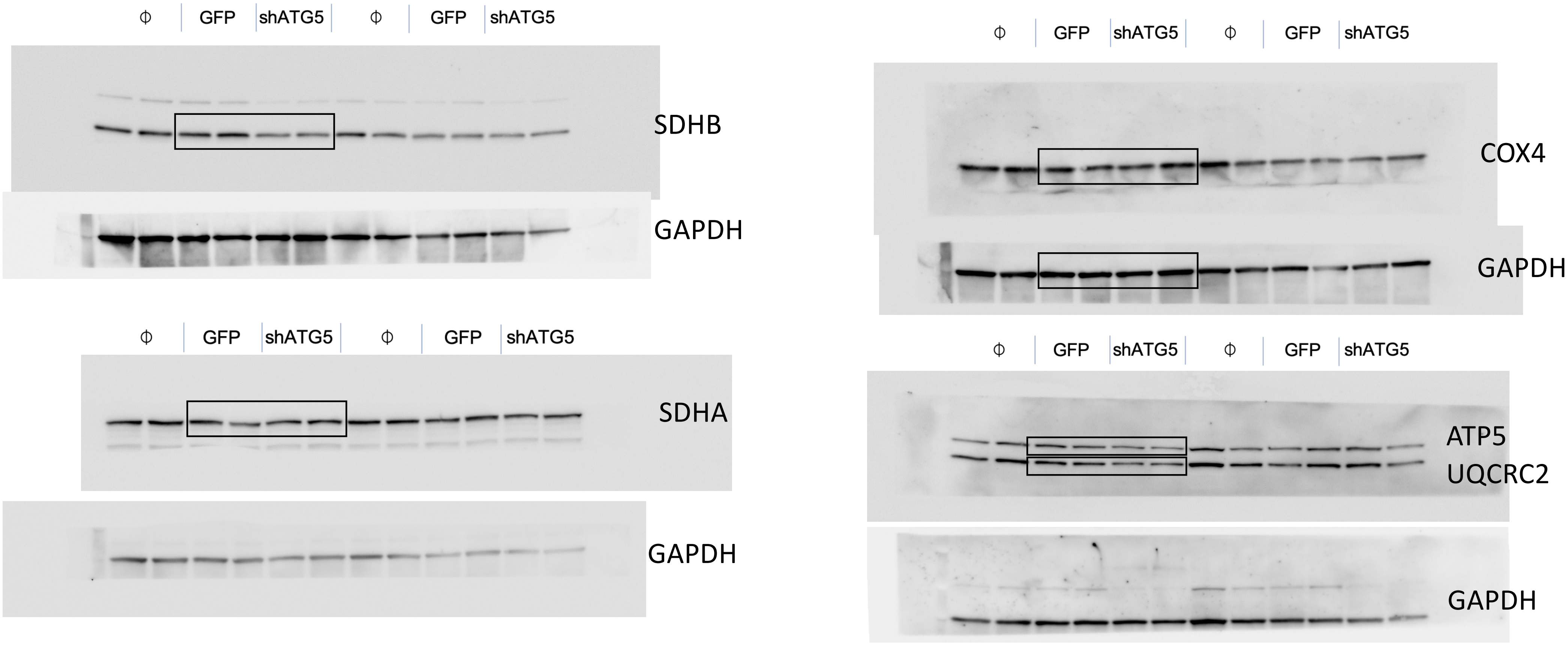
Φ
GFP
shATG5
Φ
GFP
shATG5
SDHB
GAPDH
Φ
GFP
shATG5
Φ
GFP
shATG5
SDHA
GAPDH
Φ
GFP
shATG5
Φ
GFP
shATG5
COX4
GAPDH
Φ
GFP
shATG5
Φ
GFP
shATG5
ATP5
UQCRC2
GAPDH

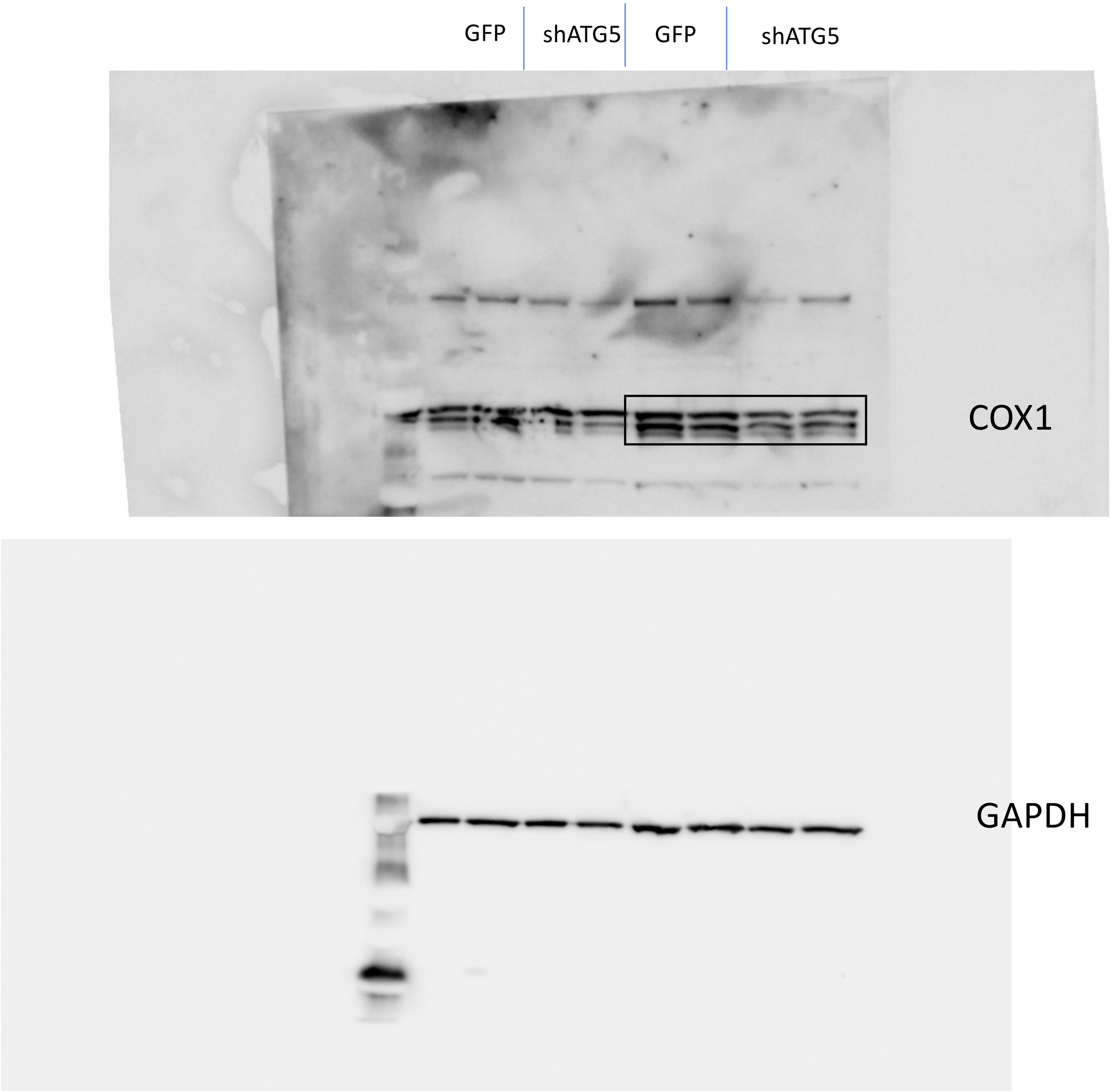
GFP
shATG5
GFP
shATG5
COX1
GAPDH

Fig 2D

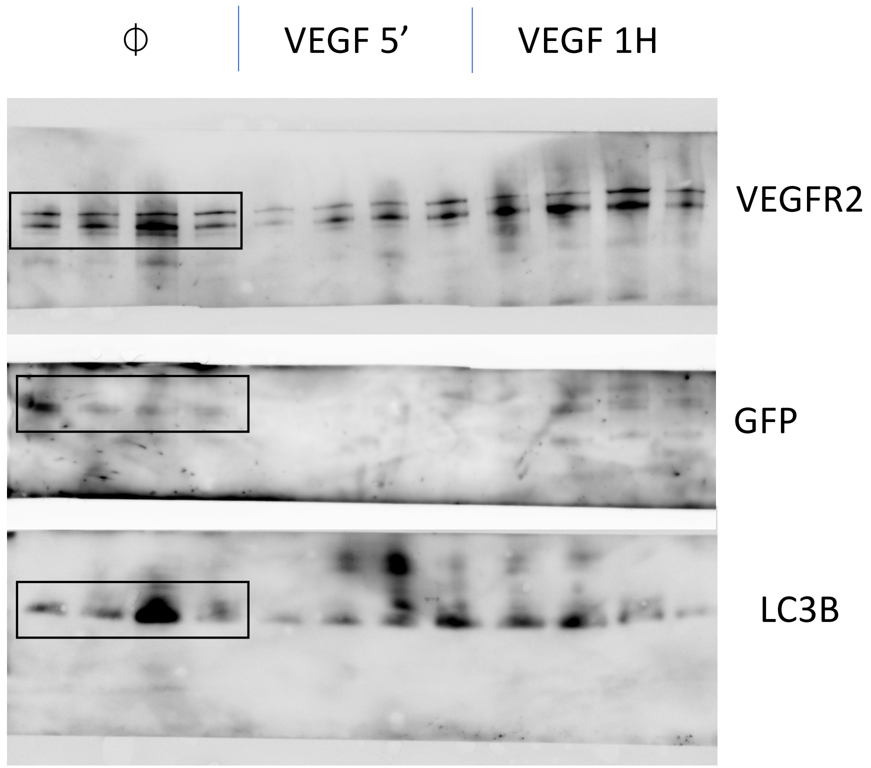

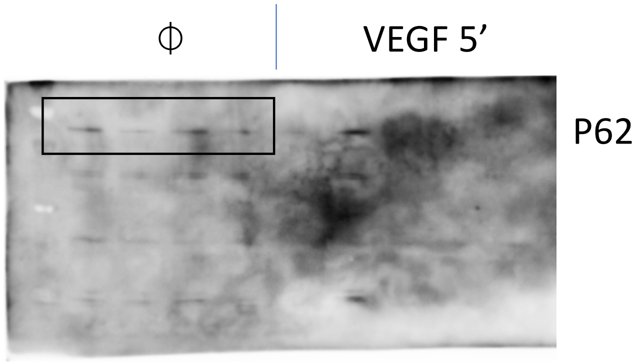

Original image for Fig2E has been lost in a computer crash

Fig 3E

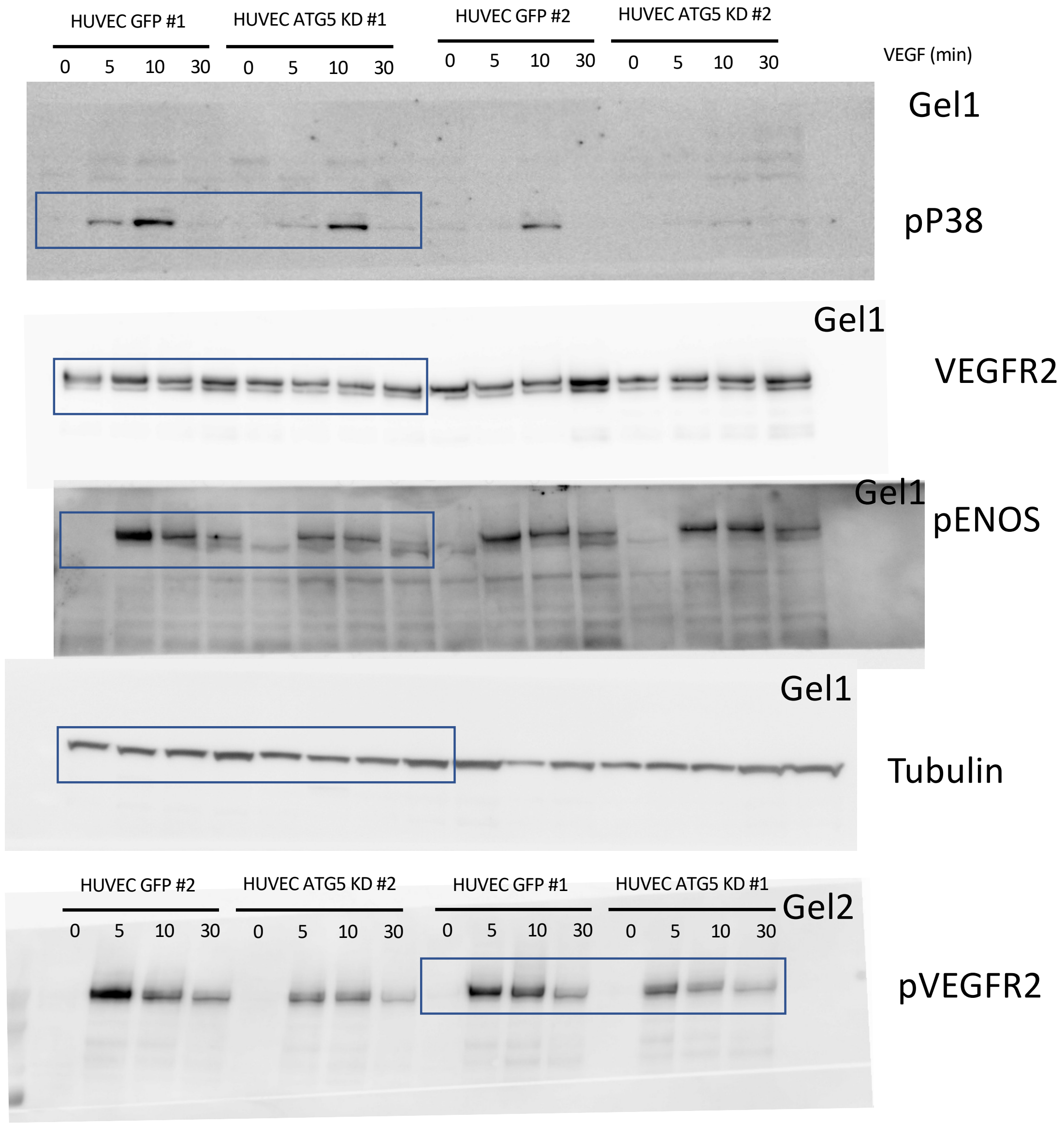

HUVEC GFP #1
HUVEC ATG5 KD #1
HUVEC GFP #2
HUVEC ATG5 KD #2
0 5 10 30 0 5 10 30 0 5 10 30 0 5 10 30
VEGF (min)
Gel1
pP38
Gel1
VEGFR2
Gel1
pENOS
Gel1
Tubulin
HUVEC GFP #2
HUVEC ATG5 KD #2
HUVEC GFP #1
HUVEC ATG5 KD #1
0 5 10 30 0 5 10 30 0 5 10 30 0 5 10 30
Gel2
pVEGFR2

Fig 5K

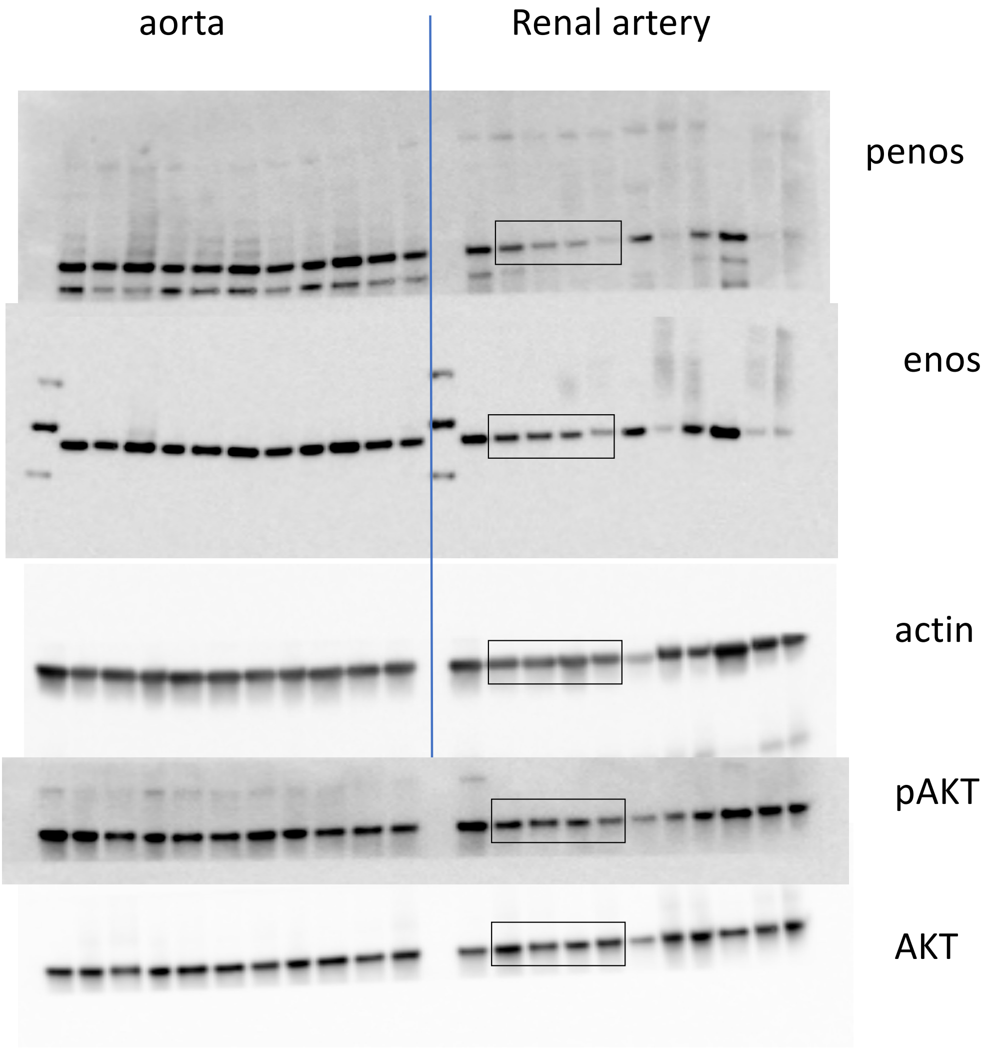

3WT 3KO 1WT 2KO 1WT 1KO

Supp Figure S3

Lung primary endothelial cells from atg5 lox/lox (WT) or cdh5.cre atg5lox/lox (KO) mice

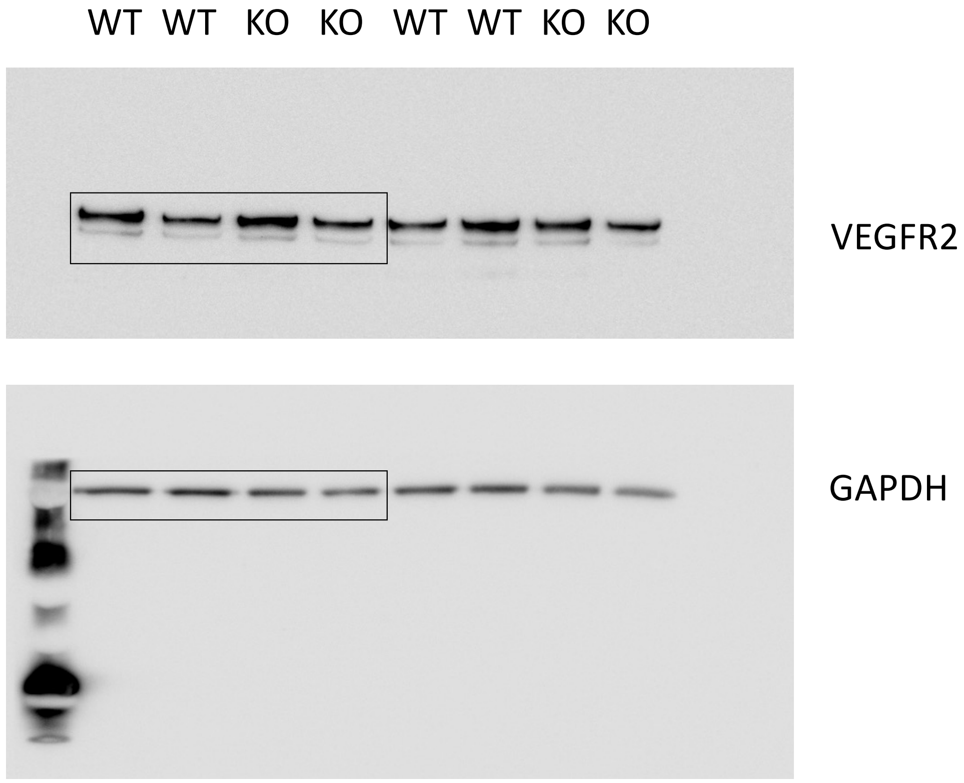

Supp Figure S3

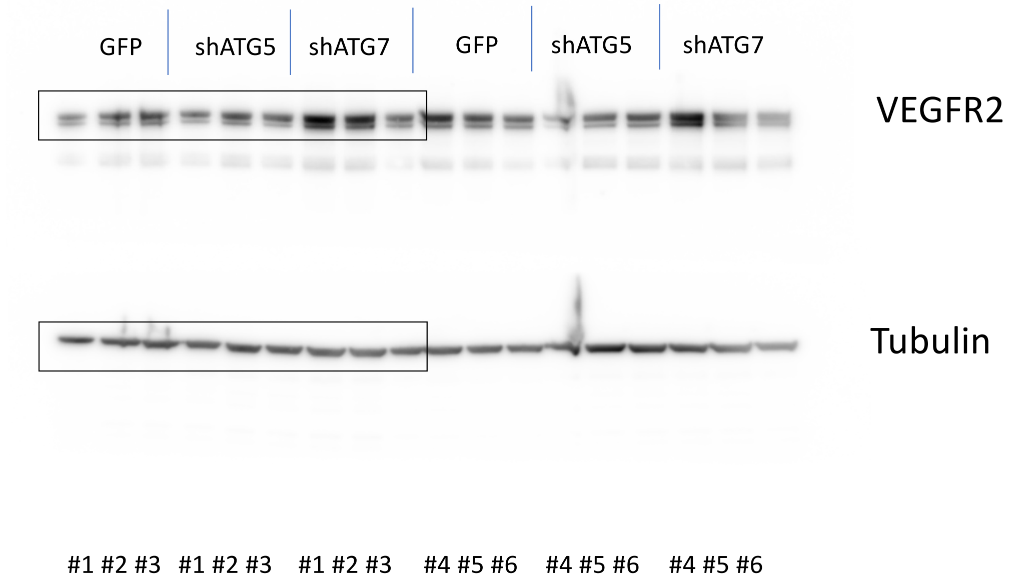

Supp Figure S3

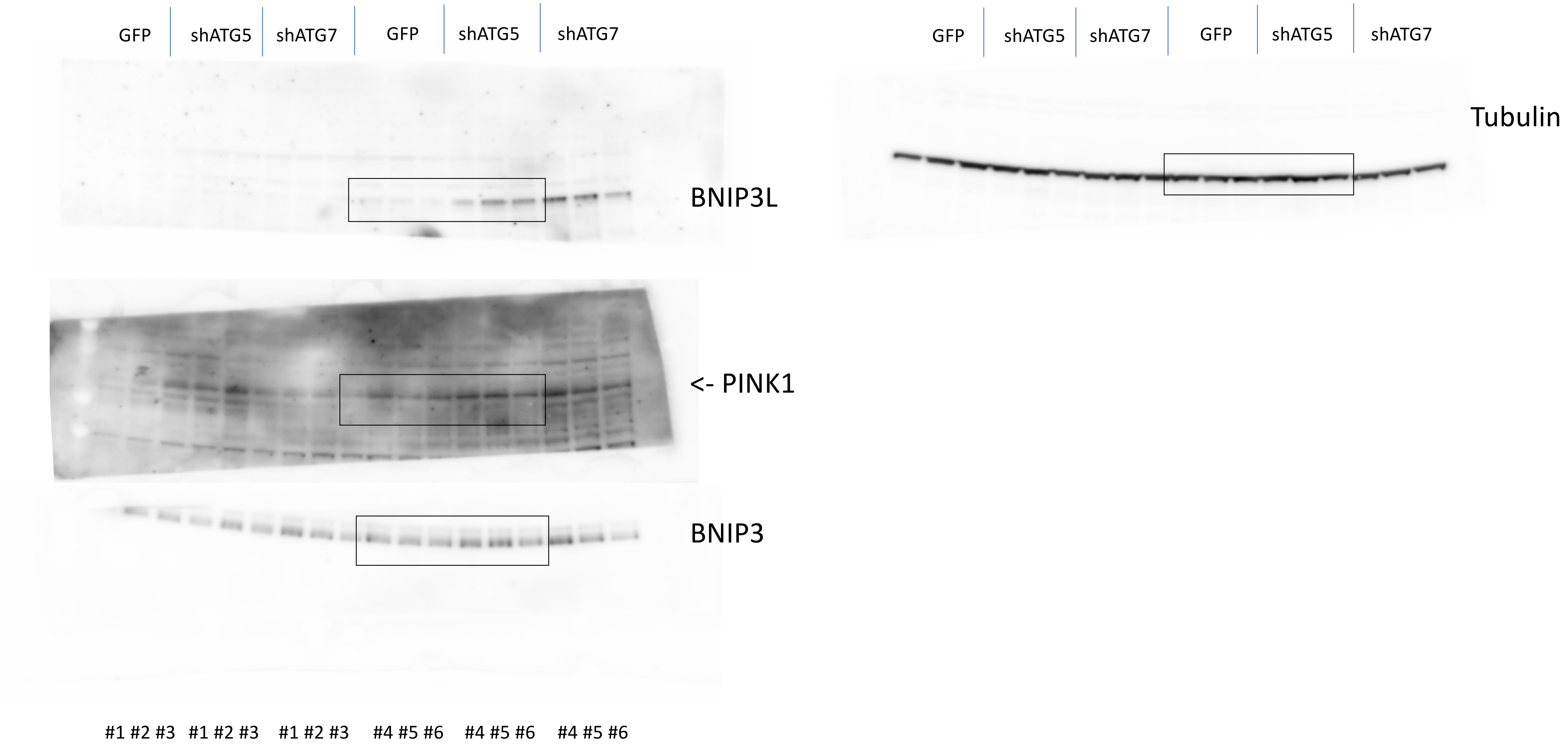

Supp Figure S4 E

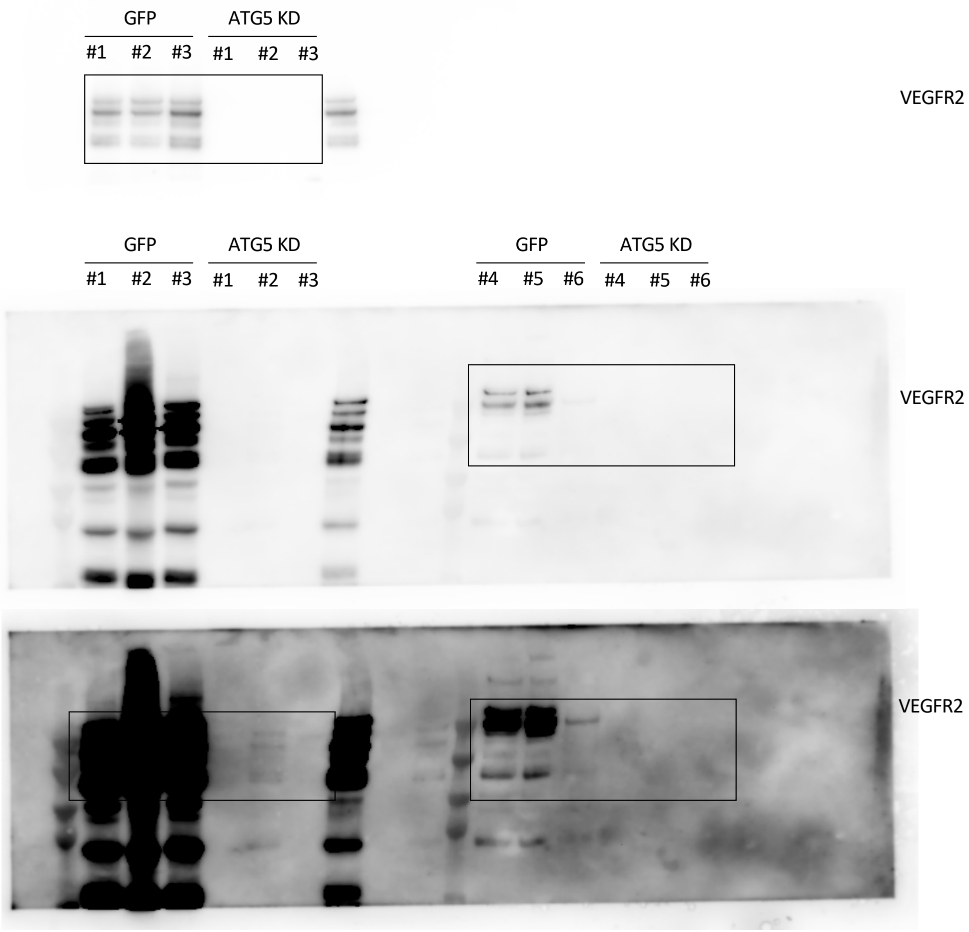

Supp Fig S5

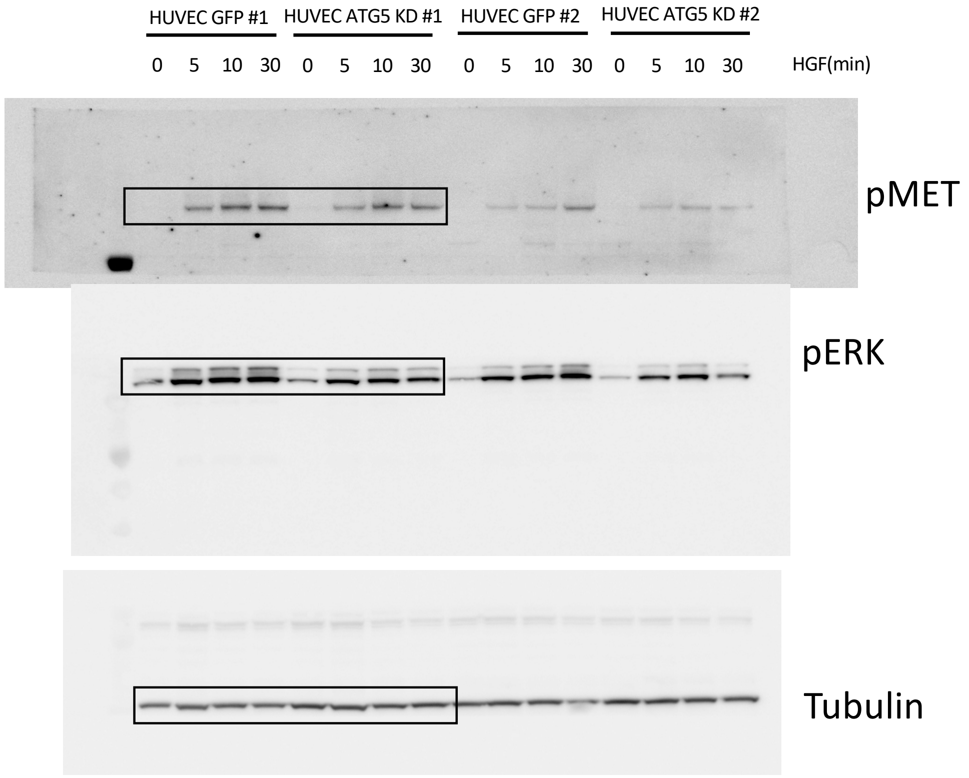
HUVEC GFP #1
HUVEC ATG5 KD #1
HUVEC GFP #2
HUVEC ATG5 KD #2
0 5 10 30 0 5 10 30 0 5 10 30 0 5 10 30
HGF(min)
pMET
pERK
Tubulin

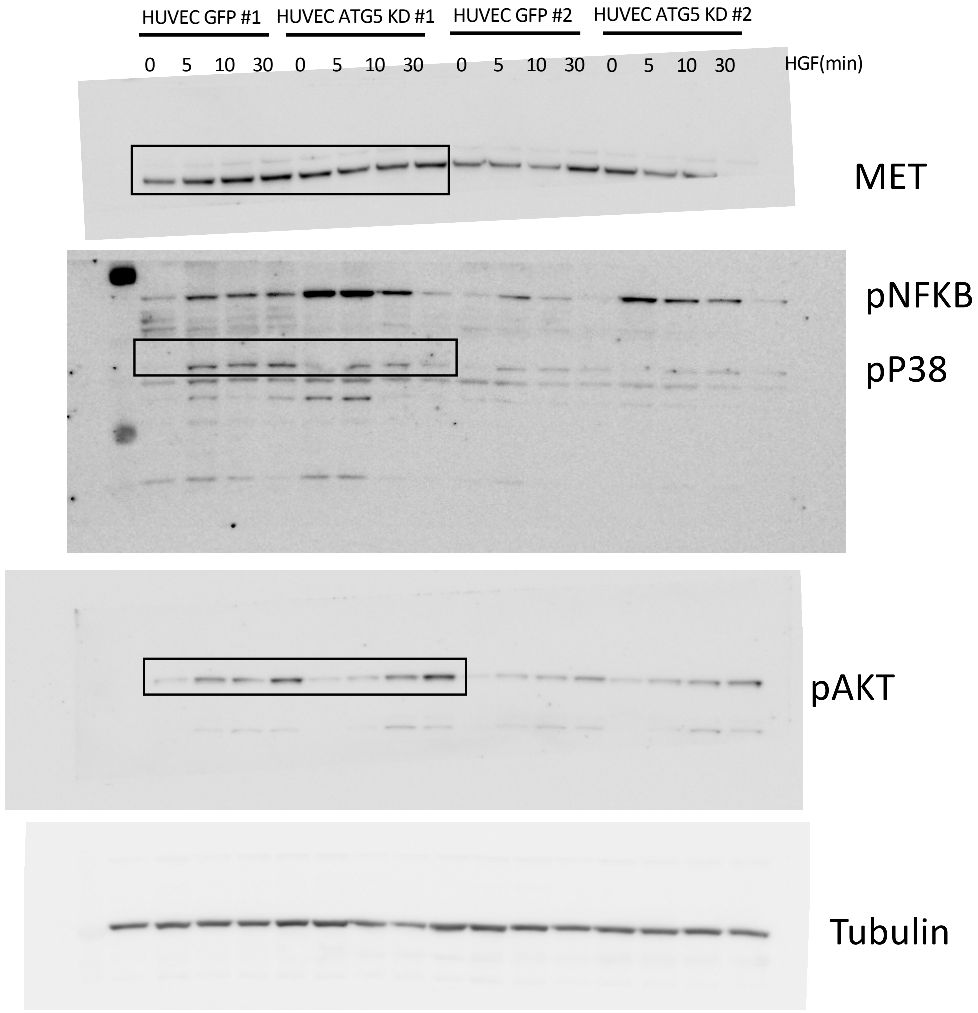
HUVEC GFP #1
HUVEC ATG5 KD #1
HUVEC GFP #2
HUVEC ATG5 KD #2
0 5 10 30 0 5 10 30 0 5 10 30 0 5 10 30
HGF(min)
MET
pNFKB
pP38
pAKT
Tubulin

Supp Fig S5

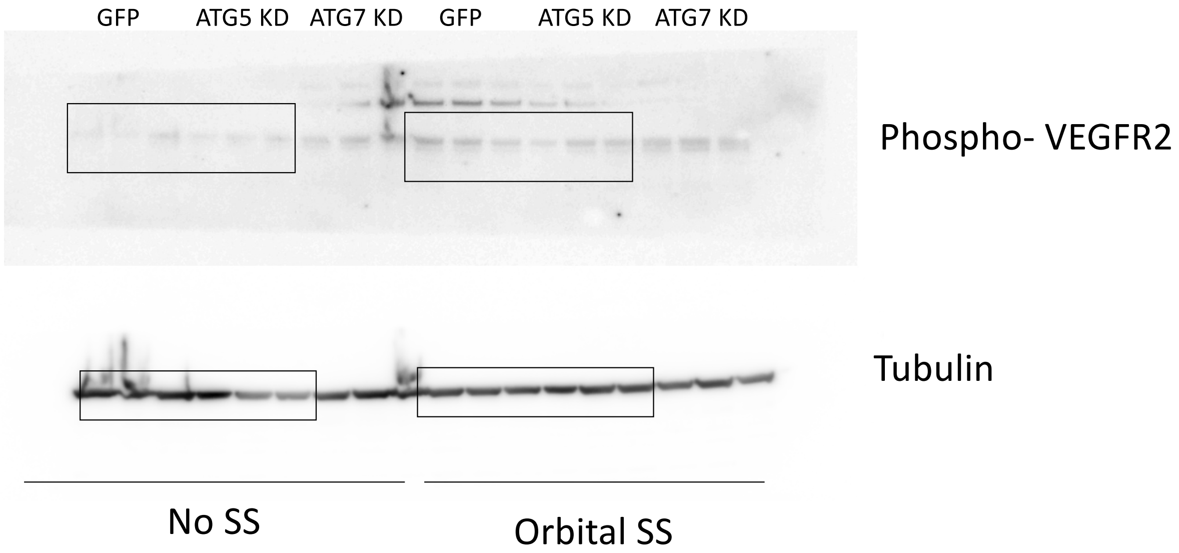
GFP
ATG5 KD
ATG7 KD
GFP
ATG5 KD
ATG7 KD
Phospho- VEGFR2
Tubulin
No SS
Orbital SS

Supp Figure S9

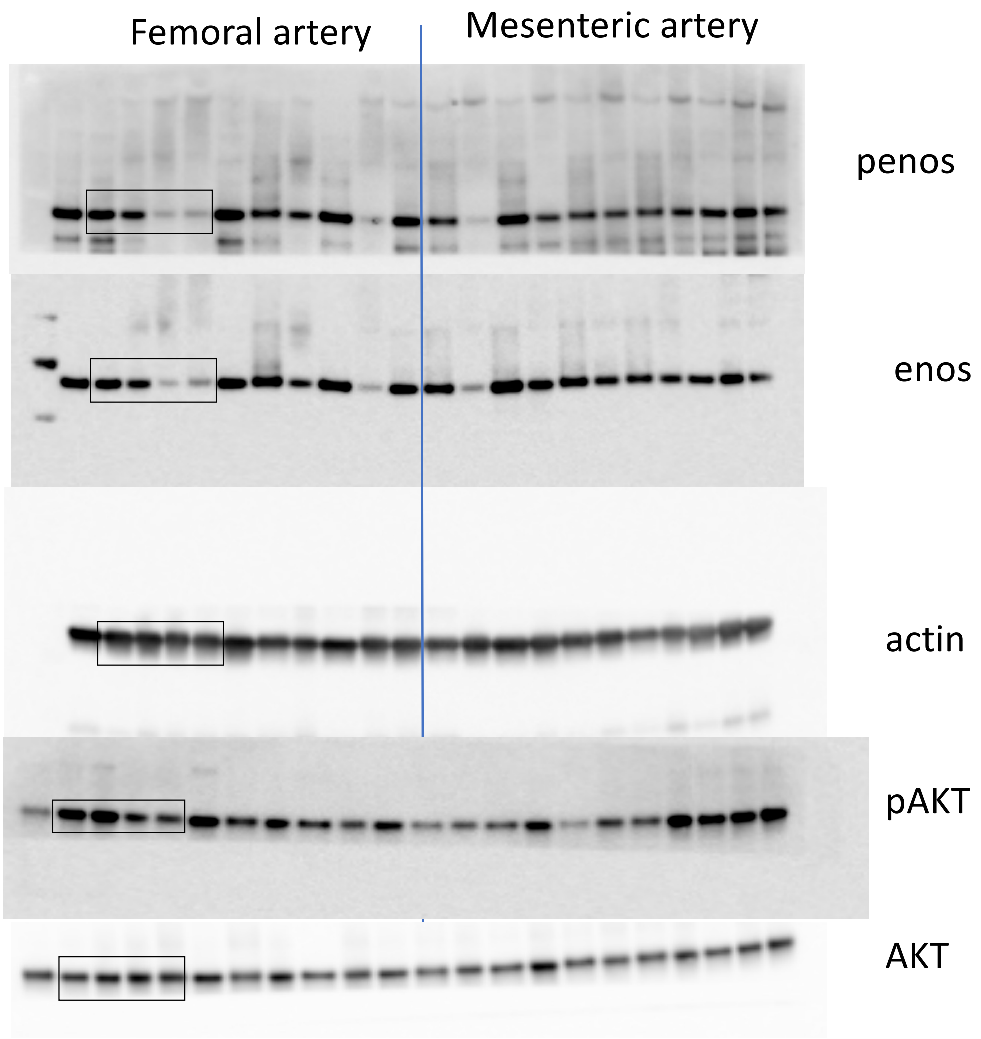

3WT 3KO 1WT 2KO 1WT 1KO
